# Parallel Synthesis of an Imidazole-4,5-dicarboxamide Library Bearing Amino Acid Esters and Alkanamines

**DOI:** 10.3390/molecules13123149

**Published:** 2008-12-15

**Authors:** Rosanna Solinas, John C. DiCesare, Paul W. Baures

**Affiliations:** Department of Chemistry and Biochemistry, The University of Tulsa, 800 South Tucker Drive, Tulsa, OK 74104, USA; E-mails: *rosannasolinas@yahoo.it* (R. S.); *john-dicesare@utulsa.edu* (J-C. D.)

**Keywords:** Imidazole, NIH Roadmap, Heterocyclic scaffold, Drug discovery.

## Abstract

The imidazole-4,5-dicarboxylic acid scaffold is readily derivatized with amino acid esters and alkanamines to afford compounds with intramolecularly hydrogen bonded conformations that mimic substituted purines and therefore are hypothesized to be potential inhibitors of kinases through competitive binding to the ATP site. In this work, a total of 126 dissymmetrically disubstituted imidazole-4,5-dicarboxamides with amino acid ester and alkanamide substituents were prepared by parallel synthesis. The library members were purified by column chromatography on silica gel and the purified compounds characterized by LC-MS with LC detection at 214 nm. A selection of the final compounds was also analyzed by ^1^H-NMR spectroscopy. The analytically pure final products have been submitted to the Molecular Library Small Molecule Repository (MLSMR) for screening in the Molecular Library Screening Center Network (MLSCN) as part of the NIH Roadmap.

## Introduction

There remains a need for selective small molecule probes of biological processes in order to discern cellular communication and signaling pathways, as well as for potential application as therapeutic agents against diseases [1,2,3,4]. In general, the most useful biological probes will also meet the parameters commonly found for drugs or drug-like molecules, including Lipinski’s “rule of five” [5] and the number of rotatable bonds [6], since such parameters are indications of solubility and cellular permeability [7]. There is nonetheless a growing awareness that biological probes or chemical tools will often violate one or more of these rules, but that this does not necessarily void the usefulness of the compounds to medicinal research [8].

Imidazole-4,5-dicarboxylic acid (I45DA) is a scaffold that is readily derivatized with amines to yield imidazole-4,5-dicarboxamides (I45DCs) [9]. Primary amines and amino acid esters retain an N-H bond in the final compound that forms a strong intramolecular hydrogen bond [9,10,11,12]. The quasi ring, in combination with the imidazole and substituents, then affords a product that is a reasonable mimic of a substituted purine [9], an important class of small molecule biological probes [13].

**Figure 1:**
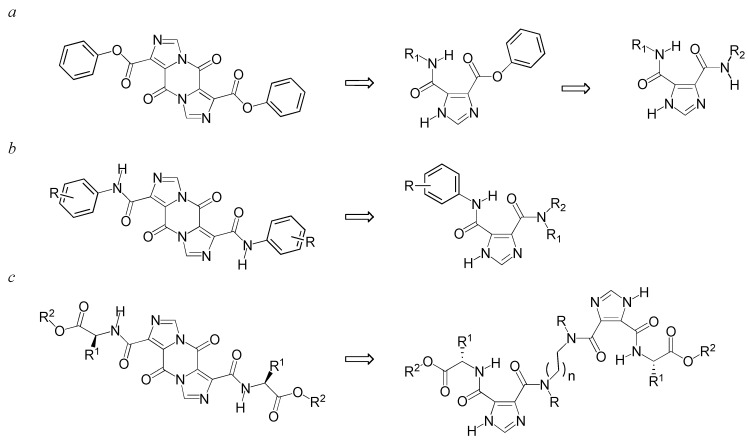
The synthetic intermediates to dissymmetrically disubstituted imidazole-4,5-dicarboxamides, including those bearing *a*) different primary alkanamines, *b*) anilines with alkanamies or amino acid esters, as well as *c*) amino acids esters and alkanamines leading to bis-I45DCs.

We have previously reported on methods to synthesize dissymmetrically disubstituted imidazole-4,5-dicarboxamides (dI45DCs) with different primary alkanamides [14], anilines with either alkanamines or amino acid esters [15], or combinations of amino acid esters and alkanediamines to create bis-I45DCs [16]. The synthetic strategies to these I45DC derivatives are different (Figure 1). The dI45DCs with only alkanamine substituents proceed through an imidazole phenyl ester-carboxamide substituted intermediate [14], while the dI45DCs substituted with an aniline and bis-I45DCs are synthesized from aniline and amino acid ester substituted pyrazines [15,16], respectively. 

The library reported herein combines amino acid ester substituents with alkanamines to produce dI45DCs where no structural examples have been previously reported in the literature. In the process of completing this library we have also improved on the synthetic methods to yield the amino acid ester pyrazine intermediates. The final compounds are all of analytical purity and have been submitted to the Molecular Library Small Molecule Repository (MLSMR) for use in the Molecular Library Screening Center Network (MLSCN) as part of the NIH Roadmap [2,17]. We have included tables of data (formula, molecular weight, clogP values, physical form of the compound, *R_f_* values, and retention times in the LC-MS) for library members **5**{1-126}, LC-MS spectra for all library members, LC-MS data for 18 reactions to crude library members, as well as ^1^H-NMR spectra for the crude reactions and purified library members for 18 representative compounds in a supporting file. The same information is also available for each compound at the project website [18]. Additional synthetic efforts that are part of the NIH Roadmap are now being reported [19,20,21,22,23,24,25,26,27,28,29,30,31].

## Results and Discussion

The building blocks for this library were selected as a minimal set that would maximize chances for identifying biologically active leads through the screens conducted by the MLSCN. All of the dI45DCs in this library are expected to favor an intramolecular hydrogen bonded conformation by analogy to similar compounds, and thereby the presentation of the building blocks in space is predisposed [9,10,11,12]. Thus, the overall shape of the library members is similar and the selected library building blocks vary the size, electrostatic, and hydrophobic characteristics of the library members within a range of values that are considered “drug-like” [5,6]. 

**Table 1:** Amino acid ester salts, **2**{*1*-*9*}, used in library synthesis.

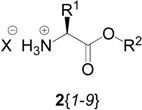			
member	R^1^	R^2^	X 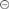
**2**{*1*}	H	C(CH_3_)_3_	Cl
**2**{*2*}	H	CH_2_Ph	Cl
**2**{*3*}	CH_3_	C(CH_3_)_3_	Cl
**2**{*4*}	CH_3_	CH_2_Ph	Cl
**2**{*5*}	CH_2_CH(CH_3_)_2_	C(CH_3_)_3_	Cl
**2**{*6*}	CH_2_CH(CH_3_)_2_	CH_2_Ph	OSO_2_C_6_H_4_CH_3_
**2**{*7*}	CH_2_Ph	C(CH_3_)_3_	Cl
**2**{*8*}	CH_2_Ph	CH_2_Ph	Cl
**2**{*9*}	(CH_2_)_4_NH_2_	C(CH_3_)_3_	Cl

**Table 2:** Alkanamines, **4**{*1*-*14*}, used in library synthesis.

HNR^3^R^4^		
member	R^3^	R^4^
**4**{*1*}^‡^	H	CH_3_
**4**{*2*}	H	(CH_2_)_4_NH-Boc
**4**{*3*}	H	CH_2_Ph
**4**{*4*}	H	*R*-CH(CH_3_)Ph
**4**{*5*}	H	*S*-CH(CH_3_)Ph
**4**{*6*}	H	[CH(CH_2_CH_2_)_2_N-Boc
**4**{*7*}^‡^	H	CH_2_C_6_H_4_CH_2_NH-Boc
**4**{*8*}	H	CH(Ph)_2_
**4**{*9*}	CH_3_	CH_2_Ph
**4**{*10*}	CH_2_CH_3_	CH_2_CH_3_
**4**{*11*}	(CH_2_CH_2_)_2_N-Boc	
**4**{*12*}	(CH_2_)(CH_2_CH_2_)C_6_H_4_	
**4**{*13*}	(CH_2_CH_2_)_2_CHCH_3_	
**4**{*14*}^‡^	(CH_2_CH_2_)_2_N-Ph	

^‡^These alkanamines were used as their hydrochloride salts.

The amino acid ester variations (Table 1) included no side chain (Gly, **2**{1,2}), a smaller (Ala, **2**{3,4}) and larger (Leu, **2**{5,6}) aliphatic side chain, and an aromatic side chain (Phe, **2**{7,8}) all with either a *tert*-butyl ester or benzyl ester, as well as a Boc protected polar side chain (Lys, **2**{9}) with a *tert*-butyl ester. Likewise, the alkanamine building blocks (Table 2) include a small and large aliphatic chain, aromatic amines with or without chirality, cyclic amines, and both primary and secondary amines. These selected building blocks create dI45DC library members with drug-like properties as illustrated by their molecular weights that range from 282−612 g/mol and an average value of 450 g/mol, as well as their cLogP values that range from -0.88−4.67 and an average value of 2.62.

**Scheme 1:**
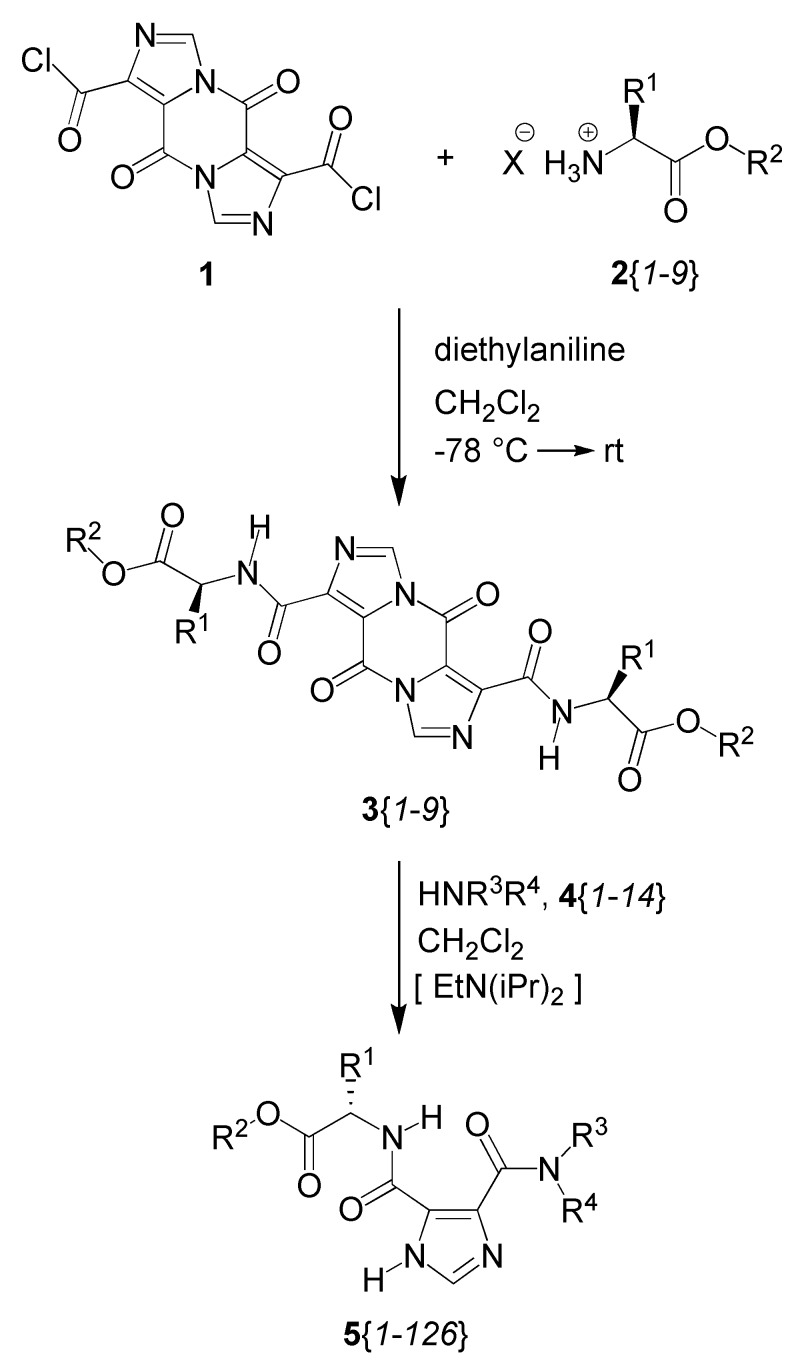
Synthetic Strategy to Library Members.

The synthetic strategy to the pyrazine intermediates and final dI45DCs is shown in Scheme 1. The starting pyrazine diacid chloride, **1**, was prepared as previously described [8]. Reaction of **1** with two equivalents of amino acid ester hydrochloride or tosylate salt, **2**{1-9}, and four equivalents of *N*,*N*-diethylaniline as a scavenger for the acid in the reaction produced good to excellent yields (76-93%) of the intermediate amino acid ester substituted pyrazines **3**{1-9} (Table 3). The reactions between these intermediates and amines **4**{1-14} were performed in culture tubes in parallel with diisopropylethyl-amine added to those reactions with an alkanamine hydrochloride salt, **4**{1,7,14}. Two equivalents of alkanamine (or alkanamine hydrochloride with the tertiary amine base) were used except in the case of methylamine hydrochloride, **4**{1}, where a total of four equivalents were used due to the increased volatility of the free alkanamine. The products, **5**{1-126}, were obtained in low to excellent yields (20-96%) following purification by silica gel chromatography (Table 4).

**Table 3:** Pyrazines Substituted with Amino Acid Esters, **3** {*1*-*9*}.

compound	amino acid ester	yield (%)
**3{***1*}	**2**{*1*}	86
**3{***2*}	**2**{*2*}	91
**3{***3*}	**2**{*3*}	87
**3{***4*}	**2**{*4*}	93
**3{***5*}	**2**{*5*}	83
**3{***6*}	**2**{*6*}	87
**3{***7*}	**2**{*7*}	86
**3{***8*}	**2**{*8*}	87
**3{***9*}	**2**{*9*}	76

**Table 4:** Amino Acid Ester—Alkanamine Disubstituted I45DCs, **5**{*1*-*63*}.

cmpd	reactants		yield (%)		cmpd	reactants		yield (%)		cmpd	reactants		yield (%)
**5{***1*}	**3{***1*}	**4{***1*}	36		**5{***43*}	**3{***4*}	**4{***1*}	62		**5{***85*}	**3{***7*}	**4{***1*}	78
**5{***2*}	**3{***1*}	**4{***2*}	65		**5{***44*}	**3{***4*}	**4{***2*}	50		**5{***86*}	**3{***7*}	**4{***2*}	69
**5{***3*}	**3{***1*}	**4{***3*}	55		**5{***45*}	**3{***4*}	**4{***3*}	44		**5{***87*}	**3{***7*}	**4{***3*}	90
**5{***4*}	**3{***1*}	**4{***4*}	90		**5{***46*}	**3{***4*}	**4{***4*}	37		**5{***88*}	**3{***7*}	**4{***4*}	76
**5{***5*}	**3{***1*}	**4{***5*}	53		**5{***47*}	**3{***4*}	**4{***5*}	38		**5{***89*}	**3{***7*}	**4{***5*}	66
**5{***6*}	**3{***1*}	**4{***6*}	42		**5{***48*}	**3{***4*}	**4{***6*}	35		**5{***90*}	**3{***7*}	**4{***6*}	57
**5{***7*}	**3{***1*}	**4{***7*}	91		**5{***49*}	**3{***4*}	**4{***7*}	65		**5{***91*}	**3{***7*}	**4{***7*}	93
**5{***8*}	**3{***1*}	**4{***8*}	88		**5{***50*}	**3{***4*}	**4{***8*}	70		**5{***92*}	**3{***7*}	**4{***8*}	80
**5{***9*}	**3{***1*}	**4{***9*}	49		**5{***51*}	**3{***4*}	**4{***9*}	49		**5{***93*}	**3{***7*}	**4{***9*}	62
**5{***10*}	**3{***1*}	**4{***10*}	39		**5{***52*}	**3{***4*}	**4{***10*}	35		**5{***94*}	**3{***7*}	**4{***10*}	73
**5{***11*}	**3{***1*}	**4{***11*}	47		**5{***53*}	**3{***4*}	**4{***11*}	48		**5{***95*}	**3{***7*}	**4{***11*}	42
**5{***12*}	**3{***1*}	**4{***12*}	52		**5{***54*}	**3{***4*}	**4{***12*}	38		**5{***96*}	**3{***7*}	**4{***12*}	36
**5{***13*}	**3{***1*}	**4{***13*}	49		**5{***55*}	**3{***4*}	**4{***13*}	31		**5{***97*}	**3{***7*}	**4{***13*}	54
**5{***14*}	**3{***1*}	**4{***14*}	74		**5{***56*}	**3{***4*}	**4{***14*}	73		**5{***98*}	**3{***7*}	**4{***14*}	79
**5{***15*}	**3{***2*}	**4{***1*}	25		**5{***57*}	**3{***5*}	**4{***1*}	77		**5{***99*}	**3{***8*}	**4{***1*}	62
**5{***16*}	**3{***2*}	**4{***2*}	37		**5{***58*}	**3{***5*}	**4{***2*}	41		**5{***100*}	**3{***8*}	**4{***2*}	57
**5{***17*}	**3{***2*}	**4{***3*}	50		**5{***59*}	**3{***5*}	**4{***3*}	42		**5{***101*}	**3{***8*}	**4{***3*}	55
**5{***18*}	**3{***2*}	**4{***4*}	49		**5{***60*}	**3{***5*}	**4{***4*}	45		**5{***102*}	**3{***8*}	**4{***4*}	38
**5{***19*}	**3{***2*}	**4{***5*}	50		**5{***61*}	**3{***5*}	**4{***5*}	20		**5{***103*}	**3{***8*}	**4{***5*}	40
**5{***20*}	**3{***2*}	**4{***6*}	43		**5{***62*}	**3{***5*}	**4{***6*}	23		**5{***104*}	**3{***8*}	**4{***6*}	42
**5{***21*}	**3{***2*}	**4{***7*}	79		**5{***63*}	**3{***5*}	**4{***7*}	74		**5{***105*}	**3{***8*}	**4{***7*}	93
**5{***22*}	**3{***2*}	**4{***8*}	75		**5{***64*}	**3{***5*}	**4{***8*}	85		**5{***106*}	**3{***8*}	**4{***8*}	80
**5{***23*}	**3{***2*}	**4{***9*}	50		**5{***65*}	**3{***5*}	**4{***9*}	29		**5{***107*}	**3{***8*}	**4{***9*}	54
**5{***24*}	**3{***2*}	**4{***10*}	35		**5{***66*}	**3{***5*}	**4{***10*}	53		**5{***108*}	**3{***8*}	**4{***10*}	21
**5{***25*}	**3{***2*}	**4{***11*}	43		**5{***67*}	**3{***5*}	**4{***11*}	37		**5{***109*}	**3{***8*}	**4{***11*}	52
**5{***26*}	**3{***2*}	**4{***12*}	45		**5{***68*}	**3{***5*}	**4{***12*}	55		**5{***110*}	**3{***8*}	**4{***12*}	51
**5{***27*}	**3{***2*}	**4{***13*}	34		**5{***69*}	**3{***5*}	**4{***13*}	31		**5{***111*}	**3{***8*}	**4{***13*}	40
**5{***28*}	**3{***2*}	**4{***14*}	67		**5{***70*}	**3{***5*}	**4{***14*}	67		**5{***112*}	**3{***8*}	**4{***14*}	82
**5{***29*}	**3{***3*}	**4{***1*}	77		**5{***71*}	**3{***6*}	**4{***1*}	71		**5{***113*}	**3{***9*}	**4{***1*}	50
**5{***30*}	**3{***3*}	**4{***2*}	58		**5{***72*}	**3{***6*}	**4{***2*}	58		**5{***114*}	**3{***9*}	**4{***2*}	55
**5{***31*}	**3{***3*}	**4{***3*}	53		**5{***73*}	**3{***6*}	**4{***3*}	46		**5{***115*}	**3{***9*}	**4{***3*}	56
**5{***32*}	**3{***3*}	**4{***4*}	52		**5{***74*}	**3{***6*}	**4{***4*}	46		**5{***116*}	**3{***9*}	**4{***4*}	48
**5{***33*}	**3{***3*}	**4{***5*}	45		**5{***75*}	**3{***6*}	**4{***5*}	39		**5{***117*}	**3{***9*}	**4{***5*}	42
**5{***34*}	**3{***3*}	**4{***6*}	41		**5{***76*}	**3{***6*}	**4{***6*}	44		**5{***118*}	**3{***9*}	**4{***6*}	32
**5{***35*}	**3{***3*}	**4{***7*}	93		**5{***77*}	**3{***6*}	**4{***7*}	83		**5{***119*}	**3{***9*}	**4{***7*}	90
**5{***36*}	**3{***3*}	**4{***8*}	92		**5{***78*}	**3{***6*}	**4{***8*}	89		**5{***120*}	**3{***9*}	**4{***8*}	93
**5{***37*}	**3{***3*}	**4{***9*}	39		**5{***79*}	**3{***6*}	**4{***9*}	54		**5{***121*}	**3{***9*}	**4{***9*}	43
**5{***38*}	**3{***3*}	**4{***10*}	46		**5{***80*}	**3{***6*}	**4{***10*}	51		**5{***122*}	**3{***9*}	**4{***10*}	45
**5{***39*}	**3{***3*}	**4{***11*}	38		**5{***81*}	**3{***6*}	**4{***11*}	50		**5{***123*}	**3{***9*}	**4{***11*}	48
**5{***40*}	**3{***3*}	**4{***12*}	50		**5{***82*}	**3{***6*}	**4{***12*}	56		**5{***124*}	**3{***9*}	**4{***12*}	44
**5{***41*}	**3{***3*}	**4{***13*}	37		**5{***83*}	**3{***6*}	**4{***13*}	42		**5{***125*}	**3{***9*}	**4{***13*}	41
**5{***42*}	**3{***3*}	**4{***14*}	96		**5{***84*}	**3{***6*}	**4{***14*}	93		**5{***126*}	**3{***9*}	**4{***14*}	91

There are no obvious patterns evident in the purified yields when analyzed by either amino acid ester or alkanamine (Table 5). Instead, the yield range, average, and median are largely alike in each of these comparisons except for three alkanamines, **4**{7,8,14}, which gave an 80% or higher average yield. 

The major impurity present in the crude reactions is an imidazole-4,5-dicarboxylic acid substituted with only the amino acid ester (an imidazole carboxamide−carboxylic acid substituent combination). The strongest evidence for this side product is from LC-MS analysis, where the crude reactions show both the desired product and a faster eluting compound with an observed m/z that is expected for this structure. This impurity is hypothesized to result from an incomplete reaction between alkanamines **4** and the intermediate pyrazines **3** or competitive hydrolysis of the pyrazine due to adventitious water or both. We see no evidence of this carboxylic acid impurity in the ^1^H-NMR spectra of the pyrazines, **3**, lending us to think that the impurity forms during the purification process of the final compounds or in the reactions to yield these compounds, and not during the washing and isolation steps in the pyrazine synthesis. Indeed, there are many final compounds from the pyrazines that give excellent yields of the final product, supporting the purity of these intermediates. The ^1^H-NMR spectrum of purified library members is comparable to the spectrum for the corresponding crude reaction and further supports the presence of only the hydrolysis product of the pyrazine, and suggests it forms during the reactions to the final products. We have not quantified the level of this impurity in these crude reactions, although the ratio of peaks observed at 214 nm suggests that it is substantial in most reactions. 

**Table 5:** Yields of purified library members based on amino acid esters, **2**{1-9}, and alkanamines, **4**{*1*-*14*}.

member	low	high	average	median
**2**{*1*}	36	91	59	53
**2**{*2*}	25	79	49	47
**2**{*3*}	37	96	58	51
**2**{*4*}	31	73	48	46
**2**{*5*}	20	85	49	44
**2**{*6*}	39	93	59	53
**2**{*7*}	36	93	68	71
**2**{*8*}	21	93	55	53
**2**{*9*}	32	93	56	48
**4{***1*}	25	78	60	62
**4{***2*}	37	69	54	57
**4{***3*}	42	90	55	53
**4{***4*}	37	90	53	48
**4{***5*}	20	66	44	42
**4{***6*}	23	57	40	42
**4{***7*}	65	93	85	90
**4{***8*}	70	93	84	85
**4{***9*}	29	62	48	49
**4{***10*}	21	73	44	45
**4{***11*}	37	52	45	47
**4{***12*}	36	56	47	50
**4{***13*}	31	54	40	40
**4{***14*}	67	96	80	79

All of the library members have been submitted to and accepted into the MLSMR for screening by the MLSCN. At the time of this writing, compounds in this library have shown bioactivity against calpain II and in cell based assays that measure an arbovirus challenge, Leishmania major promastigote inhibition, as well as levels of Amyloid Precursor Protein (APP) translation. The current biological data is best accessed through the PubChem database [32] by searching the SID identification numbers within “PubChem Substance.” Table 6 provides the compound number in this report with the corresponding PubChem SID numbers. PubChemSR is software that operates in a Windows environment and may be useful in helping researchers mine the data found in PubChem [33].

**Table 6:** Cross reference guide for library members with the PubChem database (SID identification number).

cmpd	PubChem SID^‡^		cmpd	PubChem SID^‡^	
**5{***1*}	49714445		**5{***64*}	24833560	
**5{***2*}	49733433		**5{***65*}	24833894	
**5{***3*}	49714444		**5{***66*}	24833104	
**5{***4*}	49714441		^§,&^**5{***67*}	24833105	
**5{***5*}	49731971		**5{***68*}	26732529	
**5{***6*}	49714443		**5{***69*}	26732544	
**5{***7*}	49731972		**5{***70*}	26732517	
**5{***8*}	49714442		**5{***71*}	24833022	
**5{***9*}	49731973		**5{***72*}	24833022	
**5{***10*}	49733435		**5{***73*}	24833129	
**5{***11*}	49714448		**5{***74*}	24833126	
**5{***12*}	49714446		^†^**5{***75*}	26724165	
**5{***13*}	49714447		^¶,$^**5{***76*}	24833555	
**5{***14*}	49734318		**5{***77*}	49713839	
**5{***15*}	49731969		**5{***78*}	24833096	
**5{***16*}	50096422		**5{***79*}	24833112	
**5{***17*}	49714435		**5{***80*}	24833110	
**5{***18*}	49714431		**5{***81*}	24833889	
**5{***19*}	49714432		**5{***82*}	26732526	
**5{***20*}	49714434		**5{***83*}	26732522	
**5{***21*}	49714436		**5{***84*}	26732523	
**5{***22*}	49714433		**5{***85*}	24833099	
**5{***23*}	49714439		**5{***86*}	50091477	
**5{***24*}	49714438		**5{***87*}	24833779	
**5{***25*}	49714440		^#^**5{***88*}	24833101	
**5{***26*}	49714437		**5{***89*}	24833100	
**5{***27*}	49731970		**5{***90*}	24833102	
**5{***28*}	49733434		**5{***91*}	49714426	
**5{***29*}	24833676		**5{***92*}	50096423	
**5{***30*}	24833109		**5{***93*}	49733432	
**5{***31*}	24833559		**5{***94*}	49714430	
**5{***32*}	24833114		**5{***95*}	49734317	
**5{***33*}	24833781		**5{***96*}	49714427	
**5{***34*}	24833951		**5{***97*}	49714429	
**5{***35*}	24833140		**5{***98*}	49714428	
**5{***36*}	24833558		**5{***99*}	24833131	
**5{***37*}	50091475		^^,*^5{*100*}	24833885	
**5{***38*}	24834116		^*^5{*101*}	24833127	
**5{***39*}	50091476		**5{***102*}	24833128	
**5{***40*}	26732545		**5{***103*}	24833130	
**5{***41*}	26732528		**5{***104*}	24833095	
**5{***42*}	26732527		**5{***105*}	49714449	
**5{***43*}	26724151		**5{***106*}	24834139	
**5{***44*}	24834138		**5{***107*}	24833125	
**5{***45*}	49713841		**5{***108*}	24833678	
**5{***46*}	26724154		**5{***109*}	49734320	
**5{***47*}	50096424		**5{***110*}	26732513	
**5{***48*}	24833780		**5{***111*}	50096425	
**5{***49*}	50091474		^†^**5{***112*}	26732521	
**5{***50*}	26724153		**5{***113*}	24833107	
**5{***51*}	24833694		^†^**5{***114*}	24833556	
**5{***52*}	24833113		^#^**5{***115*}	24833677	
**5{***53*}	49731978		^†^**5{***116*}	24833106	
**5{***54*}	26732519		**5{***117*}	26732518	
**5{***55*}	26732548		**5{***118*}	26724152	
**5{***56*}	49733437		**5{***119*}	49713842	
**5{***57*}	24833796		**5{***120*}	24833097	
^†^**5{***58*}	24833891		^%^**5{***121*}	24834137	
^†^**5{***59*}	24833103		**5{***122*}	24833692	
**5{***60*}	24833557		**5{***123*}	24833098	
**5{***61*}	24833561		**5{***124*}	26732532	
**5{***62*}	24833108		**5{***125*}	26732530	
**5{***63*}	49713840		**5{***126*}	26732543	

^‡^The PubChem information for each library member, along with the current bioassay results, can be found by inserting the appropriate SID number into the spaces indicated by ######### in the link: http://pubchem.ncbi.nlm.nih.gov/summary/summary.cgi?sid=########&loc=ec_rcs. Since each submission of a compound results in a unique PubChem SID number, it is useful to search for other samples of the same compound under the link “Related Structures.” Biological data may be listed for one sample of a compound and not for other samples of the same compound; ^†^These compounds have shown activity against calpain II; ^§^These compounds have shown activity against an arbovirus challenge; ^¶^These compounds have shown Leishmania major promastigote inhibition; ^&^These compounds have shown inhibition of Amyloid Precursor Protein (APP) translation; ^#^These compounds have been “cherry picked” for further biological assessment and the results are pending at the time of this writing; ^%^This compound is active as a small molecule regulator of Bcl-2 family protein interactions; ^$^This compound is active as a potentiator or agonist of neuropeptide Y receptor Y2; ^^^This compound is active as a potentiator or agonist of neuropeptide Y receptor Y1; ^*^This compound actively inhibits the RAM network.

## Experimental

### General

All apparatus was oven-dried and cooled in a desiccator. All reagents were purchased from commercial suppliers and used without further purification. Reagent grade CH_2_Cl_2 _was distilled from CaH_2_ before use. Thin layer chromatography (TLC) was performed on 250 µm glass-backed silica gel plates and visualized using UV. Column chromatography was performed on silica gel (Merck, grade 9385, 230-400 mesh, 60 Å). The columns were prepared in plastic 20 or 30 mL syringe bodies and filled to approximately 2/3 of their capacity with silica gel. This column volume was sufficient for purification of most reaction products because the most substantial impurity is the imidazole-4,5-dicarboxylic acid substituted with a carboxamide and carboxylic acid that absorbs strongly to silica gel. In select cases, a second column was necessary in order to achieve analytically pure material. Melting points were determined in glass capillary tubes and are uncorrected. The names of the final compounds are provided in Table 7 at the end of the experimental section.

### LC-MS Analysis

Characterization of the purity and identity of the library members was carried out by liquid chromatography-mass spectrometry (LC-MS) using a Varian 500-MS LC Ion Trap mass spectrometer. Solutions of the compounds were prepared at an approximate concentration of 1 mg/mL by first adding <10% by volume of CH_2_Cl_2_ to dissolve the sample and then diluting to the final volume with HPLC grade methanol. Five microliters of the sample was injected onto a Polaris 5 μm C18-A (50´2.0 mm) HPLC column and eluted with a gradient of CH_3_CN/H_2_O containing 0.1% CH_3_CO_2_H at a 0.2 mL/min flow rate. Compounds were detected at 214 nm. The gradient was as follows: 0 min., 4:6 CH_3_CN/H_2_O → 1 min., 4:6 CH_3_CN/H_2_O → 6 min., 9:1 CH_3_CN/H_2_O → 8 min., 9:1 CH_3_CN/H_2_O → 9 min., 4:6 CH_3_CN/H_2_O → 10 min., 4:6 CH_3_CN/H_2_O. The mass spectrum was recorded for the entire elution time by using ESI detection from 50-800 (m/z) with the following parameters: capillary voltage at 60.0, R_f_ loading at 100%, drying gas at 250 °C, spray chamber at 50 °C, nebulizer gas at 50.0 psi, drying gas at 25 psi, and damping gas at 0.8 mL/min.

### NMR Spectroscopy

^1^H-NMR and ^13^C-NMR spectra were recorded at 10 mM at 400 MHz and 100.5 MHz, respectively, in CDCl_3_ with CHCl_3_ as the internal reference for ^1^H (d 7.24) and CDCl_3_ as the internal reference for ^13^C (d 77.00) or DMSO-*d*_6_ with DMSO as the internal reference for ^1^H (d 2.49) and DMSO-*d*_6 _as the internal reference for ^13^C (d 39.50). Coupling constants are expressed in Hertz (Hz). Data are reported in this sequence: 1) chemical shifts (ppm); 2) spin multiplicities given as, s (singlet), bs (broad singlet), d (doublet), t (triplet), q (quartet), quintet, sextet and m (multiplet); 3) coupling constant *J* (Hz); and 4) integration.

### Synthesis

*5,10-Dioxo-5H,10H-diimidazo{1,5-a:1´,5´-d}pyrazine-1,6-dicarbonyl Glycyl Esters,* **3**{1-2}. To a dry round-bottom flask under argon was added dry CH_2_Cl_2 _(25 mL). To this stirred solvent at -78 °C were added, in order, the pyrazine diacid chloride **1** (1.00 g, 3.19 mmol), the glycine ester hydrochloride salt **2**{1-2} (6.38 mmol) and *N,N*-diethylaniline (1.91 g, 2.05 mL, 12.8 mmol). The resulting solution was held at -78 °C for 30 minutes before stirring 1 hour at room temperature. The solid precipitate was collected by vacuum filtration and washed with EtOAc to yield ****3{**1-2}**. The final products were characterized by melting point as well as ^1^H- and ^13^C-NMR spectroscopy.

**3**{1}: The product was a white solid obtained in 86% yield: mp > 250 °C; ^1^H-NMR (DMSO-*d*_6_) δ 8.97-8.94 (m, 4 H), 3.97 (d, *J* = 6.4 Hz, 4 H), 1.43 (s, 18 H); ^13^C-NMR (DMSO-*d*_6_) δ 168.4, 159.2,149.0, 143.9, 138.3, 120.6, 80.9, 41.7, 27.7.

**3**{2}: The product was a white solid obtained in 91% yield: mp 222-223 °C (dec); ^1^H-NMR (DMSO-*d*_6_) δ 9.07 (t, *J* = 6.0 Hz, 2 H), 8.97 (d, *J* = 6.0 Hz, 2 H), 7.39-7.31 (m, 10 H), 5.18 (s, 4H), 4.15 (d, *J* = 6.0 Hz, 4 H); ^13^C-NMR (DMSO-*d*_6_) δ 169.3, 159.4, 148.9, 143.7, 138.3, 135.8, 128.4, 128.0, 127.9, 120.7, 66.0, 41.1.

*5,10-dioxo-5H,10H-diimidazo{1,5-a:1´,5´-d}pyrazine-1,6-dicarbonyl Amino Acid Esters***, 3{3-9}. **To a dry round-bottom flask under argon was added dry CH_2_Cl_2 _(25 mL). To this stirred solvent at -78 °C were added, in order, the pyrazine diacid chloride **1** (1.00 g, 3.19 mmol), the amino acid ester hydrochloride or tosylate salt **2**{3-9} (6.38 mmol) and *N,N*-diethylaniline (1.91 g, 2.05 mL, 12.8 mmol). The resulting solution was held at -78 °C for 30 minutes before stirring 1 hour at room temperature. The reaction was washed against water (3×) and the organic fraction was dried over MgSO_4_, filtered and concentrated. The residue was suspended in boiling EtOAc, stirred for 15 minutes and then cooled at 0 °C. The solid product **3**{3-9} was collected by vacuum filtration and washed with EtOAc. The final product was characterized by melting point as well as ^1^H- and ^13^C-NMR spectroscopy.

**3**{3}: The product was a light yellow solid obtained in 87% yield: mp 225 °C (dec); ^1^H-NMR (DMSO-*d*_6_) δ 8.68 (d, *J* = 7.6 Hz, 2 H), 8.64 (s, 2 H), 4.71 (quintet, *J* = 7.2 Hz, 2 H), 1.51 (d, *J* = 6.8 Hz, 6 H), 1.48 (s, 18 H); ^13^C-NMR (DMSO-*d*_6_) δ 171.6, 157.2, 149.2, 146.9, 138.3, 118.9, 82.3, 49.2, 27.9, 18.4.

**3**{4}: The product was a yellow solid obtained in 93% yield: mp 141-143 °C; ^1^H-NMR (DMSO-*d*_6_) δ 8.68 (d, *J* = 7.2 Hz, 2 H), 8.62 (s, 2 H), 7.35-7.31 (m, 10 H), 5.21 (dd, *J* = 12 Hz, *J* = 12 Hz, 4 H), 4.87 (quintet, *J* = 7.2 Hz, 2 H), 1.56 (d, *J* = 7.2 Hz, 6 H); ^13^C-NMR (DMSO-*d*_6_) δ 172.2, 157.6, 148.9, 146.7, 138.4, 135.2, 128.6, 128.5, 128.2, 118.9, 67.4, 48.8, 18.3.

**3**{5}: The product was a white solid obtained in 83% yield: mp 222-223 °C (dec); ^1^H-NMR (DMSO-*d*_6_) δ 8.63 (s, 2 H), 8.51 (d, *J* = 8 Hz, 2 H), 4.79-7.74 (m, 2 H), 1.77-1.65 (m, 3.22 6 H), 1.47 (s, 18 H), 0.97 (d, *J* = 6.4 Hz, 12 H); ^13^C-NMR (DMSO-*d*_6_) δ 171.6, 157.4, 149.0, 147.1, 138.4, 118.8, 82.3, 52.0, 41.9, 28.0, 25.1, 22.8, 22.2.

**3**{6}: The product was a white solid obtained in 87% yield: mp 176-178 °C (Lit. 140-141 °C, Ref. [16]); ^1^H-NMR (DMSO-*d*_6_) δ 8.62 (s, 2 H), 8.52 (d, *J* = 8.4 Hz, 2 H), 7.35-7.29 (m, 10 H), 5.18 (s, 4 H), 4.94-4.88 (m, 2 H), 1.82-1.69 (m, 6 H), 0.95 (d, *J* = 2.4 Hz, 6 H); 0.94 (d, *J* = 2.0 Hz, 6 H); ^13^C- NMR (DMSO-*d*_6_) δ 172.1, 157.6, 149.0, 146.6, 138.4, 135.3, 128.6, 128.4, 128.2, 119.0, 67.2, 51.5, 41.4, 25.0, 22.8, 22.2.

**3**{7}: The product was a white solid obtained in 87% yield: mp 201-203 °C (dec) (Lit. 179-180 °C (dec) [10]); ^1^H-NMR (DMSO-*d*_6_) δ 8.59 (d, *J* = 4 Hz, 2 H), 8.57 (s, 2 H), 7.27-7.19 (m, 10 H), 5.00 (dd, *J* = 6.4 Hz, *J* = 6.4 Hz, 2 H), 3.22 (d, *J* = 6.4 Hz, 4 H), 1.41 (s, 18 H); ^13^C-NMR (DMSO-*d*_6_) δ 170.0, 157.3, 148.9, 146.7, 138.3, 136.0, 129.4, 128.3, 126.9, 118.8, 82.6, 54.3, 38.1, 27.9.

**3**{8}: The product was a white solid obtained in 86% yield: mp 175-177 °C; ^1^H-NMR (DMSO-*d*_6_) δ 8.56 (d, *J* = 8 Hz, 2 H), 8.53 (s, 2 H), 7.34-7.04 (m, 20 H), 5.15-5.10 (m, 6 H), 3.27 (dd, *J* = 6.0 Hz, *J* = 14, 2 H), 3.20 (dd, *J* = 6.0 Hz, *J* = 13.6, 2 H); ^13^C-NMR (DMSO-*d*_6_) δ 170.8, 157.4, 148.7, 146.4, 138.3, 135.5, 135.0, 129.3, 128.5, 127.1, 118.9, 67.4, 53.9, 37.8.

**3**{9}: The product was a white solid obtained in 76% yield: mp 150-152 °C; ^1^H-NMR (DMSO-*d*_6_) δ 8.71-8.68 (m, 4 H), 4.78-4.73 (m, 2 H), 4.49 (broad, 2 H), 3.08 (bs, 4 H), 2.01-1.94 (m, 2 H), 1.85-1.78 (m, 2 H), 1.53-1.32 (m, 44 H); ^13^C-NMR (DMSO-*d*_6_) δ 170.8, 157.5, 155.9, 149.2, 146.9, 138.6, 118.8, 82.6, 79.2, 53.2, 40.4, 32.1, 29.7, 28.4, 28.02, 28.0, 22.3.

*Dissymmetric Imidazole-4,5-dicarboxamides* **5**{2-6}, **5**{8-13}, **5**{16-20}, **5**{22-27}, **5**{30-34}, **5**{36-41}, **5**{44-48}, **5**{50-55}, **5**{58-62}, **5**{64-69}, **5**{72-76}, **5**{78-83}, **5**{86-90}, **5**{92-97}, **5**{100-104}, **5**{106-111}, **5**{114-118}**, 5**{120-125}, *Disubstituted with Amino Acid Esters* **2**{1-9} *and Alkanamines* **4** {2-6}, **4**{8-13}.****Screw capped culture tubes were dried overnight in an oven. Each of the nine amino acid ester substituted pyrazine **3**{1-9} (0.100 mmol) was transferred to 11 culture tubes followed by dry CH_2_Cl_2_ (3 mL). Solutions of the eleven different amines **4**{2-6} and **4**{8-13} at 4 M were prepared in CH_2_Cl_2_ and 50 mL (0.200 mmol) of each amine was transferred robotically to the corresponding tubes for a total of 99 tubes (9×11). The tubes were purged with argon and capped. The tubes were placed on an orbital shaker for 2 days at room temperature and the solutions gently mixed by the orbital action. The progress of the reactions was followed with TLC by using a mixture of EtOAc/hexane (1:1) as the eluant. After 2 days the solvent was evaporated and the residues were purified by column chromatography on silica gel with EtOAc/hexane (1:1) initially and then just EtOAc as the eluant. The desired fractions were combined and concentrated to give the final products. The purity and identity of the final products was tested by LC-MS and ^1^H-NMR.

*Dissymmetric Imidazole-4,5-dicarboxamides* **5**{1}, **5**{15}, **5**{29}, **5**{43}, **5**{57}, **5**{71}, **5**{85}, **5**{99}, **5**{113} *Disubstituted with Amino Acid Esters* **2**{1-9} * and Methylamine Hydrochloride* **4**{1}. Screw capped culture tubes were dried overnight in an oven. Each tube was filled with one amino acid ester substituted pyrazine **3**{1-9} (0.100 mmol). Methylamine hydrochloride **4**{1} (27.0 mg, 0.400 mmol) was then weighed and transferred into each tube, followed by dry CH_2_Cl_2_ (3 mL) for a total of 9 tubes (9×1). A 4.0 M solution of diisopropylethylamine (DIEA) in CH_2_Cl_2_ was prepared and 100 mL (0.400 mmol) of this solution was added to each tube. The tubes were purged with argon and sealed. The tubes were placed on an orbital shaker for 2 days at room temperature and the solutions gently mixed by the orbital action. The progress of the reactions was followed with TLC by using a mixture of EtOAc/hexane (1:1) as the eluant. After 2 days the solvent was evaporated and the residues were purified by column chromatography on silica gel with EtOAc/hexane (1:1) initially and then just EtOAc as the eluant. The desired fractions were combined and concentrated to give the final products. The purity and identity of the final products was tested by LC-MS and ^1^H-NMR.

*Dissymmetric Imidazole-4,5-dicarboxamides* **5**{7}, **5**{21}, **5**{35}, **5**{49}, **5**{63}, **5**{77}, **5**{91}, **5**{105}, **5**{119} *Disubstituted with Amino Acid Esters* **2**{1-9} and *1-(N-Boc-aminomethyl)-4-(aminomethyl)benzene* **4**{7}. Screw capped culture tubes were dried overnight in an oven. Each tube was filled with one amino acid ester substituted pyrazine **3**{1-9} (0.100 mmol). 1-(*N*-Boc-aminomethyl)-4-(aminomethyl)benzene **4**{7} (47.3 mg, 0.200 mmol) was then weighed and transferred into each tube, followed by dry CH_2_Cl_2_ (3 mL) for a total of 9 tubes (9×1). The tubes were purged with argon and sealed. The tubes were placed on an orbital shaker for 2 days at room temperature and the solutions gently mixed by the orbital action. The progress of the reactions was followed with TLC by using a mixture of EtOAc/hexane (1:1) as the eluant. After 2 days the solvent was evaporated and the residues were purified by column chromatography on silica gel with EtOAc/hexane (1:1) initially and then just EtOAc as the eluant. The desired fractions were combined and concentrated to give the final products. The purity and identity of the final products was tested by LC-MS and ^1^H-NMR.

*Dissymmetric Imidazole-4,5-dicarboxamides* **5**{14}, **5**{28}, **5**{42}, **5**{56}, **5**{70}, **5**{84}, **5**{98}, **5**{112}, **5**{126} *Disubstituted with Amino Acid Esters* **2**{1-9}* and 1-Phenylpiperazine Hydrochloride* **4**{14}. Screw capped culture tubes were dried overnight in an oven. Each tube was filled with one amino acid ester substituted pyrazine **3**{1-9} (0.100 mmol). 1-Phenylpiperazine hydrochloride **4**{14} (39.7 mg, 0.200 mmol) was then weighed and transferred into each tube, followed by dry CH_2_Cl_2_ (3 mL) for a total of 9 tubes (9×1). A 4.0 M solution of diisopropylethylamine (DIEA) in CH_2_Cl_2 _was prepared and 50 mL (0.200 mmol) of this solution was added to each tube. The tubes were purged with argon and sealed. The tubes were placed on an orbital shaker for 2 days at room temperature and the solutions gently mixed by the orbital action. The progress of the reactions was followed with TLC by using a mixture of EtOAc/hexane (1:1) as the eluant. After 2 days the solvent was evaporated and the residues were purified by column chromatography on silica gel with EtOAc/hexane (1:1) initially and then just EtOAc as the eluant. The desired fractions were combined and concentrated to give the final products. The purity and identity of the final products was tested by LC-MS and ^1^H-NMR.

**Table 7:** Final Product Numbers and Nomenclature.

cmpd	Name
**5{***1*}	4-[(methylamino)carbonyl]-5-[(*tert*-butoxyglycyl)carbonyl]-1*H*-imidazole
**5{***2*}	4-[5-({[(tert-butoxy)carbonyl]amino}pentylamino)carbonyl]-5-[(*tert*-butoxyglycyl)carbonyl]-1*H*-imidazole
**5{***3*}	4-[(benzylamino)carbonyl]-5-[(*tert*-butoxyglycyl)carbonyl]-1*H*-imidazole
**5{***4*}	4-[(*R*-α-methylbenzylamino)carbonyl]-5-[(*tert*-butoxyglycyl)carbonyl]-1*H*-imidazole
**5{***5*}	4-[(*S*-α-methylbenzylamino)carbonyl]-5-[(*tert*-butoxyglycyl)carbonyl]-1*H*-imidazole
**5{***6*}	4-[4-({[(tert-butoxy)carbonyl]piperidinyl}amino)carbonyl]-5-[(*tert*-butoxyglycyl)carbonyl]-1*H*-imidazole
**5{***7*}	4-{[(4-{[(*tert*-butoxy)carbonyl]aminomethyl}phenyl)methylamino]carbonyl}-5-[(*tert*-butoxyglycyl)carbonyl]-1*H*-imidazole
**5{***8*}	4-[(dibenzylamino)carbonyl]-5-[(*tert*-butoxyglycyl)carbonyl]-1*H*-imidazole
**5{***9*}	4-[(*N*-methylbenzylamino)carbonyl]-5-[(*tert*-butoxyglycyl)carbonyl]-1*H*-imidazole
**5{***10*}	4-[(*N,N*-diethylamino)carbonyl]-5-[(*tert*-butoxyglycyl)carbonyl]-1*H*-imidazole
**5{***11*}	4-({4-[(*tert*-butoxy)carbonyl]piperazinyl}carbonyl)-5-[(*tert*-butoxyglycyl)carbonyl]-1*H*-imidazole
**5{***12*}	4-[(1,2,3,4-tetrahydroisoquinolinyl)carbonyl]-5-[(*tert*-butoxyglycyl)carbonyl]-1*H*-imidazole
**5{***13*}	4-[(4-methylpiperidinyl)carbonyl]-5-[(*tert*-butoxyglycyl)carbonyl]-1*H*-imidazole
**5{***14*}	4-({4-[(*tert*-butoxy)carbonyl]piperazinyl}carbonyl)-5-[(*tert*-butoxyglycyl)carbonyl]-1*H*-imidazole
**5{***15*}	4-[(methylamino)carbonyl]-5-[(benzyloxyglycyl)carbonyl]-1 *H*-imidazole
**5{***16*}	4-[5-({[(tert-butoxy)carbonyl]amino}pentylamino)carbonyl]-5-[(benzyloxyglycyl)carbonyl]-1 *H*-imidazole
**5{***17*}	4-[(benzylamino)carbonyl]-5-[(benzyloxyglycyl)carbonyl]-1 *H*-imidazole
**5{***18*}	4-[(*R*-α-methylbenzylamino)carbonyl]-5-[(benzyloxyglycyl)carbonyl]-1*H*-imidazole
**5{***19*}	4-[(*S*-α-methylbenzylamino)carbonyl]-5-[(benzyloxyglycyl)carbonyl]-1*H*-imidazole
**5{***20*}	4-[4-({[(tert-butoxy)carbonyl]piperidinyl}amino)carbonyl]-5-[(benzyloxyglycyl)carbonyl]-1 *H*-imidazole
**5{***21*}	4-{[(4-{[(*tert*-butoxy)carbonyl]aminomethyl}phenyl)methylamino]carbonyl}-5-[(benzyloxyglycyl)carbonyl]-1*H*-imidazole
**5{***22*}	4-[(dibenzylamino)carbonyl]-5-[(benzyloxyglycyl)carbonyl]-1 *H*-imidazole
**5{***23*}	4-[(*N*-methylbenzylamino)carbonyl]-5-[(benzyloxyglycyl)carbonyl]-1*H*-imidazole
**5{***24*}	4-[(*N,N*-diethylamino)carbonyl]-5-[(benzyloxyglycyl)carbonyl]-1*H*-imidazole
**5{***25*}	4-({4-[(*tert*-butoxy)carbonyl]piperazinyl}carbonyl)-5-[(benzyloxyglycyl)carbonyl]-1*H*-imidazole
**5{***26*}	4-[(1,2,3,4-tetrahydroisoquinolinyl)carbonyl]-5-[(benzyloxyglycyl)carbonyl]-1 *H*-imidazole
**5{***27*}	4-[(4-methylpiperidinyl)carbonyl]-5-[(benzyloxyglycyl)carbonyl]-1 *H*-imidazole
**5{***28*}	4-({4-[(*tert*-butoxy)carbonyl]piperazinyl}carbonyl)-5-[(benzyloxyglycyl)carbonyl]-1*H*-imidazole
**5{***29*}	4-[(methylamino)carbonyl]-5-[(*tert*-butoxy-*S*-alanyl)carbonyl]-1*H*-imidazole
**5{***30*}	4-[5-({[(tert-butoxy)carbonyl]amino}pentylamino)carbonyl]-5-[(*tert*-butoxy-*S*-alanyl)carbonyl]-1*H*-imidazole
**5{***31*}	4-[(benzylamino)carbonyl]-5-[(*tert*-butoxy-*S*-alanyl)carbonyl]-1*H*-imidazole
**5{***32*}	4-[(*R*-α-methylbenzylamino)carbonyl]-5-[(*tert*-butoxy-*S*-alanyl)carbonyl]-1*H*-imidazole
**5{***33*}	4-[(*S*-α-methylbenzylamino)carbonyl]-5-[(*tert*-butoxy-*S*-alanyl)carbonyl]-1*H*-imidazole
**5{***34*}	4-[4-({[(tert-butoxy)carbonyl]piperidinyl}amino)carbonyl]-5-[(*tert*-butoxy-*S*-alanyl)carbonyl]-1*H*-imidazole
**5{***35*}	4-{[(4-{[(*tert*-butoxy)carbonyl]aminomethyl}phenyl)methylamino]carbonyl}-5-[(*tert*-butoxy-*S*-alanyl)carbonyl]-1*H*-imidazole
**5{***36*}	4-[(dibenzylamino)carbonyl]-5-[(*tert*-butoxy-*S*-alanyl)carbonyl]-1*H*-imidazole
**5{***37*}	4-[(*N*-methylbenzylamino)carbonyl]-5-[(*tert*-butoxy-*S*-alanyl)carbonyl]-1*H*-imidazole
**5{***38*}	4-[(*N,N*-diethylamino)carbonyl]-5-[(*tert*-butoxy-*S*-alanyl)carbonyl]-1*H*-imidazole
**5{***39*}	4-({4-[(*tert*-butoxy)carbonyl]piperazinyl}carbonyl)-5-[(*tert*-butoxy-*S*-alanyl)carbonyl]-1*H*-imidazole
**5{***40*}	4-[(1,2,3,4-tetrahydroisoquinolinyl)carbonyl]-5-[(*tert*-butoxy-*S*-alanyl)carbonyl]-1*H*-imidazole
**5{***41*}	4-[(4-methylpiperidinyl)carbonyl]-5-[(*tert*-butoxy-*S*-alanyl)carbonyl]-1*H*-imidazole
**5{***42*}	4-({4-[(*tert*-butoxy)carbonyl]piperazinyl}carbonyl)-5-[(*tert*-butoxy-*S*-alanyl)carbonyl]-1*H*-imidazole
**5{***43*}	4-[(methylamino)carbonyl]-5-[(benzyloxy- *S*-alanyl)carbonyl]-1*H*-imidazole
**5{***44*}	4-[5-({[(tert-butoxy)carbonyl]amino}pentylamino)carbonyl]-5-[(benzyloxy- *S*-alanyl)carbonyl]-1*H*-imidazole
**5{***45*}	4-[(benzylamino)carbonyl]-5-[(benzyloxy- *S*-alanyl)carbonyl]-1*H*-imidazole
**5{***46*}	4-[(*R*-α-methylbenzylamino)carbonyl]-5-[(benzyloxy-*S*-alanyl)carbonyl]-1*H*-imidazole
**5{***47*}	4-[(*S*-α-methylbenzylamino)carbonyl]-5-[(benzyloxy-*S*-alanyl)carbonyl]-1*H*-imidazole
**5{***48*}	4-[4-({[(tert-butoxy)carbonyl]piperidinyl}amino)carbonyl]-5-[(benzyloxy- *S*-alanyl)carbonyl]-1*H*-imidazole
**5{***49*}	4-{[(4-{[(*tert*-butoxy)carbonyl]aminomethyl}phenyl)methylamino]carbonyl}-5-[(benzyloxy-*S*-alanyl)carbonyl]-1*H*-imidazole
**5{***50*}	4-[(dibenzylamino)carbonyl]-5-[(benzyloxy-S-alanyl)carbonyl]-1 *H*-imidazole
**5{***51*}	4-[(*N*-methylbenzylamino)carbonyl]-5-[(benzyloxy-*S*-alanyl)carbonyl]-1*H*-imidazole
**5{***52*}	4-[(*N,N*-diethylamino)carbonyl]-5-[(benzyloxy-*S*-alanyl)carbonyl]-1*H*-imidazole
**5{***53*}	4-({4-[(*tert*-butoxy)carbonyl]piperazinyl}carbonyl)-5-[(benzyloxy-*S*-alanyl)carbonyl]-1*H*-imidazole
**5{***54*}	4-[(1,2,3,4-tetrahydroisoquinolinyl)carbonyl]-5-[(benzyloxy- *S*-alanyl)carbonyl]-1*H*-imidazole
**5{***55*}	4-[(4-methylpiperidinyl)carbonyl]-5-[(benzyloxy- *S*-alanyl)carbonyl]-1*H*-imidazole
**5{***56*}	4-({4-[(*tert*-butoxy)carbonyl]piperazinyl}carbonyl)-5-[(benzyloxy-*S*-alanyl)carbonyl]-1*H*-imidazole
**5{***57*}	4-[(methylamino)carbonyl]-5-[(*tert*-butoxy-*S*-leucyl)carbonyl]-1*H*-imidazole
**5{***58*}	4-[5-({[(tert-butoxy)carbonyl]amino}pentylamino)carbonyl]-5-[(*tert*-butoxy-*S*-leucyl)carbonyl]-1*H*-imidazole
**5{***59*}	4-[(benzylamino)carbonyl]-5-[(*tert*-butoxy-*S*-leucyl)carbonyl]-1*H*-imidazole
**5{***60*}	4-[(*R*-α-methylbenzylamino)carbonyl]-5-[(*tert*-butoxy-*S*-leucyl)carbonyl]-1*H*-imidazole
**5{***61*}	4-[(*S*-α-methylbenzylamino)carbonyl]-5-[(*tert*-butoxy-*S*-leucyl)carbonyl]-1*H*-imidazole
**5{***62*}	4-[4-({[(tert-butoxy)carbonyl]piperidinyl}amino)carbonyl]-5-[(*tert*-butoxy-*S*-leucyl)carbonyl]-1*H*-imidazole
**5{***63*}	4-{[(4-{[(*tert*-butoxy)carbonyl]aminomethyl}phenyl)methylamino]carbonyl}-5-[(*tert*-butoxy-*S*-leucyl)carbonyl]-1*H*-imidazole
**5{***64*}	4-[(dibenzylamino)carbonyl]-5-[(*tert*-butoxy-*S*-leucyl)carbonyl]-1*H*-imidazole
**5{***65*}	4-[(*N*-methylbenzylamino)carbonyl]-5-[(*tert*-butoxy-*S*-leucyl)carbonyl]-1*H*-imidazole
**5{***66*}	4-[(*N,N*-diethylamino)carbonyl]-5-[(*tert*-butoxy-*S*-leucyl)carbonyl]-1*H*-imidazole
**5{***67*}	4-({4-[(*tert*-butoxy)carbonyl]piperazinyl}carbonyl)-5-[(*tert*-butoxy-*S*-leucyl)carbonyl]-1*H*-imidazole
**5{***68*}	4-[(1,2,3,4-tetrahydroisoquinolinyl)carbonyl]-5-[(*tert*-butoxy-*S*-leucyl)carbonyl]-1*H*-imidazole
**5{***69*}	4-[(4-methylpiperidinyl)carbonyl]-5-[(*tert*-butoxy-*S*-leucyl)carbonyl]-1*H*-imidazole
**5{***70*}	4-({4-[(*tert*-butoxy)carbonyl]piperazinyl}carbonyl)-5-[(*tert*-butoxy-*S*-leucyl)carbonyl]-1*H*-imidazole
**5{***71*}	4-[(methylamino)carbonyl]-5-[(benzyloxy- *S*-leucyl)carbonyl]-1*H*-imidazole
**5{***72*}	4-[5-({[(tert-butoxy)carbonyl]amino}pentylamino)carbonyl]-5-[(benzyloxy- *S*-leucyl)carbonyl]-1*H*-imidazole
**5{***73*}	4-[(benzylamino)carbonyl]-5-[(benzyloxy- *S*-leucyl)carbonyl]-1*H*-imidazole
**5{***74*}	4-[(*R*-α-methylbenzylamino)carbonyl]-5-[(benzyloxy-*S*-leucyl)carbonyl]-1*H*-imidazole
**5{***75*}	4-[(*S*-α-methylbenzylamino)carbonyl]-5-[(benzyloxy-*S*-leucyl)carbonyl]-1*H*-imidazole
**5{***76*}	4-[4-({[(tert-butoxy)carbonyl]piperidinyl}amino)carbonyl]-5-[(benzyloxy- *S*-leucyl)carbonyl]-1*H*-imidazole
**5{***77*}	4-{[(4-{[(*tert*-butoxy)carbonyl]aminomethyl}phenyl)methylamino]carbonyl}-5-[(benzyloxy-*S*-leucyl)carbonyl]-1*H*-imidazole
**5{***78*}	4-[(dibenzylamino)carbonyl]-5-[(benzyloxy-S-leucyl)carbonyl]-1 *H*-imidazole
**5{***79*}	4-[(*N*-methylbenzylamino)carbonyl]-5-[(benzyloxy-*S*-leucyl)carbonyl]-1*H*-imidazole
**5{***80*}	4-[(*N,N*-diethylamino)carbonyl]-5-[(benzyloxy-*S*-leucyl)carbonyl]-1*H*-imidazole
**5{***81*}	4-({4-[(*tert*-butoxy)carbonyl]piperazinyl}carbonyl)-5-[(benzyloxy-*S*-leucyl)carbonyl]-1*H*-imidazole
**5{***82*}	4-[(1,2,3,4-tetrahydroisoquinolinyl)carbonyl]-5-[(benzyloxy- *S*-leucyl)carbonyl]-1*H*-imidazole
**5{***83*}	4-[(4-methylpiperidinyl)carbonyl]-5-[(benzyloxy- *S*-leucyl)carbonyl]-1*H*-imidazole
**5{***84*}	4-({4-[(*tert*-butoxy)carbonyl]piperazinyl}carbonyl)-5-[(benzyloxy-*S*-leucyl)carbonyl]-1*H*-imidazole
**5{***85*}	4-[(methylamino)carbonyl]-5-[(*tert*-butoxy-*S*-phenylalanyl)carbonyl]-1*H*-imidazole
**5{***86*}	4-[5-({[(tert-butoxy)carbonyl]amino}pentylamino)carbonyl]-5-[(*tert*-butoxy-*S*-phenylalanyl)carbonyl]-1*H*-imidazole
**5{***87*}	4-[(benzylamino)carbonyl]-5-[(*tert*-butoxy-*S*-phenylalanyl)carbonyl]-1*H*-imidazole
**5{***88*}	4-[(*R*-α-methylbenzylamino)carbonyl]-5-[(*tert*-butoxy-*S*-phenylalanyl)carbonyl]-1*H*-imidazole
**5{***89*}	4-[(*S*-α-methylbenzylamino)carbonyl]-5-[(*tert*-butoxy-*S*-phenylalanyl)carbonyl]-1*H*-imidazole
**5{***90*}	4-[4-({[(tert-butoxy)carbonyl]piperidinyl}amino)carbonyl]-5-[(*tert*-butoxy-*S*-phenylalanyl)carbonyl]-1*H*-imidazole
**5{***91*}	4-{[(4-{[(*tert*-butoxy)carbonyl]aminomethyl}phenyl)methylamino]carbonyl}-5-[(*tert*-butoxy-*S*-phenylalanyl)carbonyl]-1*H*-imidazole
**5{***92*}	4-[(dibenzylamino)carbonyl]-5-[(*tert*-butoxy-*S*-phenylalanyl)carbonyl]-1*H*-imidazole
**5{***93*}	4-[(*N*-methylbenzylamino)carbonyl]-5-[(*tert*-butoxy-*S*-phenylalanyl)carbonyl]-1*H*-imidazole
**5{***94*}	4-[(*N,N*-diethylamino)carbonyl]-5-[(*tert*-butoxy-*S*-phenylalanyl)carbonyl]-1*H*-imidazole
**5{***95*}	4-({4-[(*tert*-butoxy)carbonyl]piperazinyl}carbonyl)-5-[(*tert*-butoxy-*S*-phenylalanyl)carbonyl]-1*H*-imidazole
**5{***96*}	4-[(1,2,3,4-tetrahydroisoquinolinyl)carbonyl]-5-[(*tert*-butoxy-*S*-phenylalanyl)carbonyl]-1*H*-imidazole
**5{***97*}	4-[(4-methylpiperidinyl)carbonyl]-5-[(*tert*-butoxy-*S*-phenylalanyl)carbonyl]-1*H*-imidazole
**5{***98*}	4-({4-[(*tert*-butoxy)carbonyl]piperazinyl}carbonyl)-5-[(*tert*-butoxy-*S*-phenylalanyl)carbonyl]-1*H*-imidazole
**5{***99*}	4-[(methylamino)carbonyl]-5-[(benzyloxy- *S*-phenylalanyl)carbonyl]-1*H*-imidazole
**5{***100*}	4-[5-({[(tert-butoxy)carbonyl]amino}pentylamino)carbonyl]-5-[(benzyloxy- *S*-phenylalanyl)carbonyl]-1*H*-imidazole
**5{***101*}	4-[(benzylamino)carbonyl]-5-[(benzyloxy- *S*-phenylalanyl)carbonyl]-1*H*-imidazole
**5{***102*}	4-[(*R*-α-methylbenzylamino)carbonyl]-5-[(benzyloxy-*S*-phenylalanyl)carbonyl]-1*H*-imidazole
**5{***103*}	4-[(*S*-α-methylbenzylamino)carbonyl]-5-[(benzyloxy-*S*-phenylalanyl)carbonyl]-1*H*-imidazole
**5{***104*}	4-[4-({[(tert-butoxy)carbonyl]piperidinyl}amino)carbonyl]-5-[(benzyloxy- *S*-phenylalanyl)carbonyl]-1*H*-imidazole
**5{***105*}	4-{[(4-{[(*tert*-butoxy)carbonyl]aminomethyl}phenyl)methylamino]carbonyl}-5-[(benzyloxy-*S*-phenylalanyl)carbonyl]-1*H*-imidazole
**5{***106*}	4-[(dibenzylamino)carbonyl]-5-[(benzyloxy-S-phenylalanyl)carbonyl]-1 *H*-imidazole
**5{***107*}	4-[(*N*-methylbenzylamino)carbonyl]-5-[(benzyloxy-*S*-phenylalanyl)carbonyl]-1*H*-imidazole
**5{***108*}	4-[(*N,N*-diethylamino)carbonyl]-5-[(benzyloxy-*S*-phenylalanyl)carbonyl]-1*H*-imidazole
**5{***109*}	4-({4-[(*tert*-butoxy)carbonyl]piperazinyl}carbonyl)-5-[(benzyloxy-*S*-phenylalanyl)carbonyl]-1*H*-imidazole
**5{***110*}	4-[(1,2,3,4-tetrahydroisoquinolinyl)carbonyl]-5-[(benzyloxy- *S*-phenylalanyl)carbonyl]-1*H*-imidazole
**5{***111*}	4-[(4-methylpiperidinyl)carbonyl]-5-[(benzyloxy- *S*-phenylalanyl)carbonyl]-1*H*-imidazole
**5{***112*}	4-({4-[(*tert*-butoxy)carbonyl]piperazinyl}carbonyl)-5-[(benzyloxy-*S*-phenylalanyl)carbonyl]-1*H*-imidazole
**5{***113*}	4-[(methylamino)carbonyl]-5-[(*tert*-butoxy-*S*-[*N*^e^ -(*tert*-butoxy)carbonyl]lysyl)carbonyl]-1*H*-imidazole
**5{***114*}	4-[5-({[(tert-butoxy)carbonyl]amino}pentylamino)carbonyl]-5-[(*tert*-butoxy-*S*-[*N*^e^ -(*tert*-butoxy)carbonyl]lysyl)carbonyl]-1*H*-imidazole
**5{***115*}	4-[(benzylamino)carbonyl]-5-[(*tert*-butoxy-*S*-[*N*^e^ -(*tert*-butoxy)carbonyl]lysyl)carbonyl]-1*H*-imidazole
**5{***116*}	4-[(*R*-α-methylbenzylamino)carbonyl]-5-[(*tert*-butoxy-*S*-[*N*^e^ -(*tert*-butoxy)carbonyl]lysyl)carbonyl]-1*H*-imidazole
**5{***117*}	4-[(*S*-α-methylbenzylamino)carbonyl]-5-[(*tert*-butoxy-*S*-[*N*^e^ -(*tert*-butoxy)carbonyl]lysyl)carbonyl]-1*H*-imidazole
**5{***118*}	4-[4-({[(tert-butoxy)carbonyl]piperidinyl}amino)carbonyl]-5-[(*tert*-butoxy-*S*-[*N*^e^ -(*tert*-butoxy)carbonyl]lysyl)carbonyl]-1*H*-imidazole
**5{***119*}	4-{[(4-{[(*tert*-butoxy)carbonyl]aminomethyl}phenyl)methylamino]carbonyl}-5-[(*tert*-butoxy-*S*-[*N*^e^ -(*tert*-butoxy)carbonyl]lysyl)carbonyl]-1*H*-imidazole
**5{***120*}	4-[(dibenzylamino)carbonyl]-5-[(*tert*-butoxy-*S*-[*N*^e^ -(*tert*-butoxy)carbonyl]lysyl)carbonyl]-1*H*-imidazole
**5{***121*}	4-[(*N*-methylbenzylamino)carbonyl]-5-[(*tert*-butoxy-*S*-[*N*^e^ -(*tert*-butoxy)carbonyl]lysyl)carbonyl]-1*H*-imidazole
**5{***122*}	4-[(*N,N*-diethylamino)carbonyl]-5-[(*tert*-butoxy-*S*-[*N*^e^ -(*tert*-butoxy)carbonyl]lysyl)carbonyl]-1*H*-imidazole
**5{***123*}	4-({4-[(*tert*-butoxy)carbonyl]piperazinyl}carbonyl)-5-[(*tert*-butoxy-*S*-[*N*^e^ -(*tert*-butoxy)carbonyl]lysyl)carbonyl]-1*H*-imidazole
**5{***124*}	4-[(1,2,3,4-tetrahydroisoquinolinyl)carbonyl]-5-[(*tert*-butoxy-*S*-[*N*^e^ -(*tert*-butoxy)carbonyl]lysyl)carbonyl]-1*H*-imidazole
**5{***125*}	4-[(4-methylpiperidinyl)carbonyl]-5-[(*tert*-butoxy-*S*-[*N*^e^ -(*tert*-butoxy)carbonyl]lysyl)carbonyl]-1*H*-imidazole
**5{***126*}	4-({4-[(*tert*-butoxy)carbonyl]piperazinyl}carbonyl)-5-[(*tert*-butoxy-*S*-[*N*^e^ -(*tert*-butoxy)carbonyl]lysyl)carbonyl]-1*H*-imidazole

## Supplementary Materials

Supplementary File 1

## References

[1] Kaiser M., Wetzel S., Kumar K., Waldmann H. (2008). Biology-inspired synthesis of compound libraries. Cell Mol. Life Sci..

[2] Huryn D.M., Cosford N.D.P. (2007). The Molecular Libraries Screening Center Network (MLSCN): Identifying chemical probes of biological systems. Ann. Rep. Med. Chem..

[3] Winssinger N., Pianowski Z., Debaene F. (2007). Probing biology with Small Molecule Microarrays (SMM). Top. Curr. Chem..

[4] Haggarty S.J., Schreiber S.L., Schreiber S.L., Kapoor T.M., Weiss G. (2007). Forward chemical genetics. Chemical Biology. From Small Molecules to Systems Biology and Drug Design.

[5] Lipinski C.A. (2000). Drug-like properties and the causes of poor solubility and poor permeability. J. Pharmacol. Toxicol. Methods.

[6] Veber D.F., Johnson S.R., Cheng H.Y., Smith B.R., Ward K.W., Kopple K.D. (2002). Molecular properties that influence the oral bioavailability of drug candidates. J. Med. Chem..

[7] Lipinski C.A., Lombardo F., Dominy B.W., Feeney P.J. (2001). Experimental and computational approaches to estimate solubility and permeability in drug discovery and development settings. Adv. Drug Deliv. Rev..

[8] Lipinski C.A. (2004). Lead- and drug-like compounds: the rule of five revolution. Drug Discov. Today.

[9] Baures P.W. (2006). Imidazole-4,5-dicarboxylic acid: A versatile scaffold for drug discovery and materials research. Trends Heterocyc. Chem..

[10] Baures P.W., Caldwell A.W., Cashman C.R., Masse M.T., Van Arnam E.B., Conry R.R. (2006). The influence by substituents on the intermolecular hydrogen-bonding interactions in imidazole-4,5-dicarboxylic acid derivatives. Cryst. Growth Des..

[11] Rush J.R., Sandstrom S.L., Yang J., Davis R., Prakash O., Baures P.W. (2005). Intramolecular hydrogen bond strength and pKa determination of *N*,*N*’-disubstituted imidazole-4,5-dicarboxamides. Org. Lett..

[12] Baures P.W., Rush J.R., Wiznycia A.V., Desper J., Helfrich B.A., Beatty A.M. (2002). Intramolecular hydrogen bonding and intermolecular dimerization in the crystal structures of imidazole-4,5-dicarboxylic acid derivatives. Cryst. Growth Des..

[13] Legraverend M., Grierson D.S. (2006). The purines: potent and versatile small molecule inhibitors and modulators of key biological targets. Bioorg. Med. Chem..

[14] Wiznycia A.V., Helfrich B.A., Baures P.W. (2002). An improved method for the synthesis of dissymmetric *N*,*N*’-disubstituted imidazole-4,5-dicarboxamides. J. Org. Chem..

[15] Perchellet E.M., Perchellet J.P., Baures P.W. (2005). Imidazole-4,5-dicarboxamides with antiproliferative activity against HL-60 cells. J. Med. Chem..

[16] Wiznycia A.V., Rush J.R., Baures P.W. (2004). Synthesis of symmetric bis-imidazole-4,5-dicarboxamides bearing amino acids. J. Org. Chem..

[17] Zerhouni E. (2003). Medicine: The NIH Roadmap. Science.

[B18-molecules-13-03149] 18.A website with data for these compounds and background information for the overall project is found at the following link: http://www.imidazole.utulsa.edu.

[19] Angell Y., Chen D., Brahimi F., Saragovi H.U., Burgess K. (2008). A combinatorial method for solution-phase synthesis of labeled bivalent beta-turn mimics. J. Am. Chem. Soc..

[20] Fenster E., Rayabarapu D.K., Zhang M., Mukherjee S., Hill D., Neuenswander B., Schoenen F., Hanson P.R., Aube J. (2008). Three-component synthesis of 1,4-diazepin-5-ones and the construction of gamma-turn-like peptidomimetic libraries. J. Comb. Chem..

[21] Vedantham P., Zhang M., Gor P.J., Huang M., Georg G.I., Lushington G.H., Mitscher L.A., Ye Q.-Z., Hanson P.R. (2008). Studies towards the synthesis of methionine aminopeptidase inhibitors: diversification utilizing a ROMP-derived coupling reagent. J. Comb. Chem..

[22] Carpenter R.D., DeBerdt P.B., Holden J.B., Milinkevich K.A., Min T., Willenbring D., Fettinger J.C., Tantillo D.J., Kurth M.J. (2008). Design and synthesis of propeller-shaped dispiroisoxazolinopiperidinochromanones. J. Comb. Chem..

[23] Butler J.D., Solano D.M., Robins L.I., Haddadin M.J., Kurth M.J. (2008). A facile synthesis of new 5*H*-indazolo[3,2-b]benzo[d]-1,3-oxazines via one-pot intramolecular Bis-heterocyclizations. J. Org. Chem..

[24] Jeddeloh M.R., Holden J.B., Nouri D.H., Kurth M.J. (2007). A library of 3-aryl-4,5-dihydroisoxazole-5-carboxamides. J. Comb. Chem..

[25] Comer E., Rohan E., Deng L., Porco J.A. (2007). An approach to skeletal diversity using functional group pairing of multifunctional scaffolds. Org. Lett..

[26] Nielsen T.E., Schreiber S.L. (2008). Towards the optimal screening collection: a synthesis strategy. Angew. Chem. Int. Ed..

[27] Shang S., Iwadare H., Macks D.E., Ambrosini L.M., Tan D.S. (2007). A unified synthetic approach to polyketides having both skeletal and stereochemical diversity. Org. Lett..

[28] Houghten R.A., Pinilla C., Giulianotti M.A., Appel J.R., Dooley C.T., Nefzi A., Ostresh J.M., Yu Y., Maggiora G.M., Medina-Franco J.L., Brunner D., Schneider J. (2008). Strategies for the use of mixture-based synthetic combinatorial libraries: scaffold ranking, direct testing in vivo, and enhanced deconvolution by computational methods. J. Comb. Chem..

[29] Krchnák V., Moellmann U., Dahse H.M., Miller M.J. (2008). Solid-supported nitroso hetero Diels-Alder reactions. 1. Acylnitroso dienophiles: scope and limitations. J. Comb. Chem..

[30] Bouillon I., Krchnák V. (2007). Efficient solid-phase synthesis of 3-substituted-5-oxo-5H-thiazolo[2,3-b]-quinazoline-8-carboxamides under mild conditions with two diversity positions. J. Comb. Chem..

[31] Just Z.W., Larock R.C. (2008). Synthesis of 2(3*H*)-furanones via electrophilic cyclization. J. Org. Chem..

[B32-molecules-13-03149] 32.The PubChem database can be accessed at the following link: http://www.ncbi.nlm.nih.gov/PubMed.

[33] Hur J., Wild D.J. (2008). PubChemSR: A search and retrieval tool for PubChem. Chem. Cent. J..

