# Supplementary material for: Parallel Synthesis of an Imidazole-4,5-dicarboxamide Library Bearing Amino Acid Esters and Alkanamines
Source: Molecules. 2008 Dec 15;13(12):3149–70. doi: 10.3390/molecules13123149 (PMC2651155; doi:10.3390/molecules13123149)
Supplement: Supplementary File 1 [file molecules-13-03149-s001.pdf]

# Supporting Information

# for

# Parallel Synthesis of an Imidazole-4,5-dicarboxamide Library Bearing Amino Acid Esters and Alkanamines

**Rosanna Solinas, John C. DiCesare, and Paul W. Baures**[§]

Department of Chemistry and Biochemistry, The University of Tulsa, 800 South Tucker Drive, Tulsa, OK 74104

## Table of Contents

Page S1-3. Title Page and Table of Contents
Page S4. **Table S1.** Amino Acid Ester—Alkanamine Disubstituted I45DCs, **5**{*1-28*}.
Page S5. **Table S2.** Amino Acid Ester—Alkanamine Disubstituted I45DCs, **5**{*29-56*}.
Page S6. **Table S3.** Amino Acid Ester—Alkanamine Disubstituted I45DCs, **5**{*57-84*}.
Page S7. **Table S4.** Amino Acid Ester—Alkanamine Disubstituted I45DCs, **5**{*85-112*}.
Page S8. **Table S5.** Amino Acid Ester—Alkanamine Disubstituted I45DCs, **5**{*113-126*}.
Page S9. **Figure S1.** LC/MS data for **5{**1}.
Page S10. **Figure S2.** LC/MS data for **5{**2}.
Page S11. **Figure S3.** LC/MS data for **5{**3}.
Page S12. **Figure S4.** LC/MS data for **5{**4}.
Page S13. **Figure S5.** LC/MS data for **5{**5}.
Page S14. **Figure S6.** LC/MS data for **5{**6}.
Page S15. **Figure S7.** LC/MS data for **5{**7}.
Page S16. **Figure S8.** LC/MS data for **5{**8}.
Page S17. **Figure S9.** LC/MS data for **5{**9}.
Page S18. **Figure S10.** LC/MS data for **5{**10}.
Page S19. **Figure S11.** LC/MS data for **5{**11}.
Page S20. **Figure S12.** LC/MS data for **5{**12}.
Page S21. **Figure S13.** LC/MS data for **5{**13}.
Page S22. **Figure S14.** LC/MS data for **5{**14}.
Page S23. **Figure S15.** LC/MS data for **5{**15}.
Page S24. **Figure S16.** LC/MS data for **5{**16}.
Page S25. **Figure S17.** LC/MS data for **5{**17}.
Page S26. **Figure S18.** LC/MS data for **5{**18}.
Page S27. **Figure S19.** LC/MS data for **5{**19}.
Page S28. **Figure S20.** LC/MS data for **5{**20}.
Page S29. **Figure S21.** LC/MS data for **5{**21}.
Page S30. **Figure S22.** LC/MS data for **5{**22}.
Page S31. **Figure S23.** LC/MS data for **5{**23}.
Page S32. **Figure S24.** LC/MS data for **5{**24}.
Page S33. **Figure S25.** LC/MS data for **5{**25}.
Page S34. **Figure S26.** LC/MS data for **5{**26}.
Page S35. **Figure S27.** LC/MS data for **5{**27}.
Page S36. **Figure S28.** LC/MS data for **5{**28}.
Page S37. **Figure S29.** LC/MS data for **5{**29}.
Page S38. **Figure S30.** LC/MS data for **5{**30}.
Page S39. **Figure S31.** LC/MS data for **5{**31}.
Page S40. **Figure S32.** LC/MS data for **5{**32}.
Page S41. **Figure S33.** LC/MS data for **5{**33}.
Page S42. **Figure S34.** LC/MS data for **5{**34}.
Page S43. **Figure S35.** LC/MS data for **5{**35}.
Page S44. **Figure S36.** LC/MS data for **5{**36}.
Page S45. **Figure S37.** LC/MS data for **5{**37}.
Page S46. **Figure S38.** LC/MS data for **5{**38}.

Page S47. **Figure S39.** LC/MS data for **5{**39}.
Page S48. **Figure S40.** LC/MS data for **5{**40}.
Page S49. **Figure S41.** LC/MS data for **5{**41}.
Page S50. **Figure S42.** LC/MS data for **5{**42}.
Page S51. **Figure S43.** LC/MS data for **5{**43}.
Page S52. **Figure S44.** LC/MS data for **5{**44}.
Page S53. **Figure S45.** LC/MS data for **5{**45}.
Page S54. **Figure S46.** LC/MS data for **5{**46}.
Page S55. **Figure S47.** LC/MS data for **5{**47}.
Page S56. **Figure S48.** LC/MS data for **5{**48}.
Page S57. **Figure S49.** LC/MS data for **5{**49}.
Page S58. **Figure S50.** LC/MS data for **5{**50}.
Page S59. **Figure S51.** LC/MS data for **5{**51}.
Page S60. **Figure S52.** LC/MS data for **5{**52}.
Page S61. **Figure S53.** LC/MS data for **5{**53}.
Page S62. **Figure S54.** LC/MS data for **5{**54}.
Page S63. **Figure S55.** LC/MS data for **5{**55}.
Page S64. **Figure S56.** LC/MS data for **5{**56}.
Page S65. **Figure S57.** LC/MS data for **5{**57}.
Page S66. **Figure S58.** LC/MS data for **5{**58}.
Page S67. **Figure S59.** LC/MS data for **5{**59}.
Page S68. **Figure S60.** LC/MS data for **5{**60}.
Page S69. **Figure S61.** LC/MS data for **5{**61}.
Page S70. **Figure S62.** LC/MS data for **5{**62}.
Page S71. **Figure S63.** LC/MS data for **5{**63}.
Page S72. **Figure S64.** LC/MS data for **5{**64}.
Page S73. **Figure S65.** LC/MS data for **5{**65}.
Page S74. **Figure S66.** LC/MS data for **5{**66}.
Page S75. **Figure S67.** LC/MS data for **5{**67}.
Page S76. **Figure S68.** LC/MS data for **5{**68}.
Page S77. **Figure S69.** LC/MS data for **5{**69}.
Page S78. **Figure S70.** LC/MS data for **5{**70}.
Page S79. **Figure S71.** LC/MS data for **5{**71}.
Page S80. **Figure S72.** LC/MS data for **5{**72}.
Page S81. **Figure S73.** LC/MS data for **5{**73}.
Page S82. **Figure S74.** LC/MS data for **5{**74}.
Page S83. **Figure S75.** LC/MS data for **5{**75}.
Page S84. **Figure S76.** LC/MS data for **5{**76}.
Page S85. **Figure S77.** LC/MS data for **5{**77}.
Page S86. **Figure S78.** LC/MS data for **5{**78}.
Page S87. **Figure S79.** LC/MS data for **5{**79}.
Page S88. **Figure S80.** LC/MS data for **5{**80}.
Page S89. **Figure S81.** LC/MS data for **5{**81}.
Page S90. **Figure S82.** LC/MS data for **5{**82}.
Page S91. **Figure S83.** LC/MS data for **5{**83}.
Page S92. **Figure S84.** LC/MS data for **5{**84}.
Page S93. **Figure S85.** LC/MS data for **5{**85}.
Page S94. **Figure S86.** LC/MS data for **5{**86}.
Page S95. **Figure S87.** LC/MS data for **5{**87}.
Page S96. **Figure S88.** LC/MS data for **5{**88}.
Page S97. **Figure S89.** LC/MS data for **5{**89}.
Page S98. **Figure S90.** LC/MS data for **5{**90}.
Page S99. **Figure S91.** LC/MS data for **5{**91}.
Page S100. **Figure S92.** LC/MS data for **5{**92}.
Page S101. **Figure S93.** LC/MS data for **5{**93}.
Page S102. **Figure S94.** LC/MS data for **5{**94}.
Page S103. **Figure S95.** LC/MS data for **5{**95}.
Page S104. **Figure S96.** LC/MS data for **5{**96}.
Page S105. **Figure S97.** LC/MS data for **5{**97}.
Page S106. **Figure S98.** LC/MS data for **5{**98}.
Page S107. **Figure S99.** LC/MS data for **5{**99}.
Page S108. **Figure S100.** LC/MS data for **5{**100}.
Page S109. **Figure S101.** LC/MS data for **5{**101}.
Page S110. **Figure S102.** LC/MS data for **5{**102}.
Page S111. **Figure S103.** LC/MS data for **5{**103}.
Page S112. **Figure S104.** LC/MS data for **5{**104}.
Page S113. **Figure S105.** LC/MS data for **5{**105}.
Page S114. **Figure S106.** LC/MS data for **5{**106}.
Page S115. **Figure S107.** LC/MS data for **5{**107}.
Page S116. **Figure S108.** LC/MS data for **5{**108}.
Page S117. **Figure S109.** LC/MS data for **5{**109}.
Page S118. **Figure S110.** LC/MS data for **5{**110}.
Page S119. **Figure S111.** LC/MS data for **5{**111}.
Page S120. **Figure S112.** LC/MS data for **5{**112}.
Page S121. **Figure S113.** LC/MS data for **5{**113}.
Page S122. **Figure S114.** LC/MS data for **5{**114}.
Page S123. **Figure S115.** LC/MS data for **5{**115}.
Page S124. **Figure S116.** LC/MS data for **5{**116}.
Page S125. **Figure S117.** LC/MS data for **5{**117}.
Page S126. **Figure S118.** LC/MS data for **5{**118}.
Page S127. **Figure S119.** LC/MS data for **5{**119}.
Page S128. **Figure S120.** LC/MS data for **5{**120}.
Page S129. **Figure S121.** LC/MS data for **5{**121}.
Page S130. **Figure S122.** LC/MS data for **5{**122}.
Page S131. **Figure S123.** LC/MS data for **5{**123}.
Page S132. **Figure S124.** LC/MS data for **5{**124}.
Page S133. **Figure S125.** LC/MS data for **5{**125}.
Page S134. **Figure S126.** LC/MS data for **5{**126}.
Page S135. **Figure S127.** LC/MS data for the crude reaction to yield **5{**2}.
Page S136. **Figure S128.** LC/MS data for the crude reaction to yield **5{**6}.
Page S137. **Figure S129.** LC/MS data for the crude reaction to yield **5{**26}.
Page S138. **Figure S130.** LC/MS data for the crude reaction to yield **5{**28}.
Page S139. **Figure S131.** LC/MS data for the crude reaction to yield **5{**38}.

Page S140. **Figure S132.** LC/MS data for the crude reaction to yield **5{**39}.
Page S141. **Figure S133.** LC/MS data for the crude reaction to yield **5{**44}.
Page S142. **Figure S134.** LC/MS data for the crude reaction to yield **5{**48}.
Page S143. **Figure S135.** LC/MS data for the crude reaction to yield **5{**59}.
Page S144. **Figure S136.** LC/MS data for the crude reaction to yield **5{**63}.
Page S145. **Figure S137.** LC/MS data for the crude reaction to yield **5{**72}.
Page S146. **Figure S138.** LC/MS data for the crude reaction to yield **5{**74}.
Page S147. **Figure S139.** LC/MS data for the crude reaction to yield **5{**93}.
Page S148. **Figure S140.** LC/MS data for the crude reaction to yield **5{**97}.
Page S149. **Figure S141.** LC/MS data for the crude reaction to yield **5{**100}.
Page S150. **Figure S142.** LC/MS data for the crude reaction to yield **5{**104}.
Page S151. **Figure S143.** LC/MS data for the crude reaction to yield **5{**119}.
Page S152. **Figure S144.** LC/MS data for the crude reaction to yield **5{**126}.
Page S153. **Figure S145.** $^1$H-NMR for **5{**2}.
Page S154. **Figure S146.** $^1$H-NMR for **5{**6}.
Page S155. **Figure S147.** $^1$H-NMR for **5{**26}.
Page S156. **Figure S148.** $^1$H-NMR for **5{**28}.
Page S157. **Figure S149.** $^1$H-NMR for **5{**38}.
Page S158. **Figure S150.** $^1$H-NMR for **5{**39}.
Page S159. **Figure S151.** $^1$H-NMR for **5{**44}.
Page S160. **Figure S152.** $^1$H-NMR for **5{**48}.
Page S161. **Figure S153.** $^1$H-NMR for **5{**59}.
Page S162. **Figure S154.** $^1$H-NMR for **5{**63}.
Page S163. **Figure S155.** $^1$H-NMR for **5{**72}.
Page S164. **Figure S156.** $^1$H-NMR for **5{**74}.
Page S165. **Figure S157.** $^1$H-NMR for **5{**93}.
Page S166. **Figure S158.** $^1$H-NMR for **5{**97}.
Page S167. **Figure S159.** $^1$H-NMR for **5{**100}.
Page S168. **Figure S160.** $^1$H-NMR for **5{**104}.
Page S169. **Figure S161.** $^1$H-NMR for **5{**119}.
Page S170. **Figure S162.** $^1$H-NMR for **5{**126}.
Page S171. **Figure S163.** $^1$H-NMR for the crude reaction to yield **5{**26}.
Page S172. **Figure S164.** $^1$H-NMR for the crude reaction to yield **5{**2}.
Page S173. **Figure S165.** $^1$H-NMR for the crude reaction to yield **5{**6}.
Page S174. **Figure S166.** $^1$H-NMR for the crude reaction to yield **5{**26}.
Page S175. **Figure S167.** $^1$H-NMR for the crude reaction to yield **5{**28}.
Page S176. **Figure S168.** $^1$H-NMR for the crude reaction to yield **5{**39}.
Page S177. **Figure S169.** $^1$H-NMR for the crude reaction to yield **5{**44}.
Page S178. **Figure S170.** $^1$H-NMR for the crude reaction to yield **5{**48}.
Page S179. **Figure S171.** $^1$H-NMR for the crude reaction to yield **5{**59}.
Page S180. **Figure S172.** $^1$H-NMR for the crude reaction to yield **5{**63}.
Page S181. **Figure S173.** $^1$H-NMR for the crude reaction to yield **5{**72}.
Page S182. **Figure S174.** $^1$H-NMR for the crude reaction to yield **5{**74}.
Page S183. **Figure S175.** $^1$H-NMR for the crude reaction to yield **5{**93}.
Page S184. **Figure S176.** $^1$H-NMR for the crude reaction to yield **5{**97}.
Page S185. **Figure S177.** $^1$H-NMR for the crude reaction to yield **5{**100}.
Page S186. **Figure S178.** $^1$H-NMR for the crude reaction to yield **5{**104}.
Page S187. **Figure S179.** $^1$H-NMR for the crude reaction to yield **5{**119}.
Page S188. **Figure S180.** $^1$H-NMR for the crude reaction to yield **5{**126}.

**Table S1.** Amino Acid Ester—Alkanamine Disubstituted I45DCs, **5**{*1-28*}.

| compound | formula | MW | C log*P* | form | $R_f$ | $R_t$ (min) |
|---|---|---|---|---|---|---|
| **5**{*1*} | $C_{12}H_{18}N_4O_4$ | 282.30 | -0.88 | glass film | 0.16 | 1.50 |
| **5**{*2*} | $C_{21}H_{35}N_5O_6$ | 453.53 | 1.09 | glass film | 0.22 | 3.70 |
| **5**{*3*} | $C_{18}H_{22}N_4O_4$ | 358.39 | 1.42 | solid | 0.27 | 3.45 |
| **5**{*4*} | $C_{19}H_{24}N_4O_4$ | 372.42 | 1.39 | glass film | 0.26 | 4.10 |
| **5**{*5*} | $C_{19}H_{24}N_4O_4$ | 372.42 | 1.39 | glass film | 0.26 | 4.10 |
| **5**{*6*} | $C_{21}H_{33}N_5O_6$ | 451.52 | 1.00 | solid | 0.15 | 3.95 |
| **5**{*7*} | $C_{24}H_{33}N_5O_6$ | 487.55 | 2.13 | glass film | 0.25 | 4.30 |
| **5**{*8*} | $C_{24}H_{26}N_4O_4$ | 434.49 | 2.77 | glass film | 0.33 | 5.52 |
| **5**{*9*} | $C_{19}H_{24}N_4O_4$ | 372.42 | 2.05 | solid | 0.19 | 2.40 |
| **5**{*10*} | $C_{15}H_{24}N_4O_4$ | 324.38 | 0.92 | glass film | 0.26 | 1.65 |
| **5**{*11*} | $C_{20}H_{31}N_5O_6$ | 437.49 | 2.00 | solid | 0.14 | 3.18 |
| **5**{*12*} | $C_{20}H_{24}N_4O_4$ | 384.43 | 2.34 | glass film | 0.23 | 2.50 |
| **5**{*13*} | $C_{17}H_{26}N_4O_4$ | 350.41 | 1.58 | solid | 0.18 | 2.05 |
| **5**{*14*} | $C_{21}H_{27}N_5O_4$ | 413.47 | 1.20 | solid | 0.17 | 2.32 |
| **5**{*15*} | $C_{15}H_{16}N_4O_4$ | 316.31 | -0.07 | solid | 0.20 | 1.60 |
| **5**{*16*} | $C_{24}H_{33}N_5O_6$ | 487.55 | 1.90 | glass film | 0.22 | 2.15 |
| **5**{*17*} | $C_{21}H_{20}N_4O_4$ | 392.41 | 1.56 | solid | 0.46 | 3.80 |
| **5**{*18*} | $C_{22}H_{22}N_4O_4$ | 406.43 | 1.87 | glass film | 0.20 | 4.85 |
| **5**{*19*} | $C_{22}H_{22}N_4O_4$ | 406.43 | 1.87 | glass film | 0.20 | 4.85 |
| **5**{*20*} | $C_{24}H_{31}N_5O_6$ | 485.53 | 1.13 | glass film | 0.31 | 4.25 |
| **5**{*21*} | $C_{27}H_{31}N_5O_6$ | 521.56 | 2.26 | solid | 0.24 | 4.47 |
| **5**{*22*} | $C_{27}H_{24}N_4O_4$ | 468.50 | 2.91 | glass film | 0.59 | 5.85 |
| **5**{*23*} | $C_{22}H_{22}N_4O_4$ | 406.43 | 2.53 | glass film | 0.32 | 2.58 |
| **5**{*24*} | $C_{19}H_{22}N_4O_4$ | 358.39 | 1.40 | glass film | 0.32 | 1.75 |
| **5**{*25*} | $C_{23}H_{29}N_5O_6$ | 471.51 | 2.48 | solid | 0.20 | 2.35 |
| **5**{*26*} | $C_{23}H_{22}N_4O_4$ | 418.45 | 2.81 | solid | 0.23 | 2.85 |
| **5**{*27*} | $C_{20}H_{24}N_4O_4$ | 384.43 | 2.05 | glass film | 0.24 | 3.30 |
| **5**{*28*} | $C_{24}H_{25}N_5O_4$ | 447.49 | 1.68 | solid | 0.22 | 3.95 |

**Table S2.** Amino Acid Ester—Alkanamine Disubstituted I45DCs, **5**{*29-56*}.

| compound | formula | MW | C log*P* | form | $R_f$ | $R_t$ (min) |
|---|---|---|---|---|---|---|
| **5**{*29*} | $C_{13}H_{20}N_4O_4$ | 296.32 | -0.58 | glass film | 0.14 | 1.70 |
| **5**{*30*} | $C_{22}H_{37}N_5O_6$ | 467.56 | 1.40 | glass film | 0.14 | 4.40 |
| **5**{*31*} | $C_{19}H_{24}N_4O_4$ | 372.42 | 1.73 | glass film | 0.22 | 4.30 |
| **5**{*32*} | $C_{20}H_{26}N_4O_4$ | 386.44 | 2.04 | glass film | 0.30 | 4.90 |
| **5**{*33*} | $C_{20}H_{26}N_4O_4$ | 386.44 | 2.04 | glass film | 0.30 | 4.90 |
| **5**{*34*} | $C_{22}H_{35}N_5O_6$ | 465.54 | 1.31 | glass film | 0.13 | 4.72 |
| **5**{*35*} | $C_{25}H_{35}N_5O_6$ | 501.58 | 2.44 | glass film | 0.19 | 4.87 |
| **5**{*36*} | $C_{25}H_{28}N_4O_4$ | 448.51 | 3.08 | glass film | 0.32 | 6.10 |
| **5**{*37*} | $C_{20}H_{26}N_4O_4$ | 386.44 | 2.36 | glass film | 0.15 | 3.10 |
| **5**{*38*} | $C_{16}H_{26}N_4O_4$ | 338.40 | 1.23 | glass film | 0.24 | 1.95 |
| **5**{*39*} | $C_{21}H_{33}N_5O_6$ | 451.52 | 2.31 | glass film | 0.10 | 2.75 |
| **5**{*40*} | $C_{21}H_{26}N_4O_4$ | 398.46 | 2.65 | glass film | 0.15 | 3.50 |
| **5**{*41*} | $C_{18}H_{28}N_4O_4$ | 364.44 | 1.89 | solid | 0.12 | 2.60 |
| **5**{*42*} | $C_{22}H_{29}N_5O_4$ | 427.50 | 1.51 | glass film | 0.09 | 3.32 |
| **5**{*43*} | $C_{16}H_{18}N_4O_4$ | 330.34 | 0.24 | solid | 0.11 | 1.80 |
| **5**{*44*} | $C_{25}H_{35}N_5O_6$ | 501.58 | 1.87 | glass film | 0.14 | 4.60 |
| **5**{*45*} | $C_{22}H_{22}N_4O_4$ | 406.43 | 1.87 | glass film | 0.26 | 4.42 |
| **5**{*46*} | $C_{23}H_{24}N_4O_4$ | 420.46 | 2.18 | glass film | 0.25 | 4.95 |
| **5**{*47*} | $C_{23}H_{24}N_4O_4$ | 420.46 | 2.18 | glass film | 0.25 | 4.95 |
| **5**{*48*} | $C_{25}H_{33}N_5O_6$ | 499.56 | 1.44 | glass film | 0.14 | 4.82 |
| **5**{*49*} | $C_{28}H_{33}N_5O_6$ | 535.59 | 2.57 | glass film | 0.14 | 4.97 |
| **5**{*50*} | $C_{28}H_{26}N_4O_4$ | 482.53 | 3.21 | glass film | 0.30 | 6.05 |
| **5**{*51*} | $C_{23}H_{24}N_4O_4$ | 420.46 | 2.84 | glass film | 0.14 | 3.57 |
| **5**{*52*} | $C_{19}H_{24}N_4O_4$ | 372.42 | 1.71 | solid | 0.18 | 2.12 |
| **5**{*53*} | $C_{24}H_{31}N_5O_6$ | 485.53 | 2.79 | solid | 0.11 | 4.18 |
| **5**{*54*} | $C_{24}H_{24}N_4O_4$ | 432.47 | 3.12 | glass film | 0.18 | 3.92 |
| **5**{*55*} | $C_{21}H_{26}N_4O_4$ | 398.46 | 2.36 | solid | 0.12 | 2.95 |
| **5**{*56*} | $C_{25}H_{27}N_5O_4$ | 461.51 | 1.99 | glass film | 0.09 | 3.62 |

**Table S3.** Amino Acid Ester—Alkanamine Disubstituted I45DCs, **5**{*57-84*}.

| compound | formula | MW | C log*P* | form | $R_f$ | $R_t$ (min) |
|---|---|---|---|---|---|---|
| **5**{*57*} | $C_{16}H_{26}N_4O_4$ | 338.40 | 0.88 | glass film | 0.26 | 3.75 |
| **5**{*58*} | $C_{25}H_{43}N_5O_6$ | 509.64 | 2.85 | glass film | 0.23 | 6.22 |
| **5**{*59*} | $C_{22}H_{30}N_4O_4$ | 414.50 | 3.19 | glass film | 0.41 | 6.23 |
| **5**{*60*} | $C_{23}H_{32}N_4O_4$ | 428.52 | 3.50 | glass film | 0.42 | 6.73 |
| **5**{*61*} | $C_{23}H_{32}N_4O_4$ | 428.52 | 3.50 | glass film | 0.42 | 6.73 |
| **5**{*62*} | $C_{25}H_{41}N_5O_6$ | 507.62 | 2.77 | glass film | 0.29 | 6.55 |
| **5**{*63*} | $C_{28}H_{41}N_5O_6$ | 543.66 | 3.89 | glass film | 0.26 | 6.52 |
| **5**{*64*} | $C_{28}H_{34}N_4O_4$ | 490.59 | 4.54 | glass film | 0.52 | 7.67 |
| **5**{*65*} | $C_{23}H_{32}N_4O_4$ | 428.52 | 3.82 | solid | 0.24 | 5.55 |
| **5**{*66*} | $C_{19}H_{32}N_4O_4$ | 380.48 | 2.69 | solid | 0.33 | 4.45 |
| **5**{*67*} | $C_{24}H_{39}N_5O_6$ | 493.60 | 3.77 | solid | 0.21 | 5.27 |
| **5**{*68*} | $C_{24}H_{32}N_4O_4$ | 440.53 | 4.10 | solid | 0.28 | 5.73 |
| **5**{*69*} | $C_{21}H_{34}N_4O_4$ | 406.52 | 3.34 | solid | 0.25 | 5.20 |
| **5**{*70*} | $C_{25}H_{35}N_5O_4$ | 469.58 | 2.97 | solid | 0.19 | 5.60 |
| **5**{*71*} | $C_{19}H_{24}N_4O_4$ | 372.42 | 1.70 | glass film | 0.16 | 4.80 |
| **5**{*72*} | $C_{28}H_{41}N_5O_6$ | 543.66 | 3.33 | glass film | 0.16 | 6.20 |
| **5**{*73*} | $C_{25}H_{28}N_4O_4$ | 448.51 | 3.32 | glass film | 0.30 | 6.90 |
| **5**{*74*} | $C_{26}H_{30}N_4O_4$ | 462.54 | 3.63 | glass film | 0.47 | 6.75 |
| **5**{*75*} | $C_{26}H_{30}N_4O_4$ | 462.54 | 3.63 | glass film | 0.47 | 6.75 |
| **5**{*76*} | $C_{28}H_{39}N_5O_6$ | 541.64 | 2.90 | glass film | 0.27 | 6.47 |
| **5**{*77*} | $C_{31}H_{39}N_5O_6$ | 577.67 | 4.02 | solid | 0.24 | 6.40 |
| **5**{*78*} | $C_{31}H_{32}N_4O_4$ | 524.61 | 4.67 | glass film | 0.52 | 7.45 |
| **5**{*79*} | $C_{26}H_{30}N_4O_4$ | 462.54 | 4.29 | glass film | 0.22 | 5.75 |
| **5**{*80*} | $C_{22}H_{30}N_4O_4$ | 414.50 | 3.16 | glass film | 0.29 | 4.85 |
| **5**{*81*} | $C_{27}H_{37}N_5O_6$ | 527.61 | 4.24 | solid | 0.13 | 5.45 |
| **5**{*82*} | $C_{27}H_{30}N_4O_4$ | 474.55 | 4.58 | glass film | 0.23 | 5.85 |
| **5**{*83*} | $C_{24}H_{32}N_4O_4$ | 440.54 | 3.82 | solid | 0.15 | 5.45 |
| **5**{*84*} | $C_{28}H_{33}N_5O_4$ | 503.59 | 3.44 | glass film | 0.12 | 5.75 |

**Table S4.** Amino Acid Ester—Alkanamine Disubstituted I45DCs, **5**{*85-112*}.

| compound | formula | MW | C log*P* | form | $R_f$ | $R_t$ (min) |
|---|---|---|---|---|---|---|
| **5**{*85*} | $C_{19}H_{24}N_4O_4$ | 372.42 | 1.18 | glass film | 0.26 | 3.80 |
| **5**{*86*} | $C_{28}H_{41}N_5O_6$ | 543.66 | 3.15 | glass film | 0.29 | 6.10 |
| **5**{*87*} | $C_{25}H_{28}N_4O_4$ | 448.51 | 2.81 | glass film | 0.41 | 6.08 |
| **5**{*88*} | $C_{26}H_{30}N_4O_4$ | 462.54 | 3.12 | glass film | 0.36 | 6.65 |
| **5**{*89*} | $C_{26}H_{30}N_4O_4$ | 462.54 | 3.12 | glass film | 0.36 | 6.65 |
| **5**{*90*} | $C_{28}H_{39}N_5O_6$ | 541.64 | 2.39 | glass film | 0.26 | 6.40 |
| **5**{*91*} | $C_{31}H_{39}N_5O_6$ | 577.67 | 3.51 | glass film | 0.31 | 6.45 |
| **5**{*92*} | $C_{31}H_{32}N_4O_4$ | 524.61 | 4.16 | glass film | 0.48 | 7.50 |
| **5**{*93*} | $C_{26}H_{30}N_4O_4$ | 462.54 | 3.78 | glass film | 0.31 | 5.35 |
| **5**{*94*} | $C_{22}H_{30}N_4O_4$ | 414.50 | 2.65 | glass film | 0.36 | 4.30 |
| **5**{*95*} | $C_{27}H_{37}N_5O_6$ | 527.61 | 3.73 | solid | 0.21 | 5.12 |
| **5**{*96*} | $C_{27}H_{30}N_4O_4$ | 474.55 | 4.06 | glass film | 0.26 | 5.80 |
| **5**{*97*} | $C_{24}H_{30}N_4O_4$ | 462.54 | 3.12 | glass film | 0.36 | 5.09 |
| **5**{*98*} | $C_{28}H_{32}N_5O_4$ | 440.54 | 3.30 | glass film | 0.24 | 5.45 |
| **5**{*99*} | $C_{22}H_{22}N_4O_4$ | 406.43 | 1.66 | solid | 0.16 | 4.05 |
| **5**{*100*} | $C_{31}H_{39}N_5O_6$ | 577.67 | 3.63 | glass film | 0.19 | 6.12 |
| **5**{*101*} | $C_{28}H_{26}N_4O_4$ | 482.53 | 3.28 | glass film | 0.29 | 6.25 |
| **5**{*102*} | $C_{29}H_{28}N_4O_4$ | 496.56 | 3.59 | glass film | 0.29 | 6.75 |
| **5**{*103*} | $C_{29}H_{28}N_4O_4$ | 496.56 | 3.59 | glass film | 0.29 | 6.75 |
| **5**{*104*} | $C_{31}H_{37}N_5O_6$ | 575.66 | 2.86 | glass film | 0.18 | 6.35 |
| **5**{*105*} | $C_{34}H_{37}N_5O_6$ | 611.69 | 3.99 | glass film | 0.22 | 6.28 |
| **5**{*106*} | $C_{34}H_{30}N_4O_4$ | 558.63 | 4.63 | glass film | 0.35 | 7.25 |
| **5**{*107*} | $C_{29}H_{28}N_4O_4$ | 496.56 | 4.25 | glass film | 0.19 | 5.60 |
| **5**{*108*} | $C_{25}H_{28}N_4O_4$ | 448.51 | 3.12 | glass film | 0.19 | 5.30 |
| **5**{*109*} | $C_{30}H_{35}N_5O_6$ | 561.63 | 4.20 | glass film | 0.09 | 5.30 |
| **5**{*110*} | $C_{30}H_{28}N_4O_4$ | 508.57 | 4.54 | glass film | 0.22 | 5.73 |
| **5**{*111*} | $C_{27}H_{30}N_4O_4$ | 474.55 | 3.78 | glass film | 0.15 | 5.42 |
| **5**{*112*} | $C_{31}H_{31}N_5O_4$ | 537.61 | 3.40 | glass film | 0.12 | 5.75 |

**Table S5.** Amino Acid Ester—Alkanamine Disubstituted I45DCs, **5**{*113-126*}.

| compound | formula | MW | C log*P* | form | $R_f$ | $R_t$ (min) |
|---|---|---|---|---|---|---|
| **5**{*113*} | $C_{21}H_{35}N_5O_6$ | 453.53 | 0.87 | glass film | 0.16 | 3.25 |
| **5**{*114*} | $C_{30}H_{52}N_6O_8$ | 624.77 | 2.83 | glass film | 0.19 | 5.70 |
| **5**{*115*} | $C_{27}H_{39}N_5O_6$ | 529.63 | 3.18 | glass film | 0.28 | 5.65 |
| **5**{*116*} | $C_{28}H_{41}N_5O_6$ | 543.66 | 3.14 | glass film | 0.28 | 6.05 |
| **5**{*117*} | $C_{28}H_{41}N_5O_6$ | 543.66 | 3.14 | glass film | 0.28 | 6.05 |
| **5**{*118*} | $C_{30}H_{50}N_6O_8$ | 622.75 | 2.75 | glass film | 0.19 | 5.90 |
| **5**{*119*} | $C_{33}H_{50}N_6O_8$ | 658.79 | 3.88 | glass film | 0.12 | 5.90 |
| **5**{*120*} | $C_{33}H_{43}N_5O_6$ | 605.72 | 4.52 | glass film | 0.34 | 6.95 |
| **5**{*121*} | $C_{28}H_{41}N_5O_6$ | 543.66 | 3.80 | glass film | 0.12 | 5.10 |
| **5**{*122*} | $C_{24}H_{41}N_5O_6$ | 495.61 | 2.67 | glass film | 0.20 | 4.15 |
| **5**{*123*} | $C_{29}H_{48}N_6O_8$ | 608.73 | 3.75 | glass film | 0.15 | 4.97 |
| **5**{*124*} | $C_{29}H_{41}N_5O_6$ | 555.67 | 4.09 | glass film | 0.20 | 5.30 |
| **5**{*125*} | $C_{26}H_{43}N_5O_6$ | 521.65 | 3.33 | glass film | 0.15 | 4.85 |
| **5**{*126*} | $C_{30}H_{44}N_6O_6$ | 584.71 | 2.96 | glass film | 0.12 | 5.25 |

**Note on Morphology of Samples.** The purified products were transferred as $CH_2Cl_2$ solutions into vials for storage and allowed to evaporate at room temperature. It is noteworthy that a majority of the samples yield a transparent glass film (78%) from this process, with a smaller percentage of samples yielding largely amorphous white solids (22%). Primary amines yielded 8 solids from 72 samples (11%), while secondary amines yielded 20 solids from 54 samples (37%). This difference is likely due to a specific intramolecular hydrogen bonded conformation that forms in library members substituted with a secondary alkanamine as compared with the equilibrium of two intramolecular hydrogen bonded conformations for library members substituted with primary alkanamines. The library members are all readily soluble in $CH_2Cl_2$ and this volatile solvent is a good choice for the transfer of samples; however, we also know from our past research with I45DCs that this is a poor crystallization solvent and better results are obtained from either 2-propanone or methanol (Baures PW, Rush JR, Wiznycia AV, Desper J, Helfrich BA, Beatty AM: Intramolecular hydrogen bonding and intermolecular dimerization in the crystal structures of imidazole-4,5-dicarboxylic acid derivatives. *Cryst. Growth Des.* 2002, **2**:653-664). Thus, the observed percentages of solids versus transparent glass films, as well as percentages of amorphous solids versus crystalline solids, could very well change with a complete investigation of solvent choices.

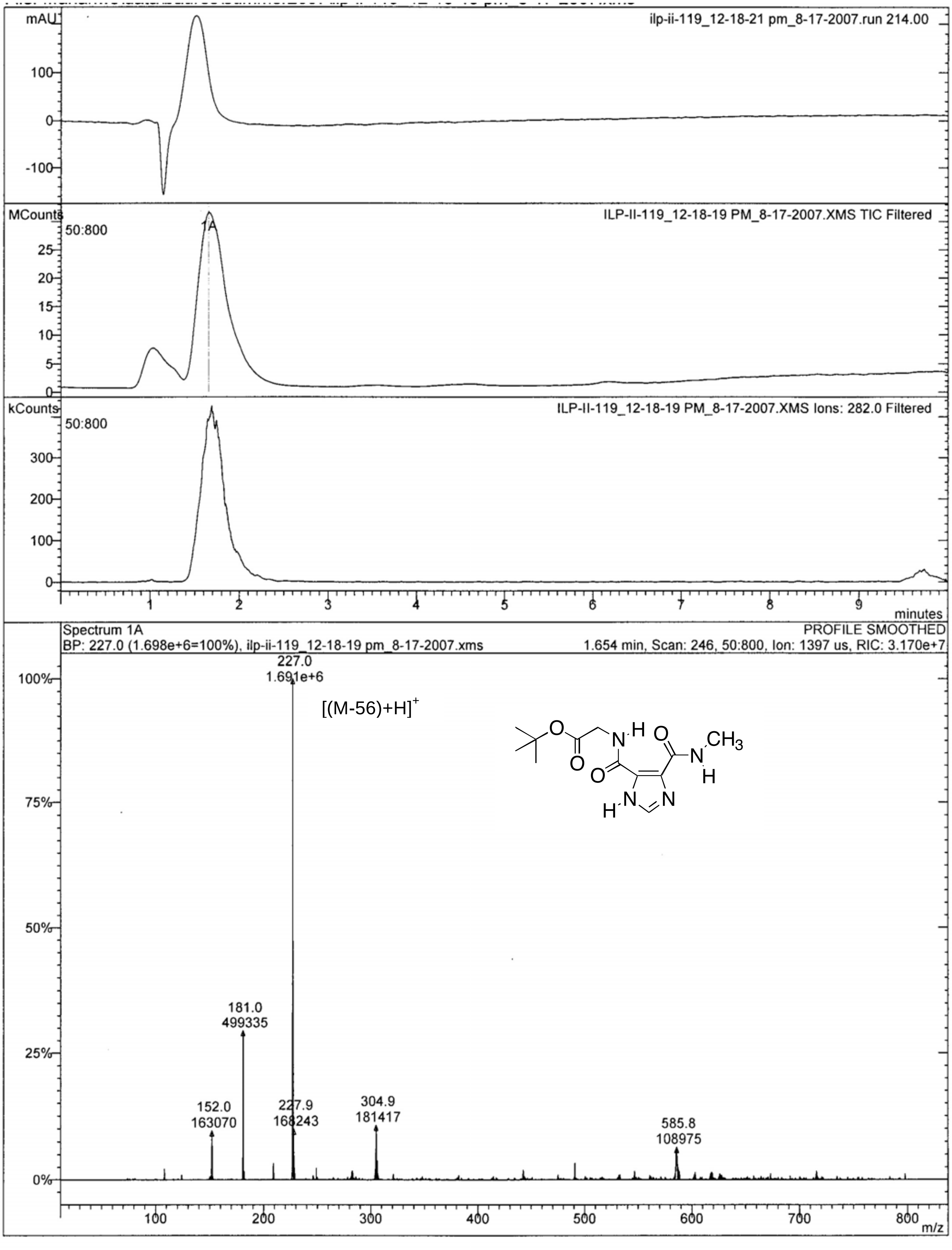


**Figure S1.** LC/MS data for **5{**1}.

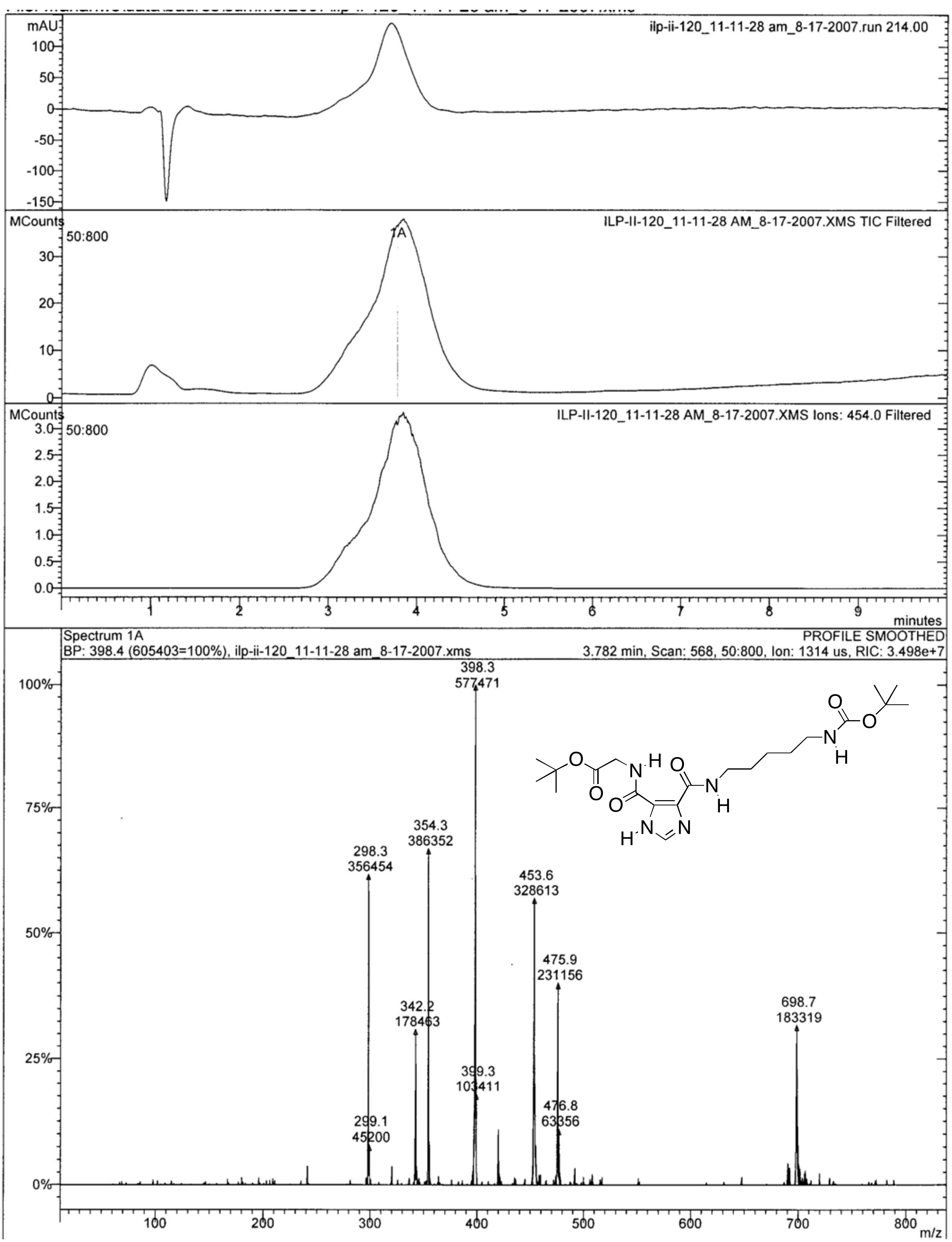


**Figure S2.** LC/MS data for **5{**2}.

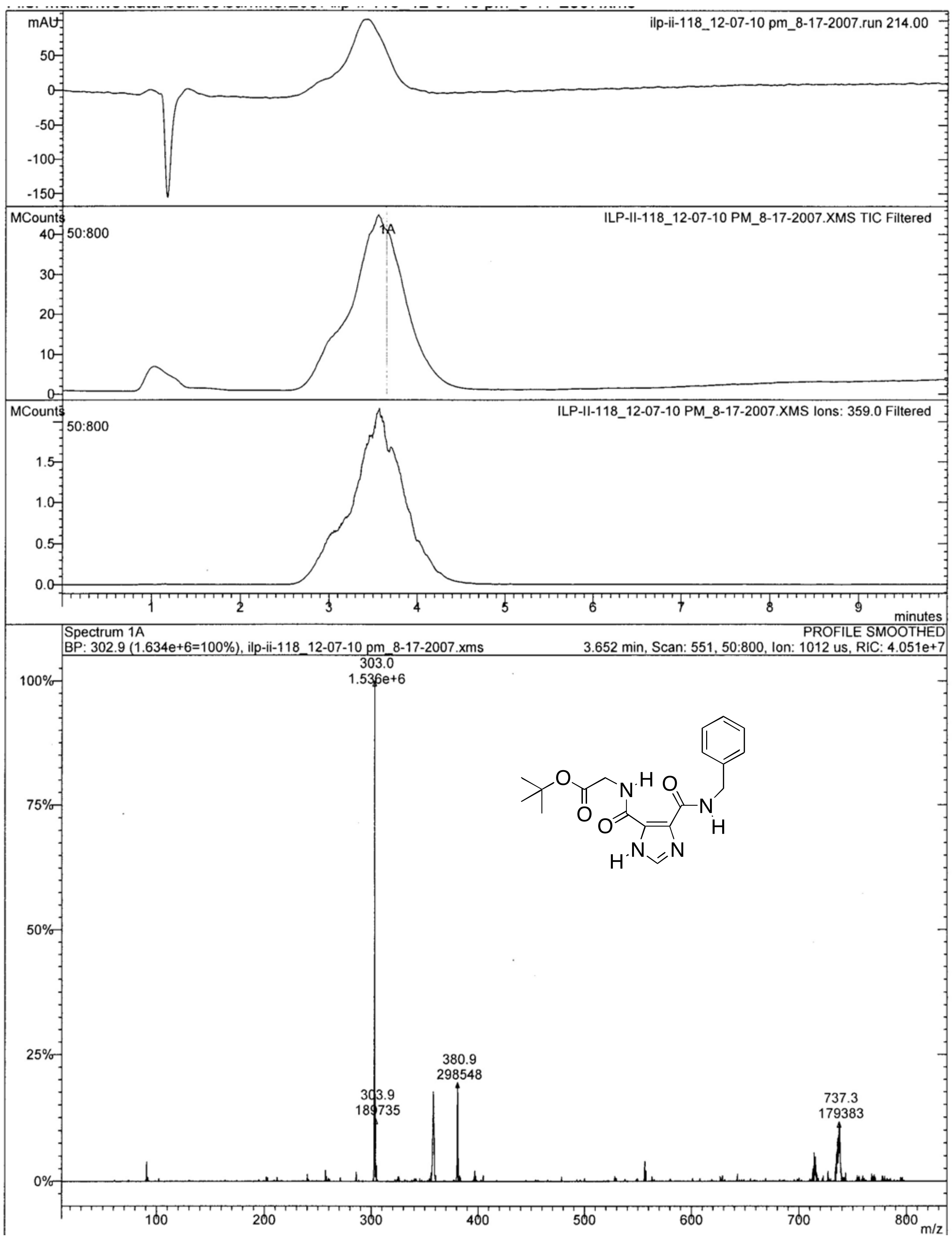


**Figure S3.** LC/MS data for **5{**3}.

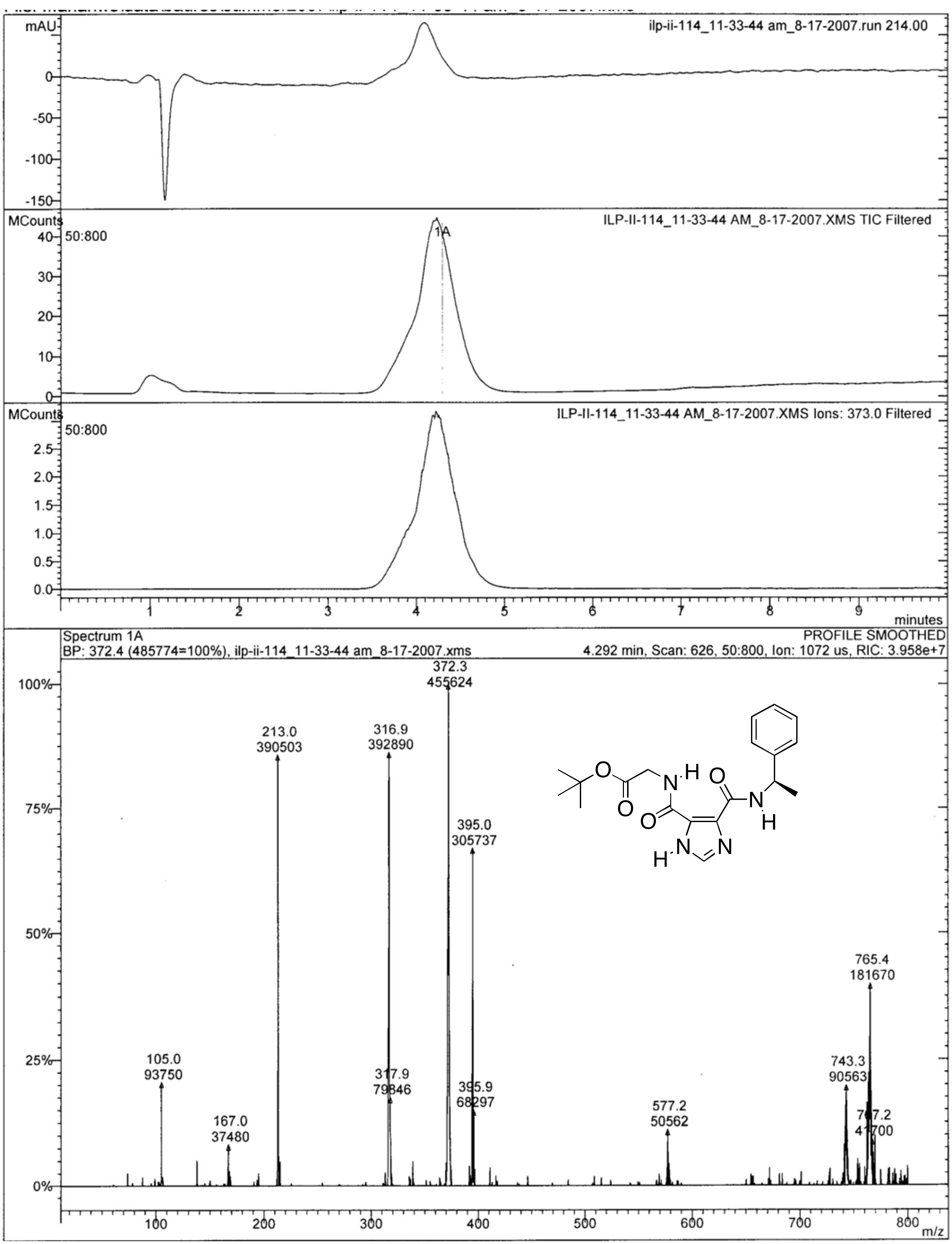


**Figure S4.** LC/MS data for **5{**4}.

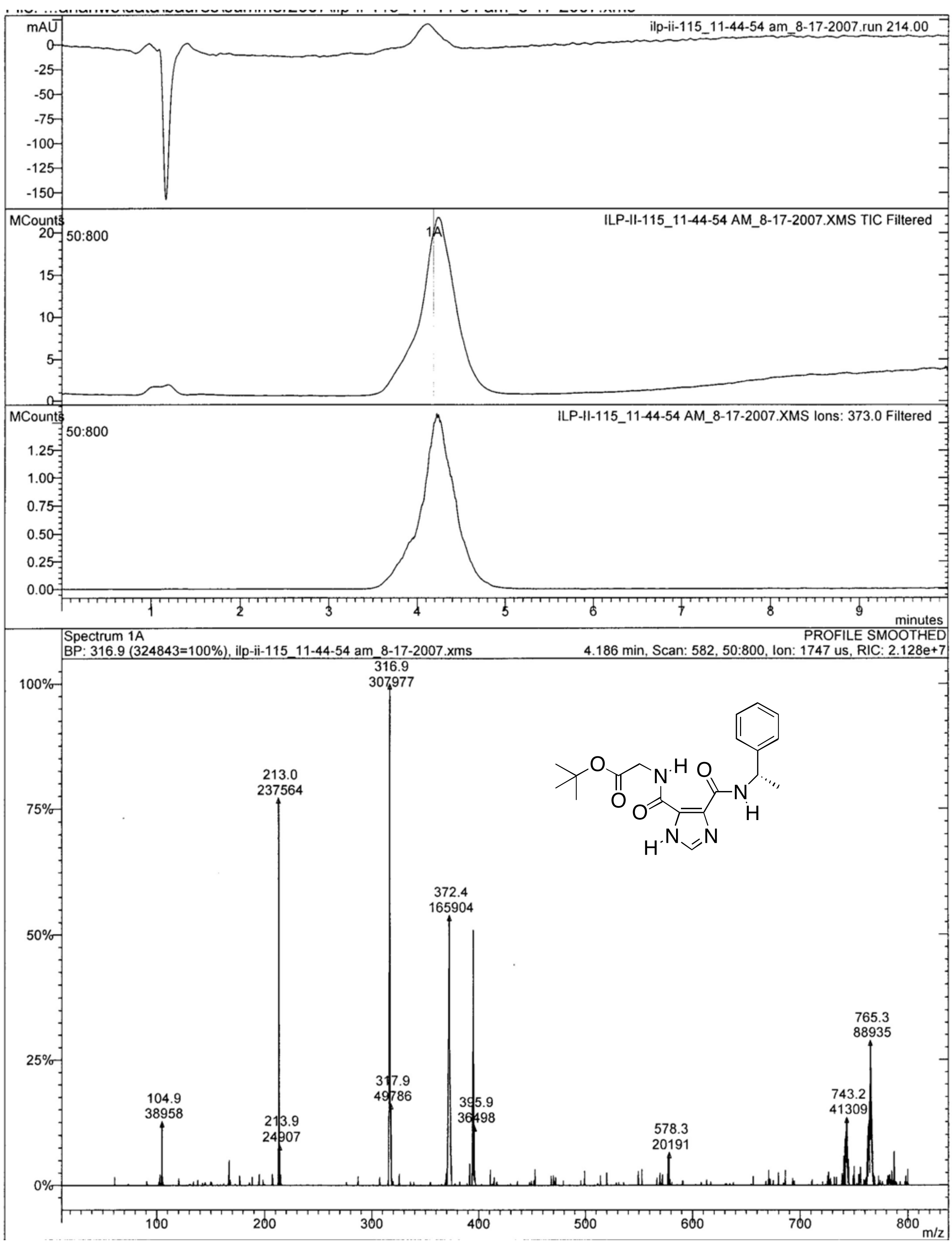


**Figure S5.** LC/MS data for **5{**5}.

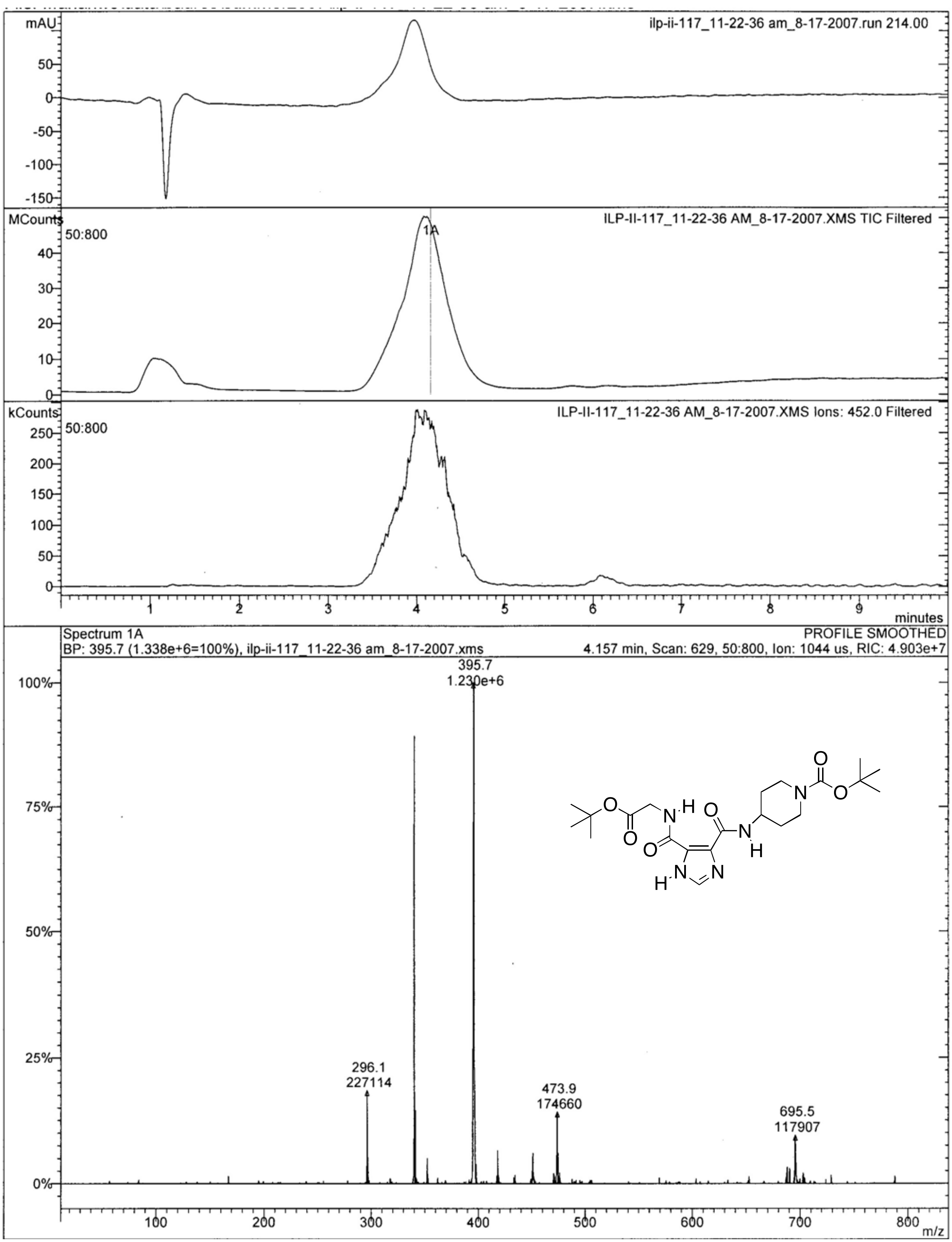


**Figure S6.** LC/MS data for **5{**6}.

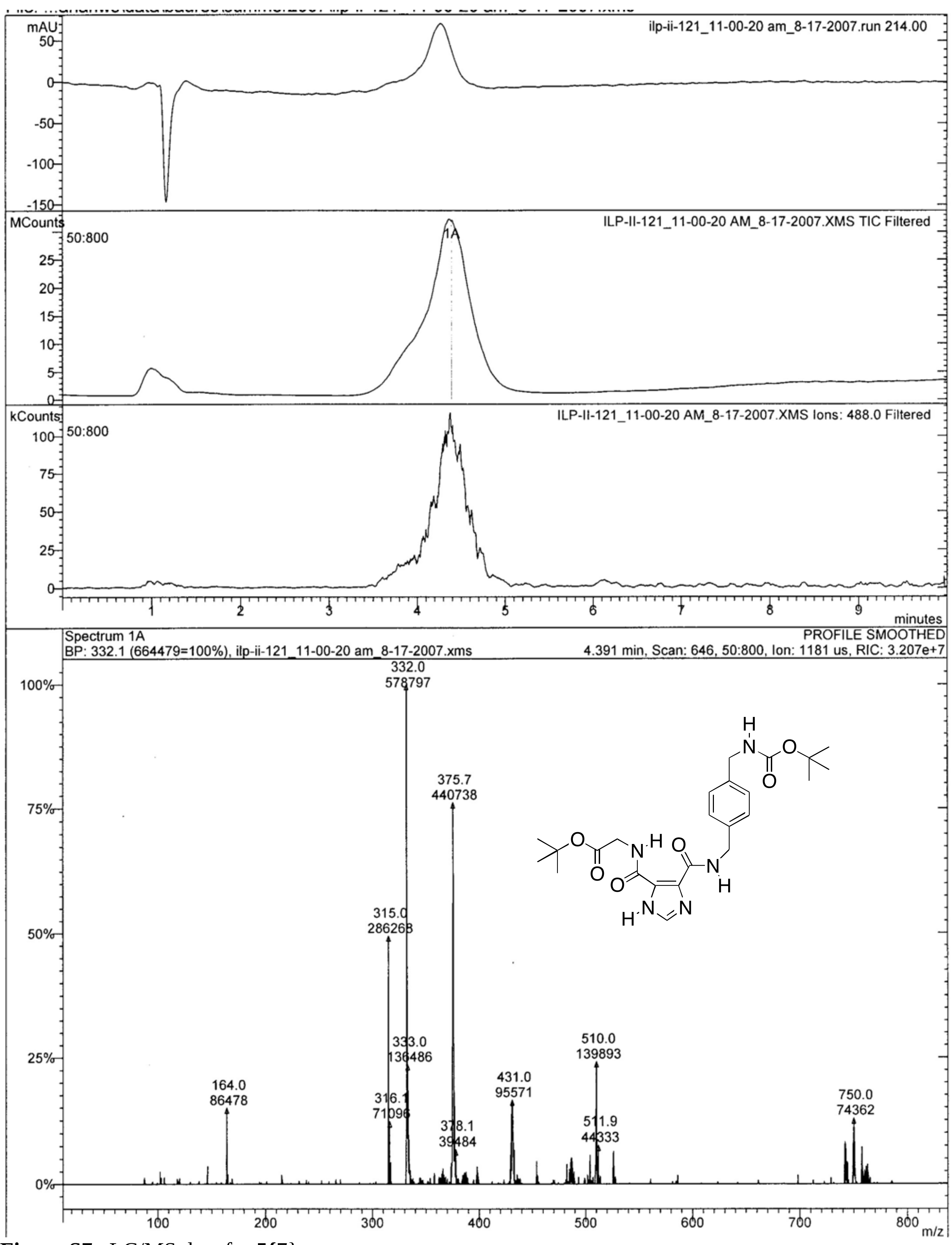


**Figure S7.** LC/MS data for **5{**7}.

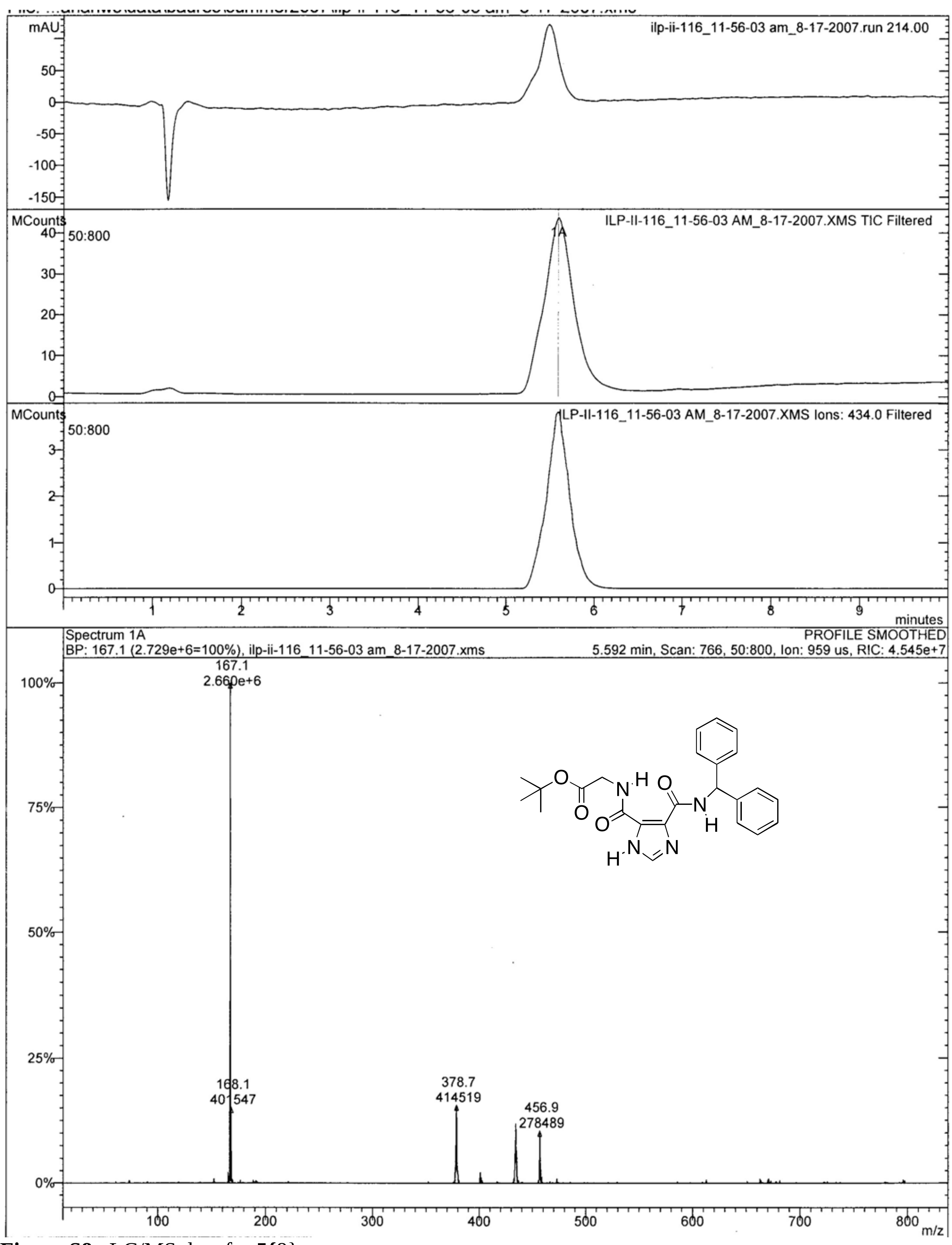


**Figure S8.** LC/MS data for **5{**8}.

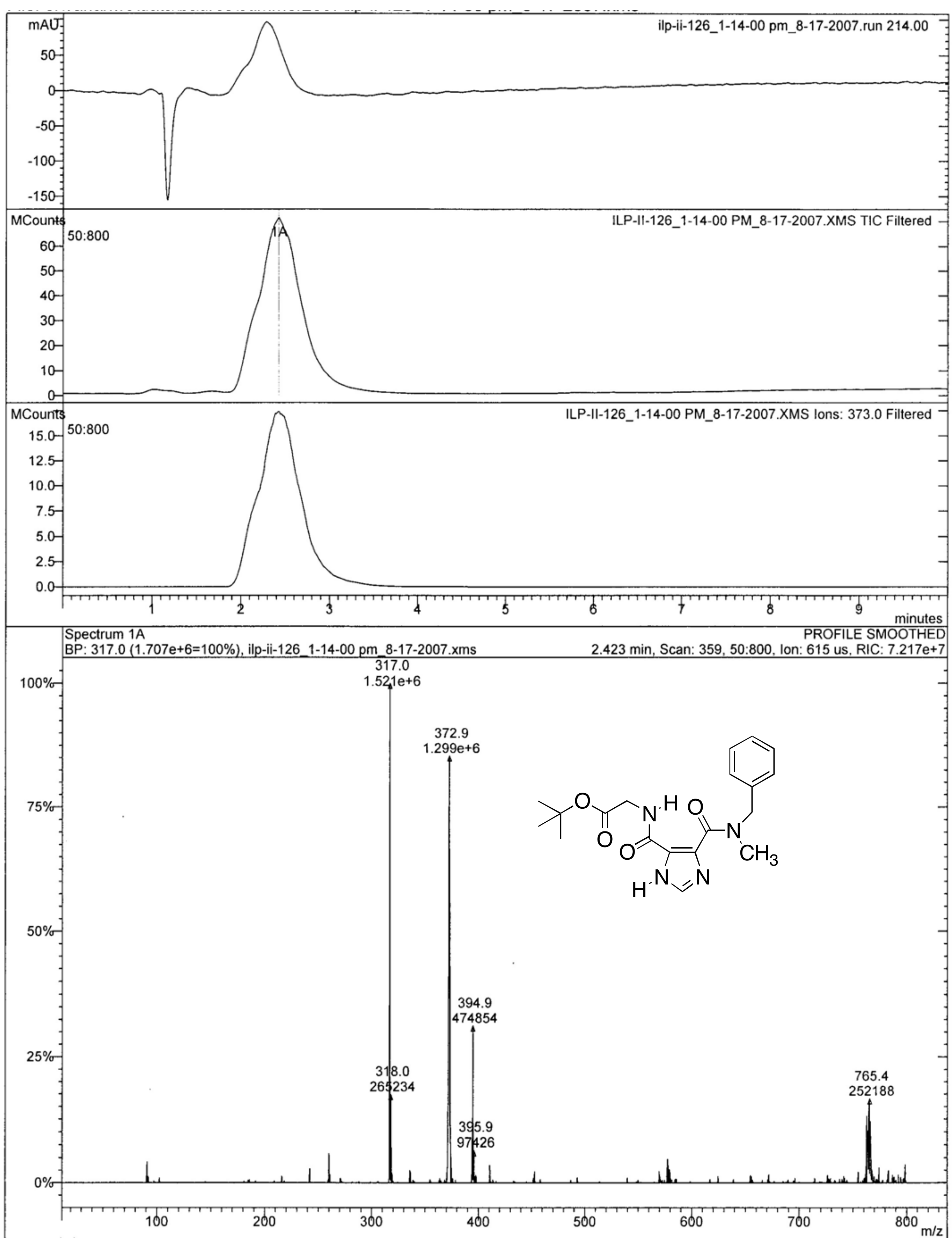


**Figure S9.** LC/MS data for **5{**9}.

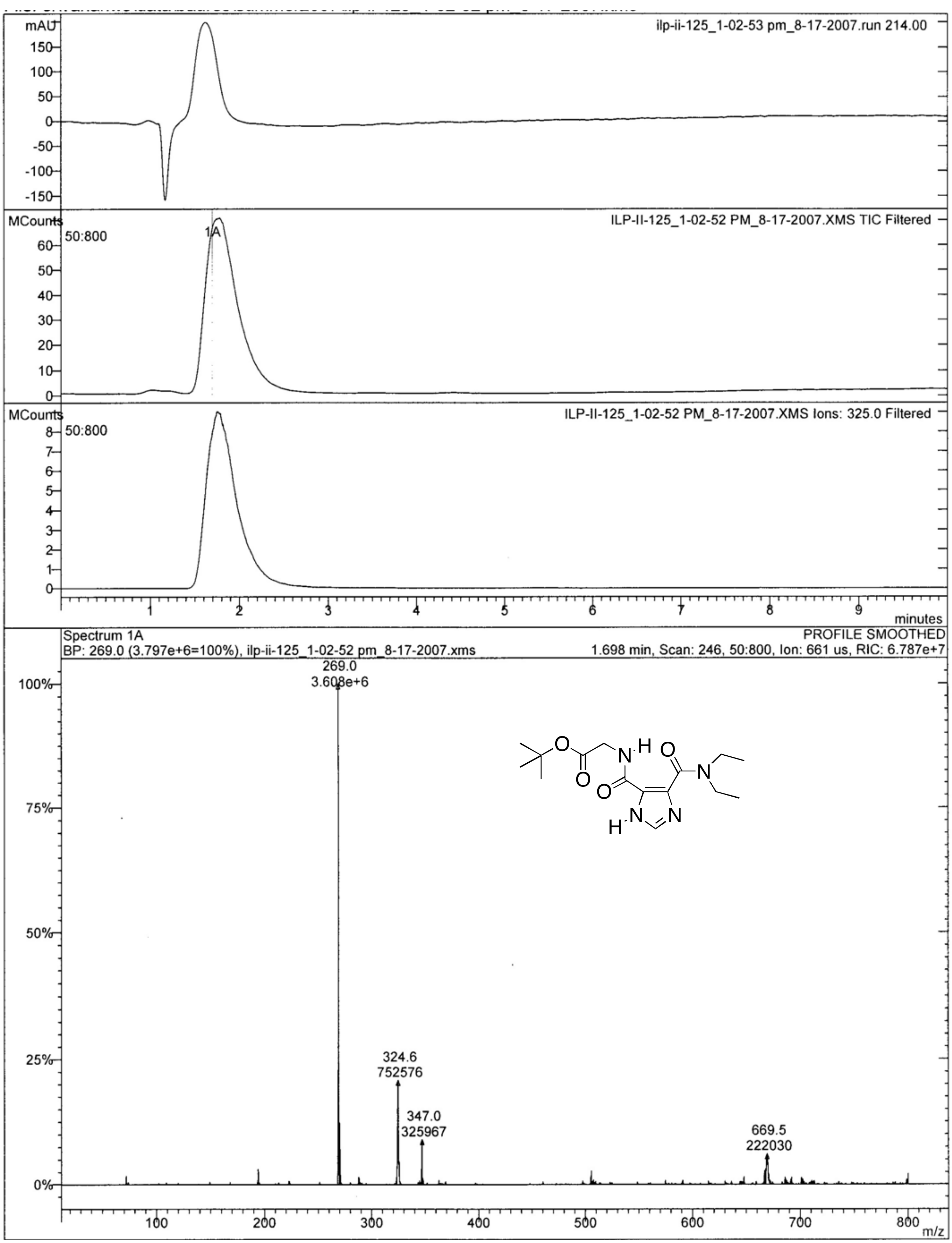


**Figure S10.** LC/MS data for **5{**10}.

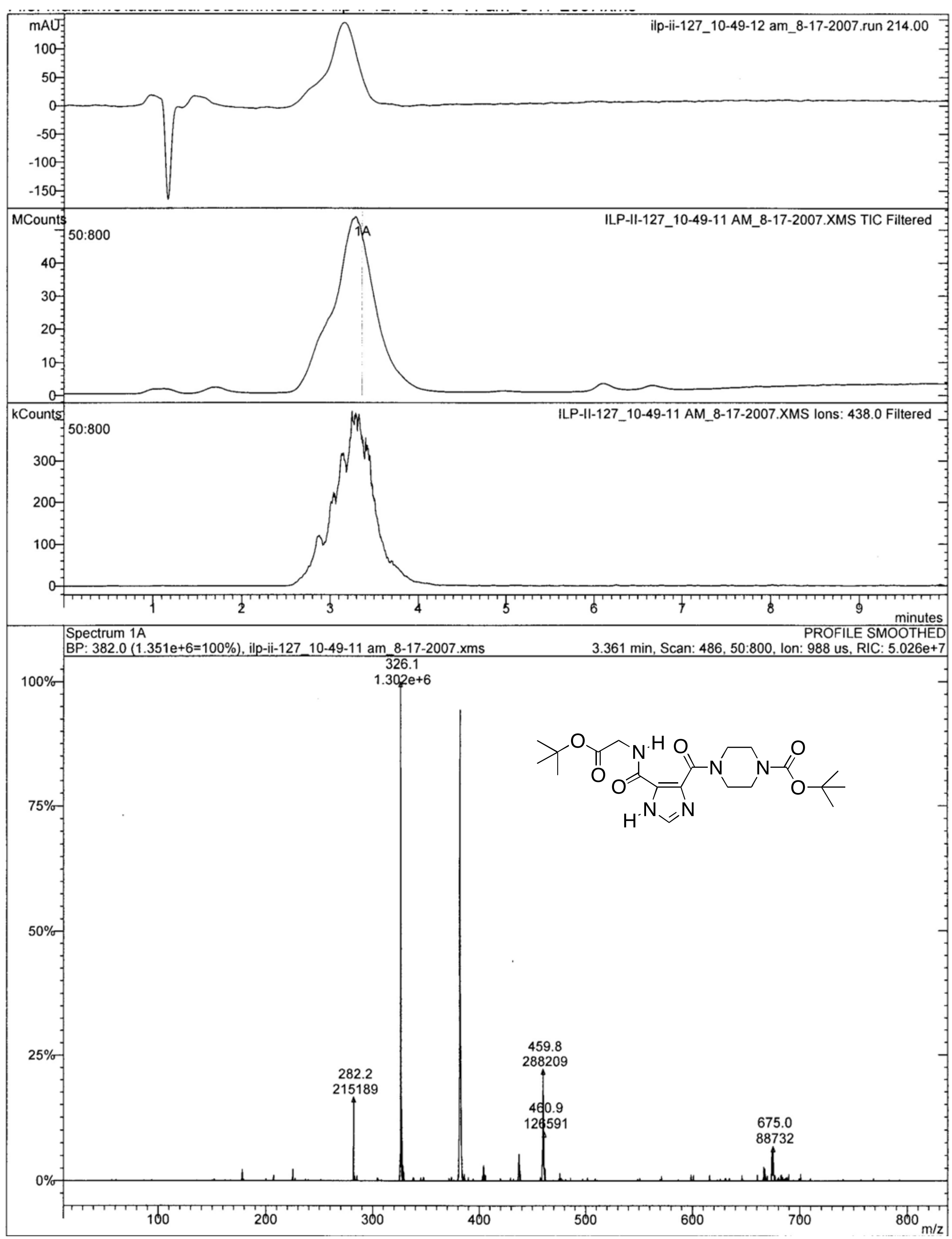


**Figure S11.** LC/MS data for **5{**11}.

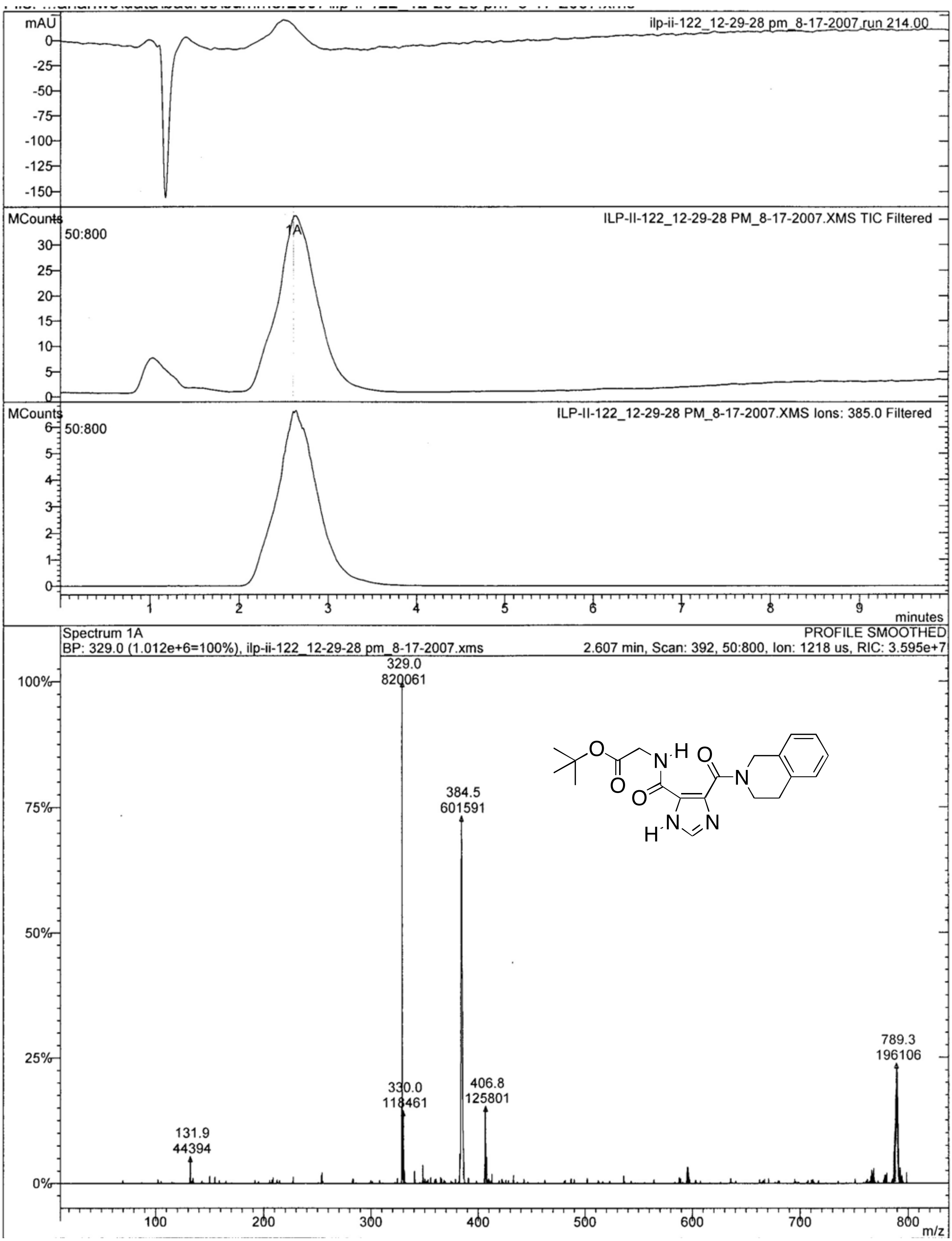


**Figure S12.** LC/MS data for **5{**12}.

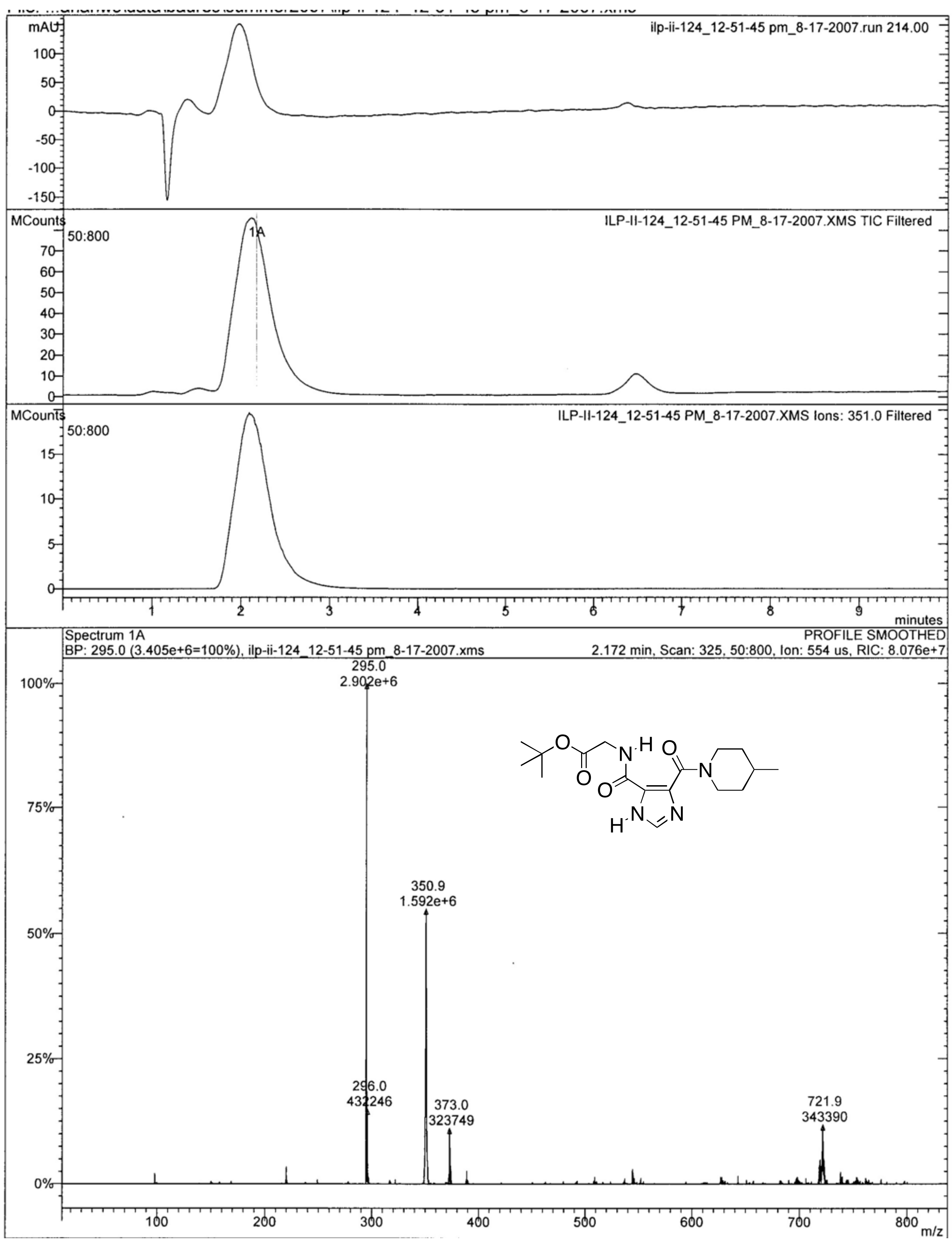


**Figure S13.** LC/MS data for **5{**13}.

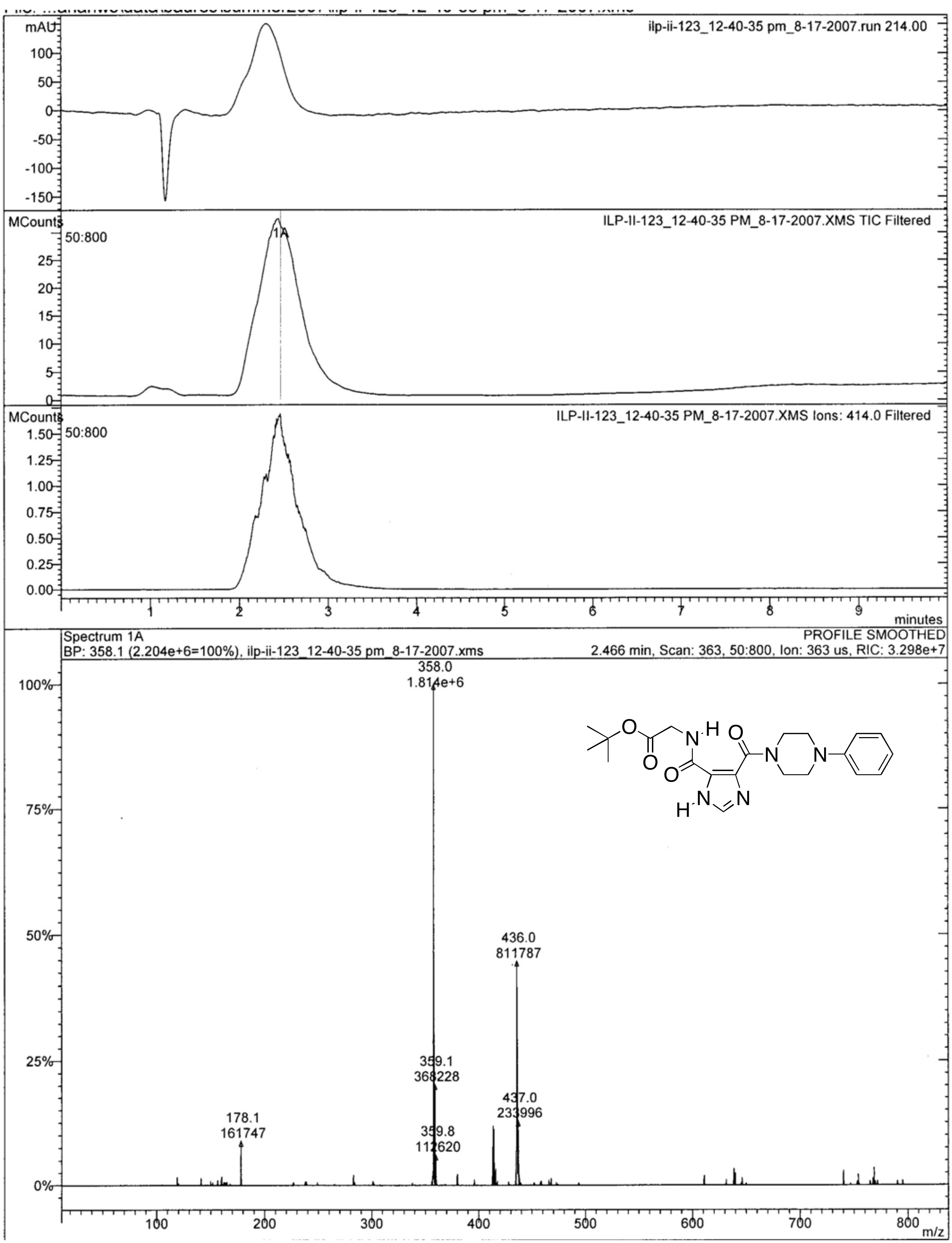


**Figure S14.** LC/MS data for **5{**14}.

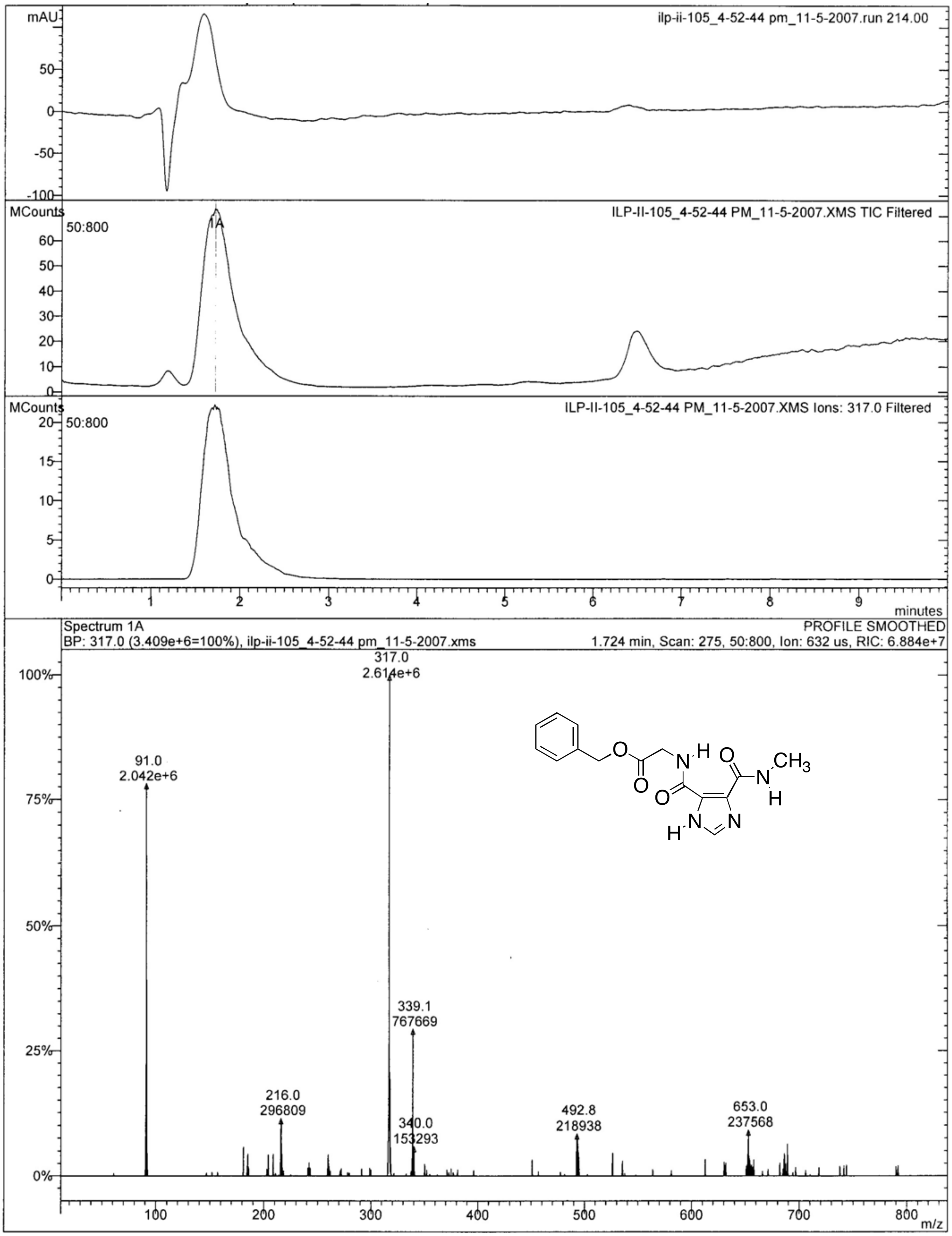


**Figure S15.** LC/MS data for **5{**15}.

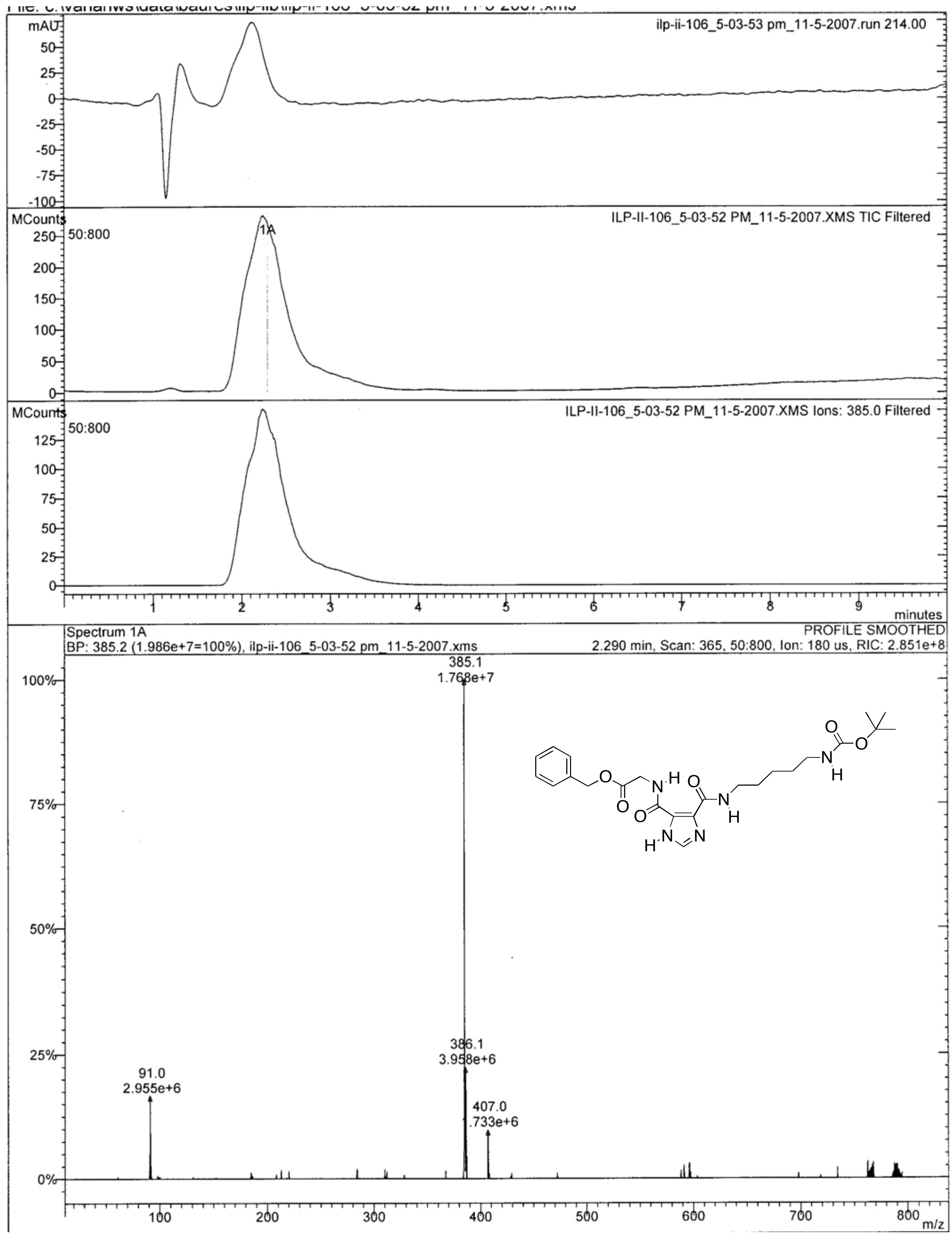


**Figure S16.** LC/MS data for **5{**16}.

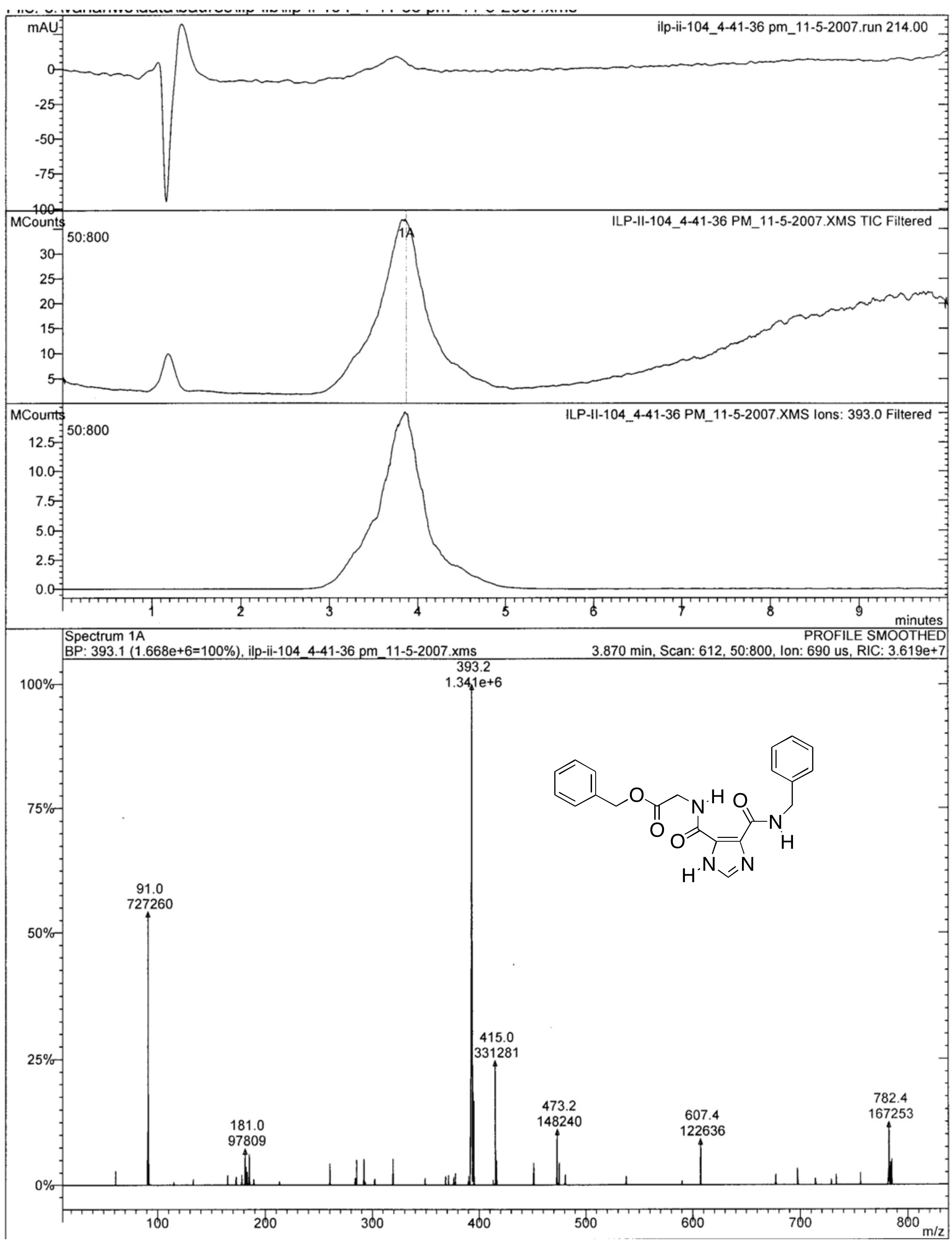


**Figure S17.** LC/MS data for **5{**17}.

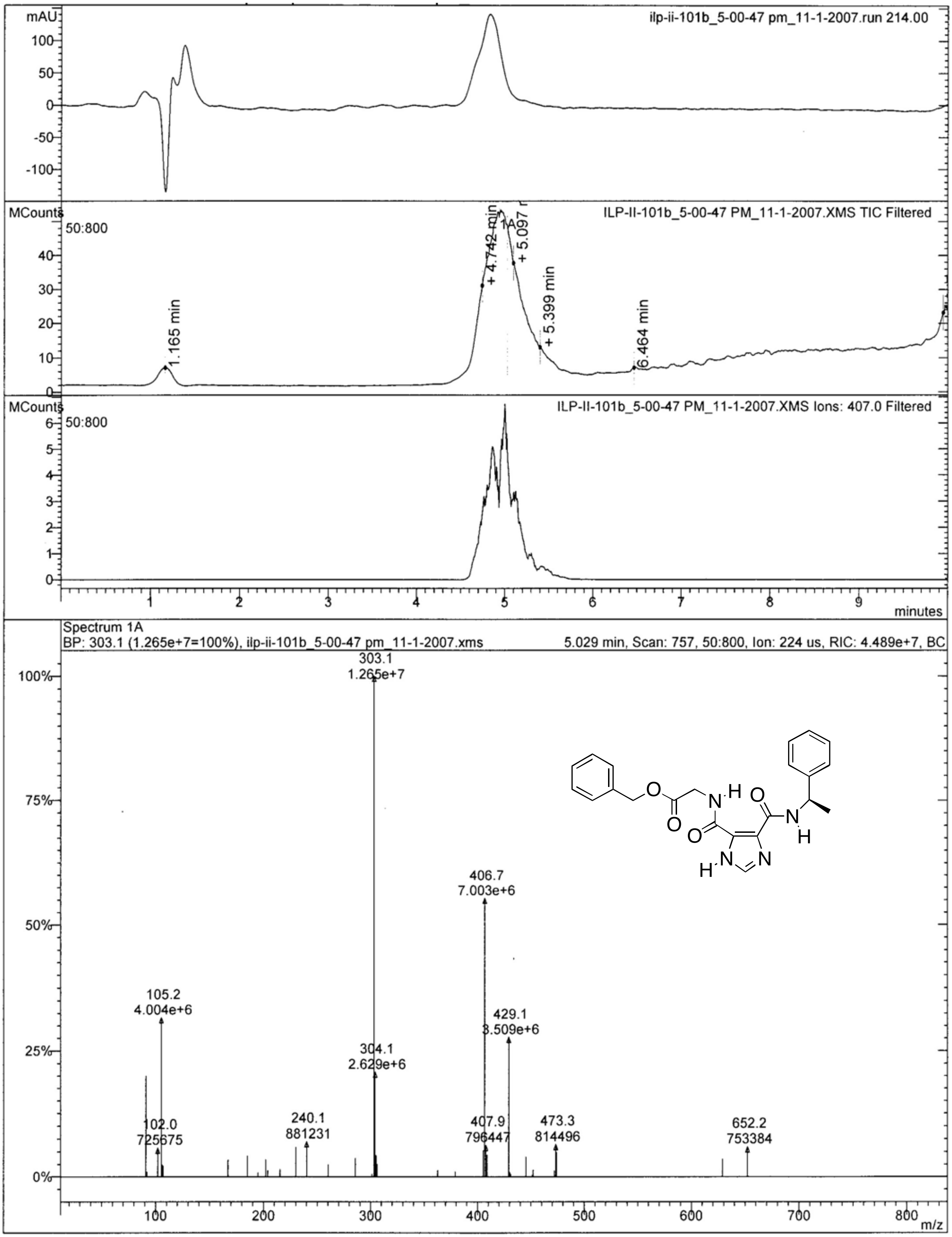


**Figure S18.** LC/MS data for **5{**18}.

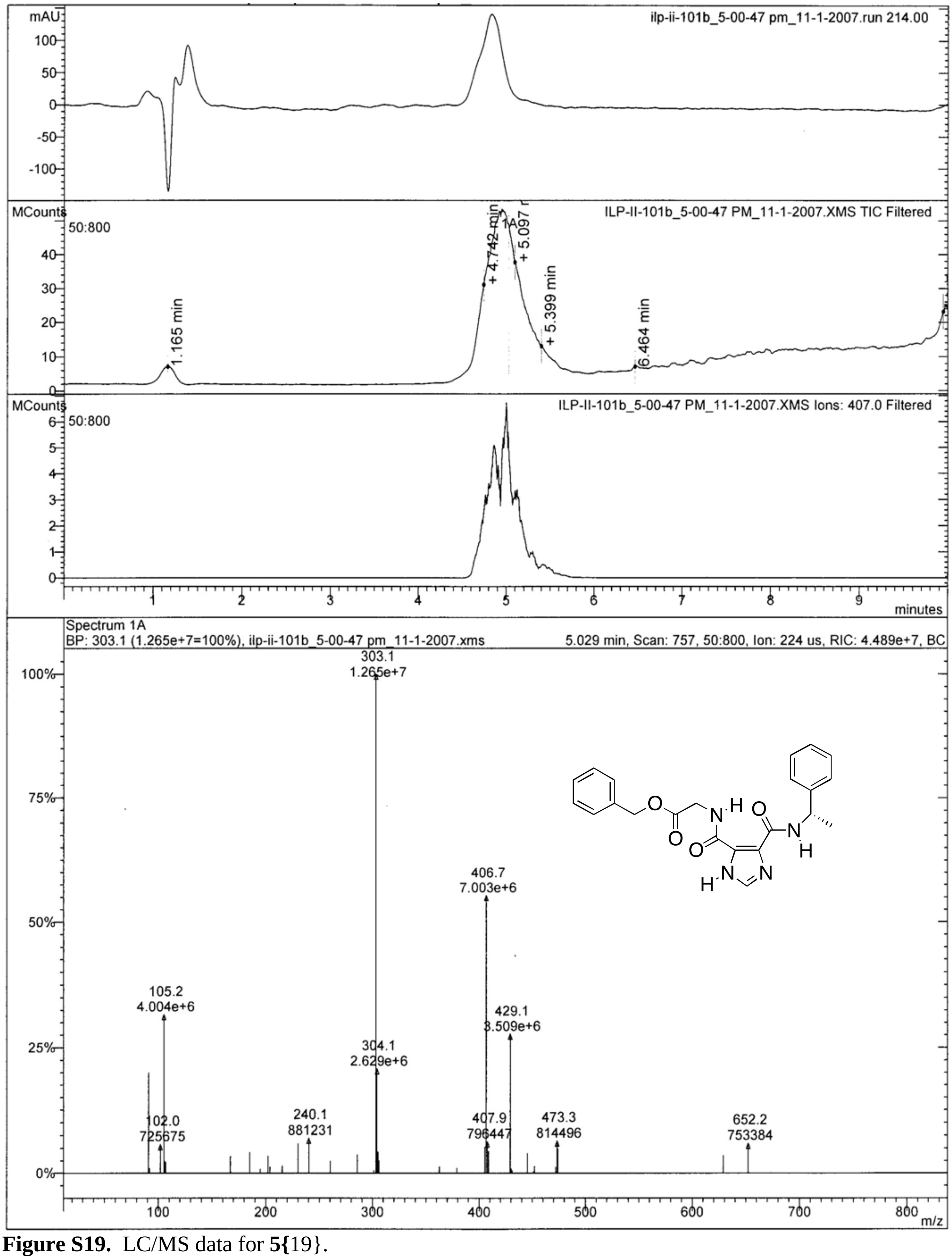


**Figure S19.** LC/MS data for **5{**19}.

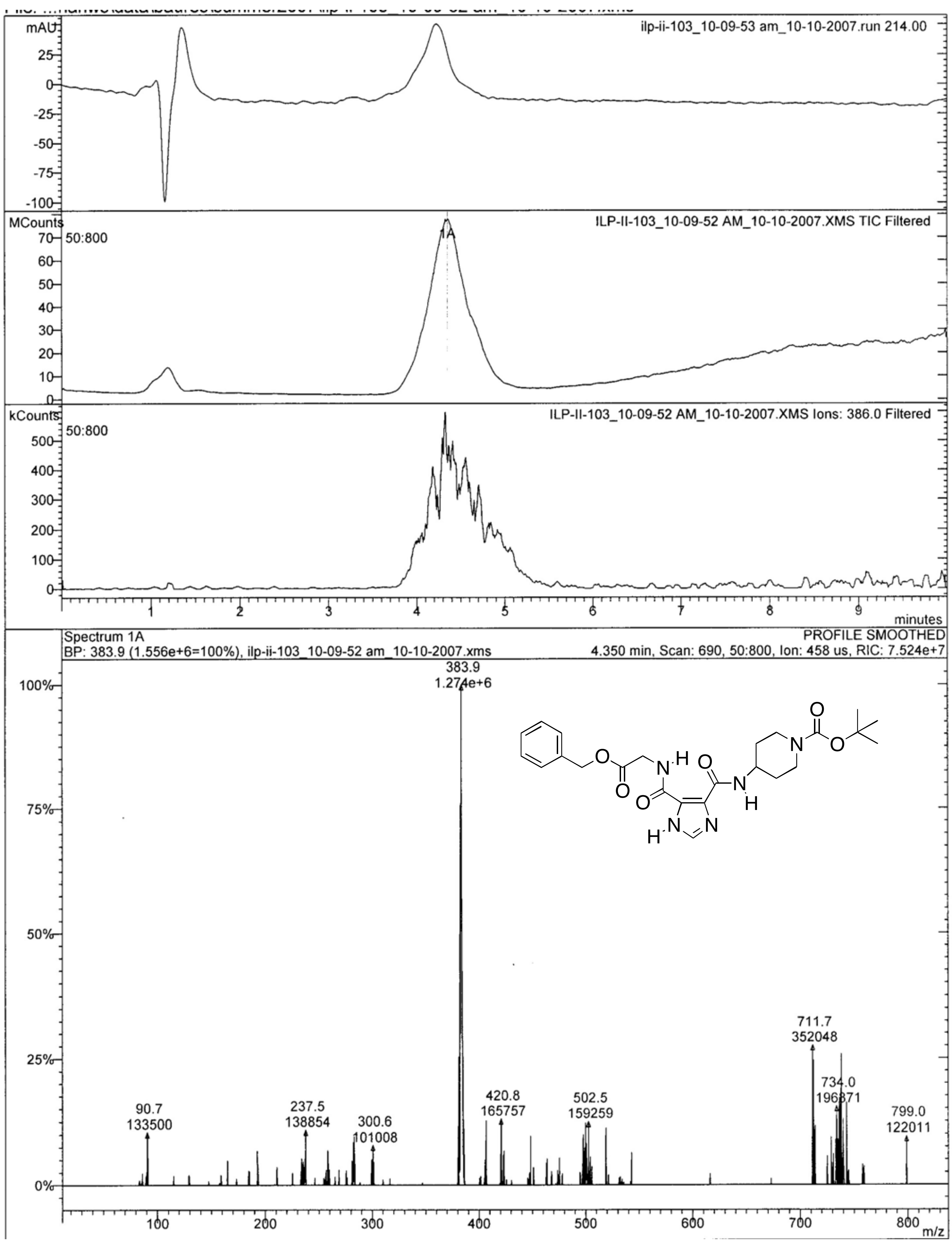


**Figure S20.** LC/MS data for **5{**20}.

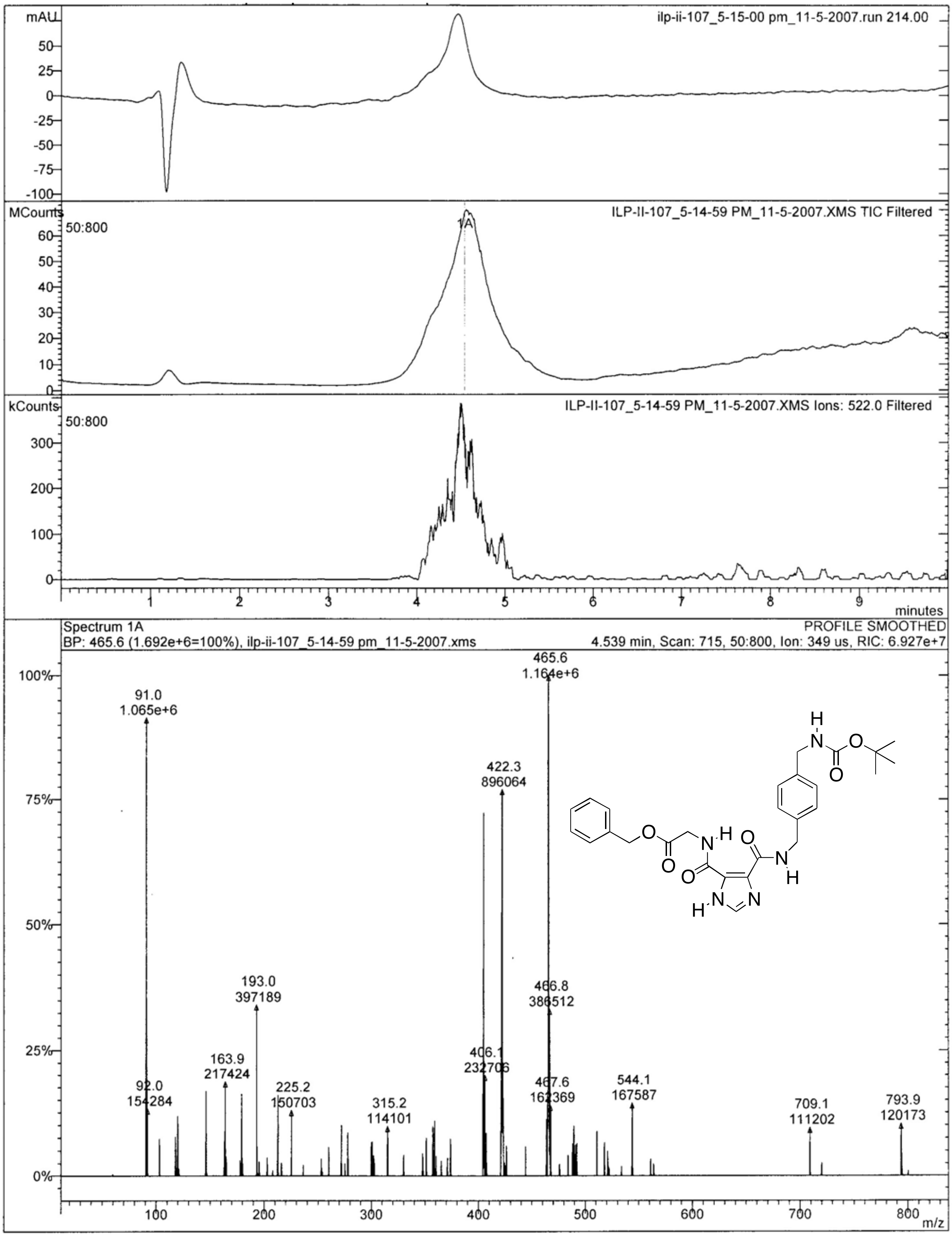


**Figure S21.** LC/MS data for **5{**21}.

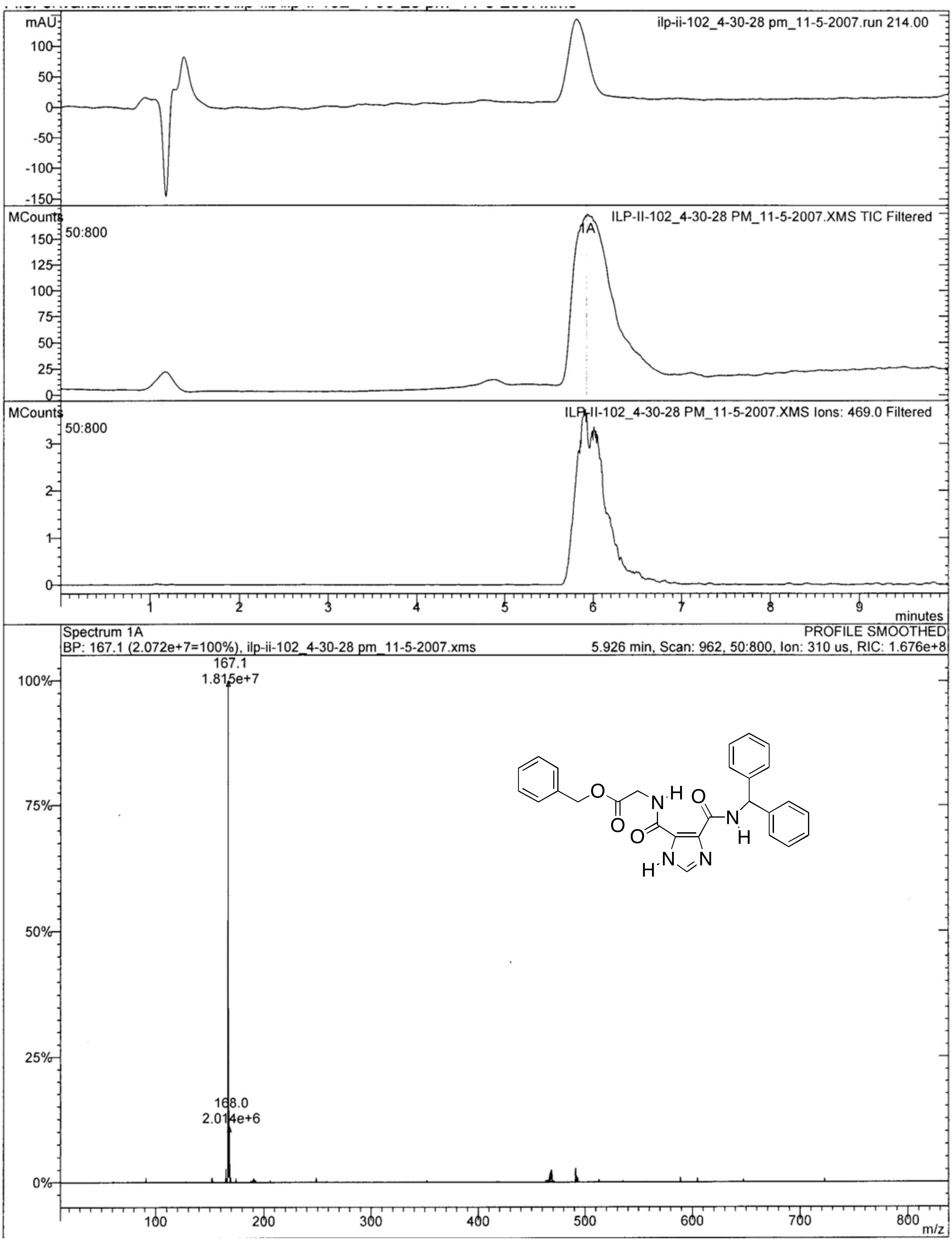


**Figure S22.** LC/MS data for **5{**22}.

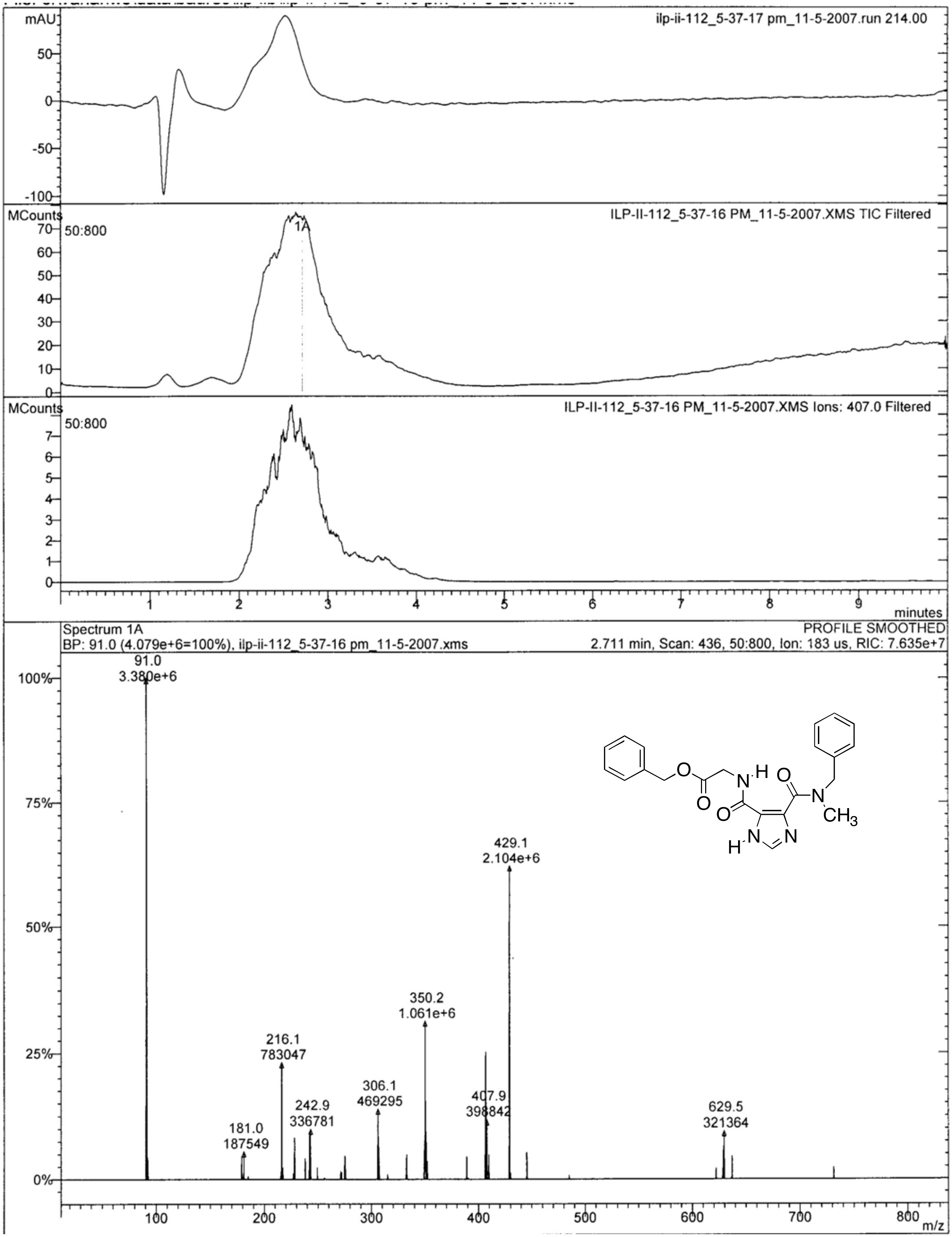


**Figure S23.** LC/MS data for **5{**23}.

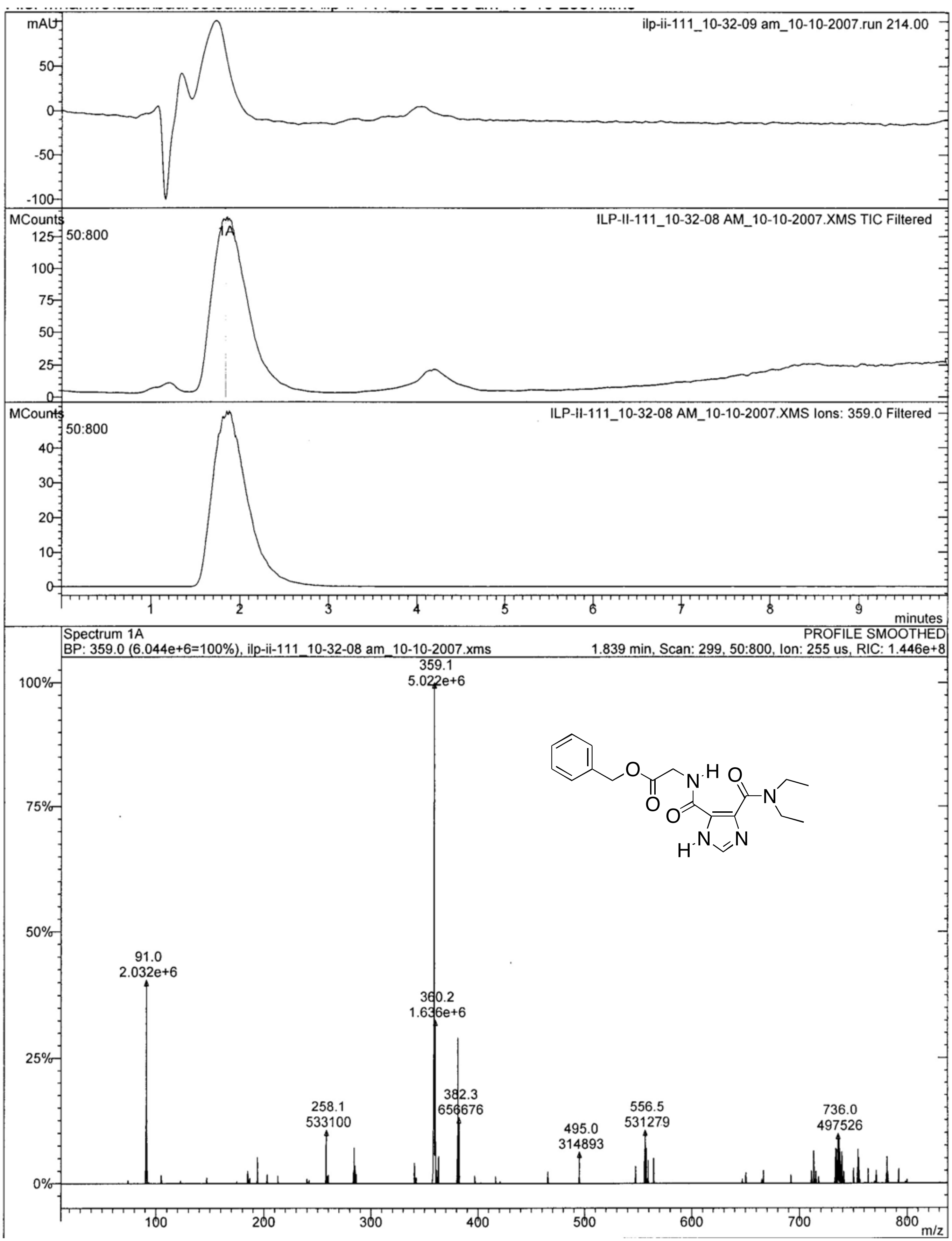


**Figure S42.** LC/MS data for **5{**24}.

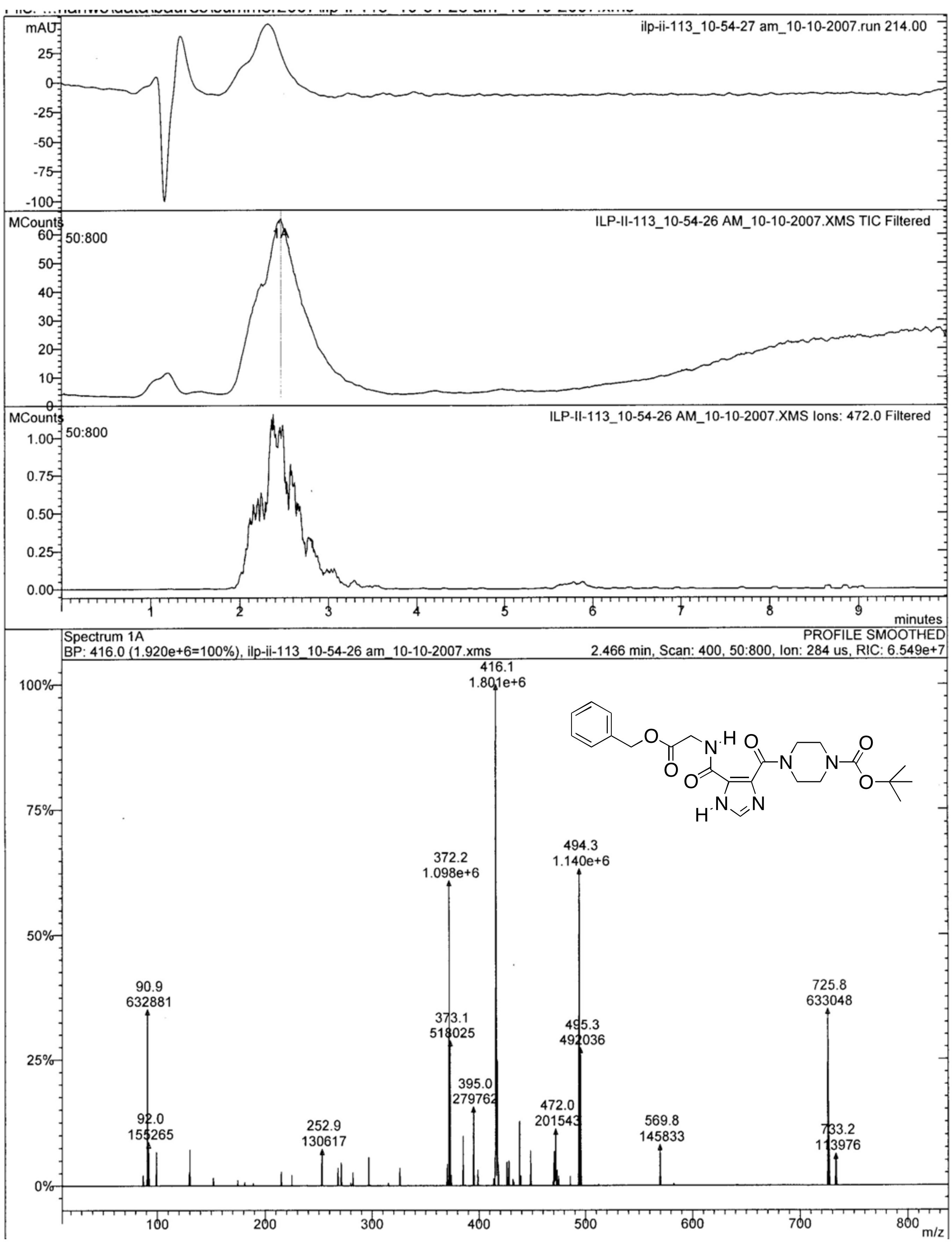


**Figure S25.** LC/MS data for **5{**25}.

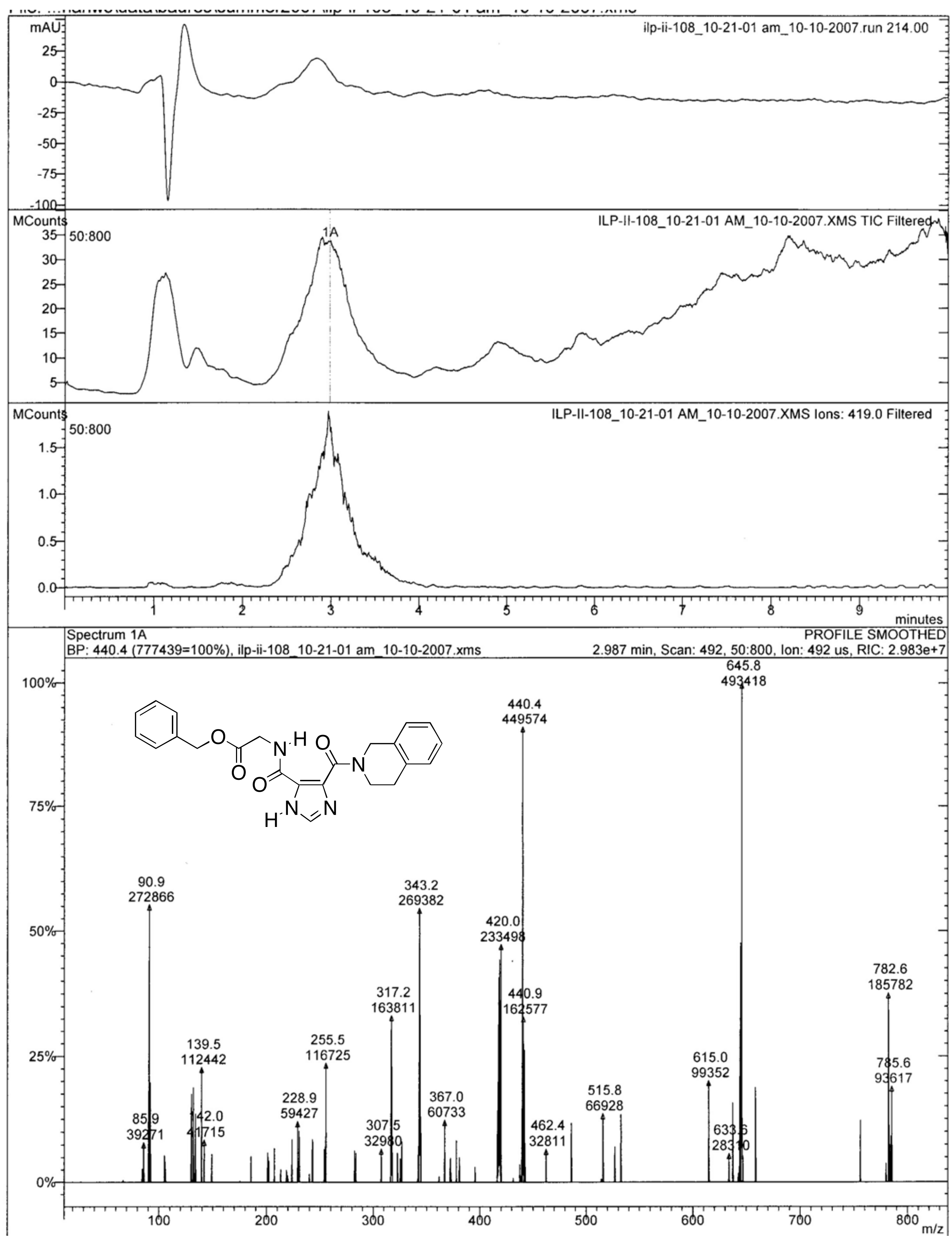


**Figure S26.** LC/MS data for **5{**26}.

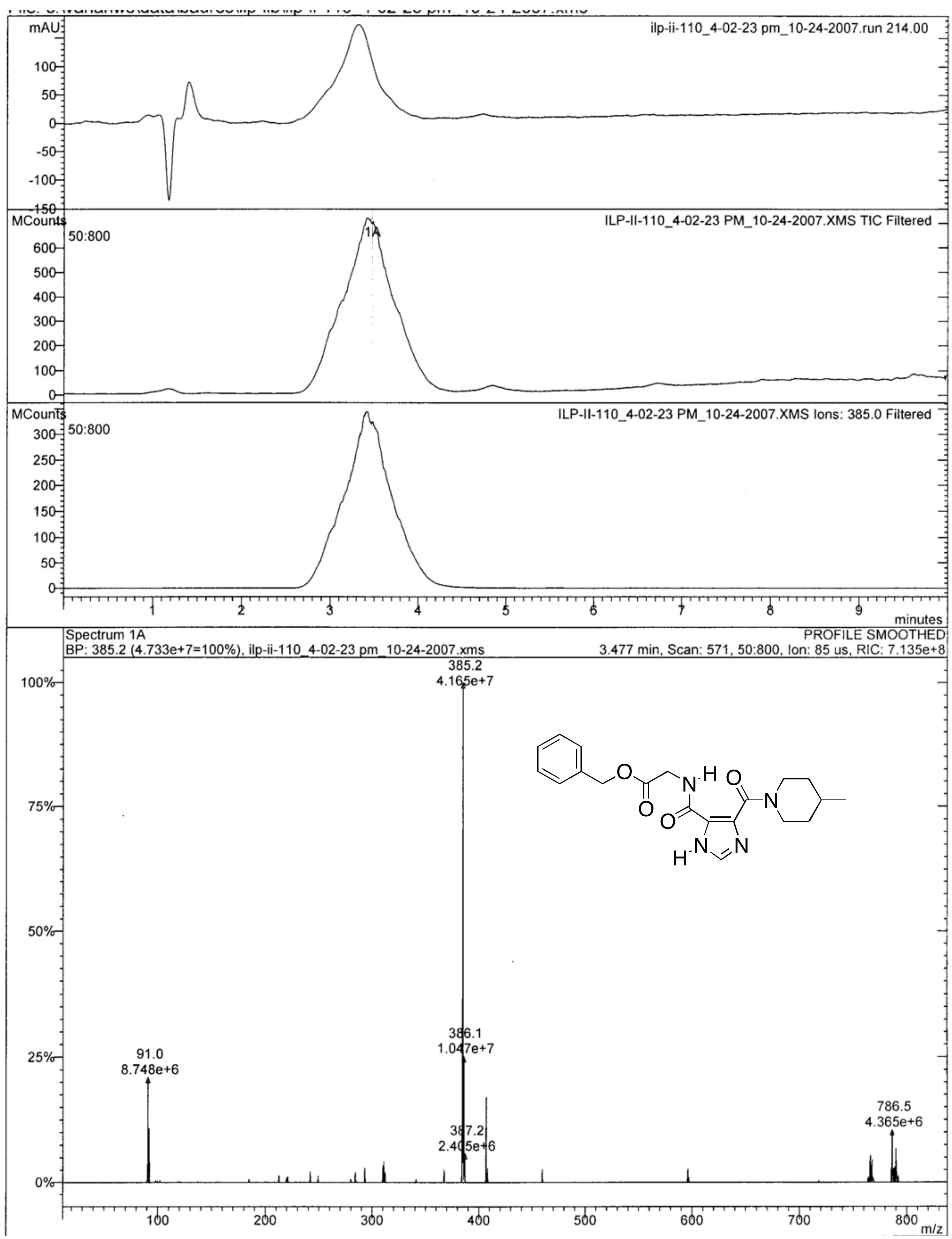


**Figure S27.** LC/MS data for **5{**27}.

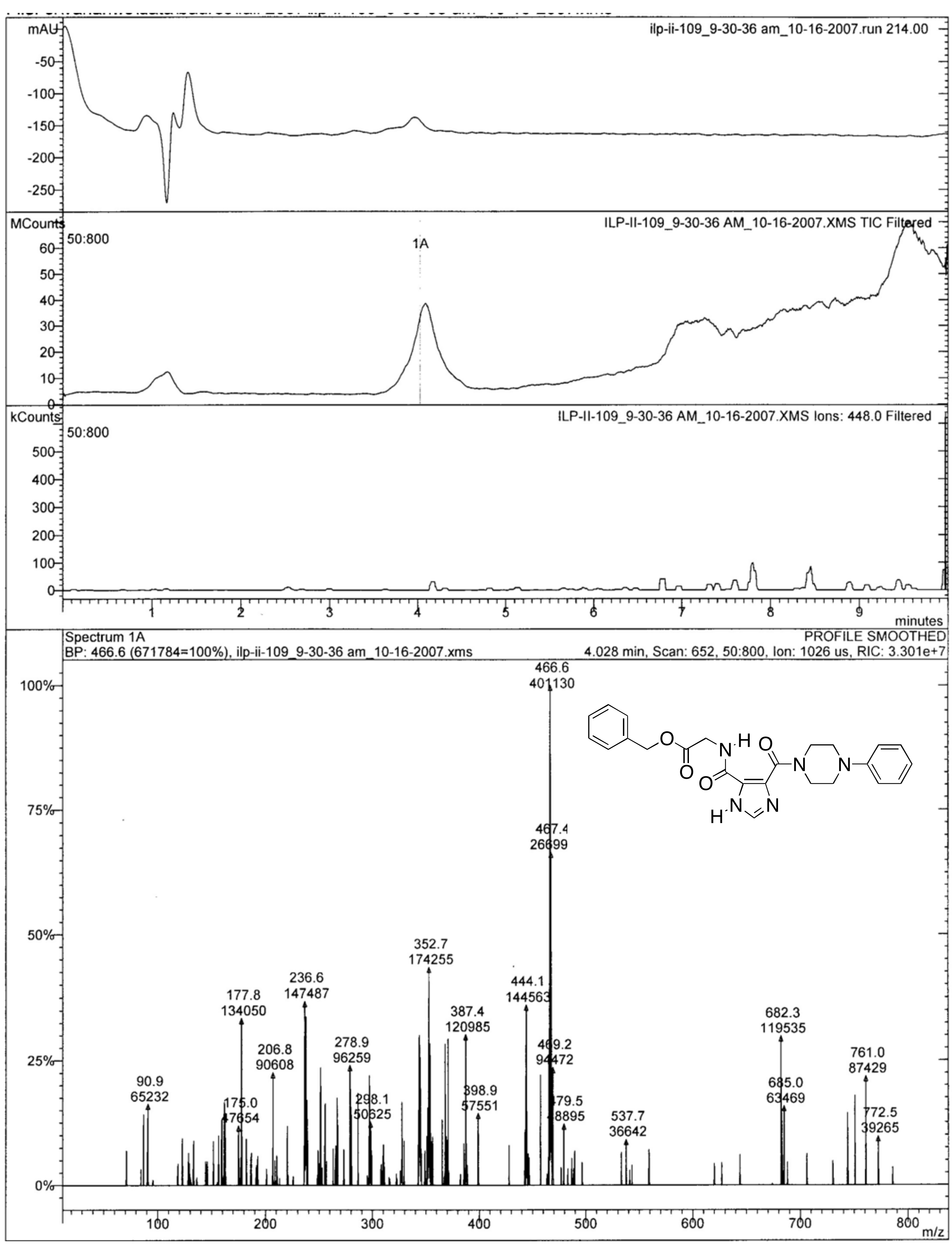


**Figure S28.** LC/MS data for **5{**28}.

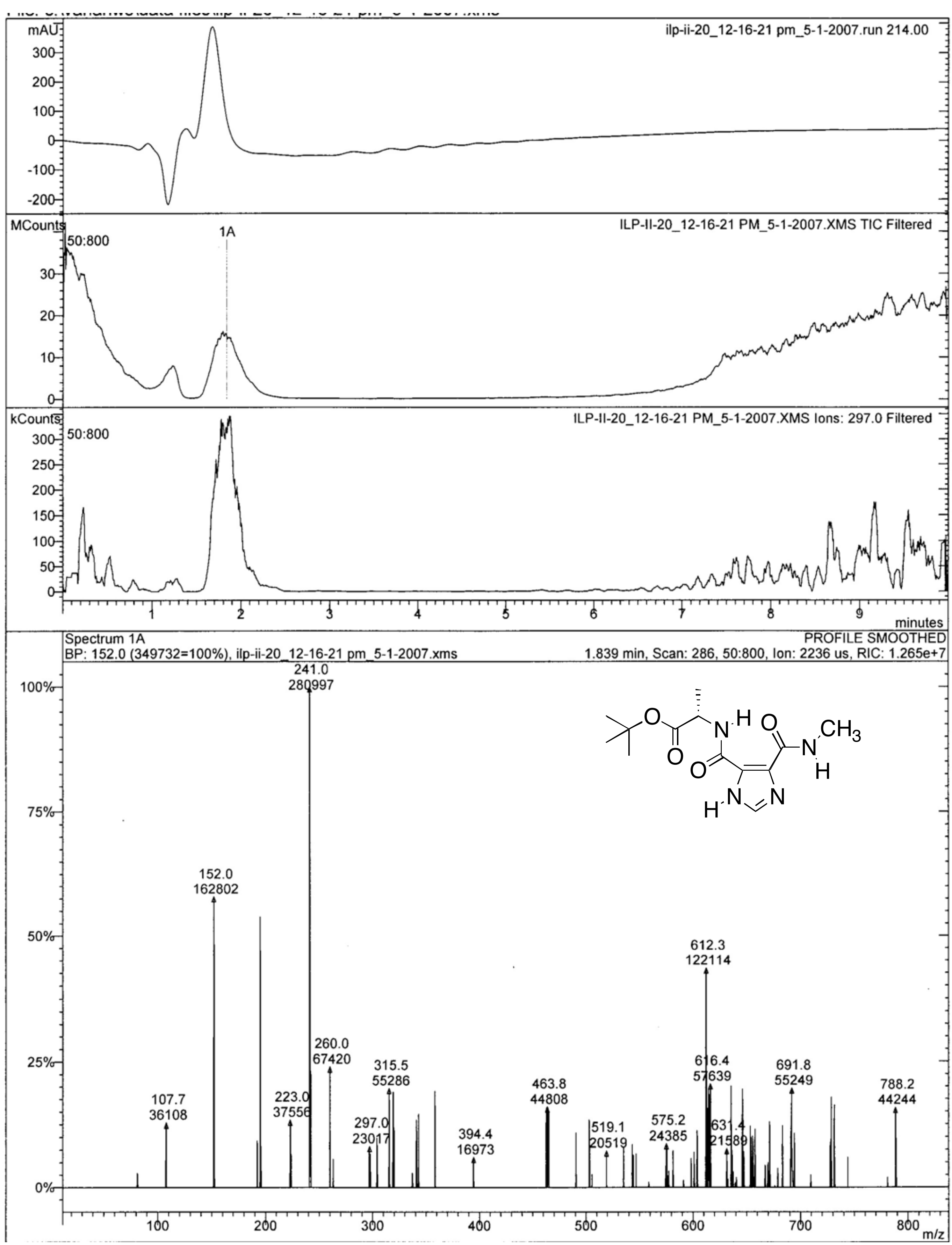


**Figure S29.** LC/MS data for **5{**29}.

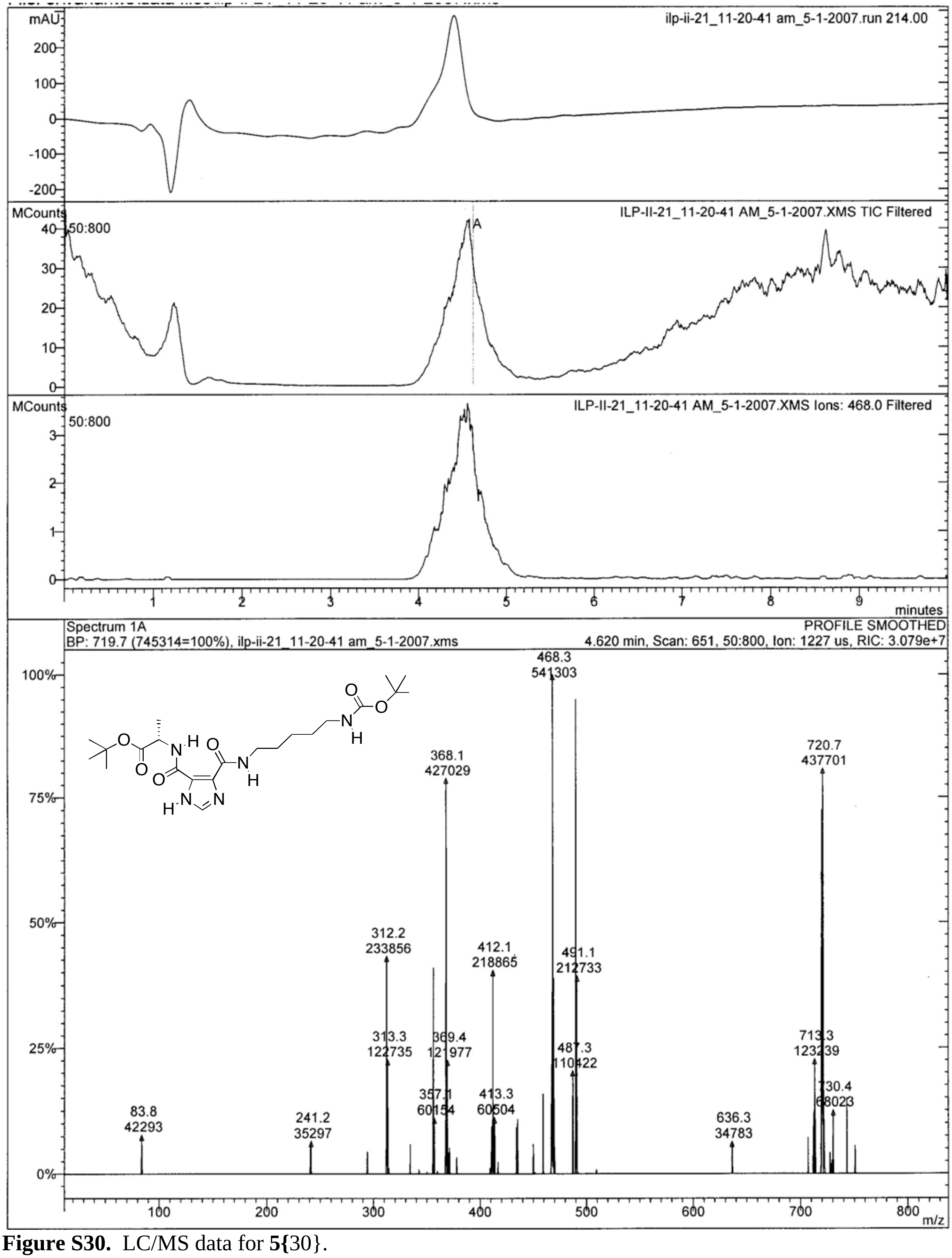


**Figure S30.** LC/MS data for **5{**30}.

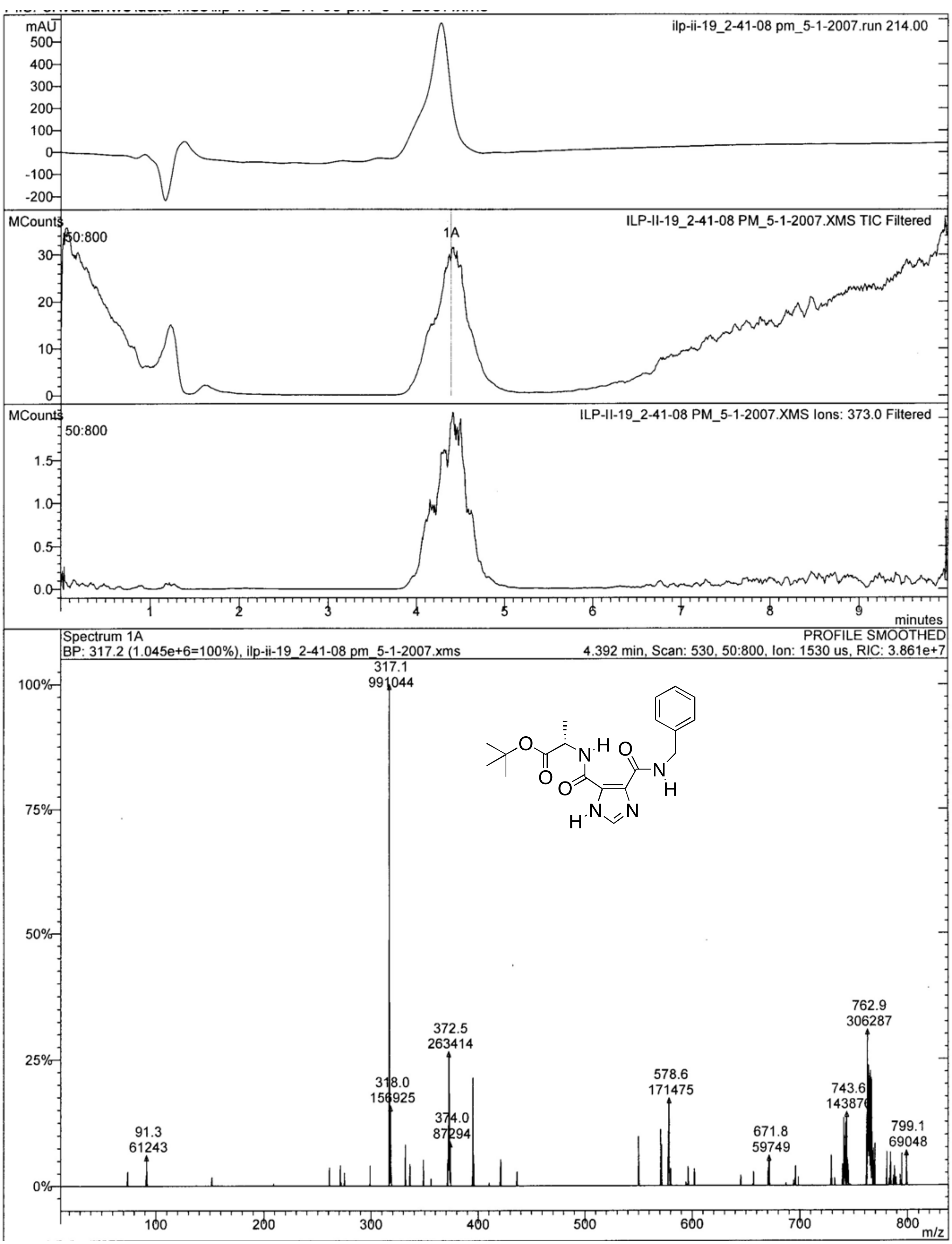


**Figure S31.** LC/MS data for **5{**31}.

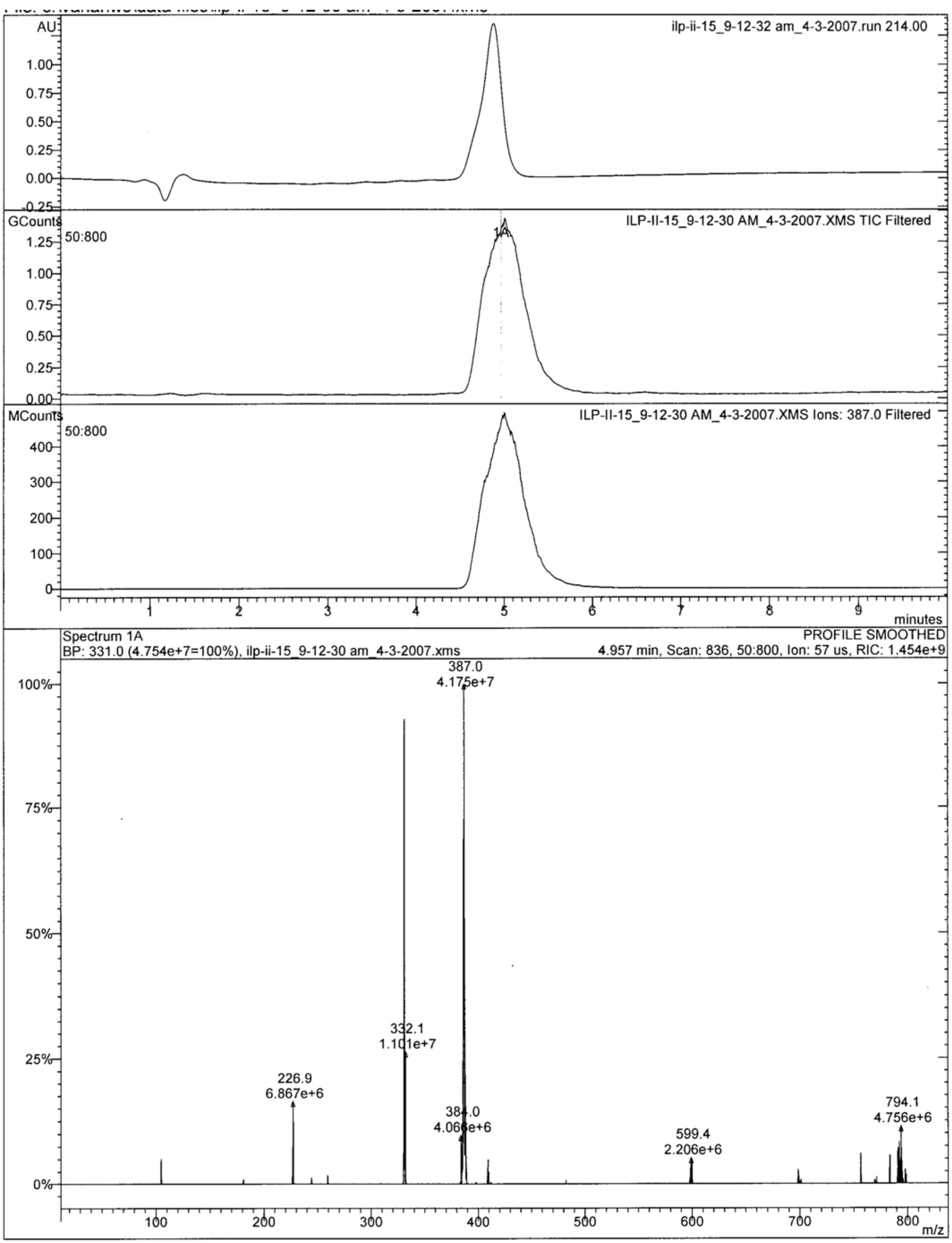


**Figure S32.** LC/MS data for **5{**32}.

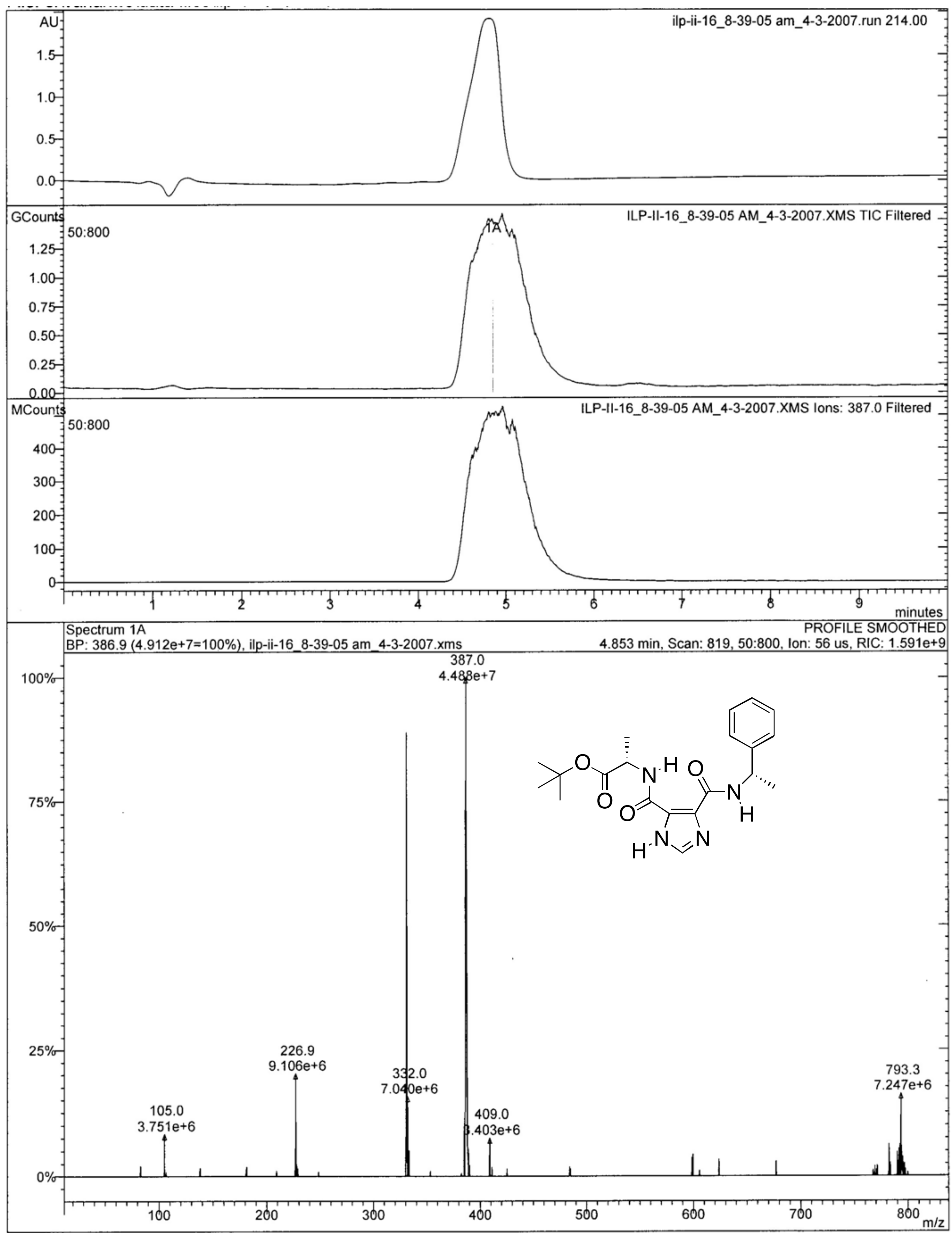


**Figure S33.** LC/MS data for **5{**33}.

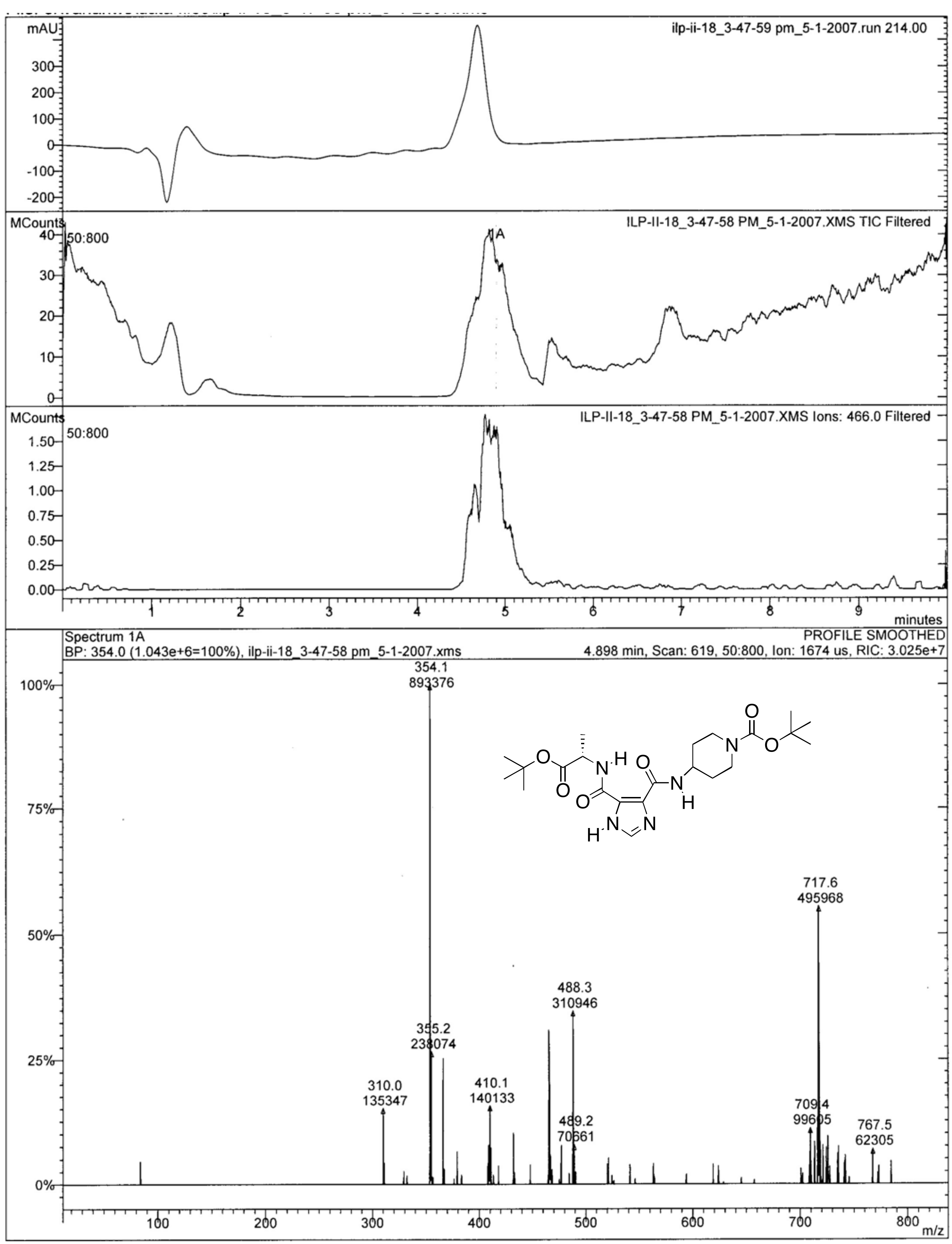


**Figure S34.** LC/MS data for **5{**34}.

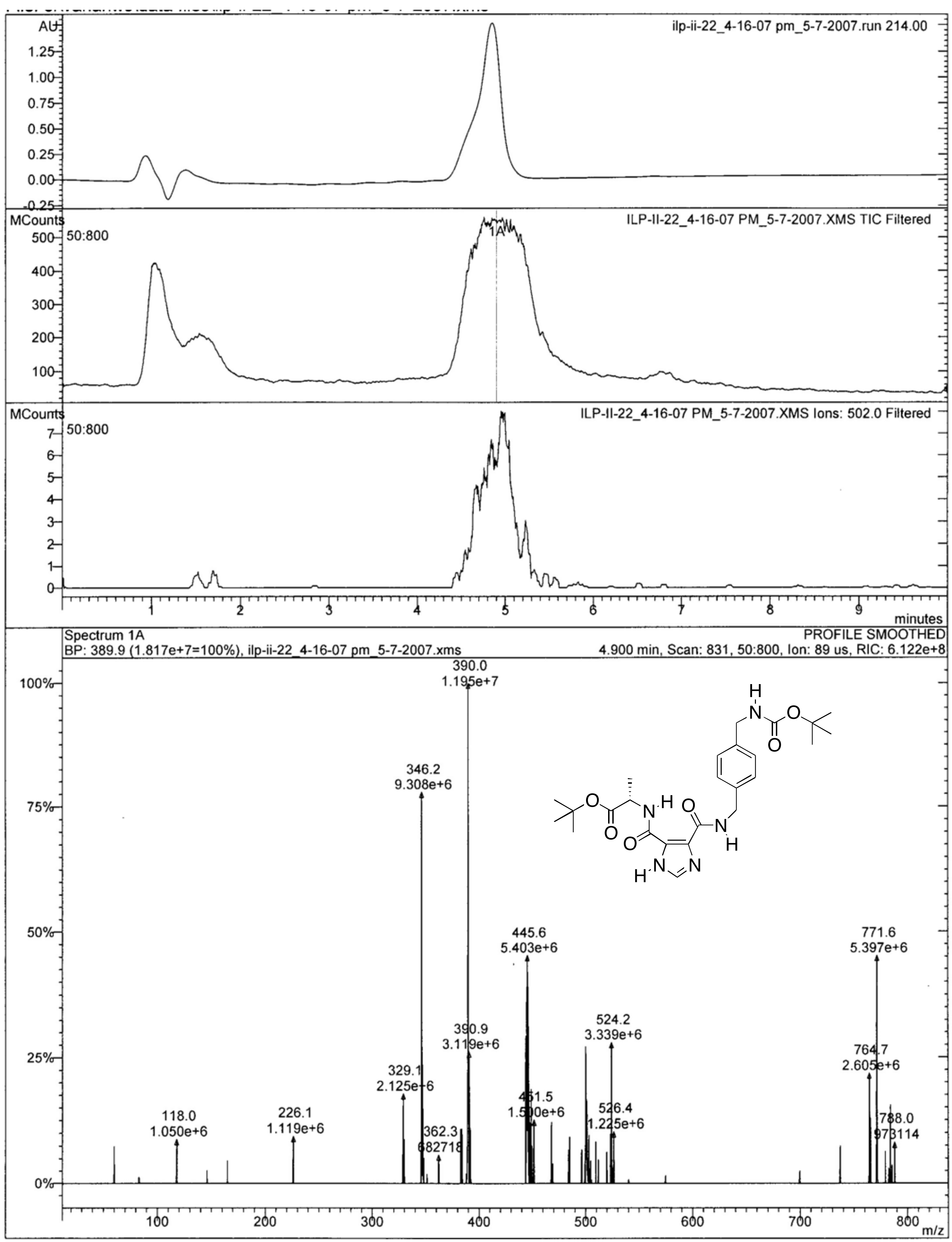


**Figure S35.** LC/MS data for **5{**35}.

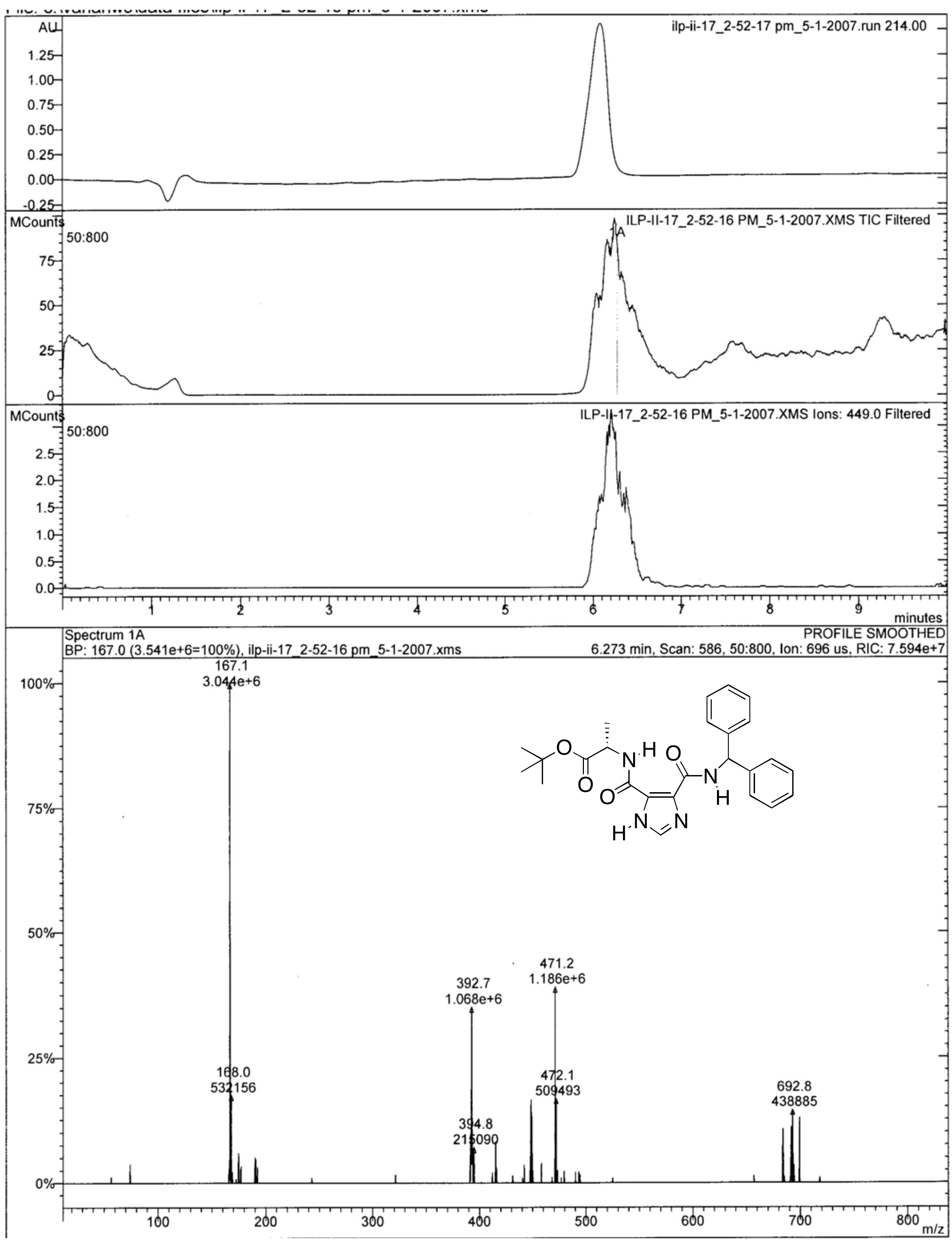


**Figure S36.** LC/MS data for **5{**36}.

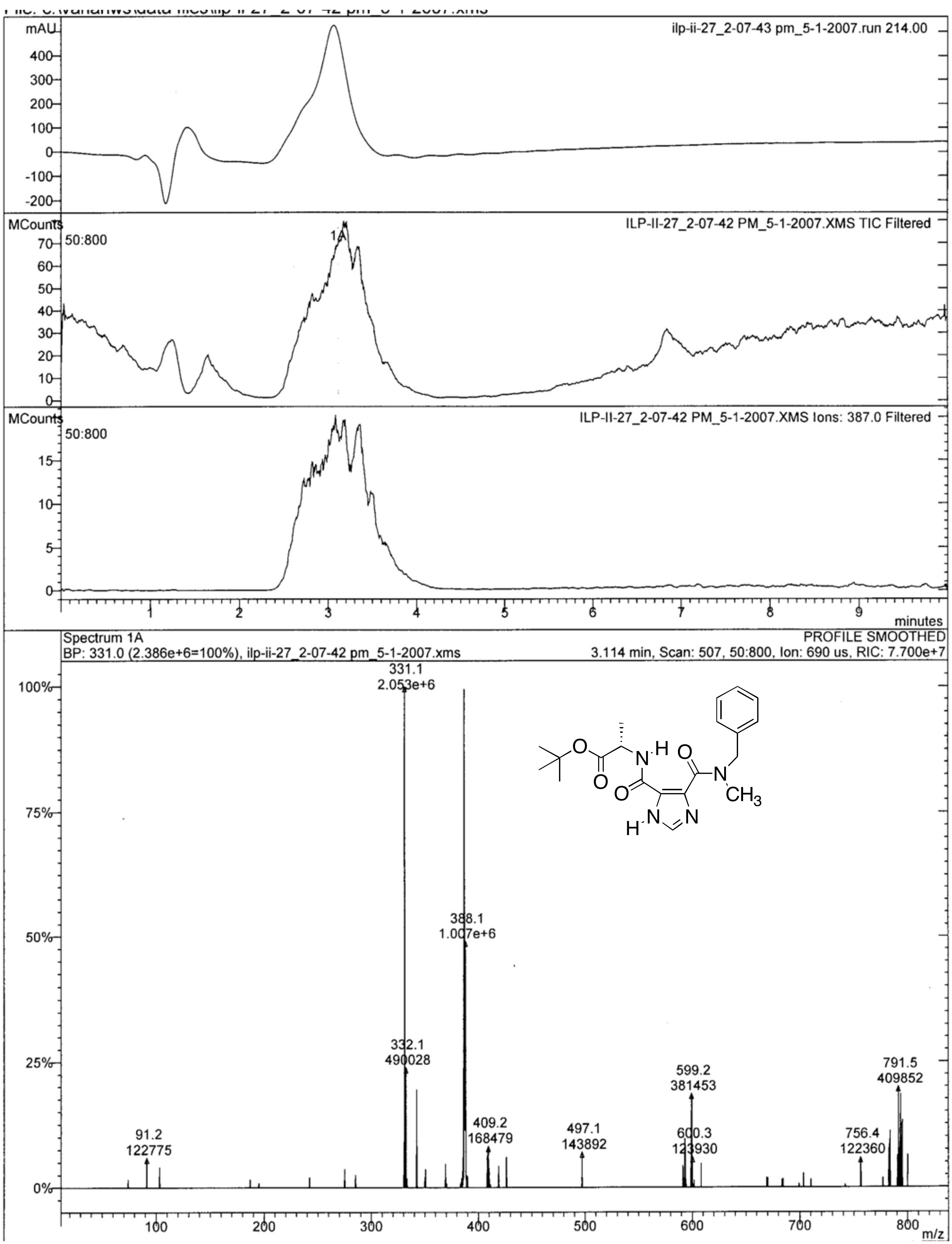


**Figure S37.** LC/MS data for **5{**37}.

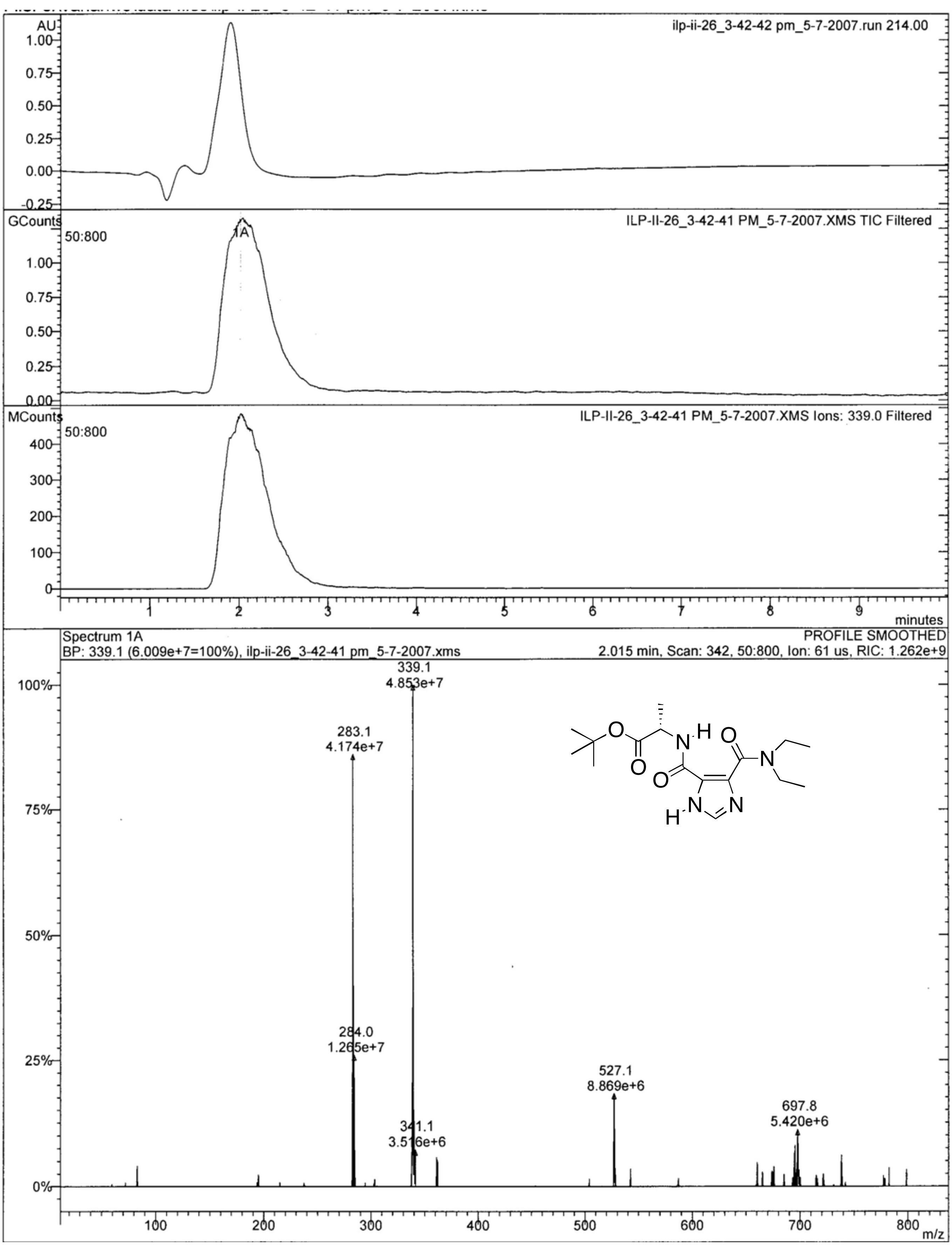


**Figure S38.** LC/MS data for **5{**38}.

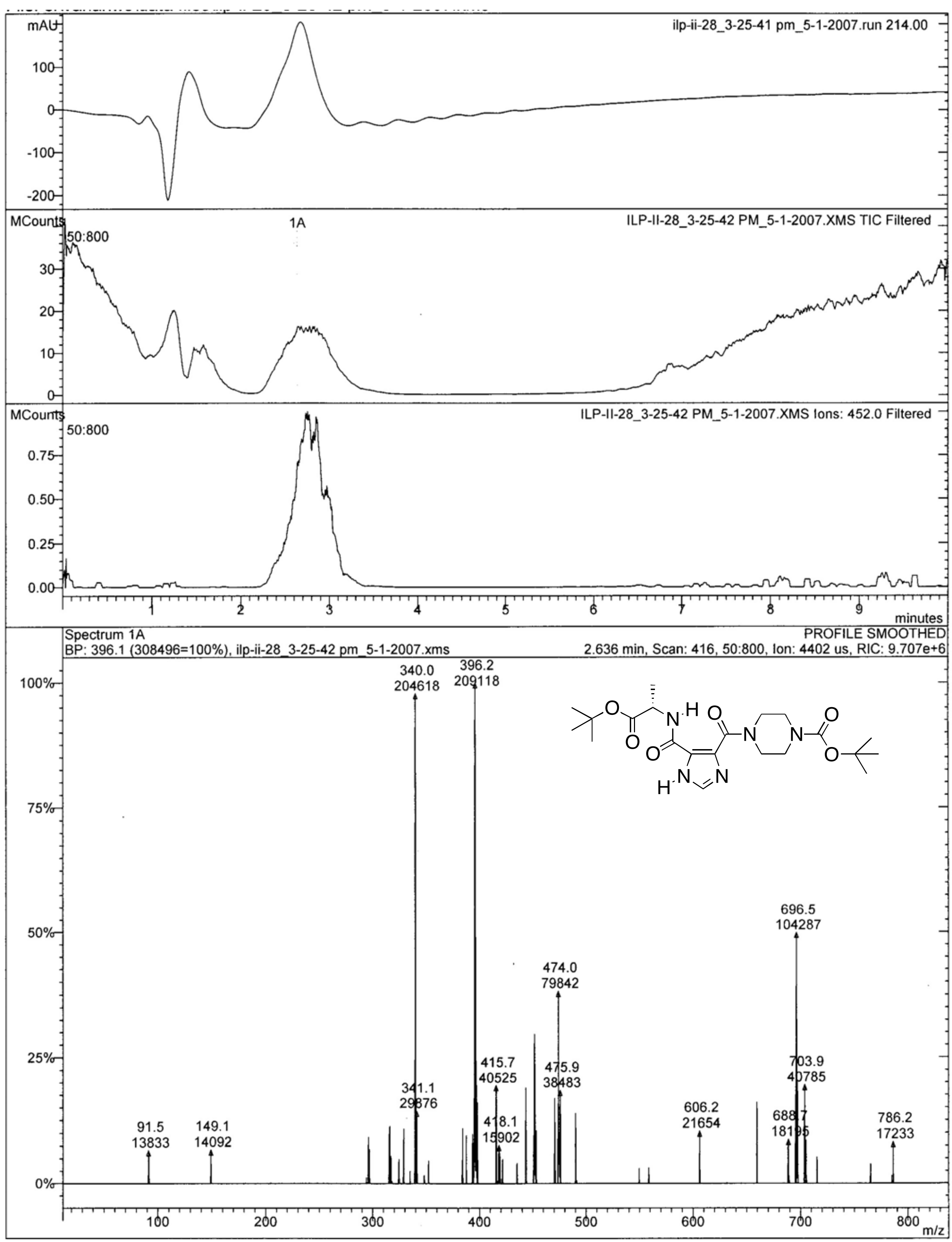


**Figure S39.** LC/MS data for **5{**39}.

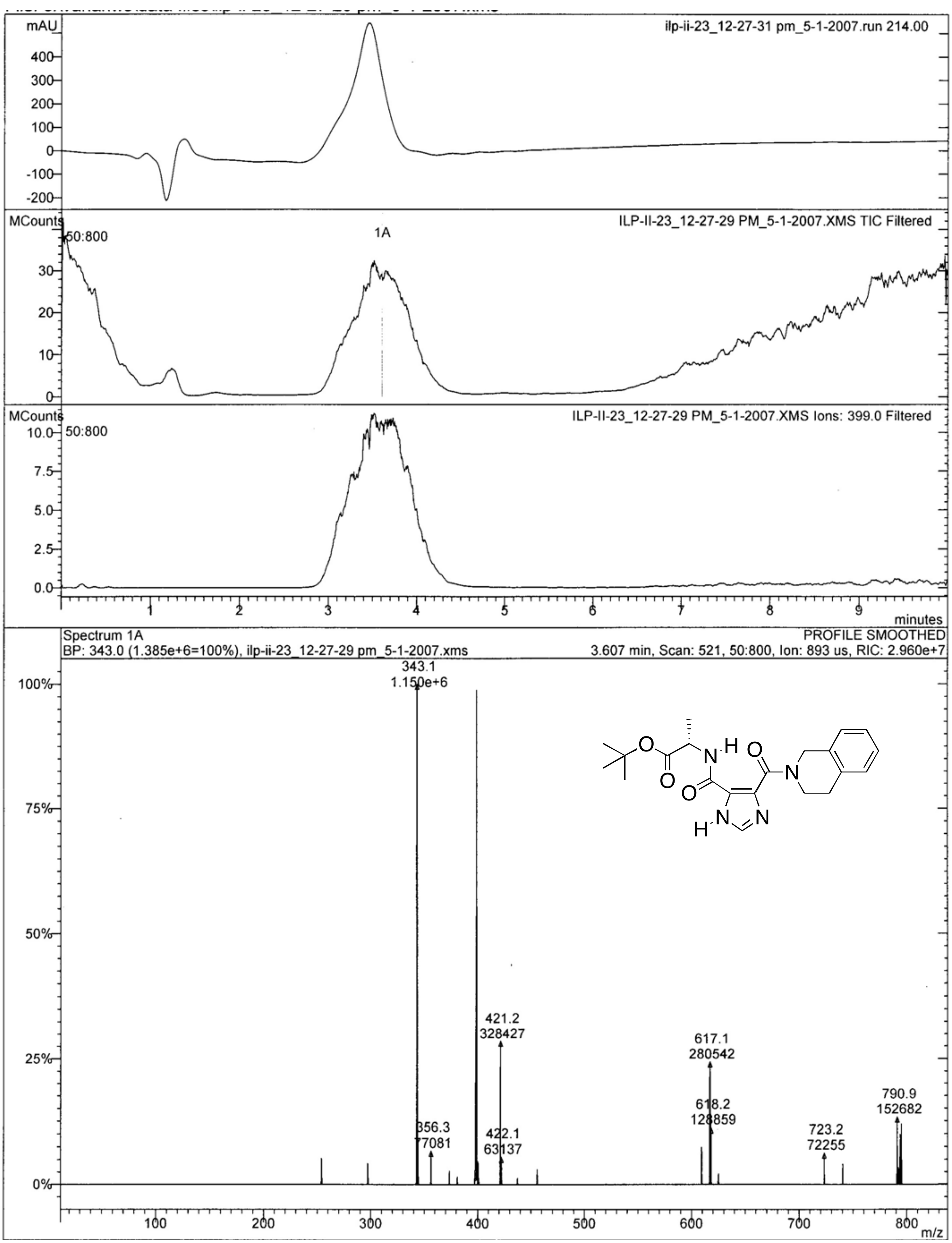


**Figure S40.** LC/MS data for **5{**40}.

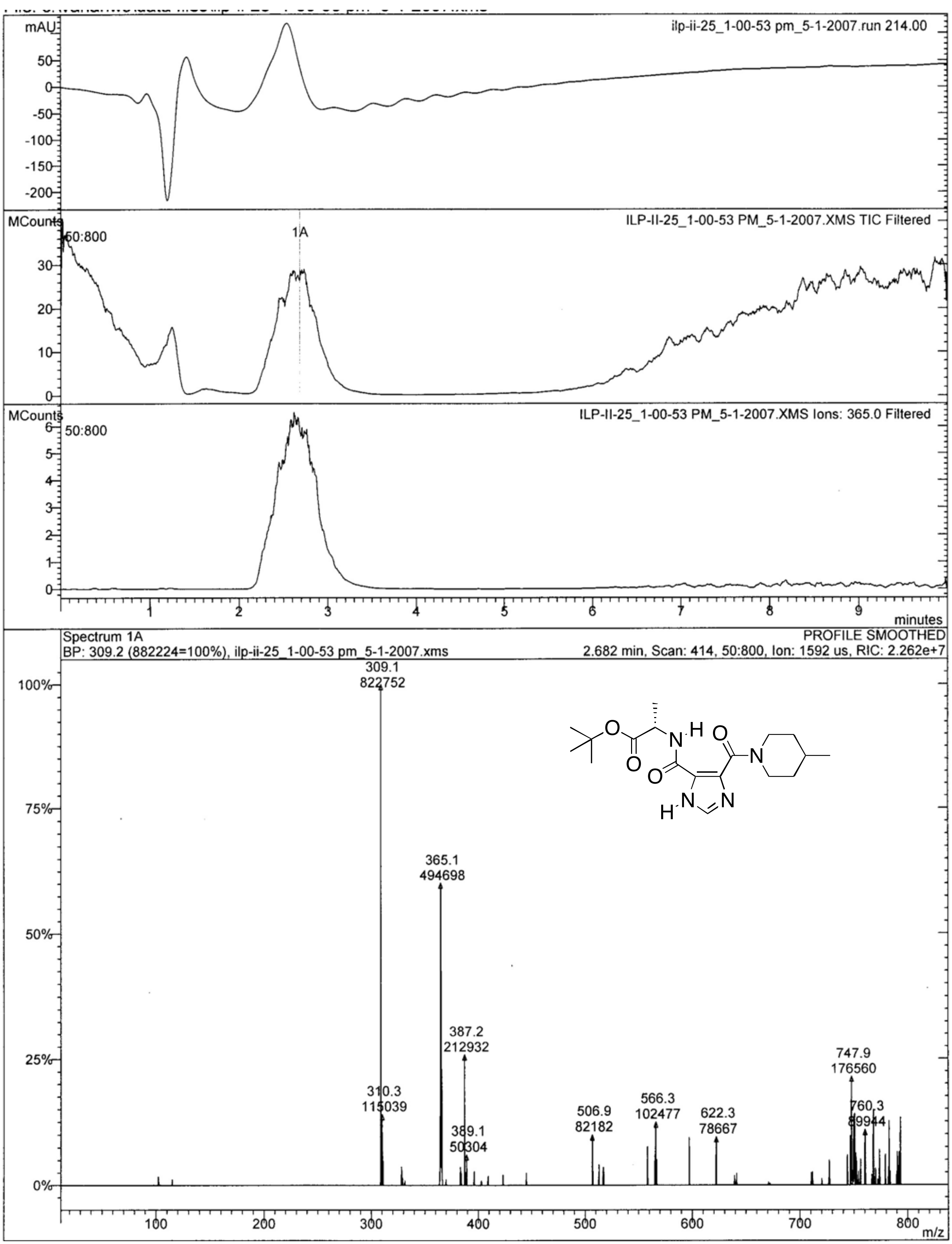


**Figure S41.** LC/MS data for **5{**41}.

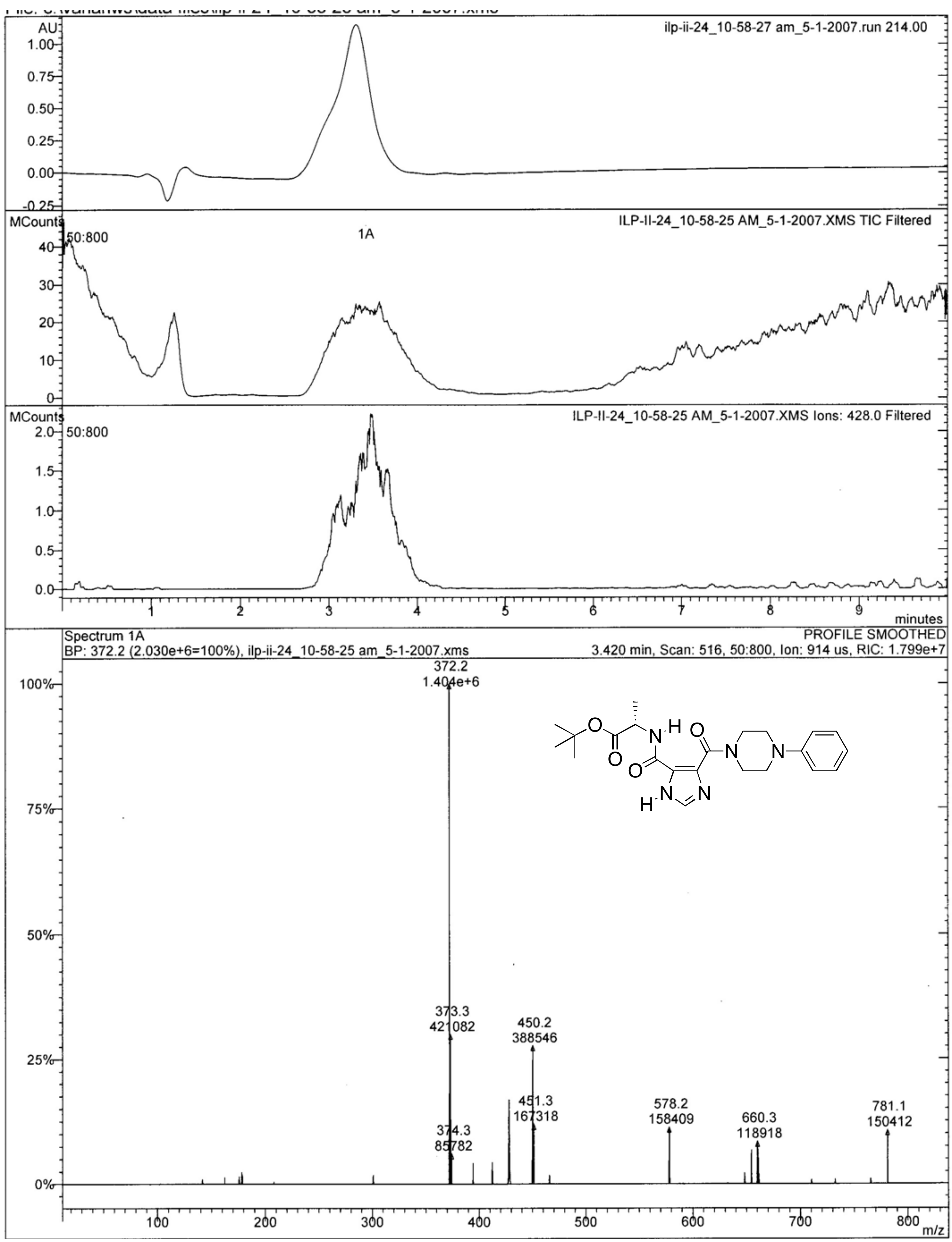


**Figure S42.** LC/MS data for **5{**42}.

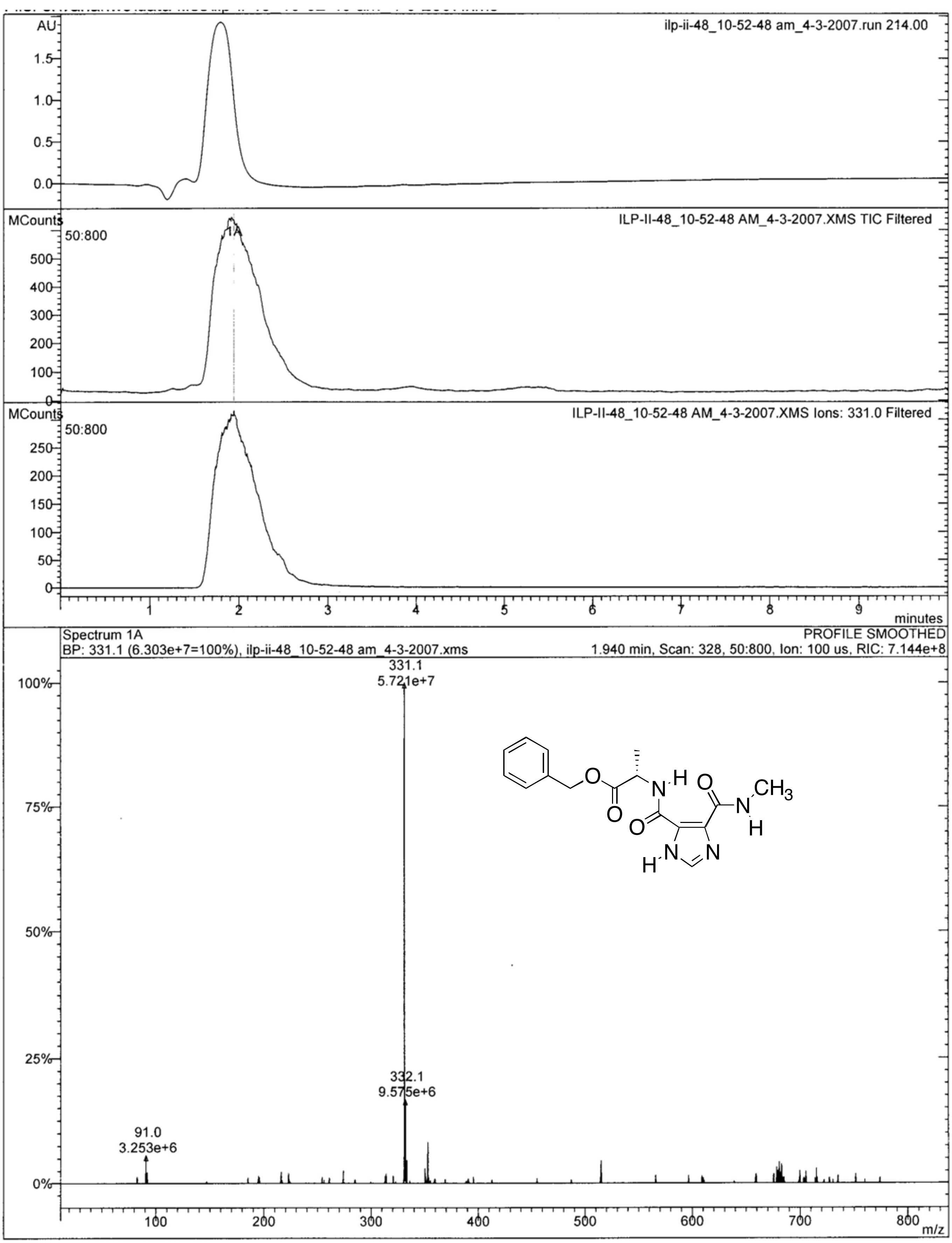


**Figure S43.** LC/MS data for **5{**43}.

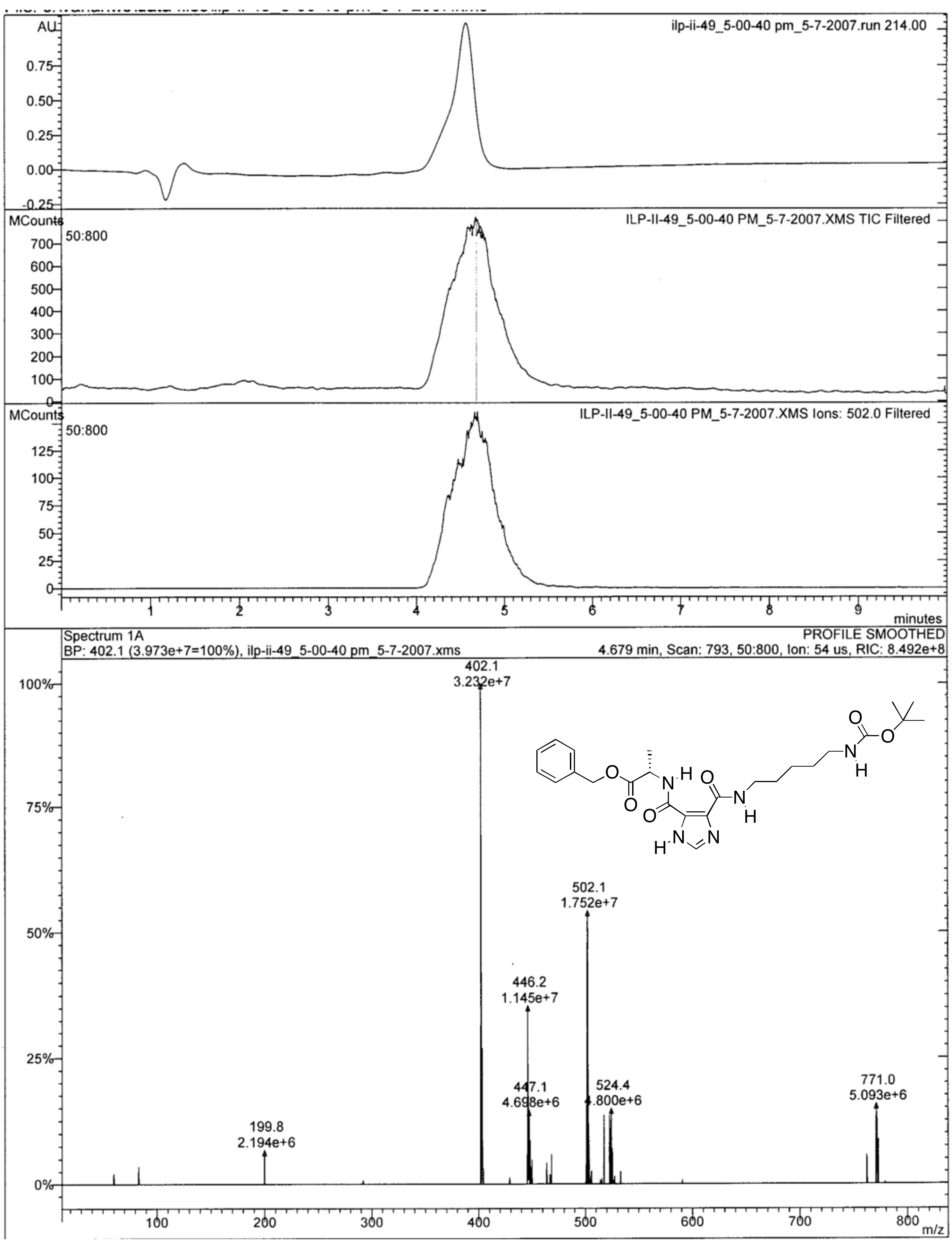


**Figure S44.** LC/MS data for **5{**44}.

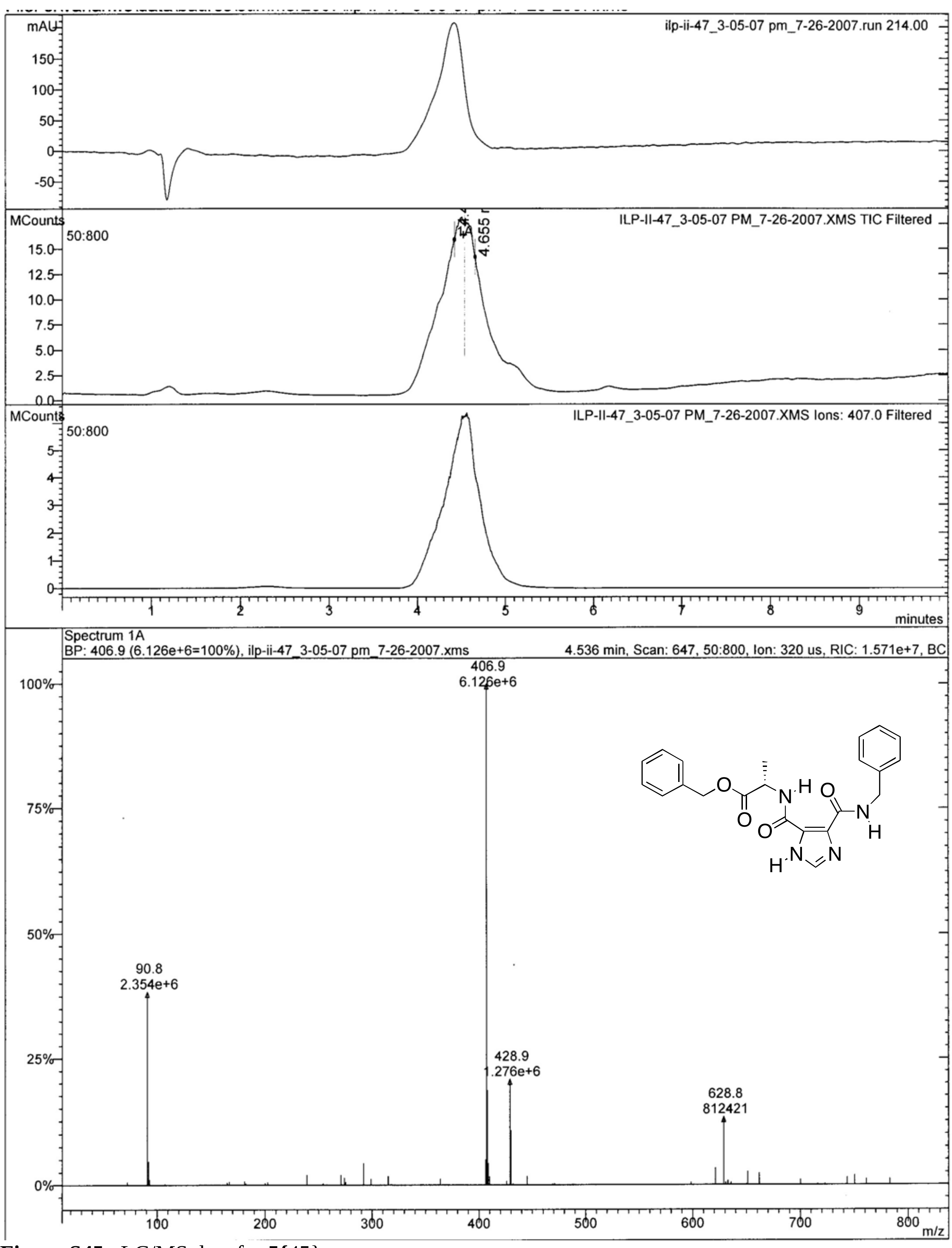


**Figure S45.** LC/MS data for **5{**45}.

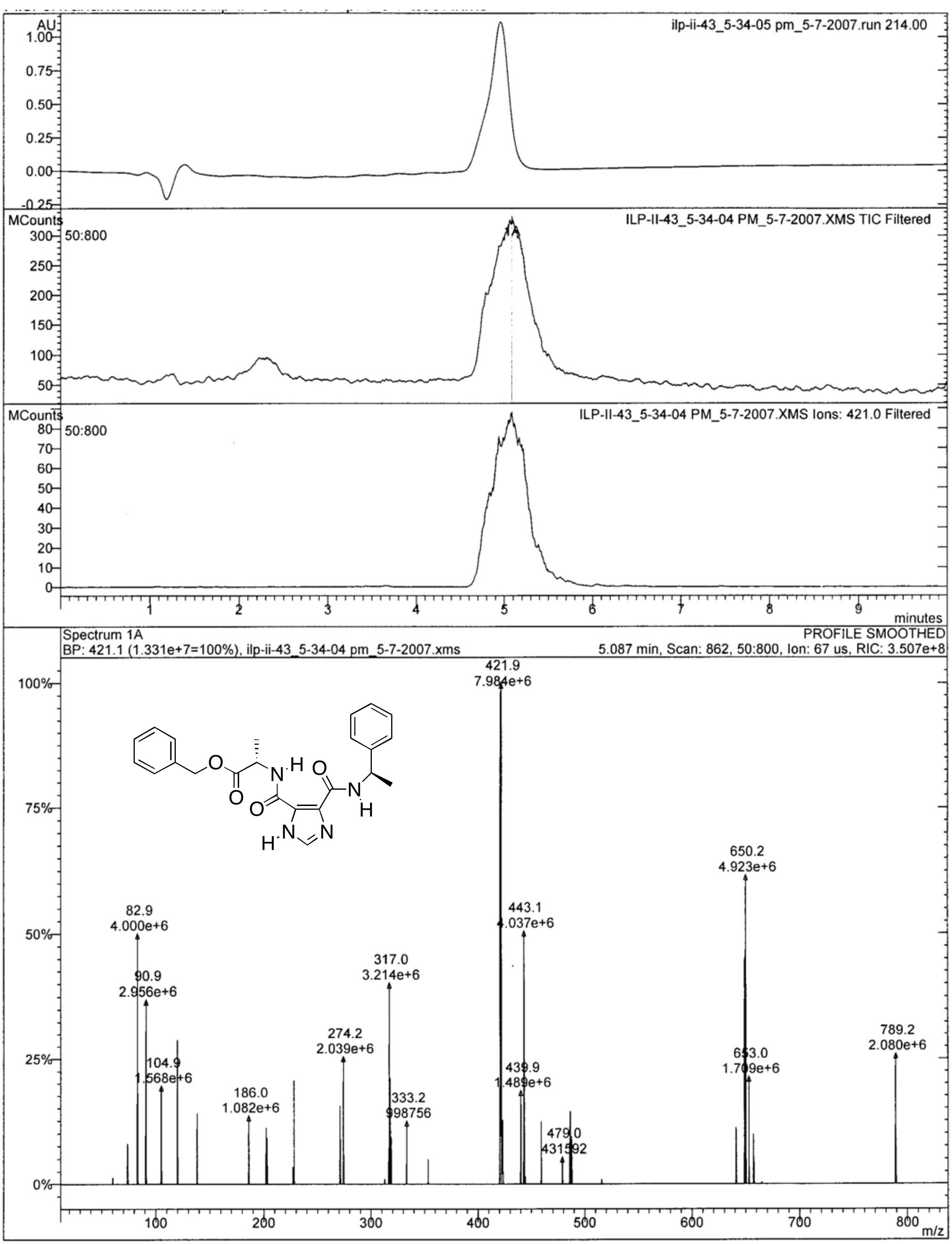


**Figure S46.** LC/MS data for **5{**46}.

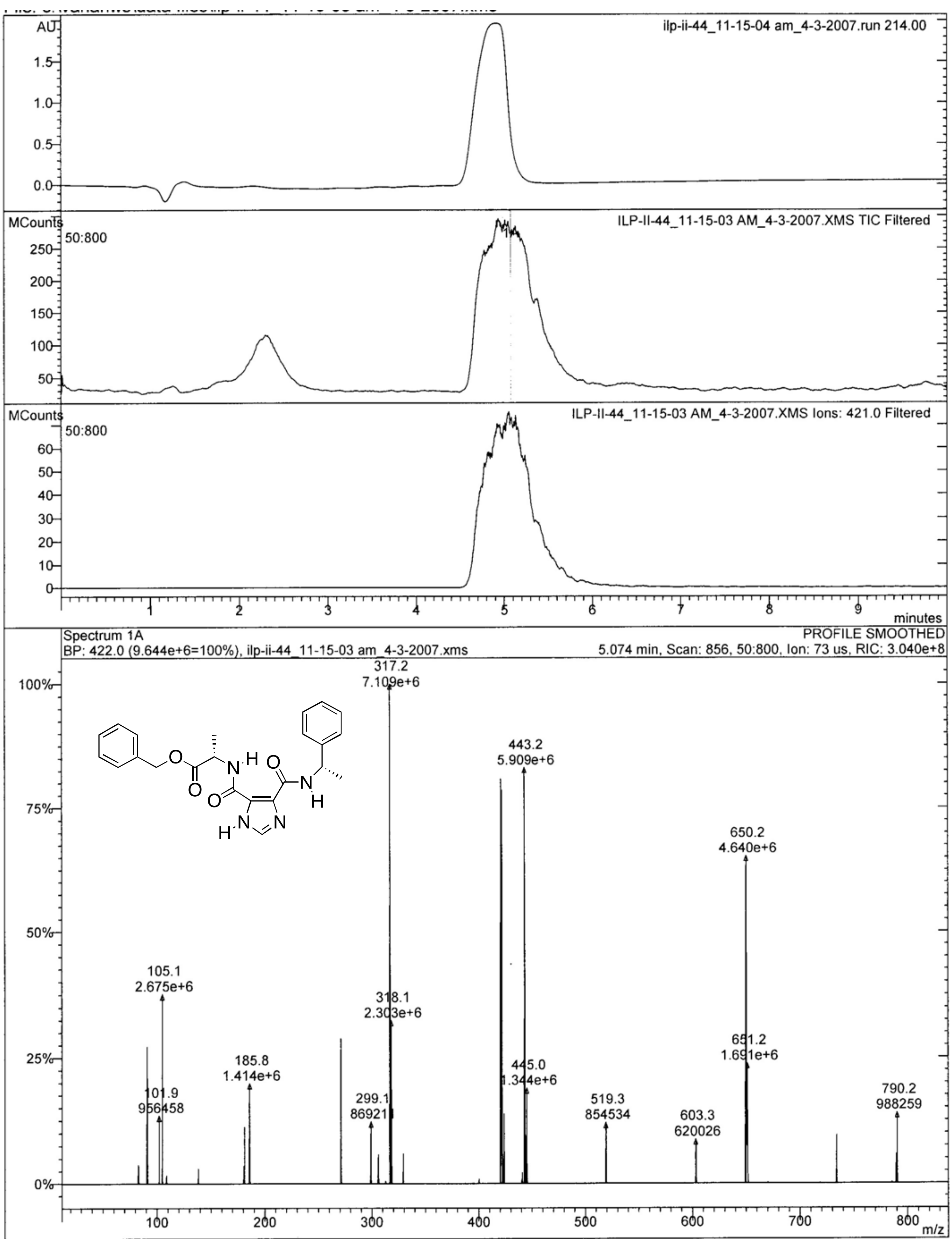


**Figure S47.** LC/MS data for **5{**47}.

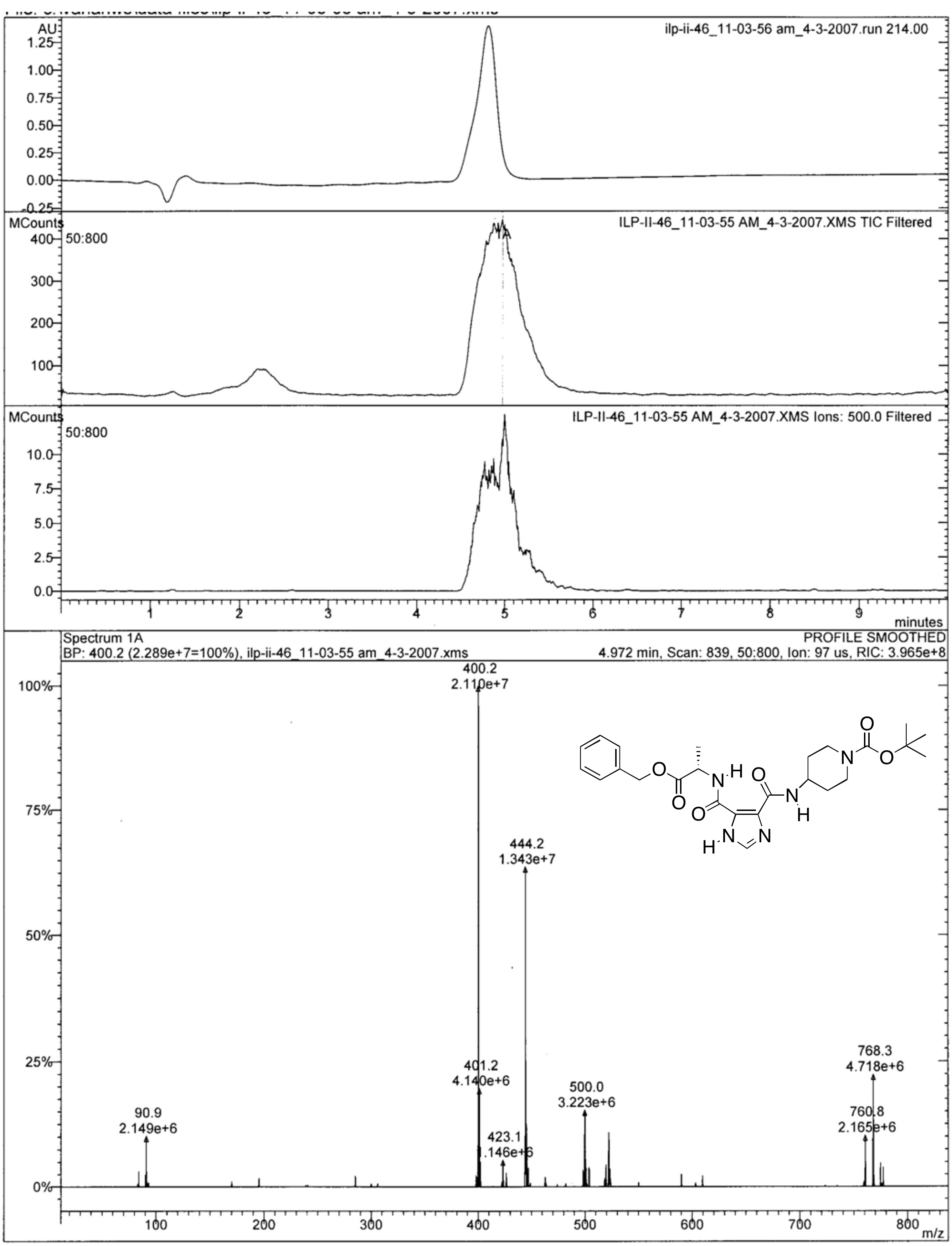


**Figure S48.** LC/MS data for **5{**48}.

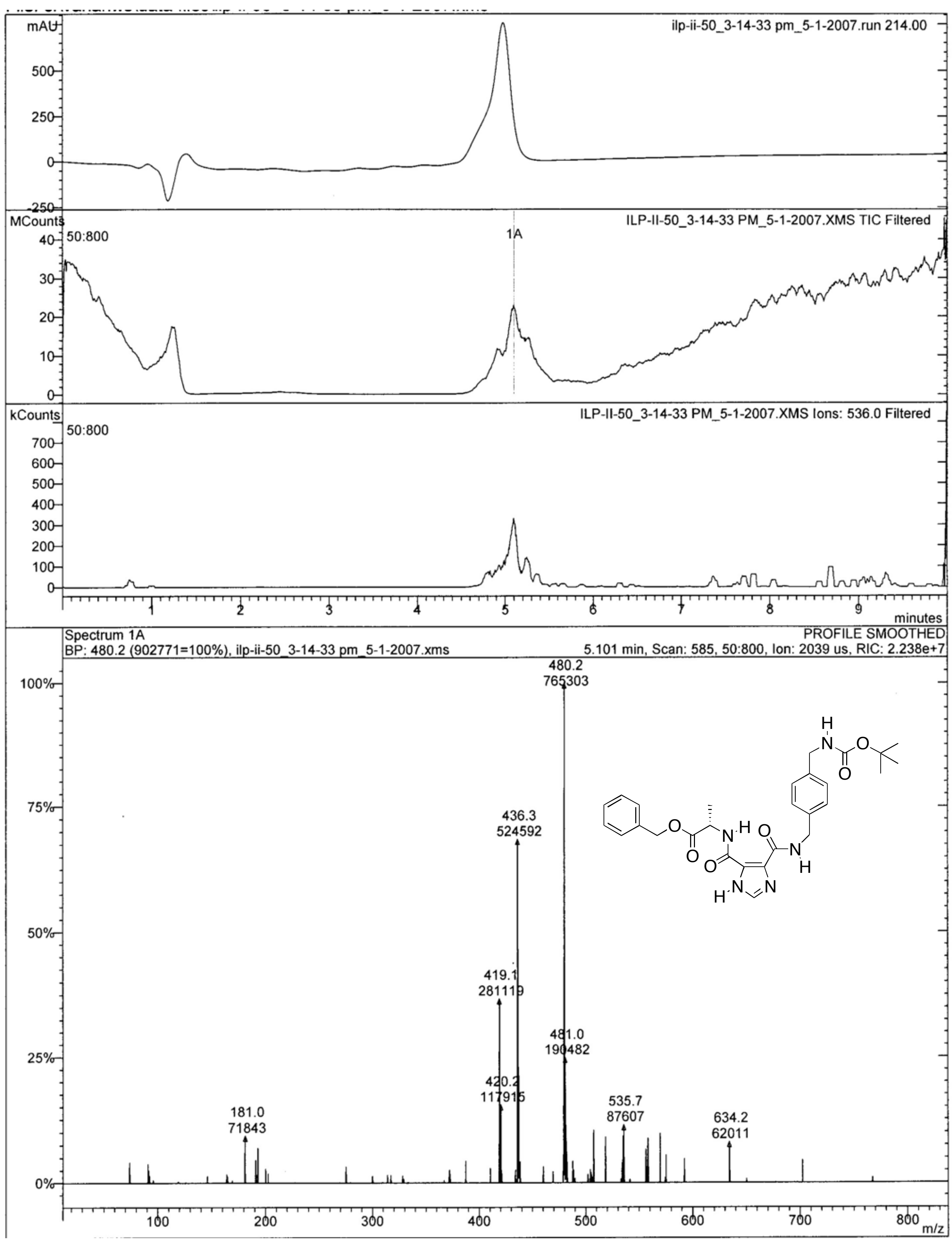


**Figure S49.** LC/MS data for **5{**49}.

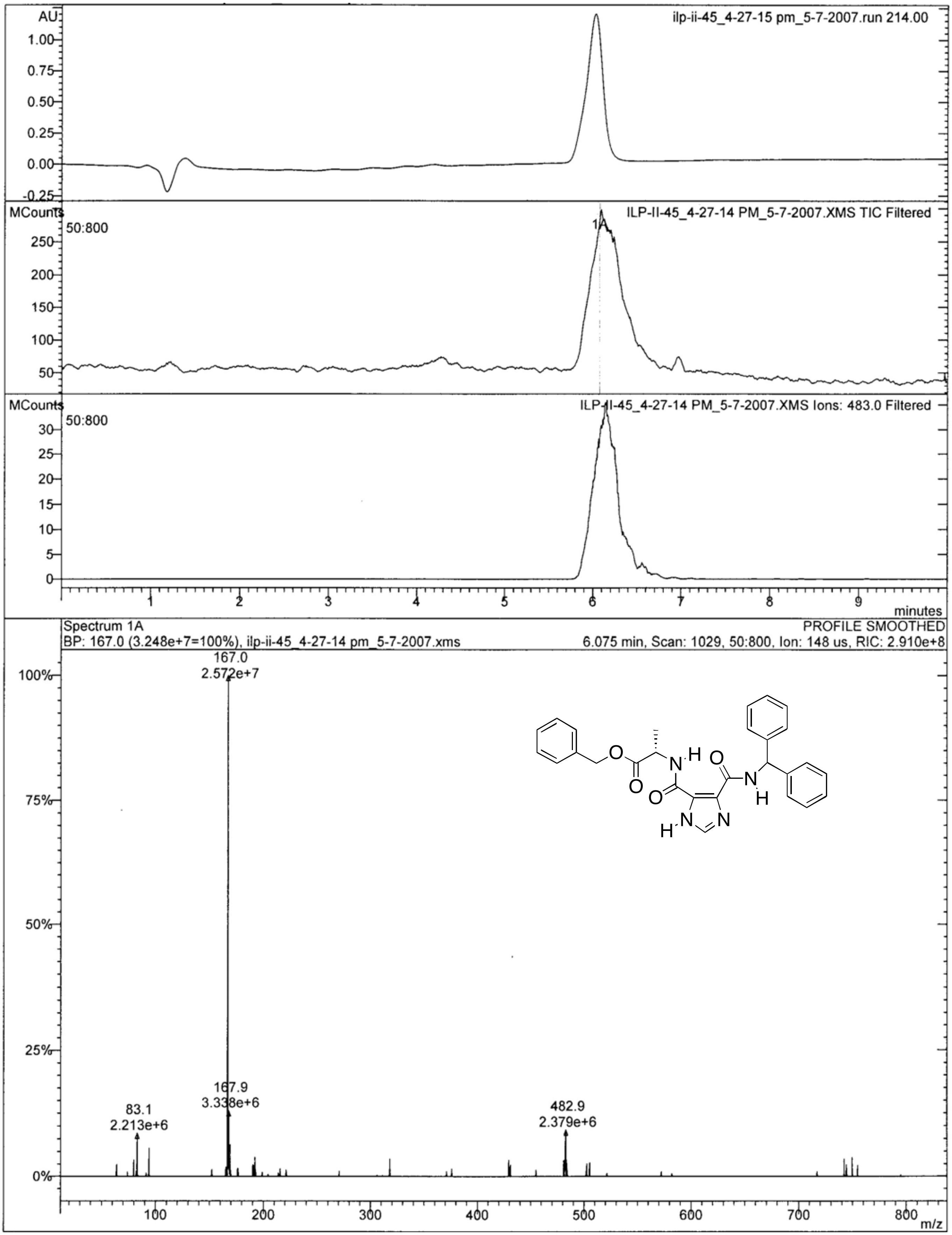


**Figure S50.** LC/MS data for **5{**50}.

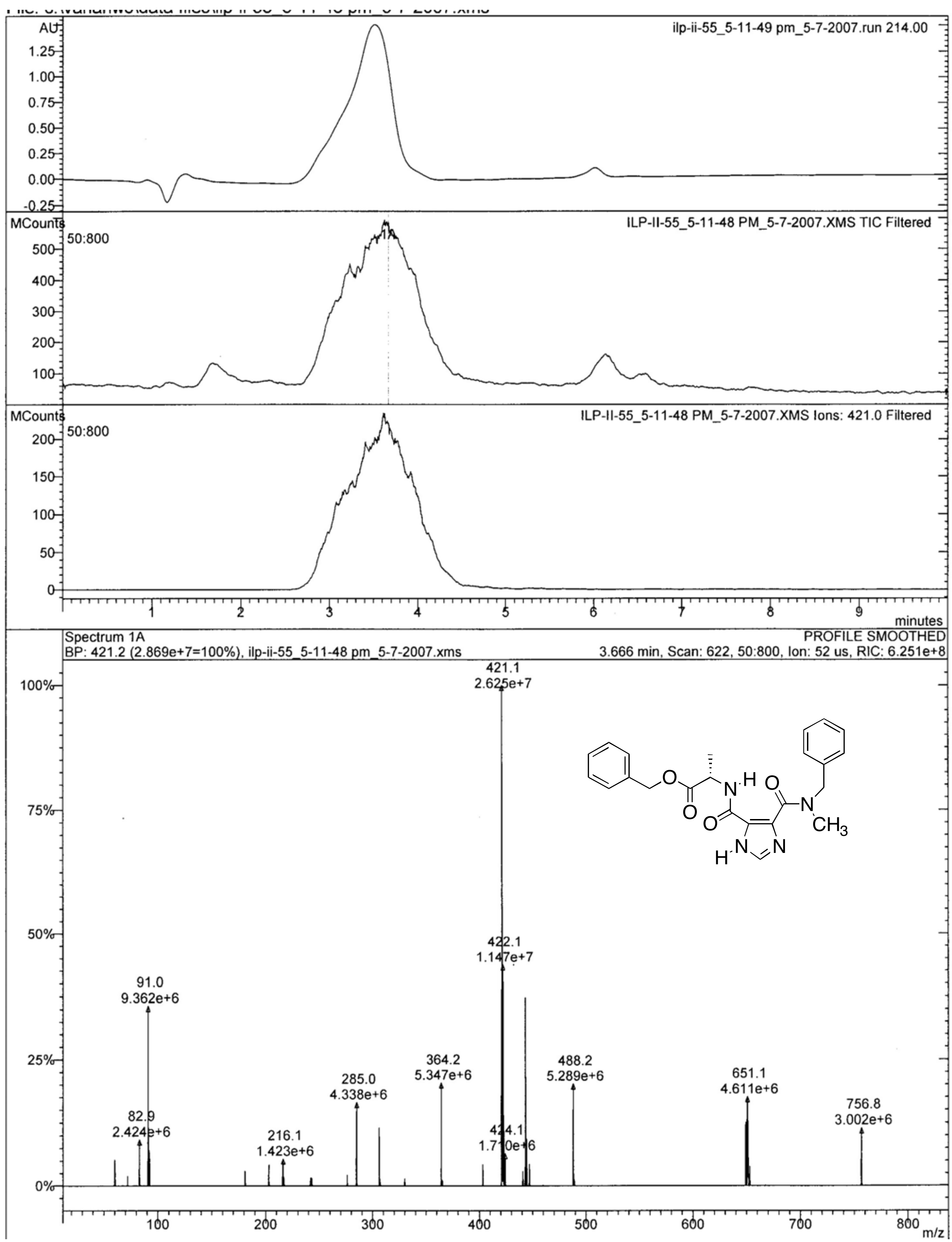


**Figure S51.** LC/MS data for **5{**51}.

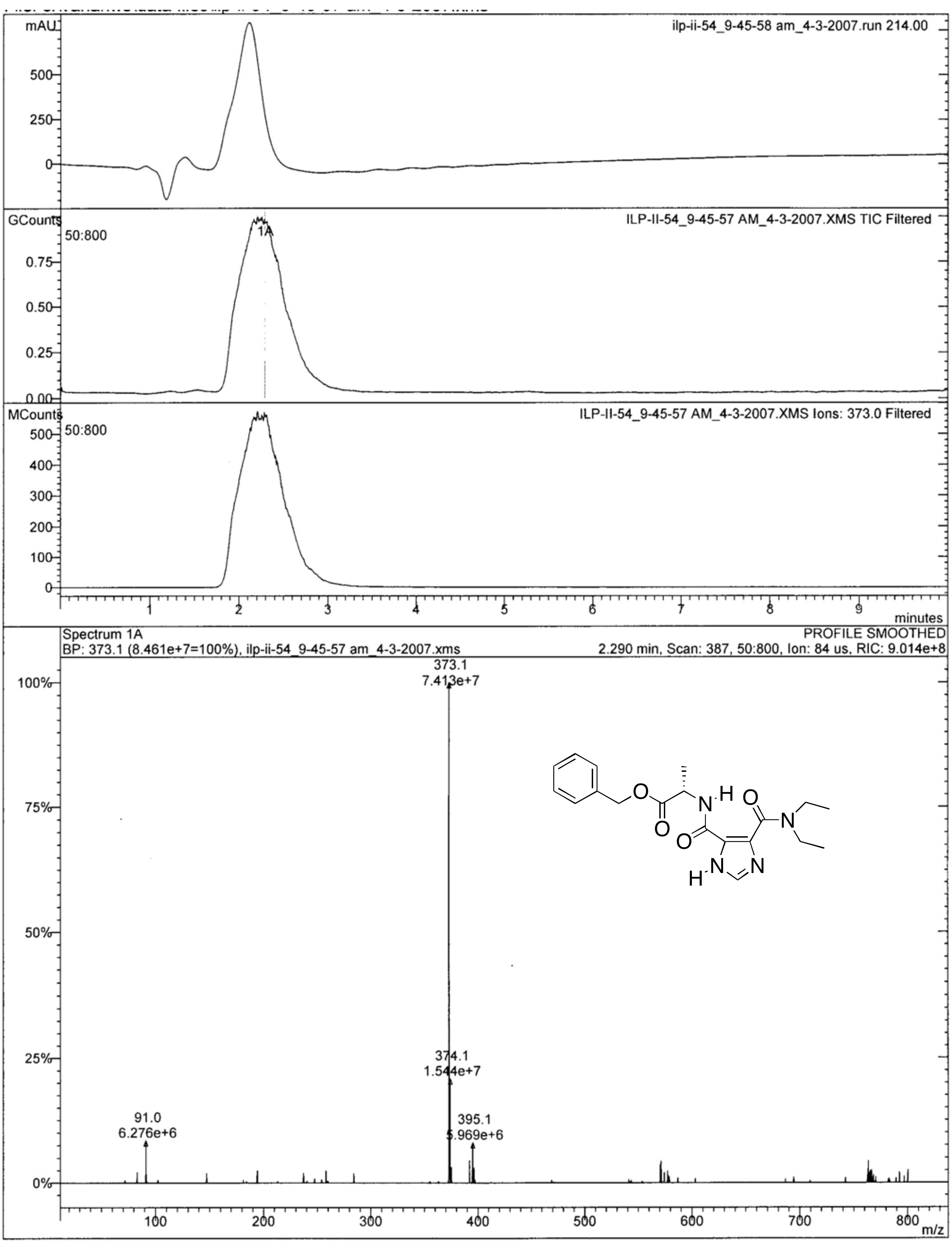


**Figure S52.** LC/MS data for **5{**52}.

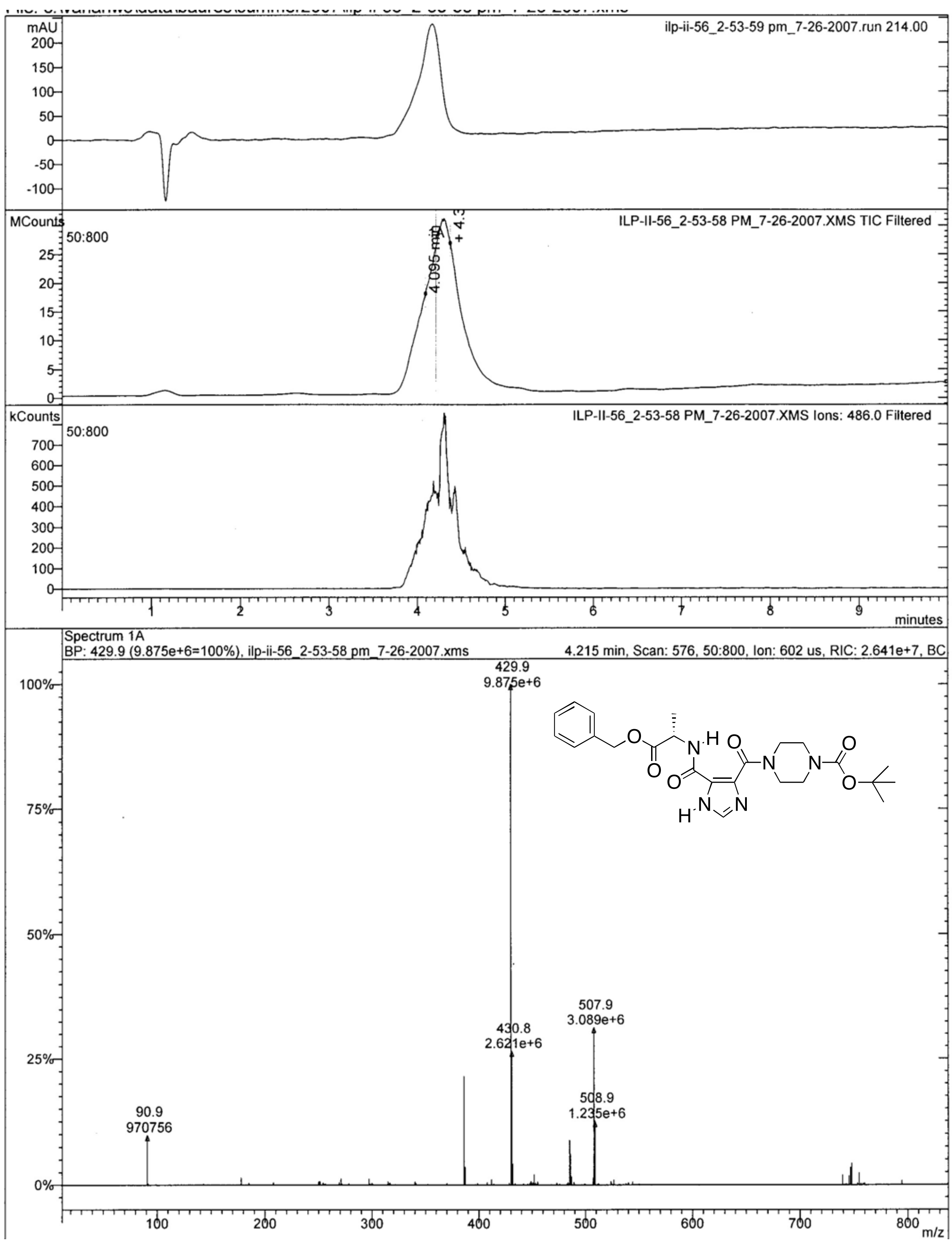


**Figure S53.** LC/MS data for **5{**53}.

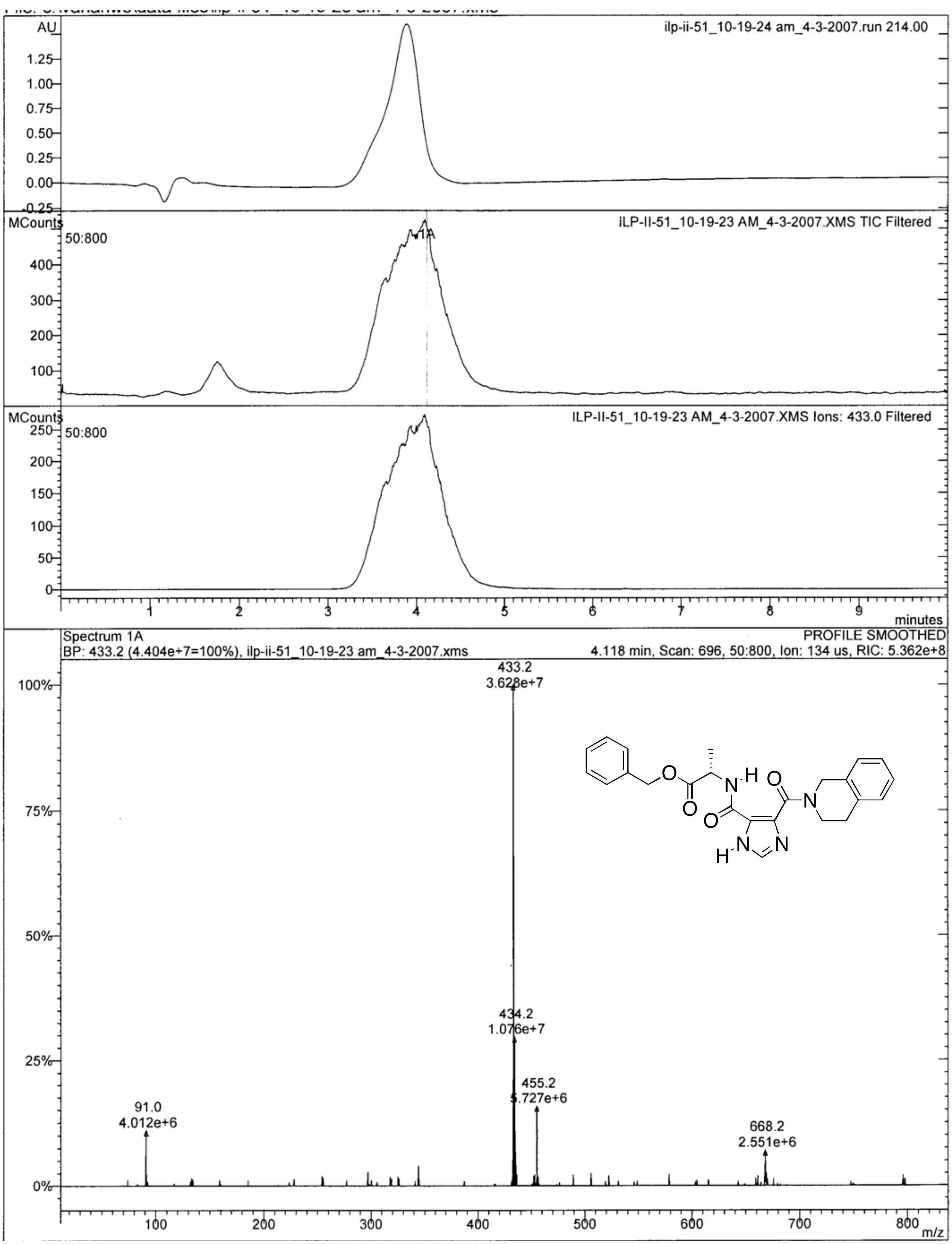


**Figure S54.** LC/MS data for **5{**54}.

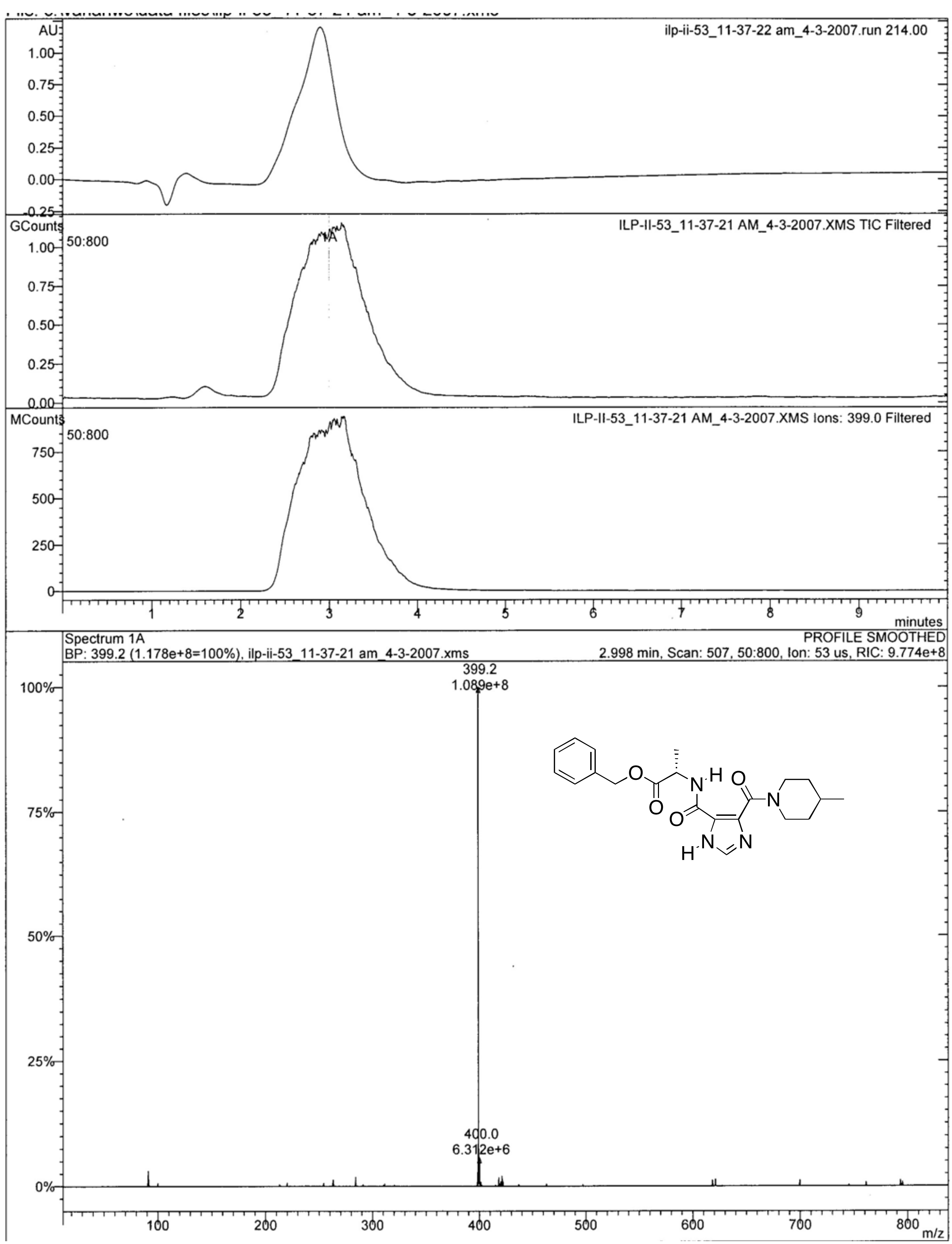


**Figure S55.** LC/MS data for **5{**55}.

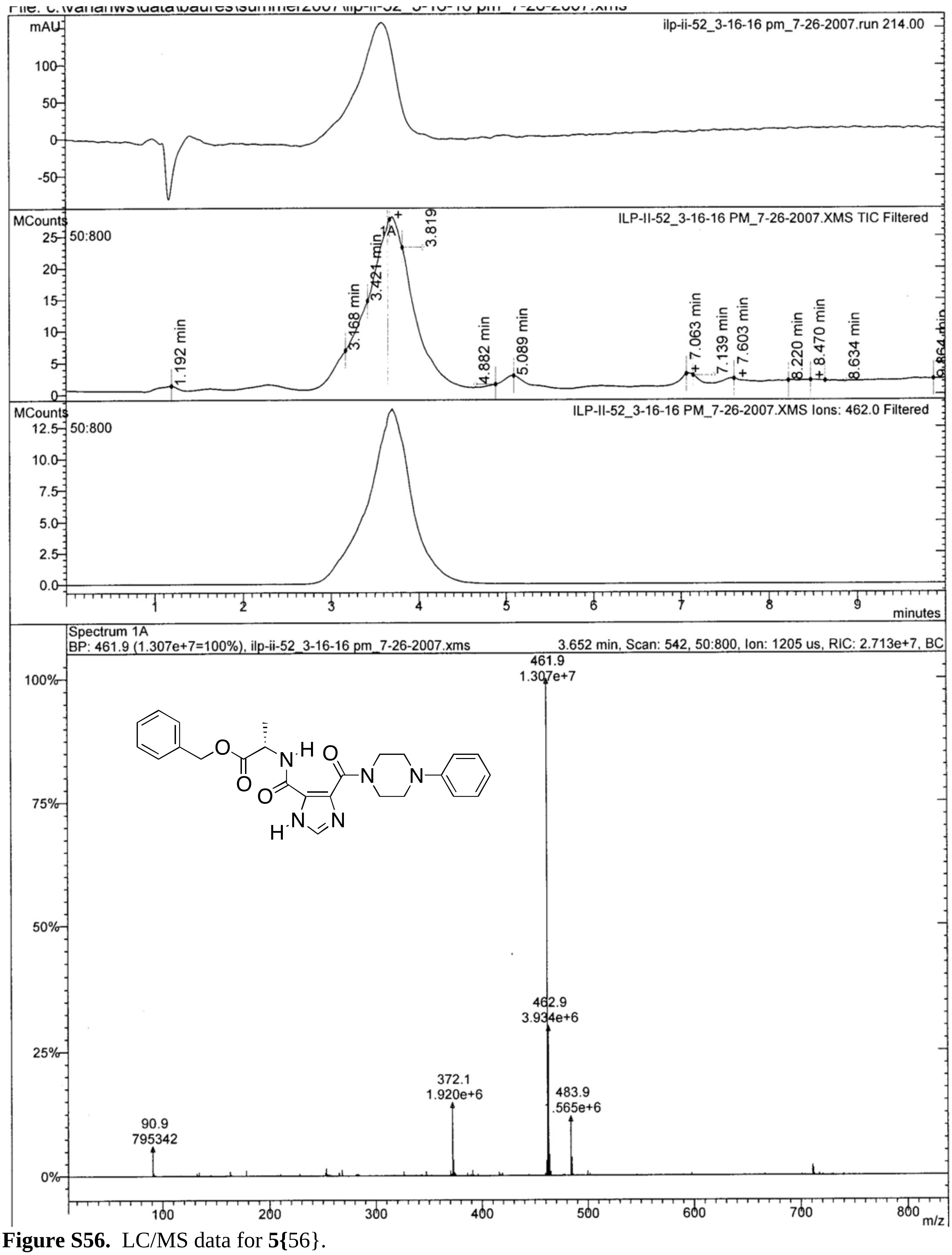


**Figure S56.** LC/MS data for **5{**56}.

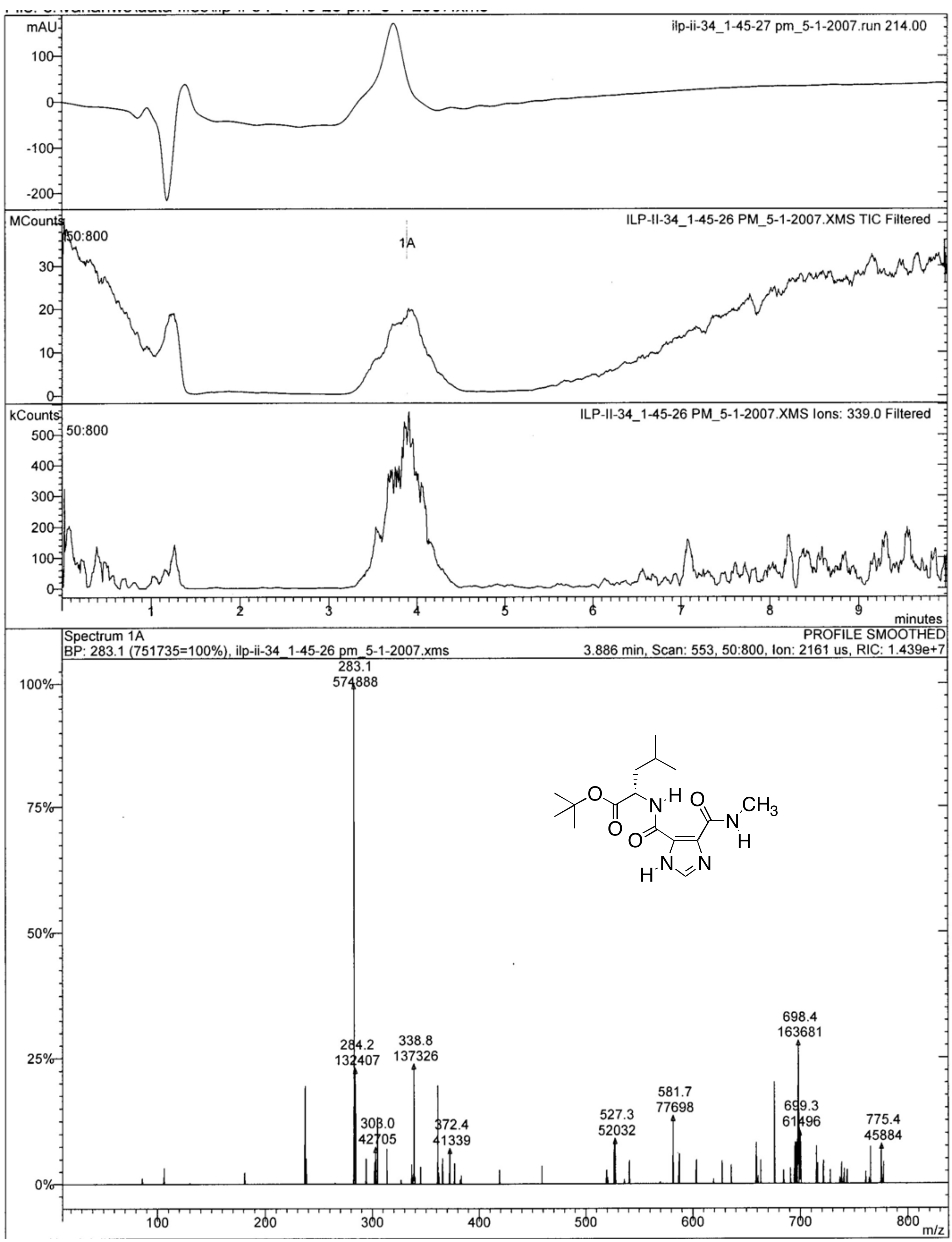


**Figure S57.** LC/MS data for **5{**57}.

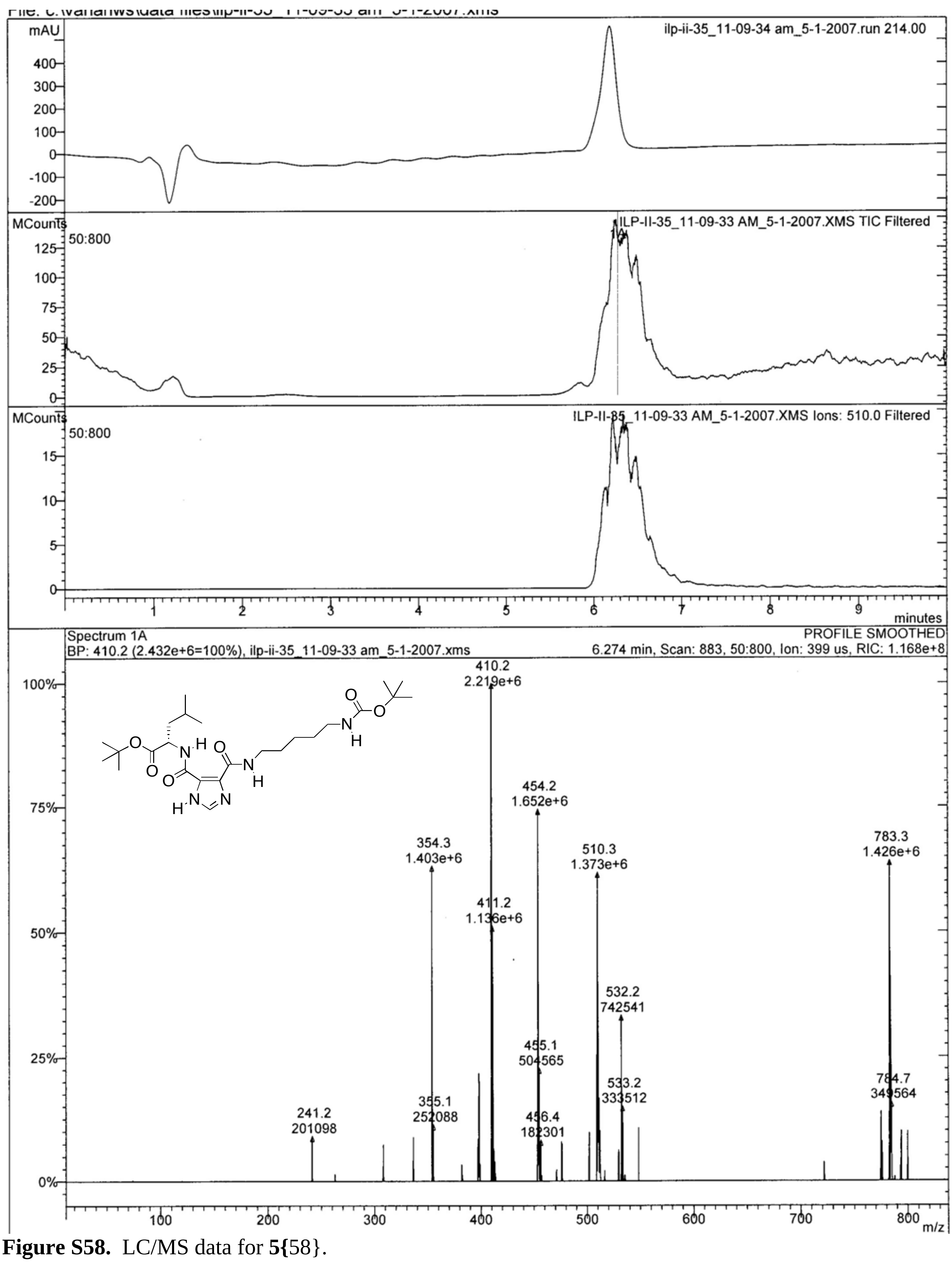


**Figure S58.** LC/MS data for **5{**58}.

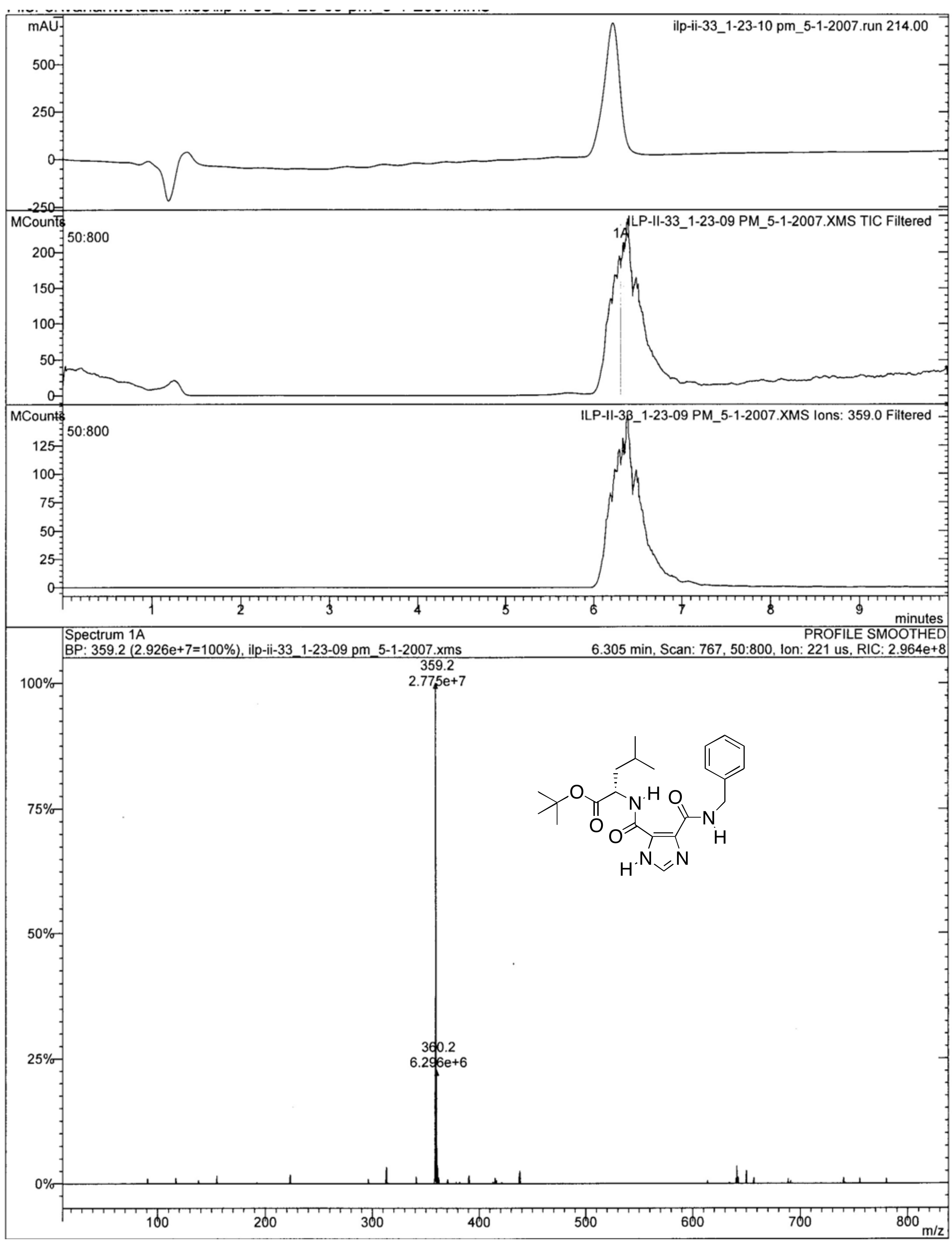


**Figure S59.** LC/MS data for **5{**59}.

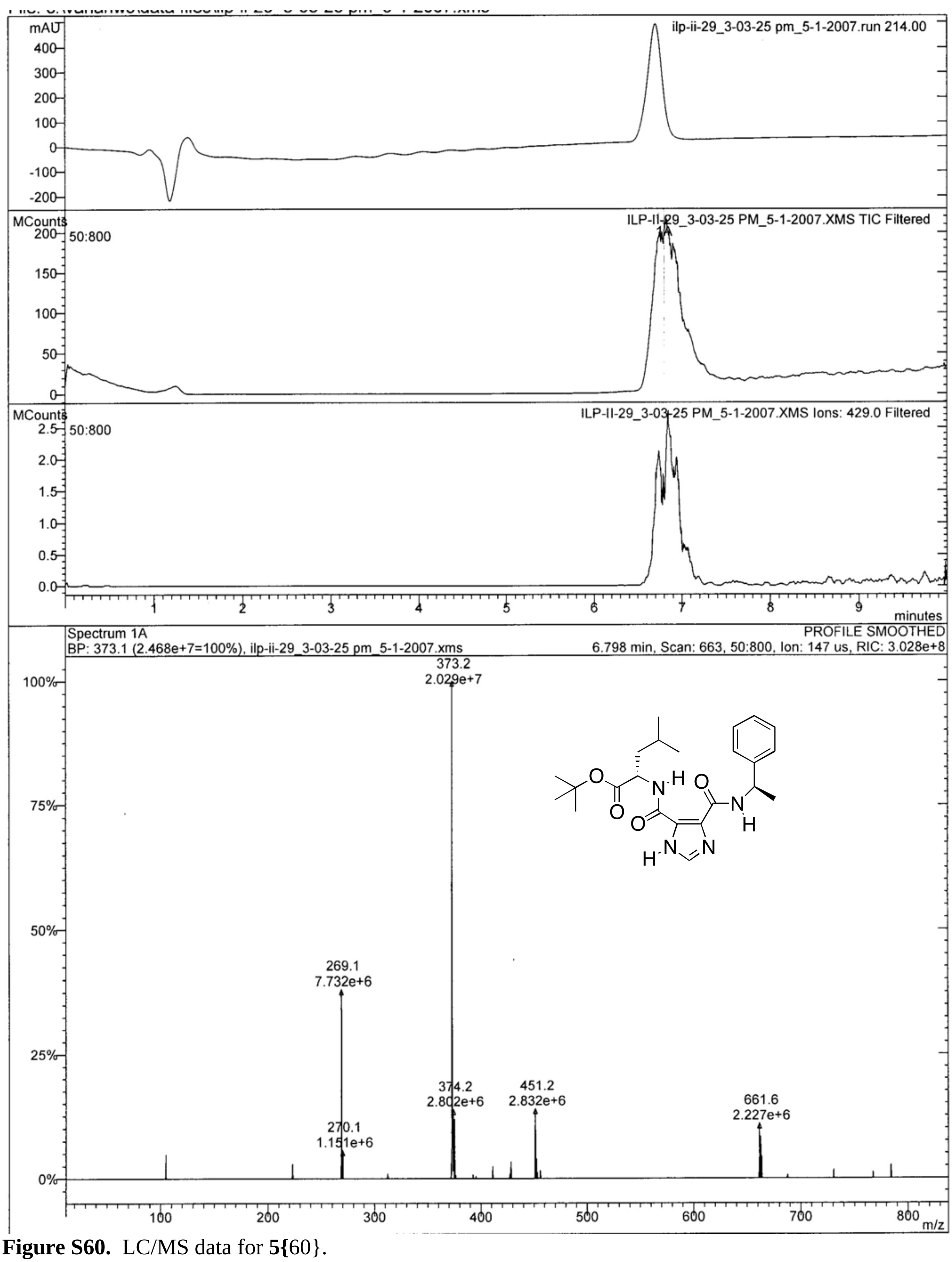


**Figure S60.** LC/MS data for **5{**60}.

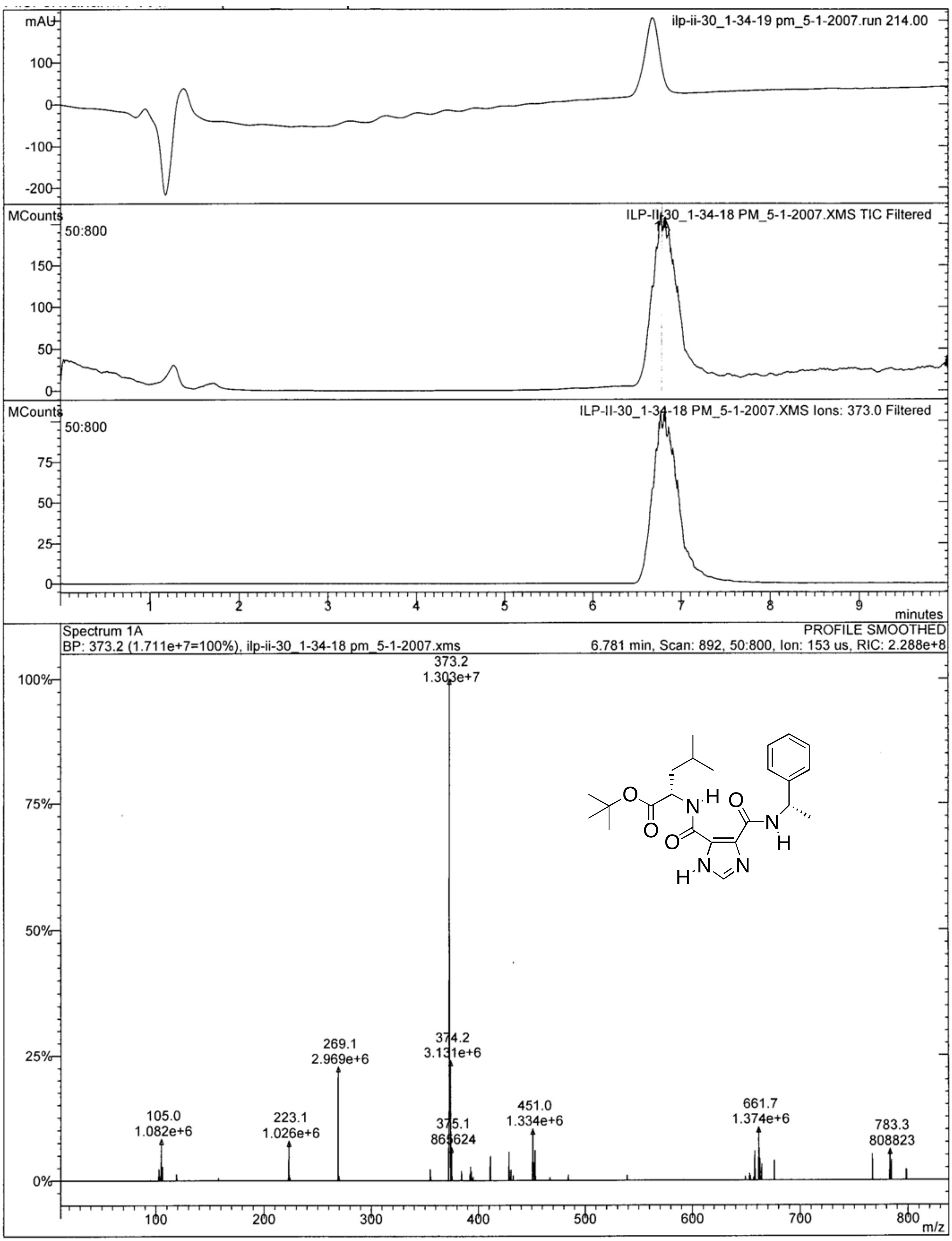


**Figure S61.** LC/MS data for **5{**61}.

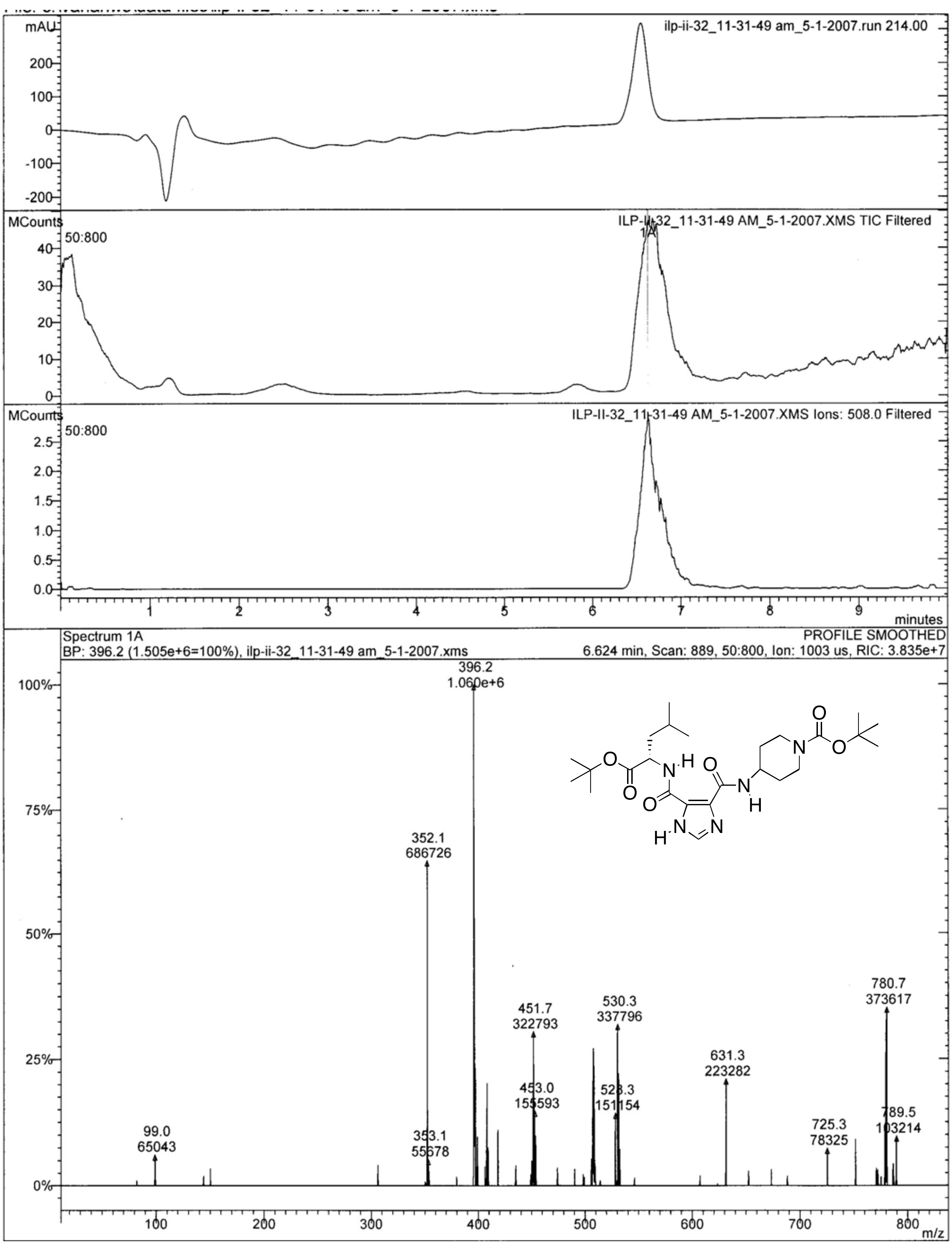


**Figure S62.** LC/MS data for **5{**62}.

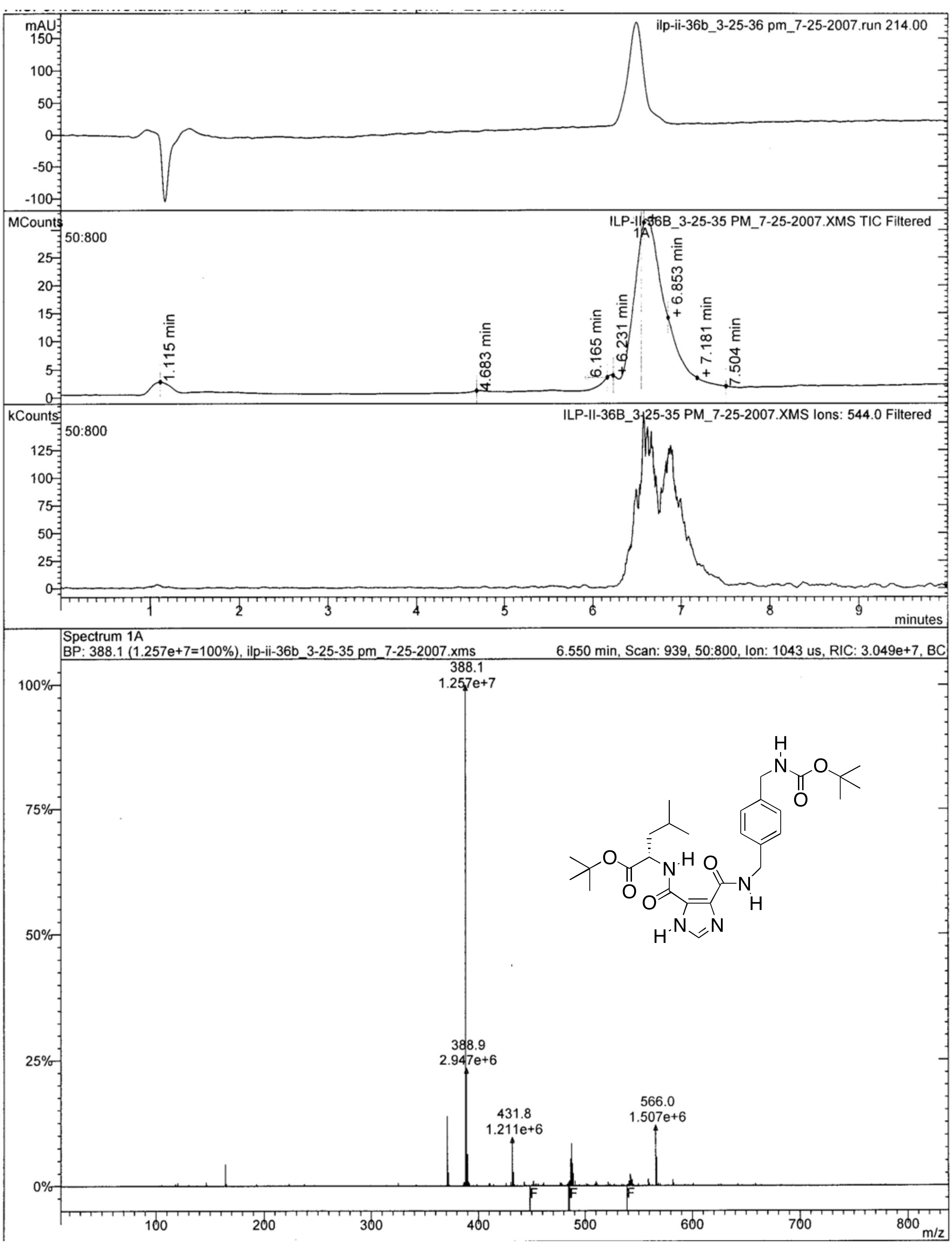


**Figure S63.** LC/MS data for **5{**63}.

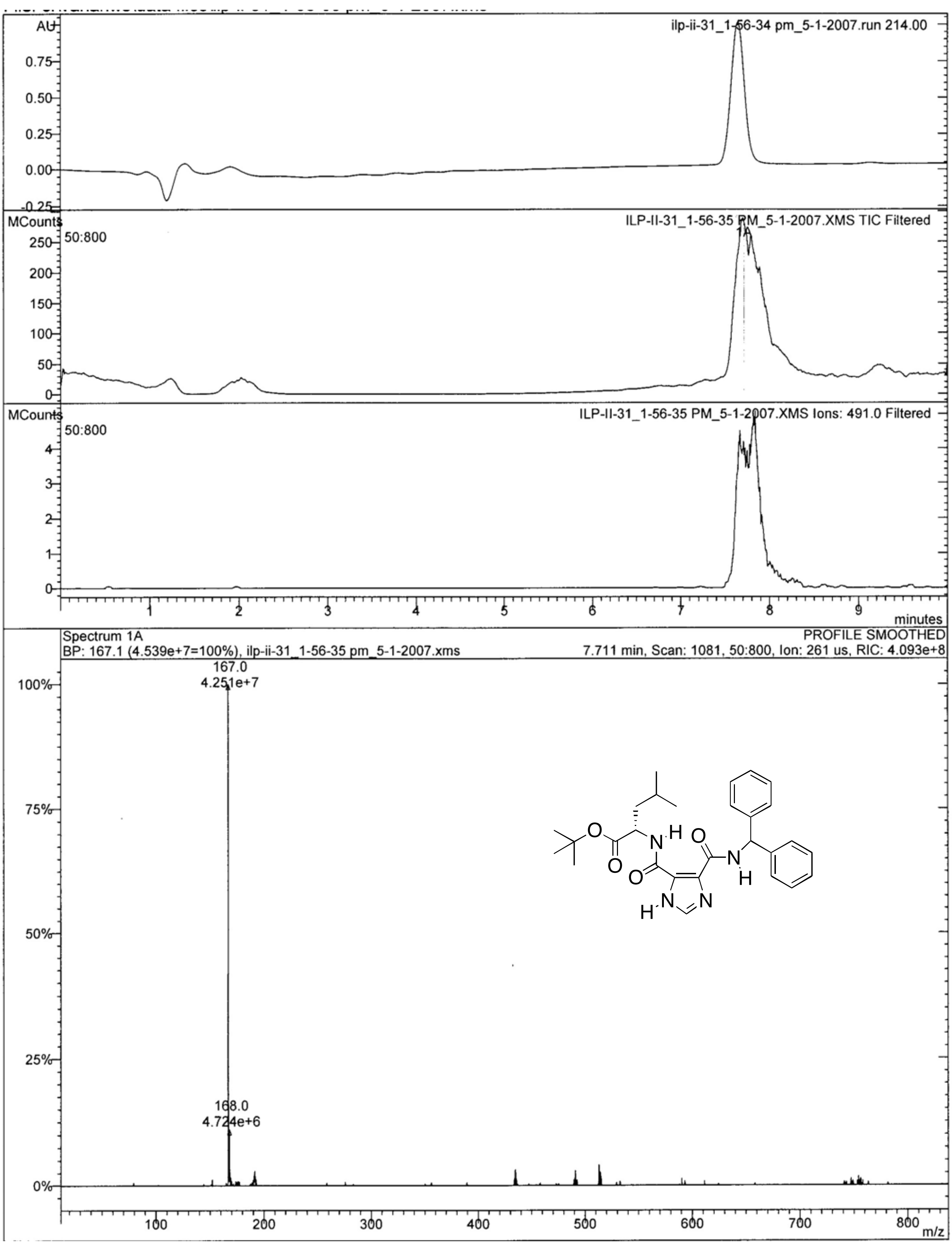


**Figure S64.** LC/MS data for **5{**64}.

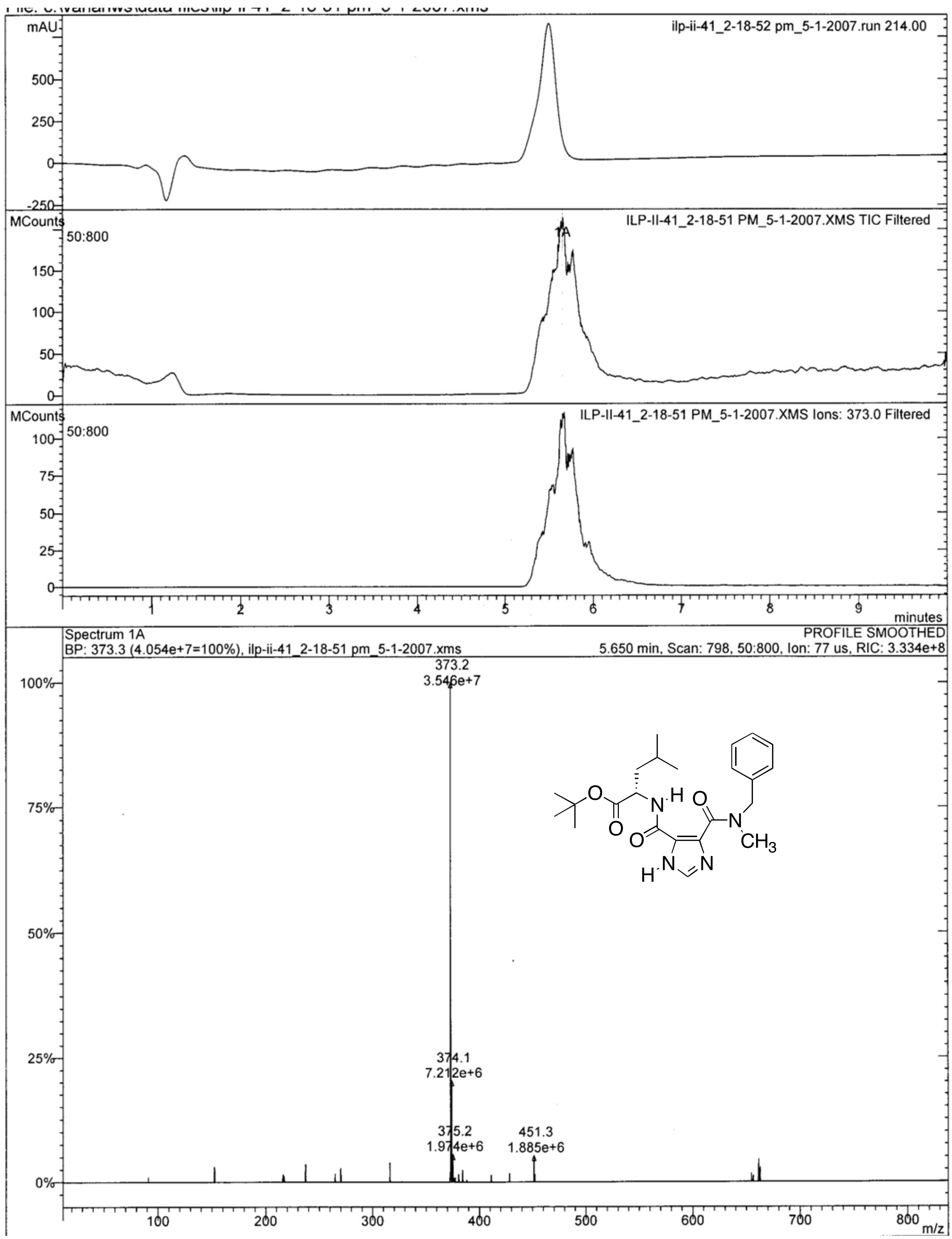


**Figure S65.** LC/MS data for **5{**65}.

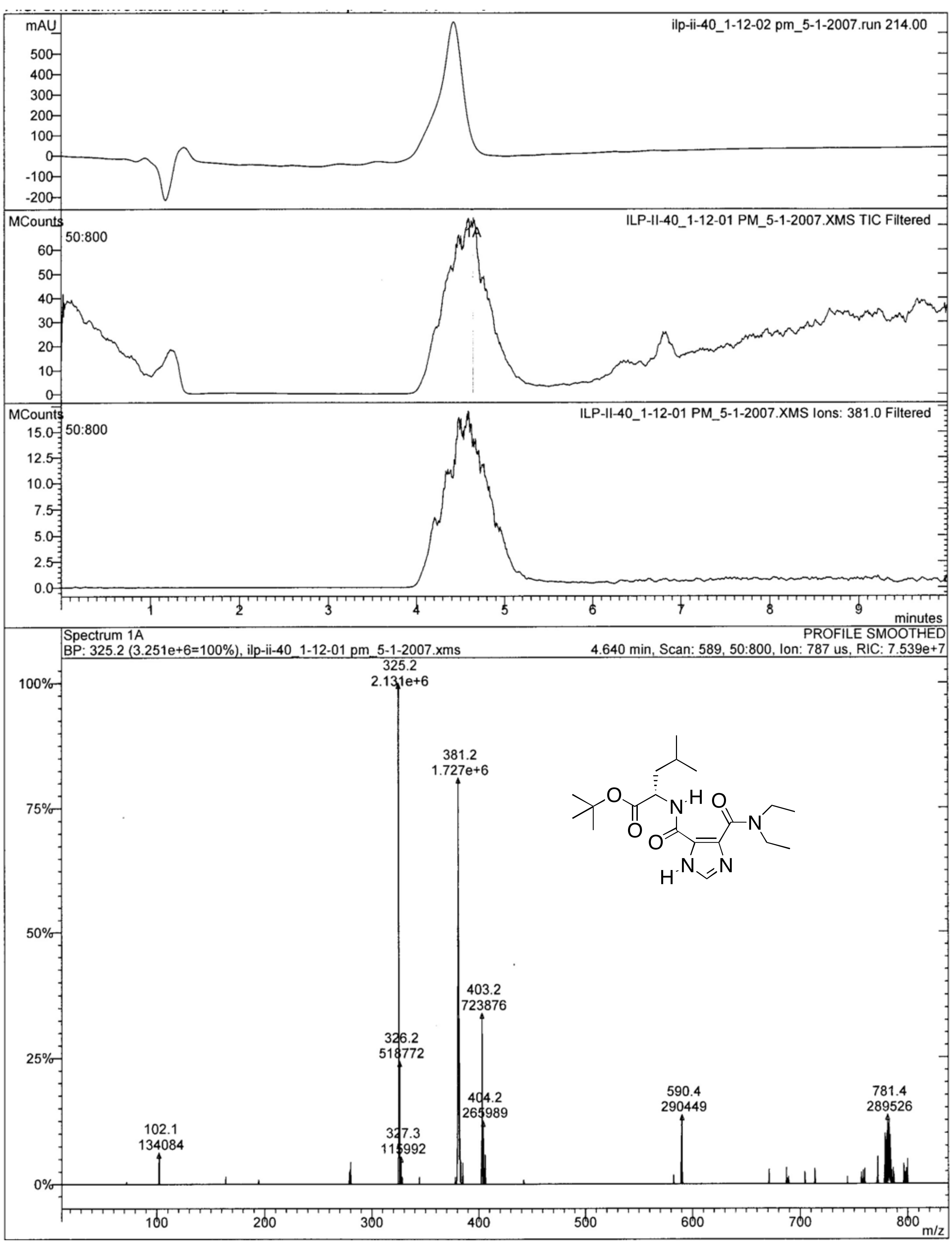


**Figure S66.** LC/MS data for **5{**66}.

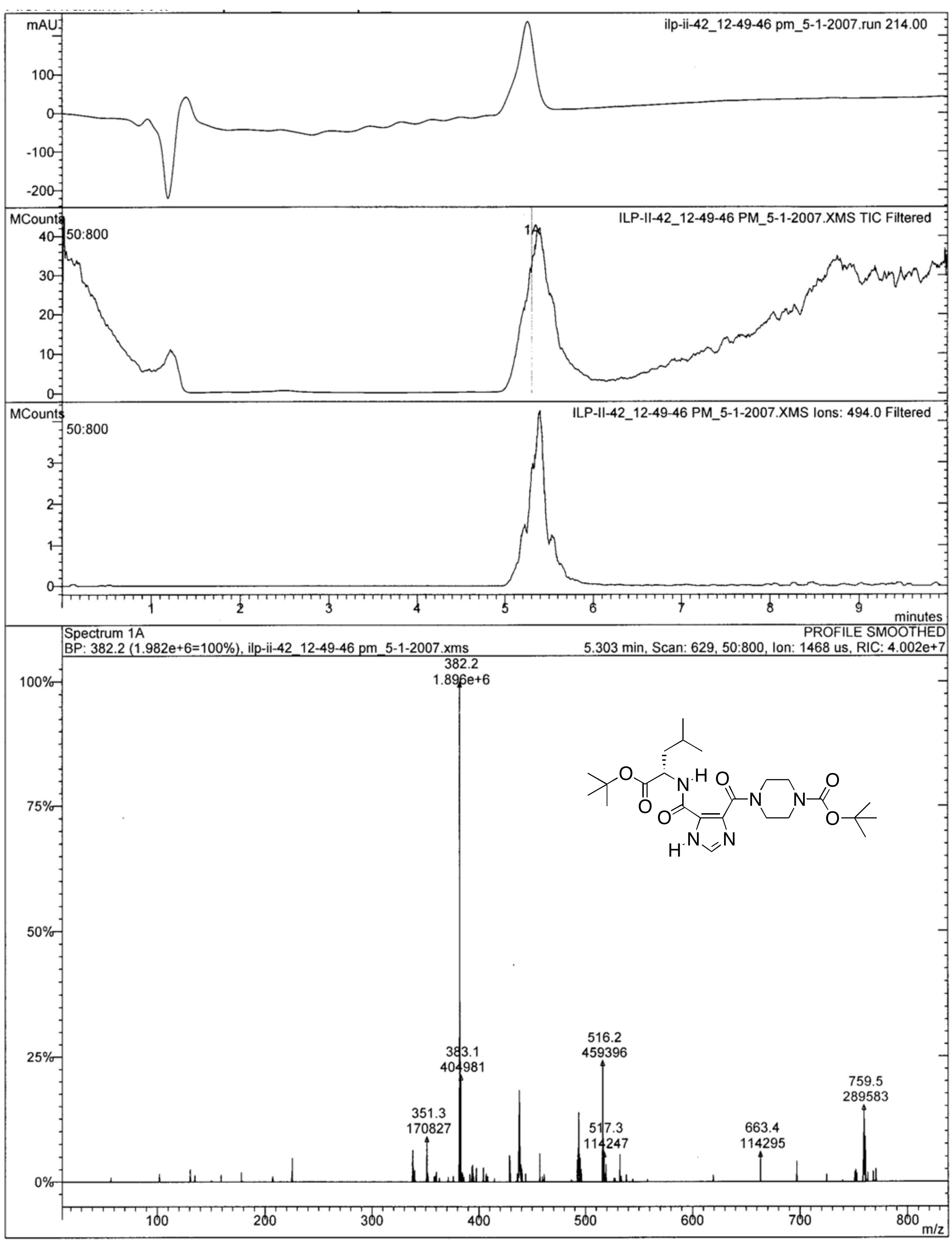


**Figure S67.** LC/MS data for **5{**67}.

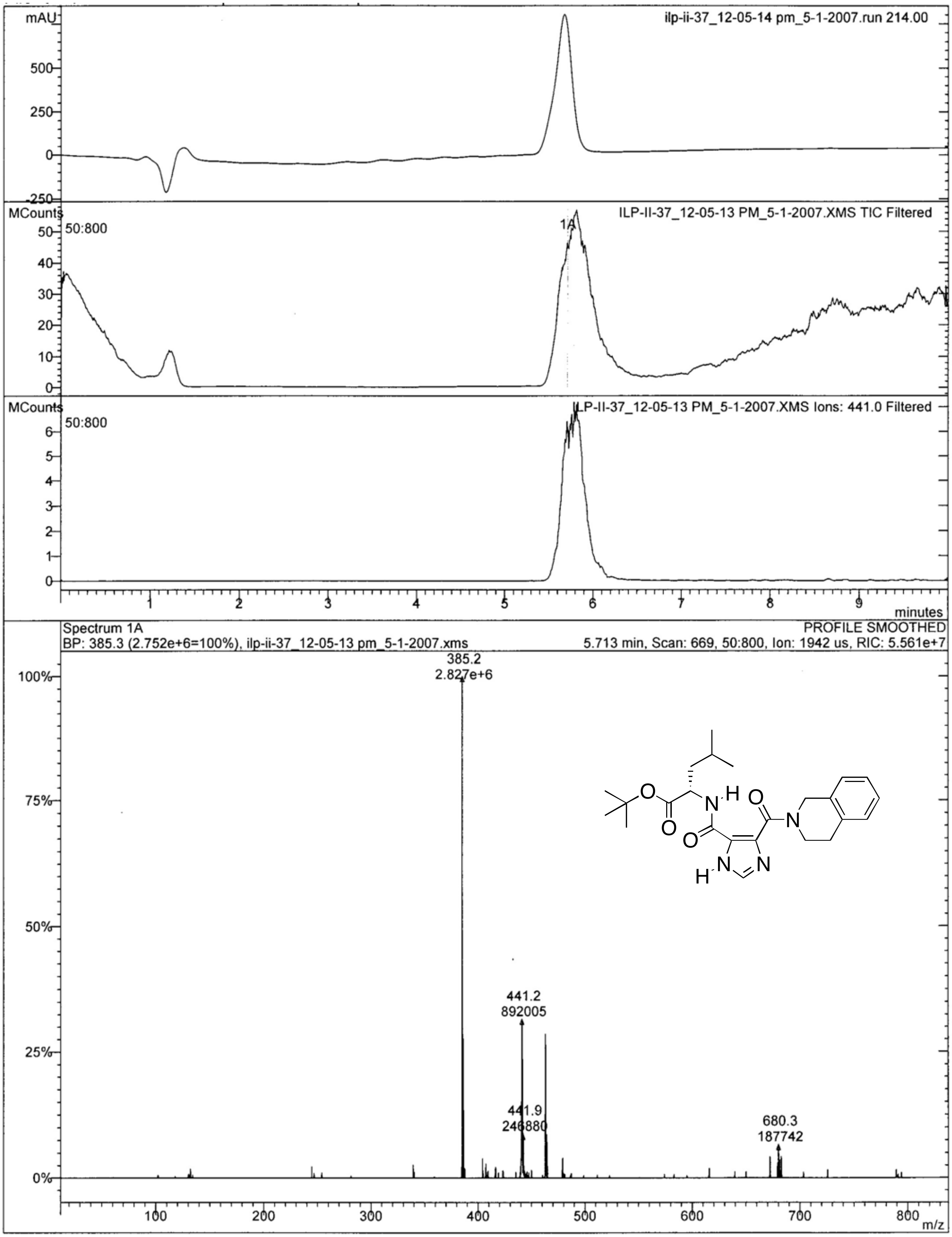


**Figure S68.** LC/MS data for **5{**68}.

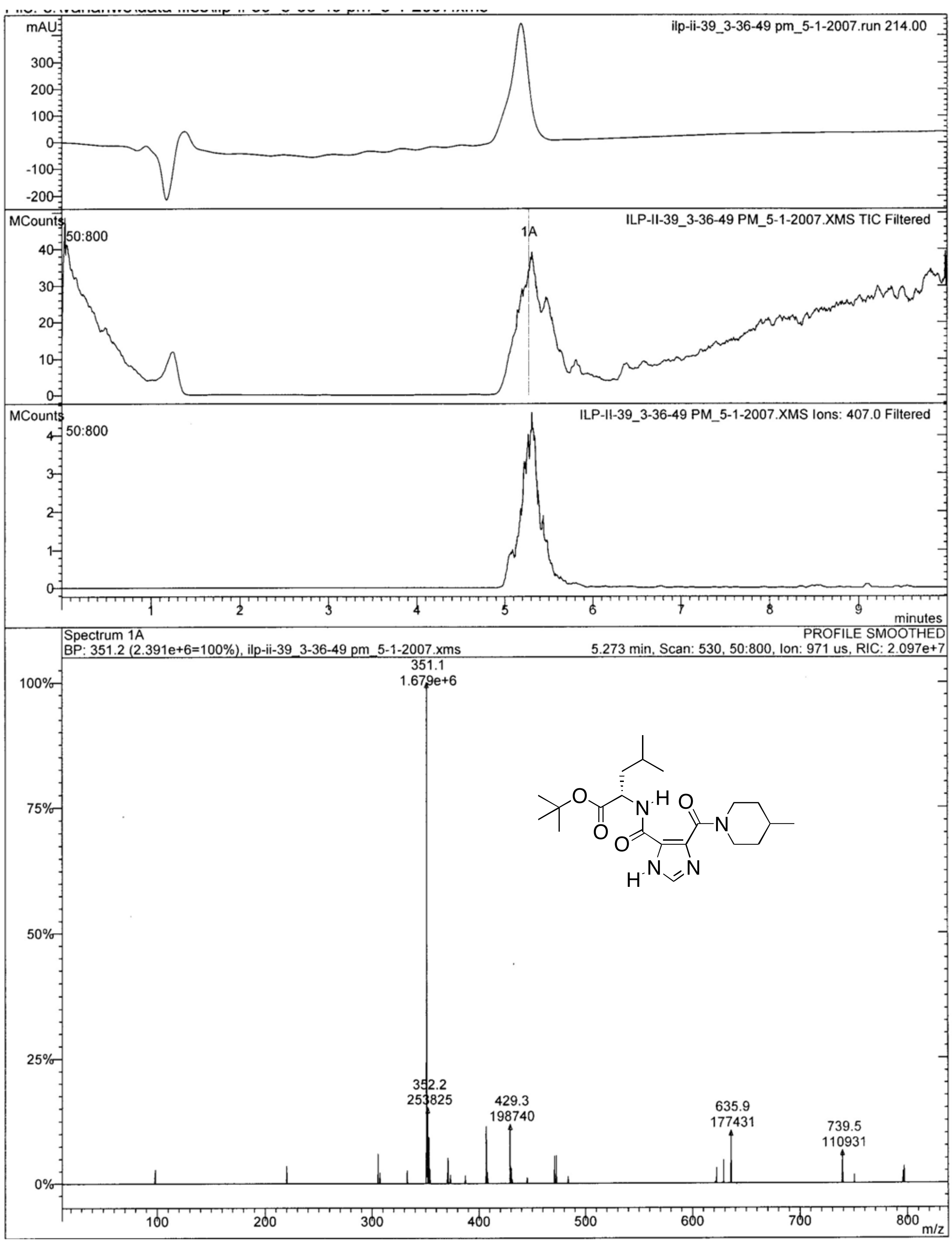


**Figure S69.** LC/MS data for **5{**69}.

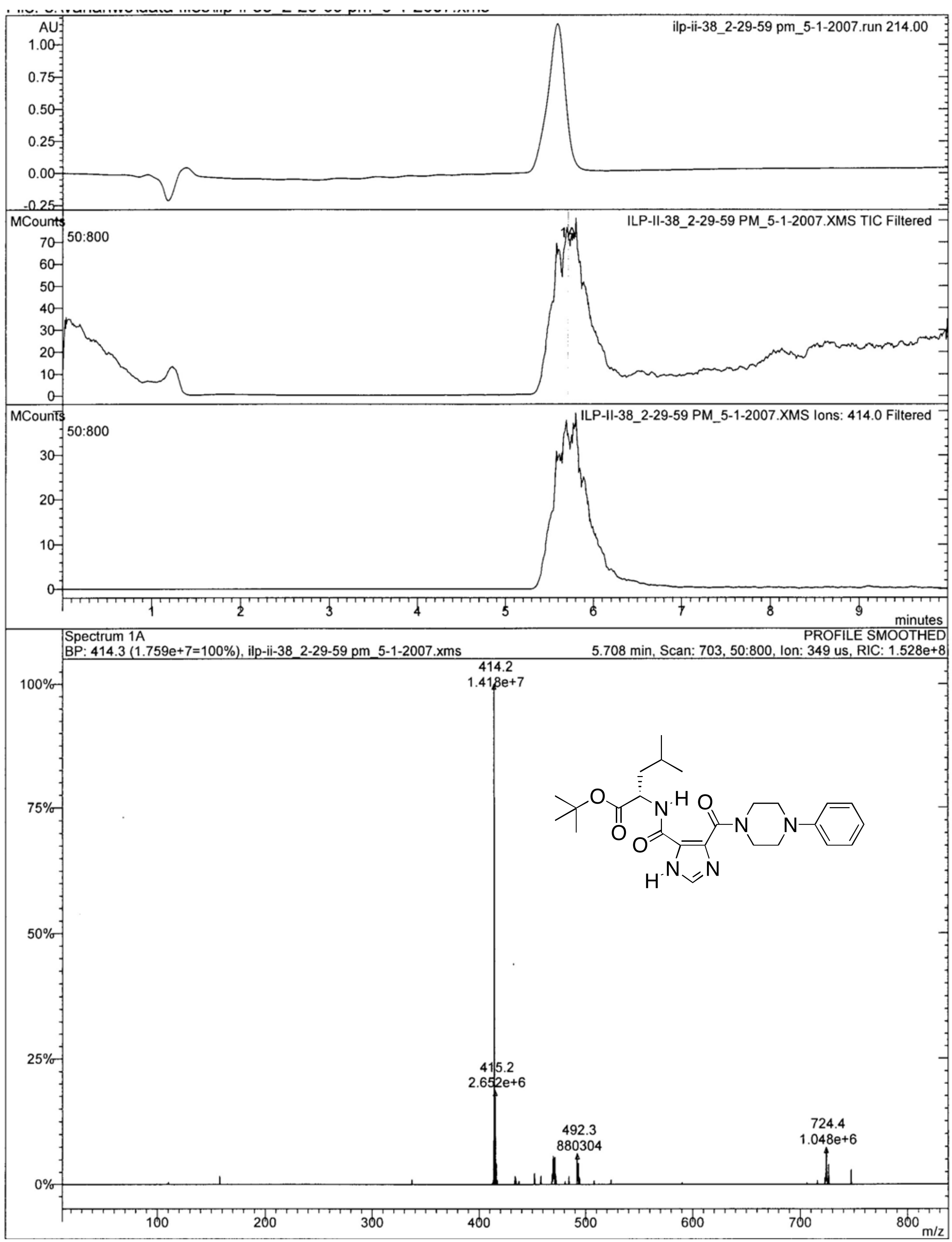


**Figure S70.** LC/MS data for **5{**70}.

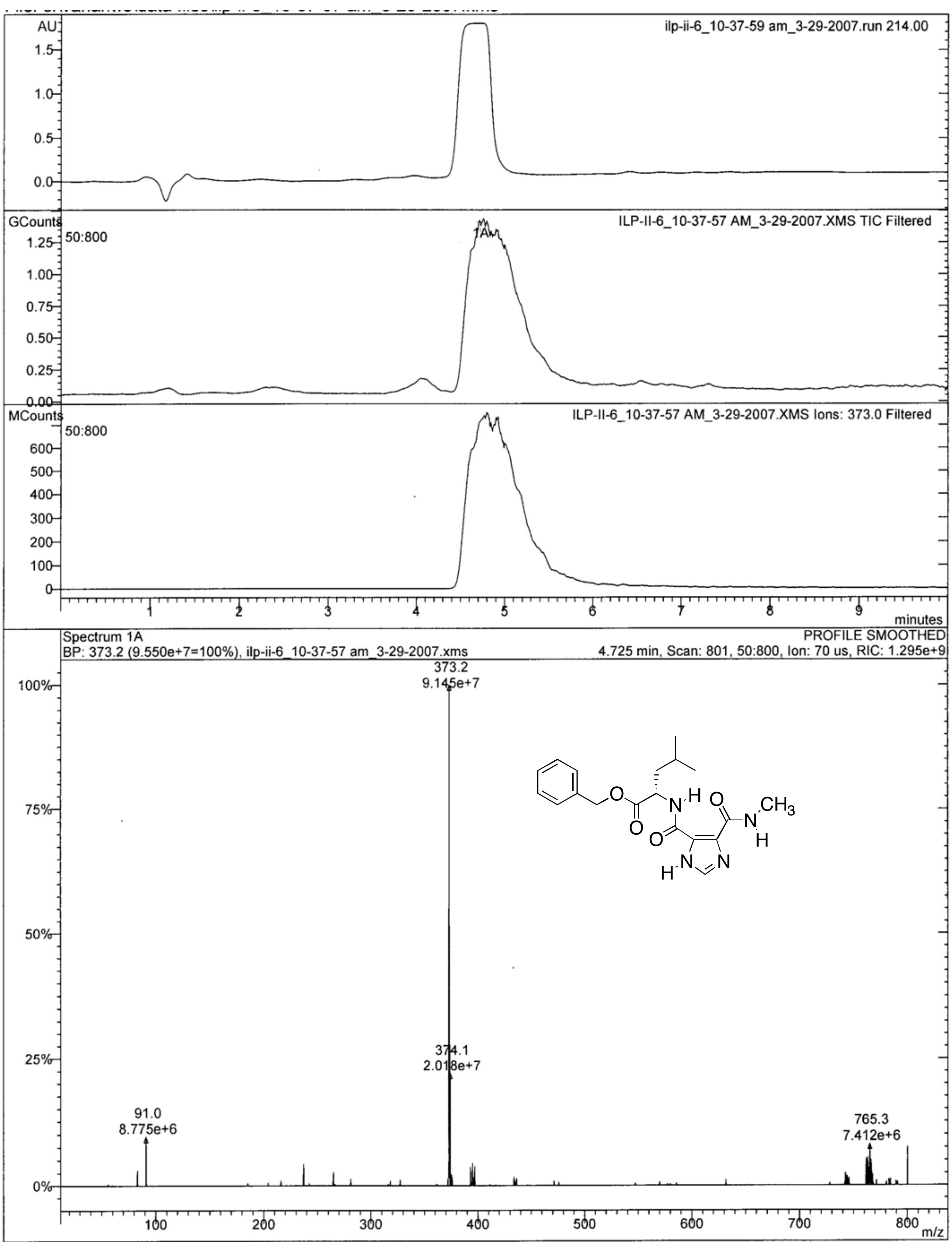


**Figure S71.** LC/MS data for **5{**71}.

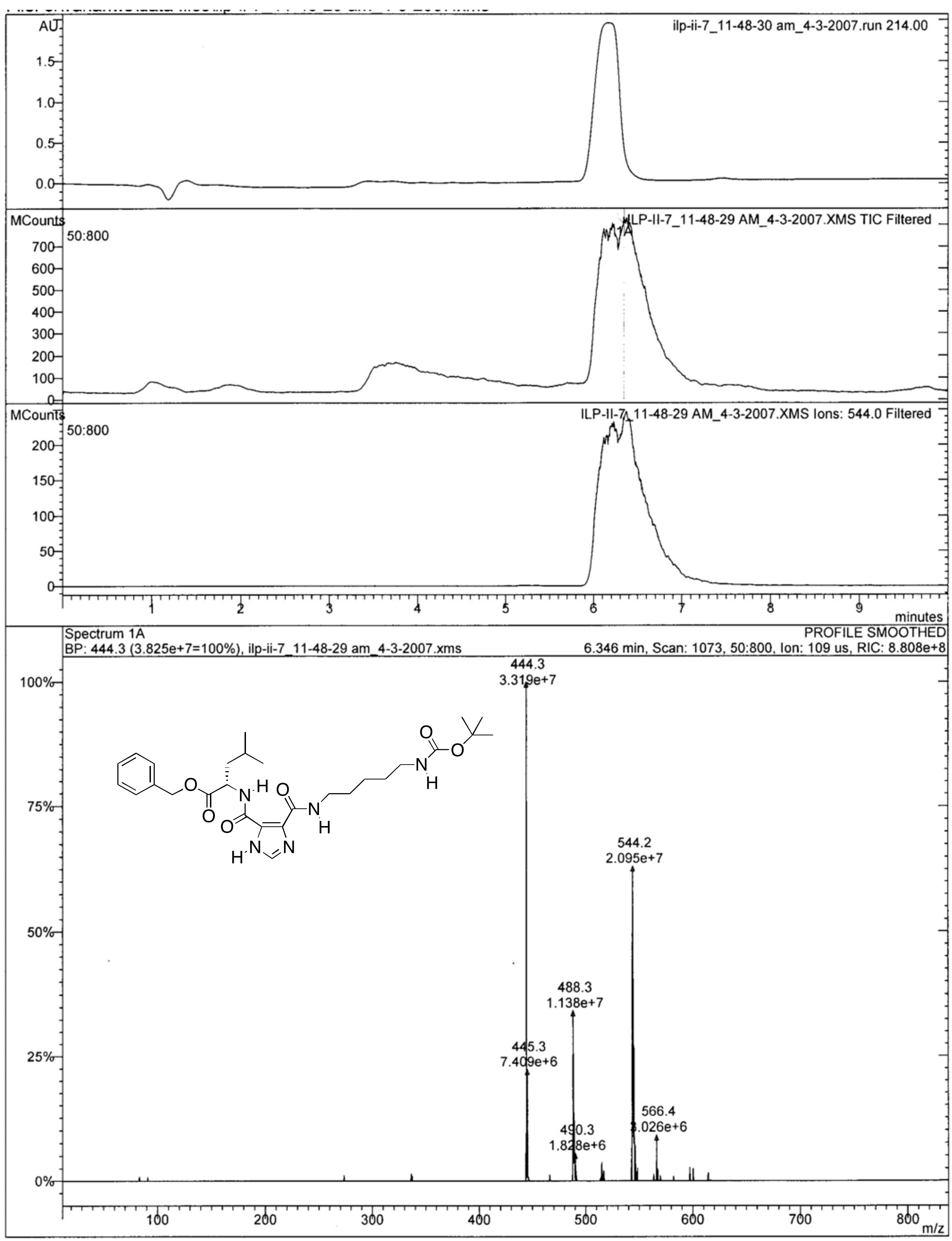


**Figure S72.** LC/MS data for **5{**72}.

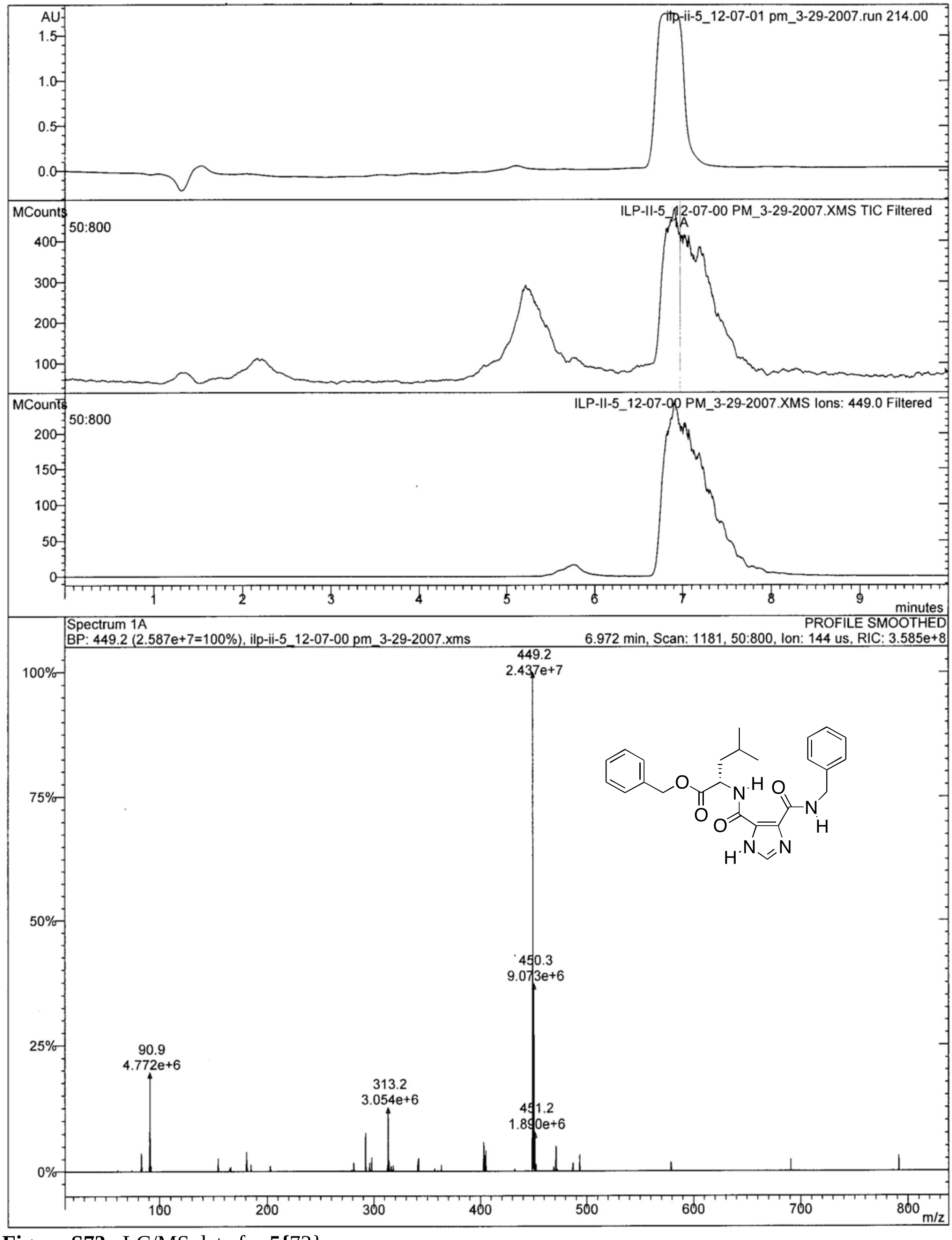


**Figure S73.** LC/MS data for **5{**73}.

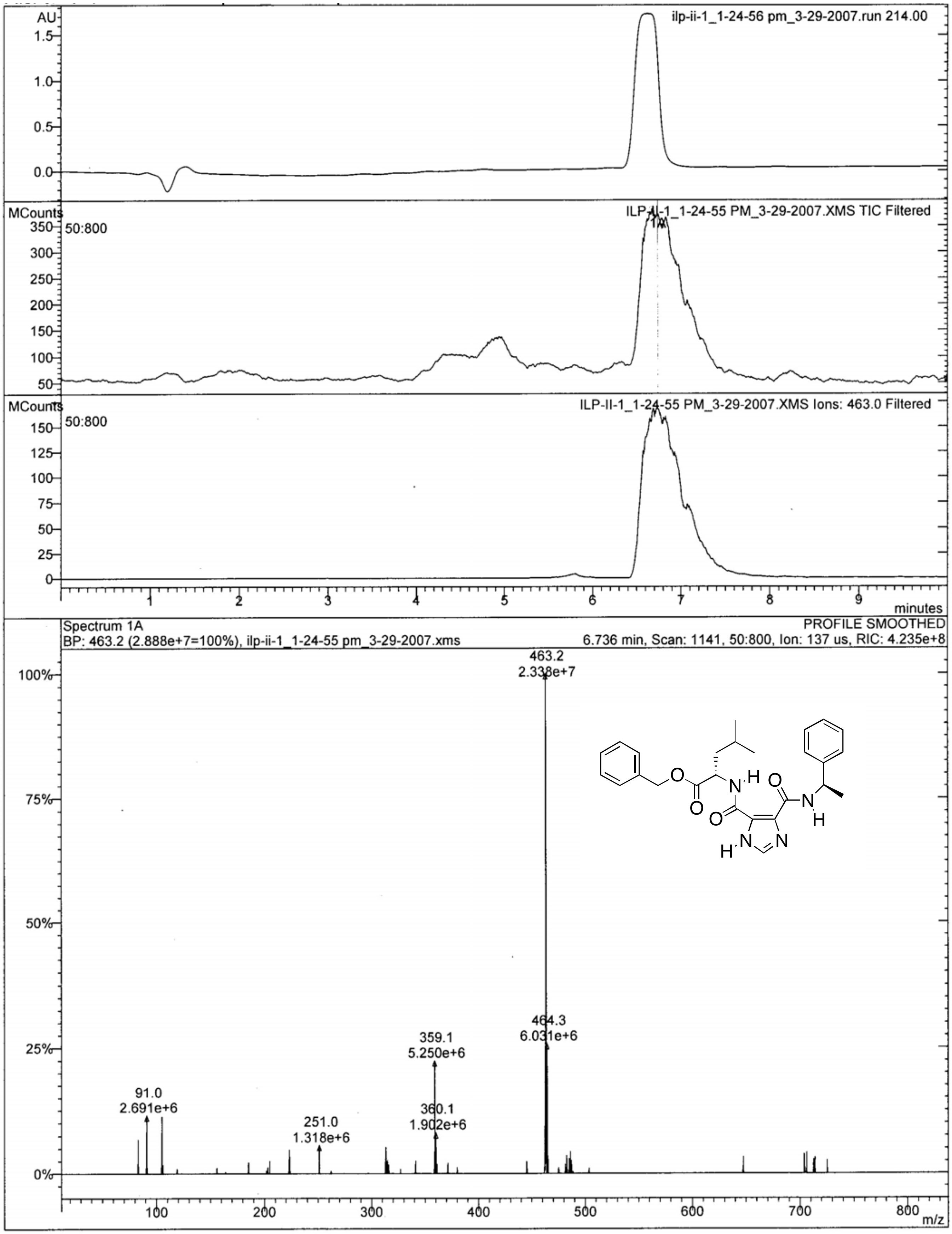


**Figure S74.** LC/MS data for **5{**74}.

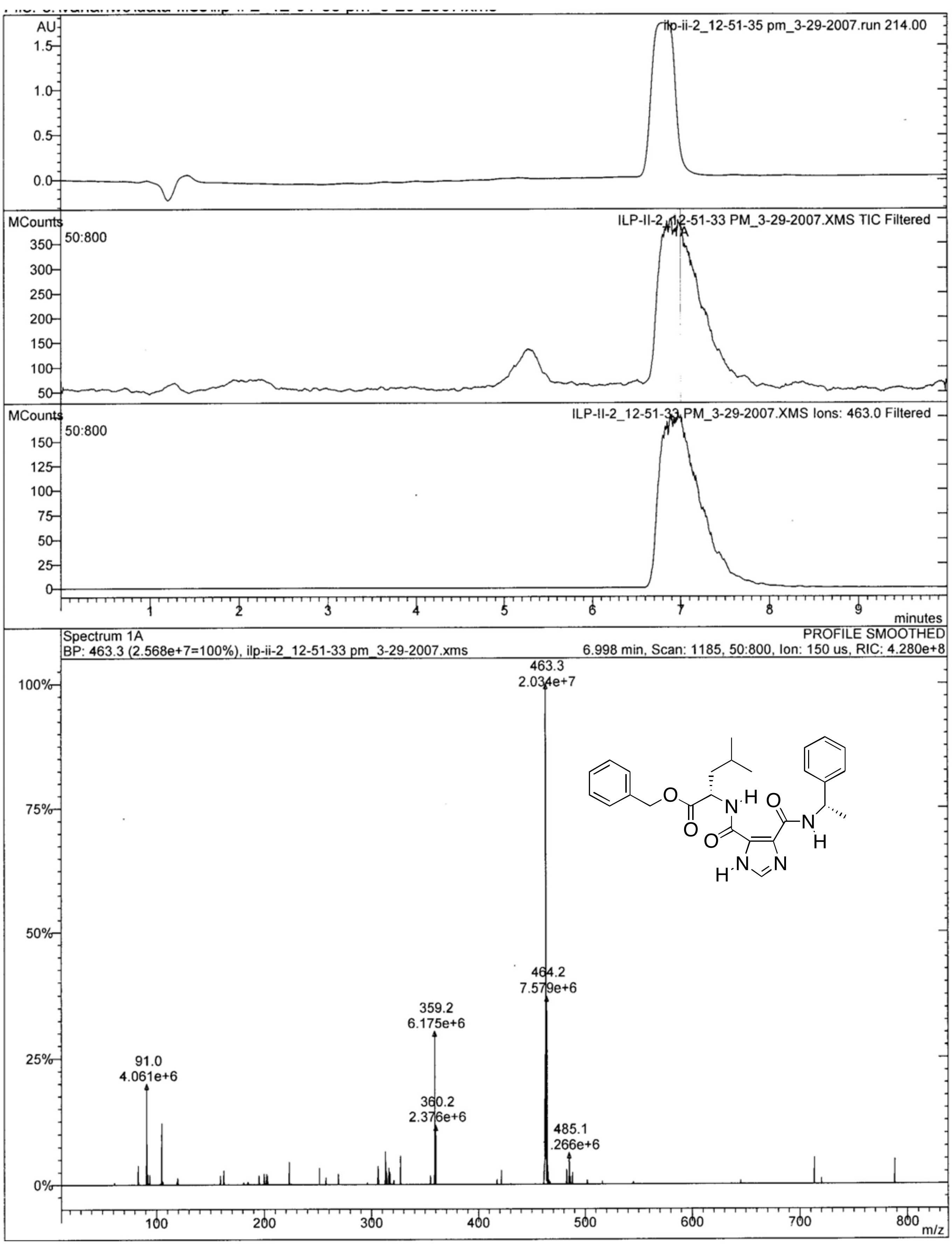


**Figure S75.** LC/MS data for **5{**75}.

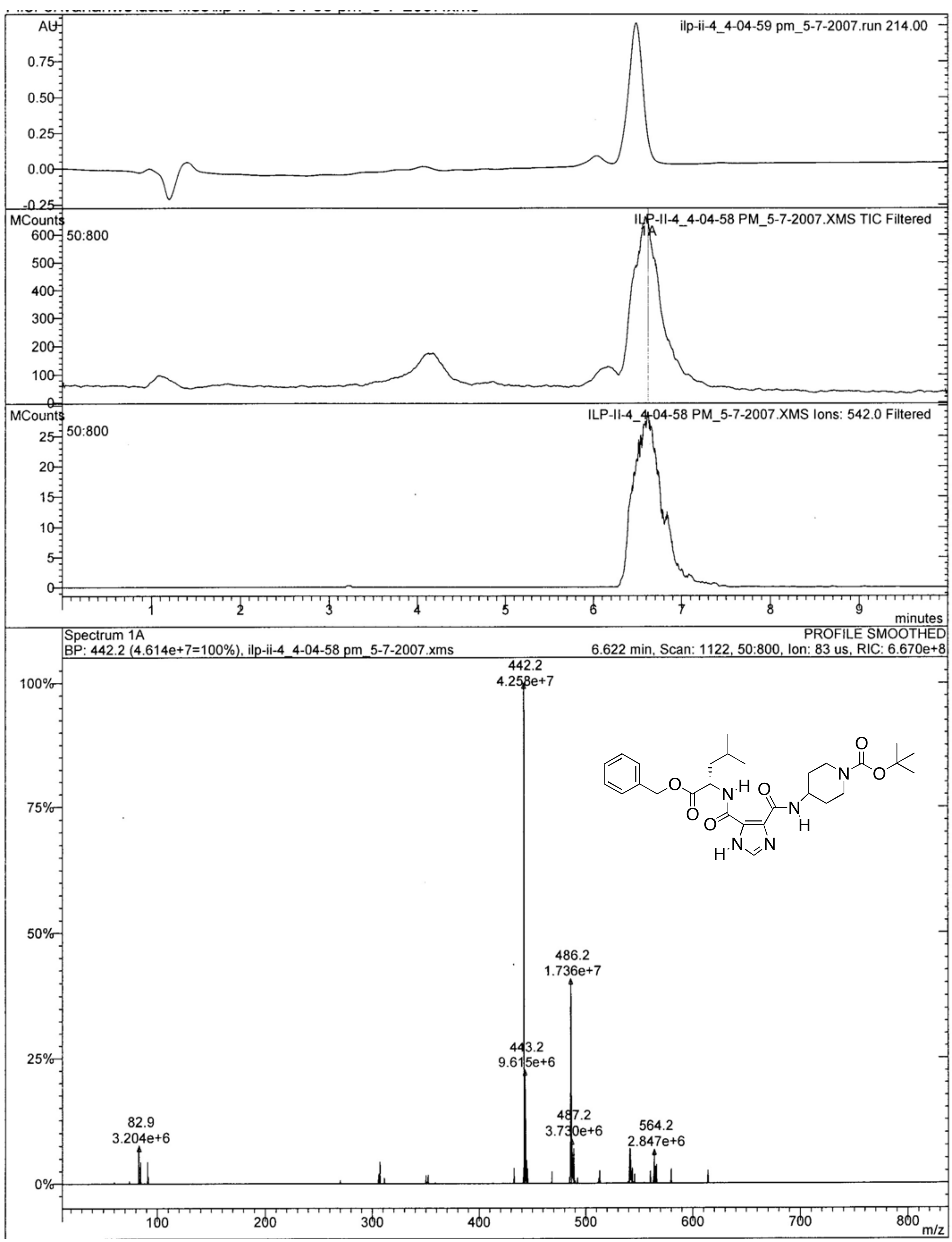


**Figure S76.** LC/MS data for **5{**76}.

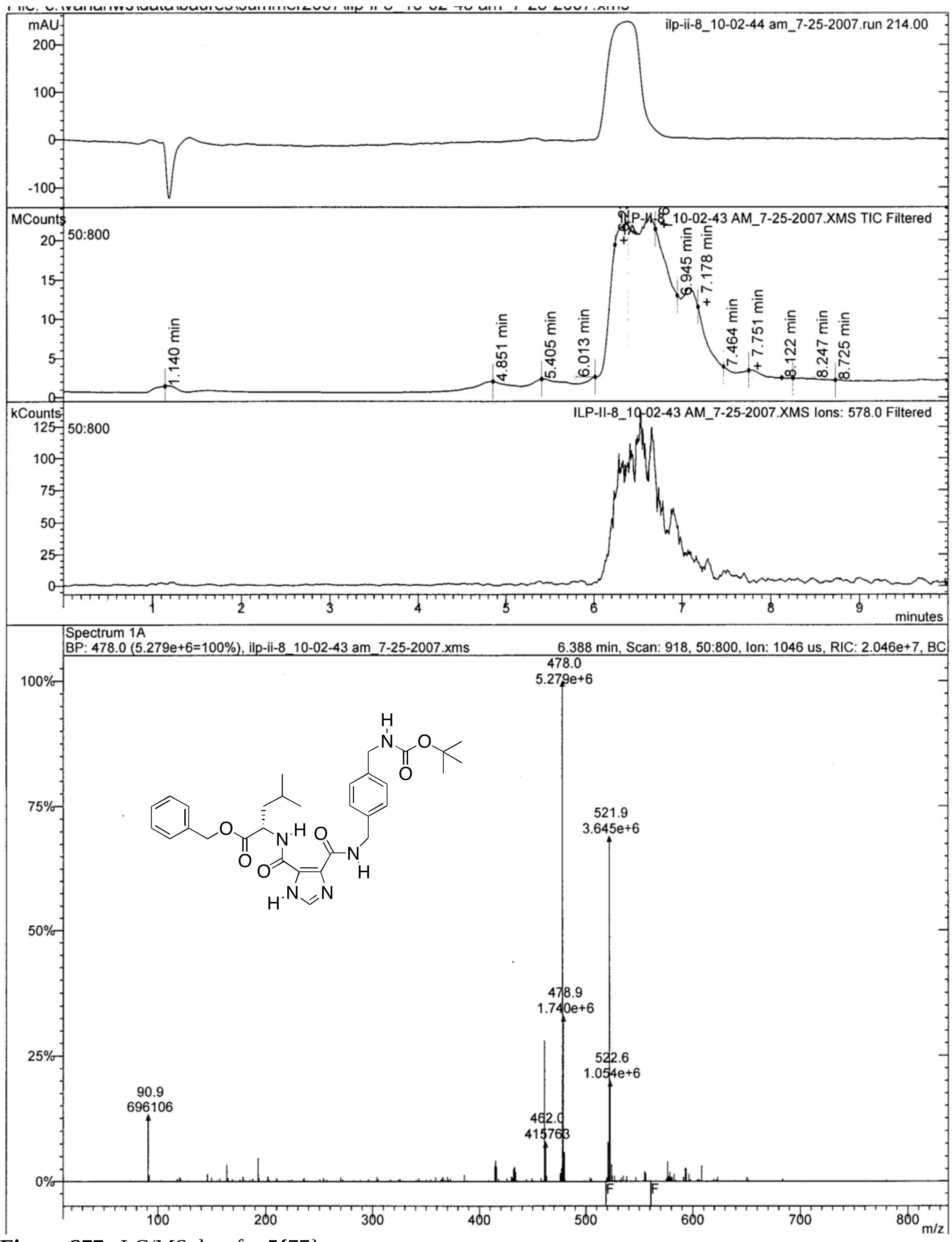


**Figure S77.** LC/MS data for **5{**77}.

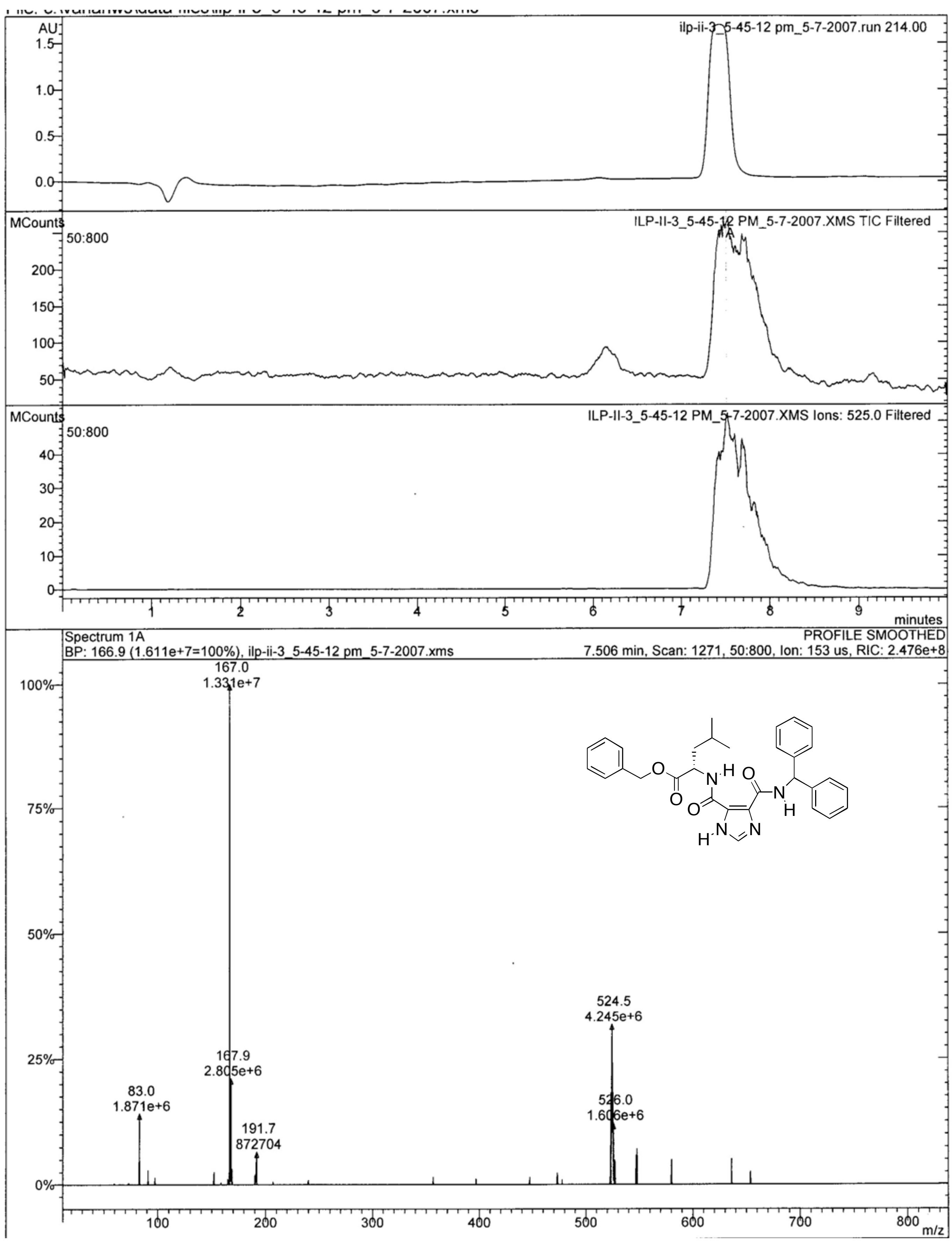


**Figure S78.** LC/MS data for **5{**78}.

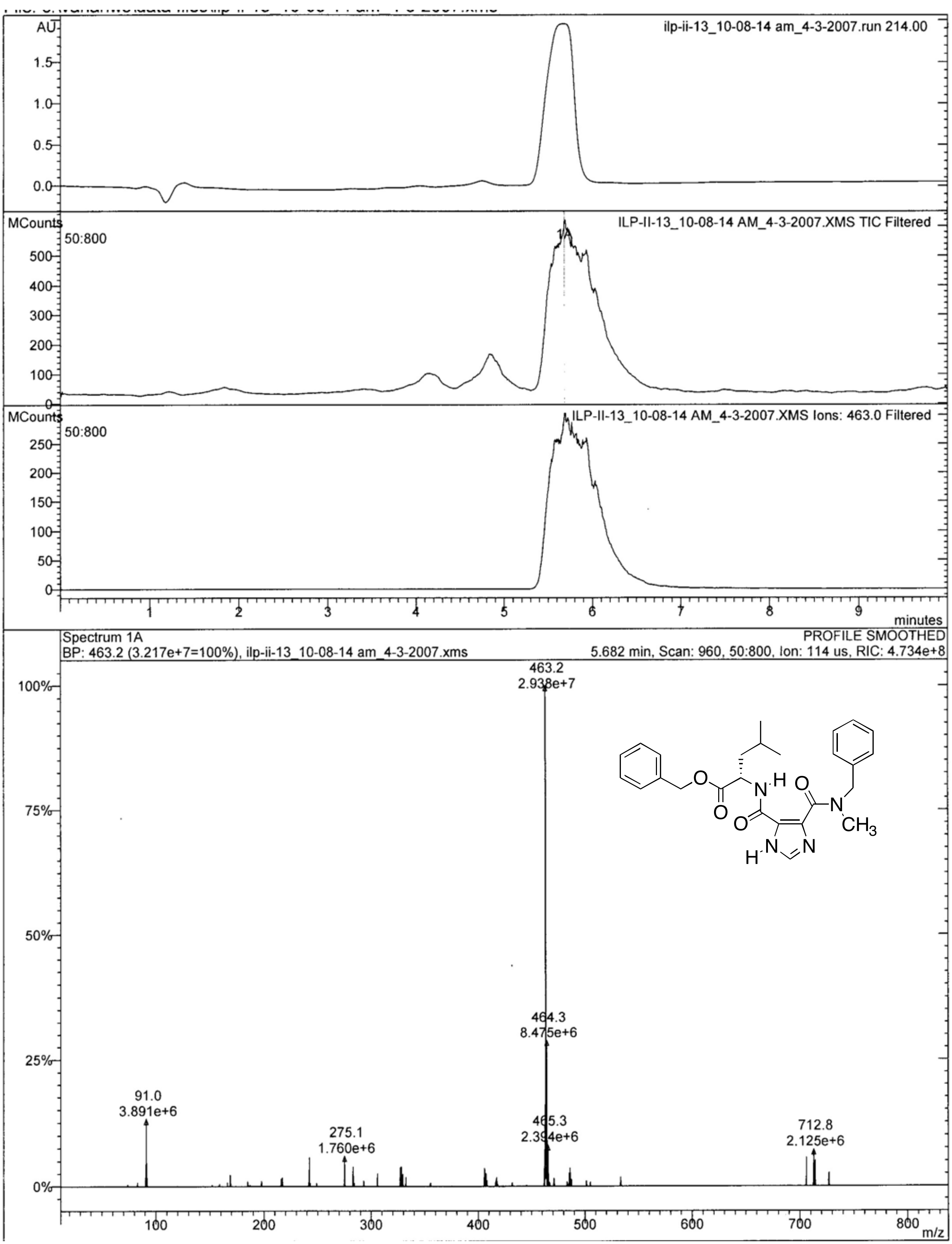


**Figure S79.** LC/MS data for **5{**79}.

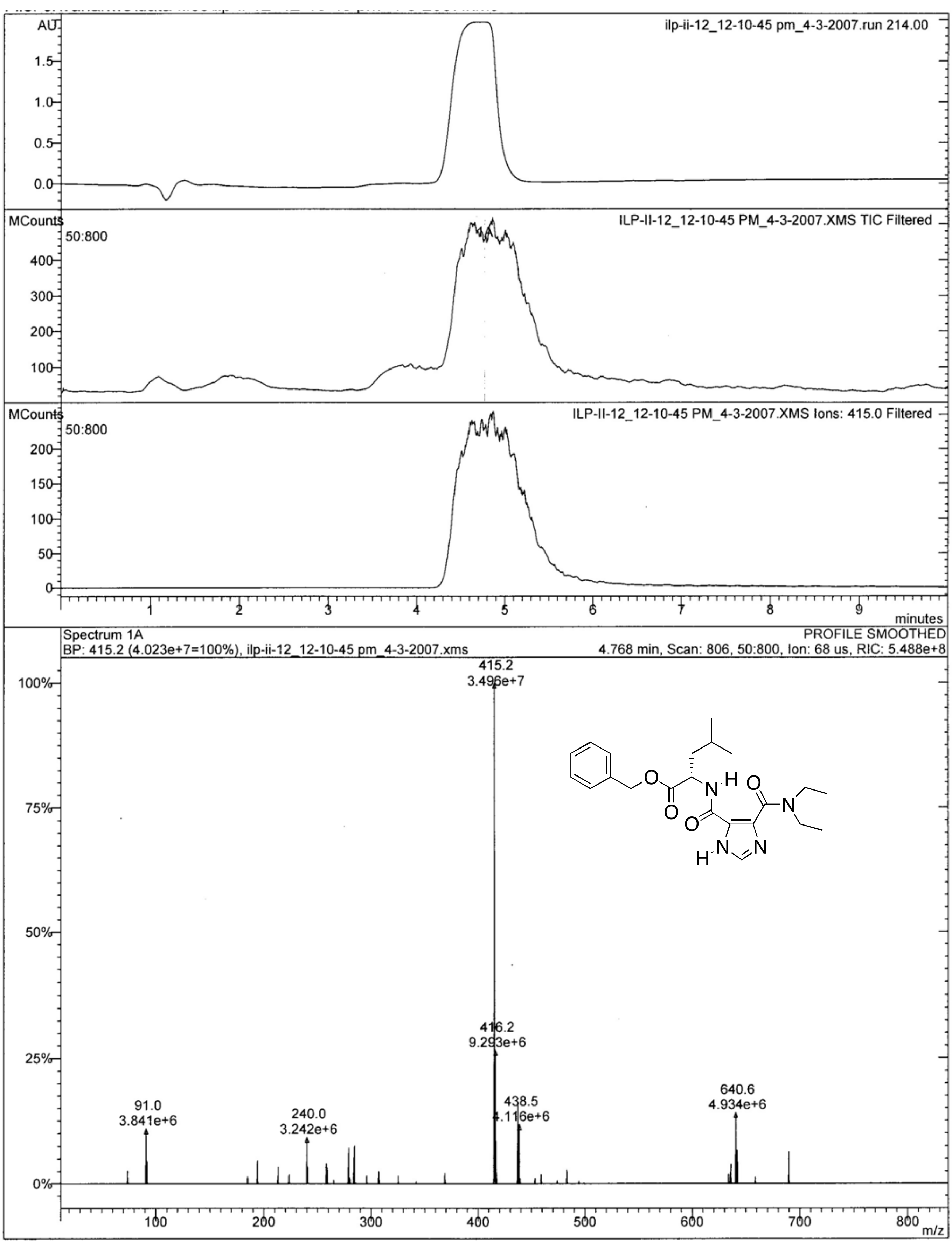


**Figure S80.** LC/MS data for **5{**80}.

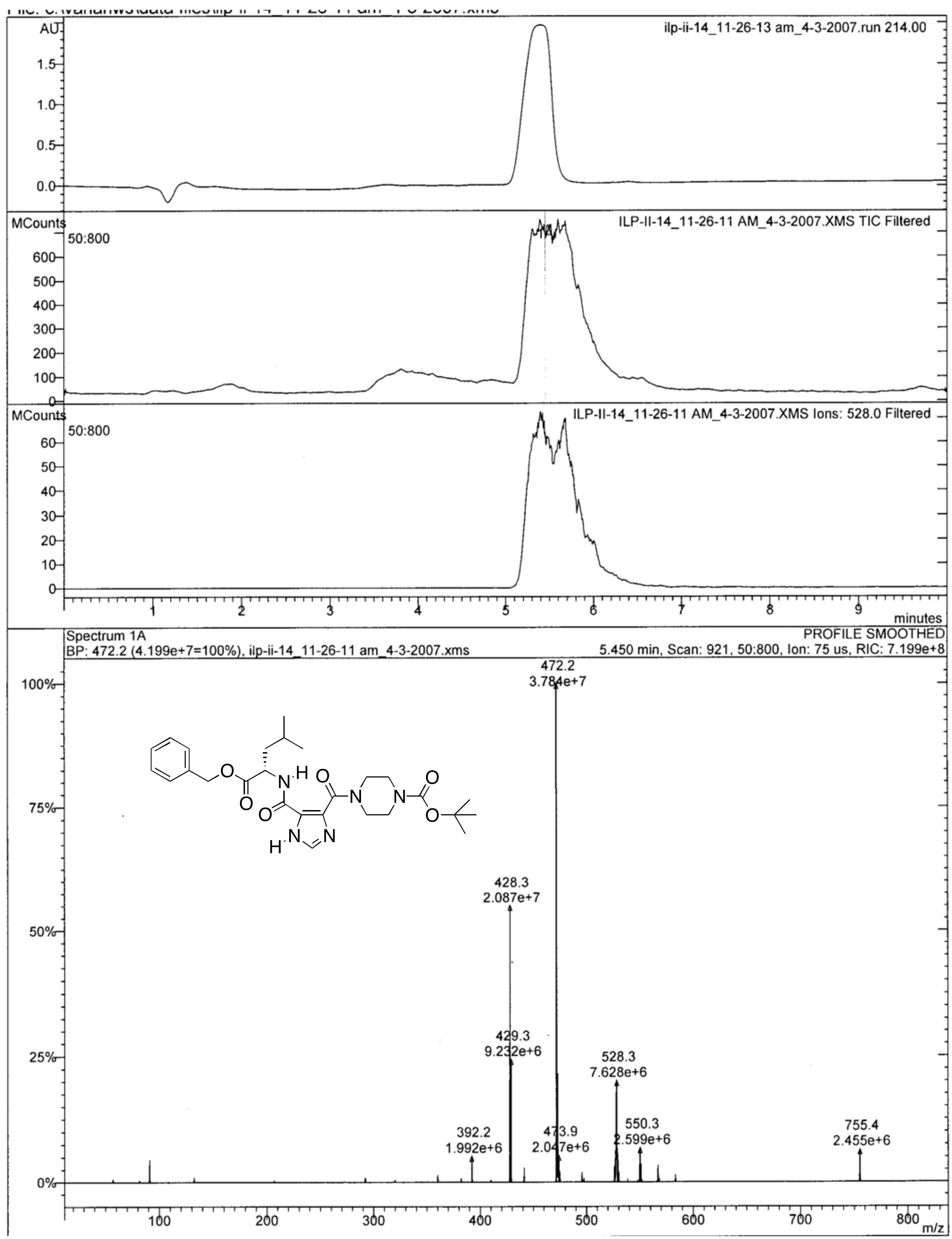


**Figure S81.** LC/MS data for **5{**81}.

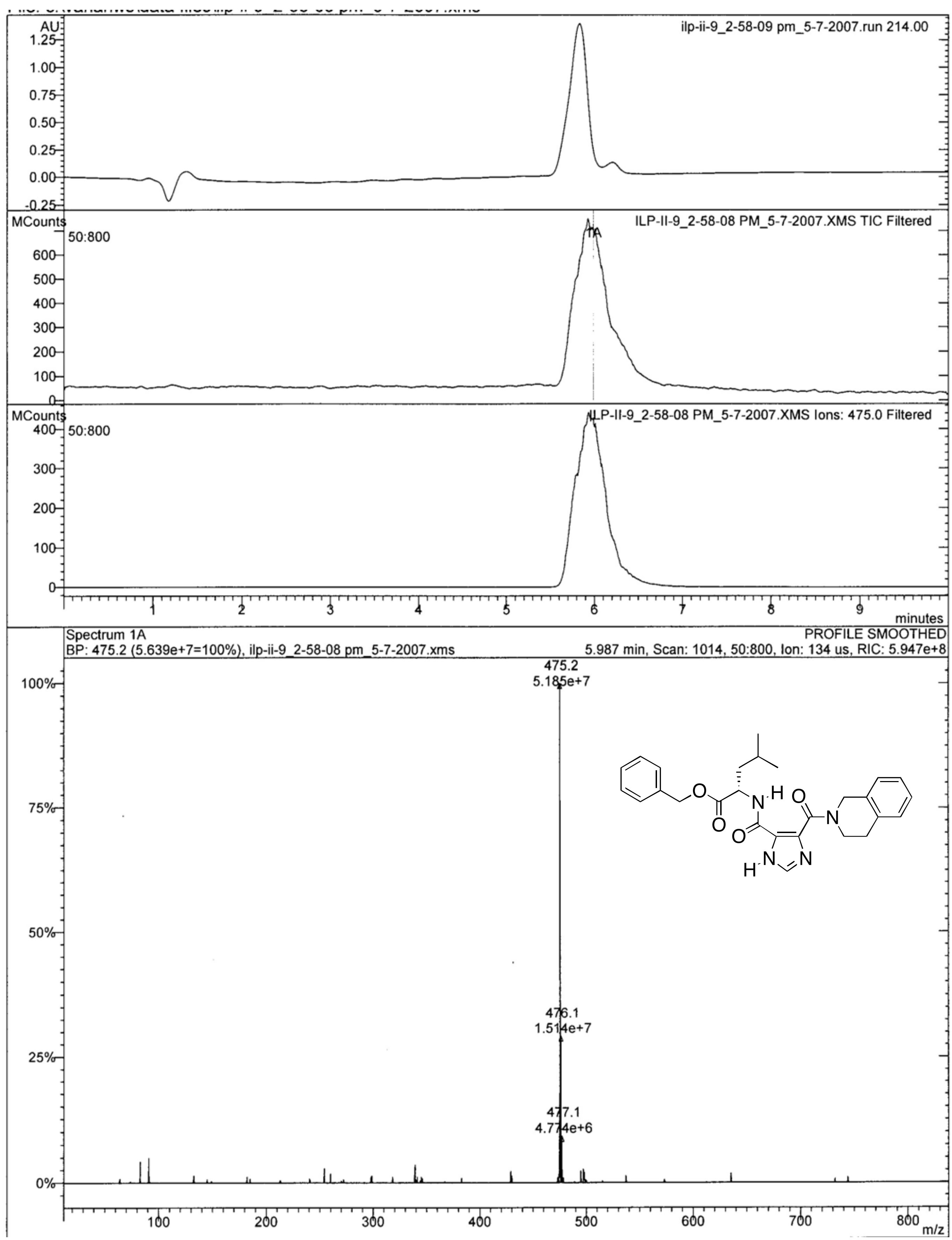


**Figure S82.** LC/MS data for **5{**82}.

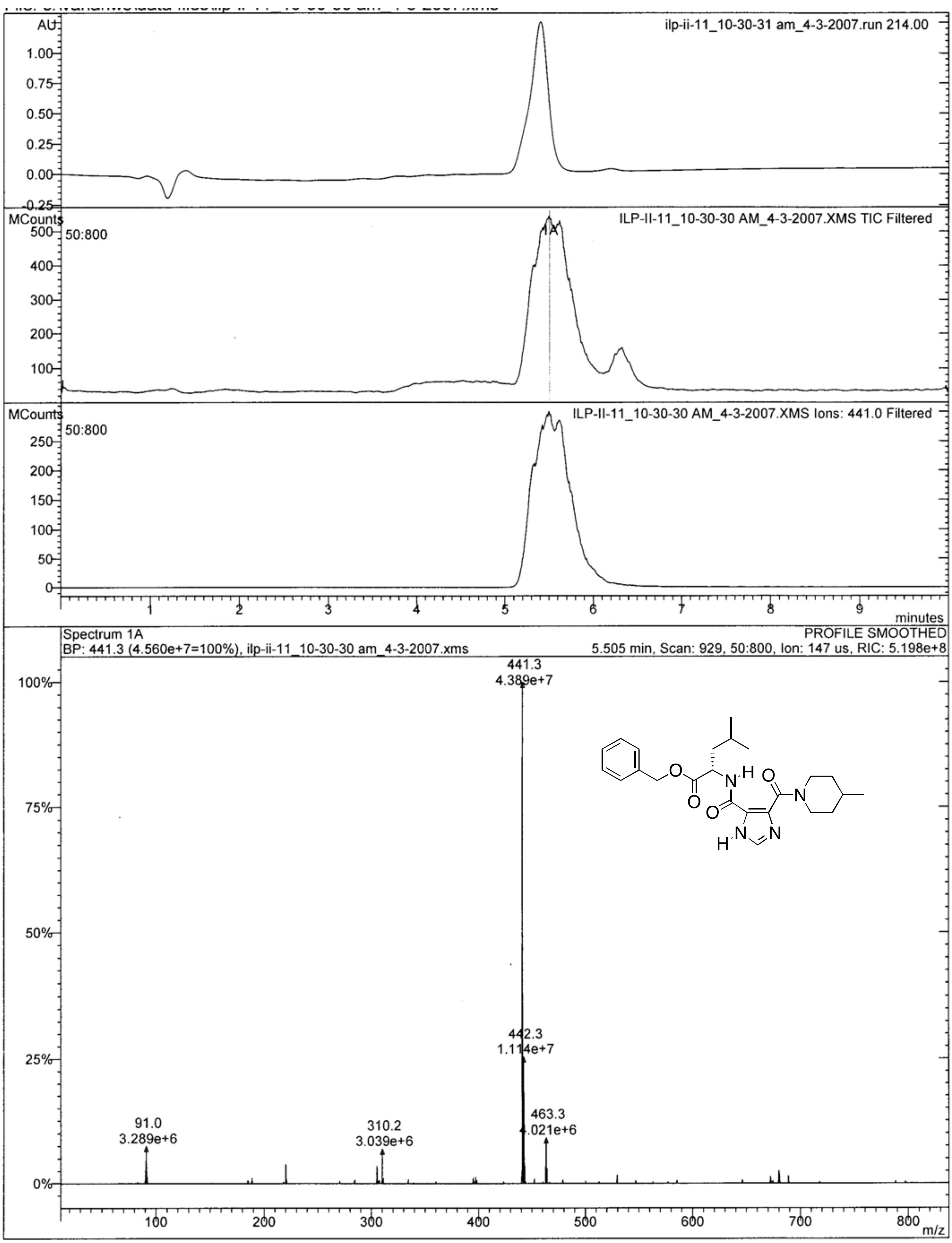


**Figure S83.** LC/MS data for **5{**83}.

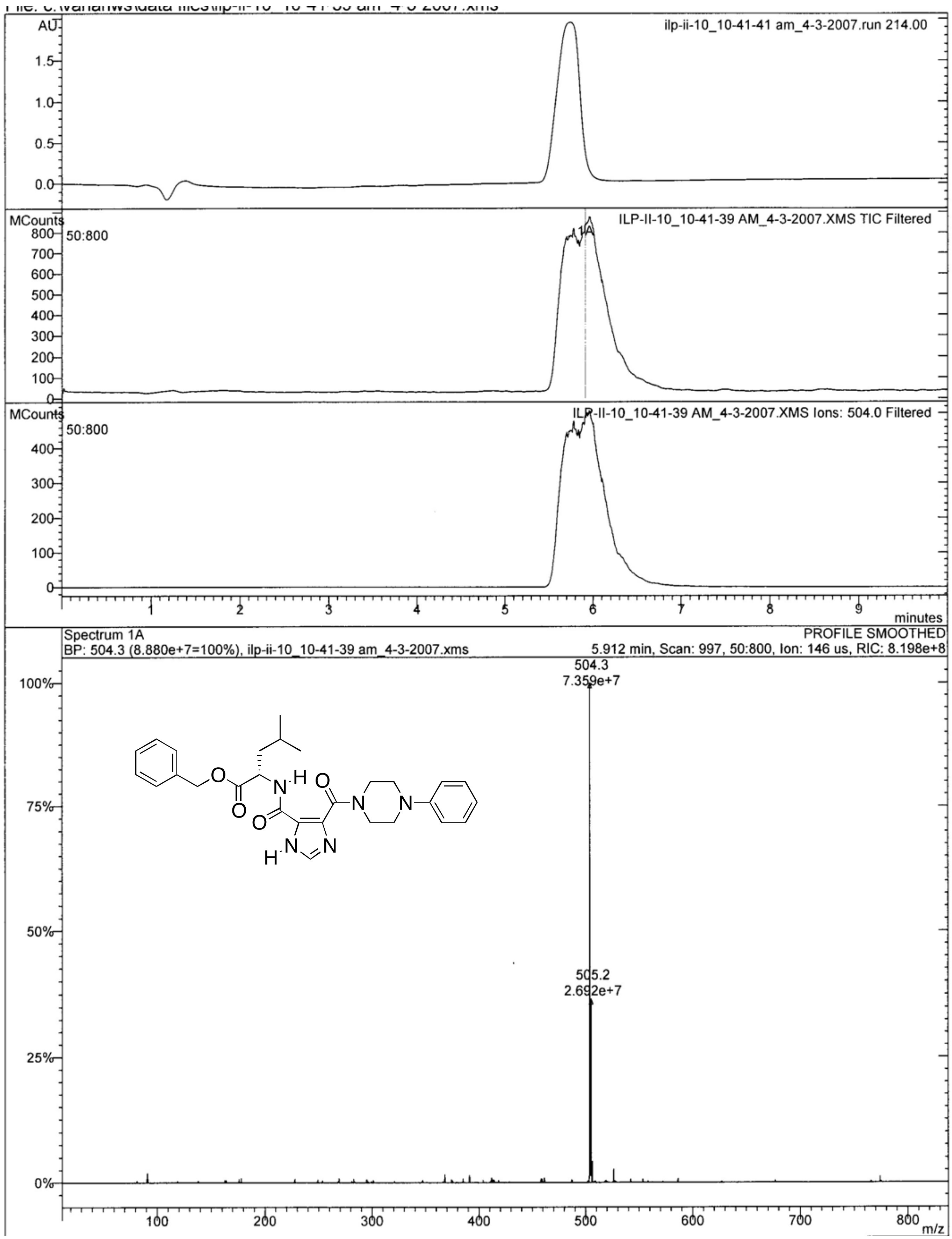


**Figure S84.** LC/MS data for **5{**84}.

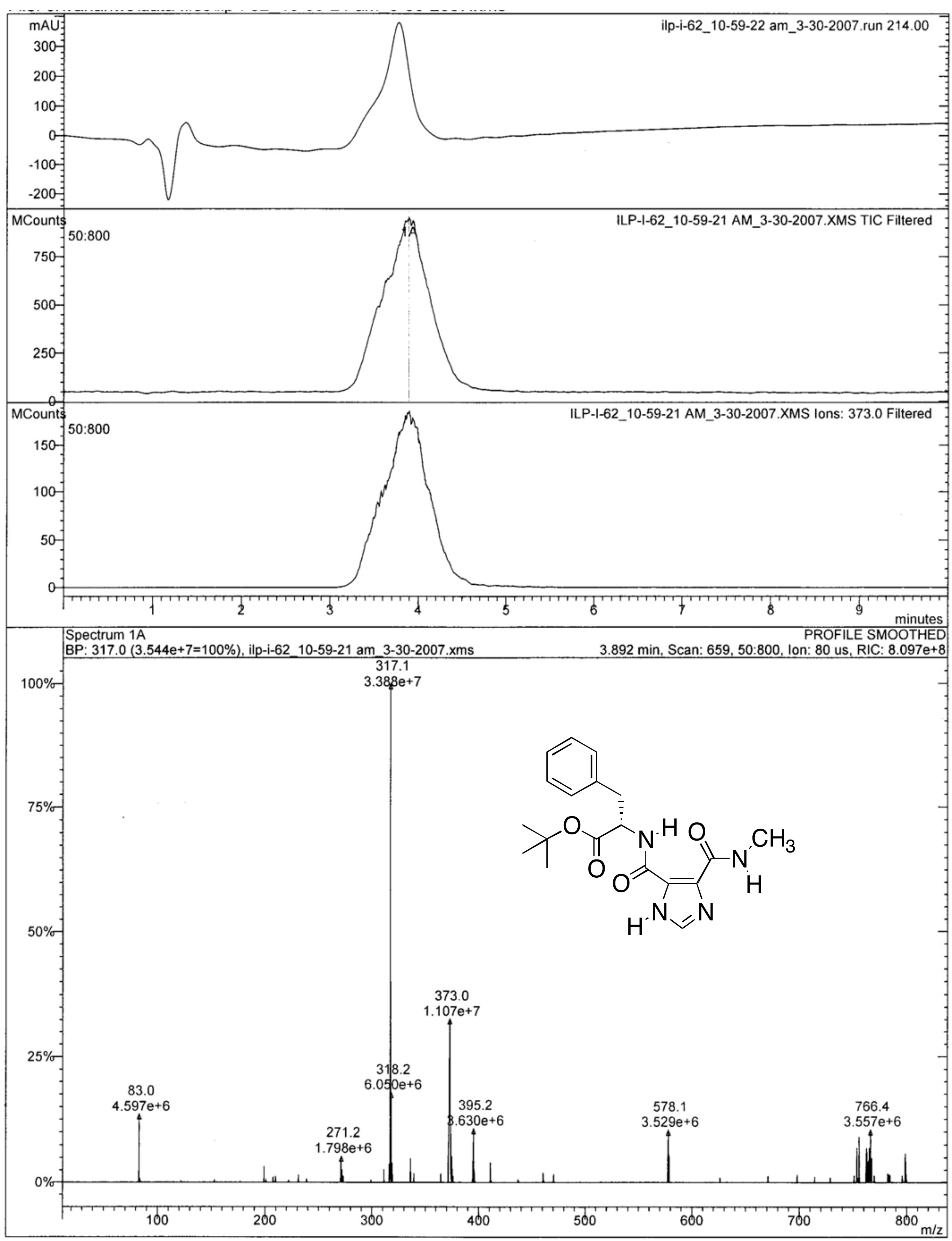


**Figure S85.** LC/MS data for **5{**85}.

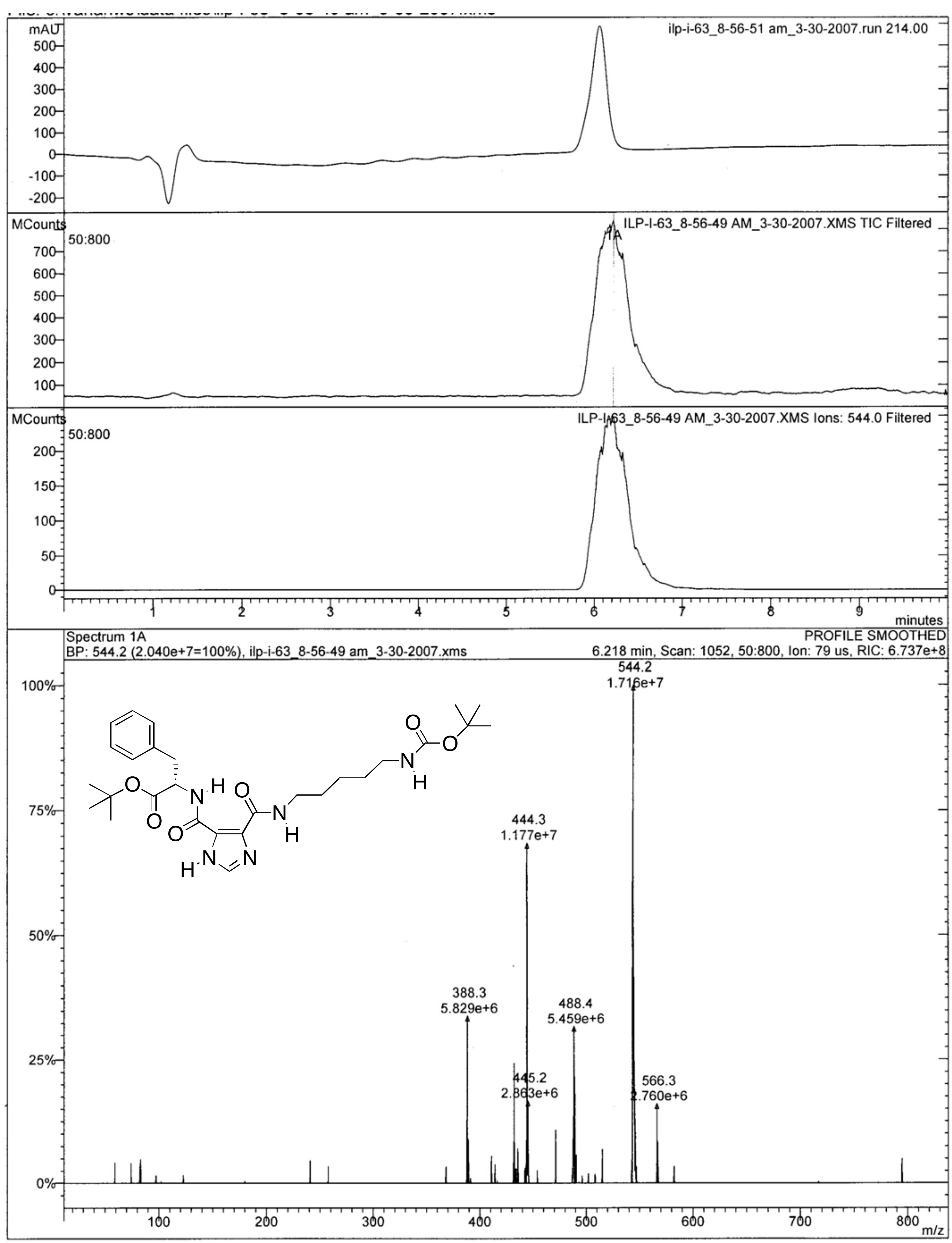


**Figure S86.** LC/MS data for **5{**86}.

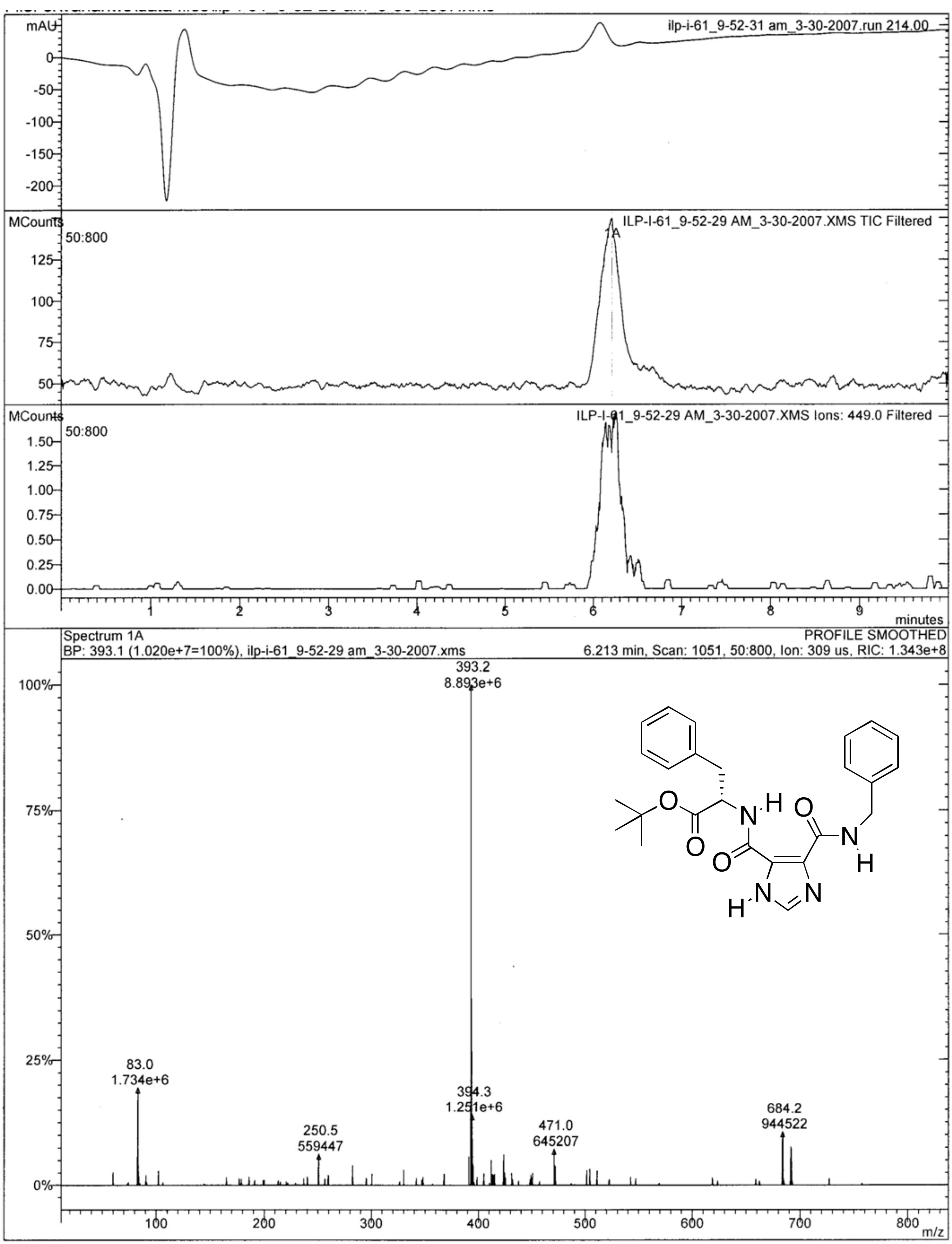


**Figure S87.** LC/MS data for **5{**87}.

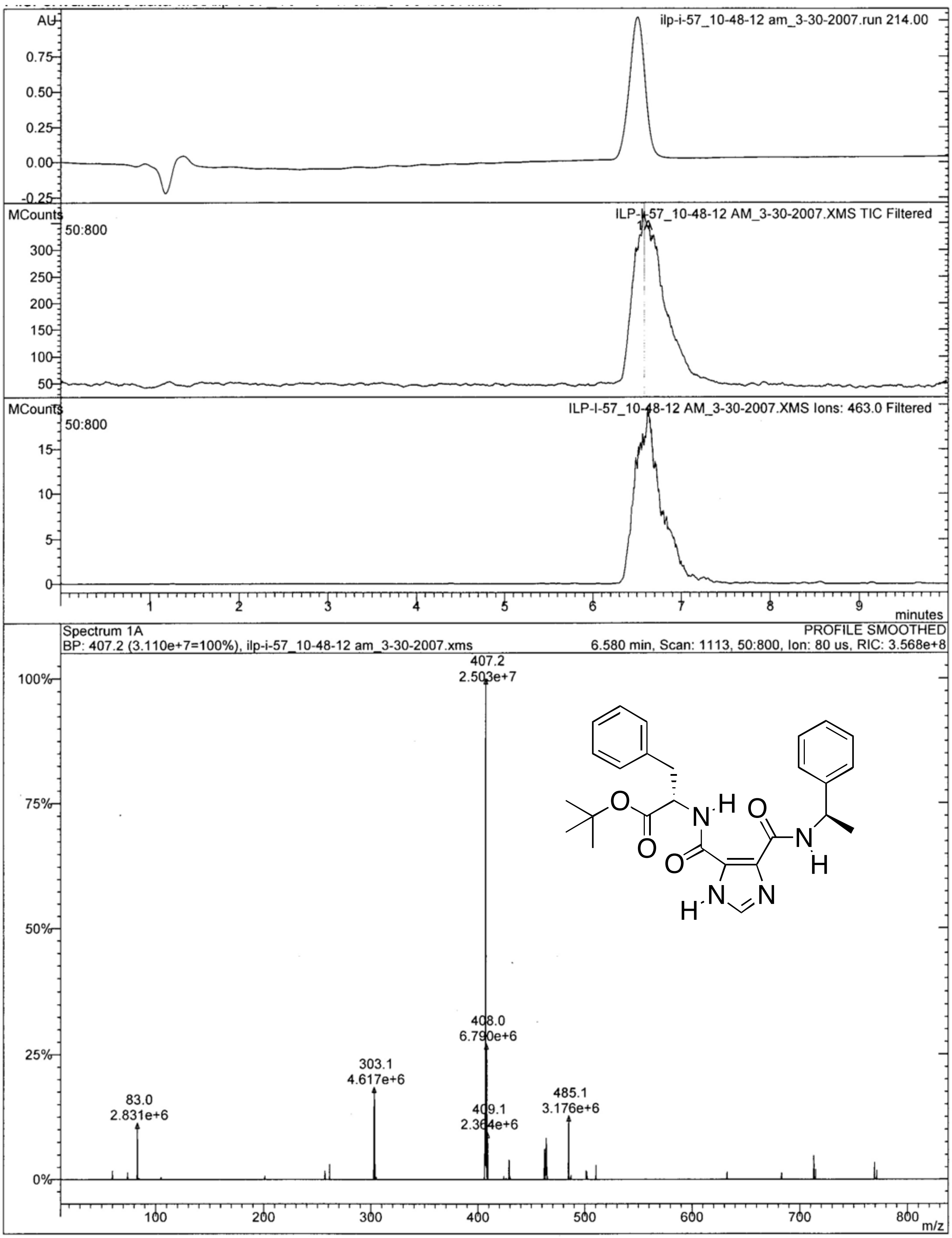


**Figure S88.** LC/MS data for **5{**88}.

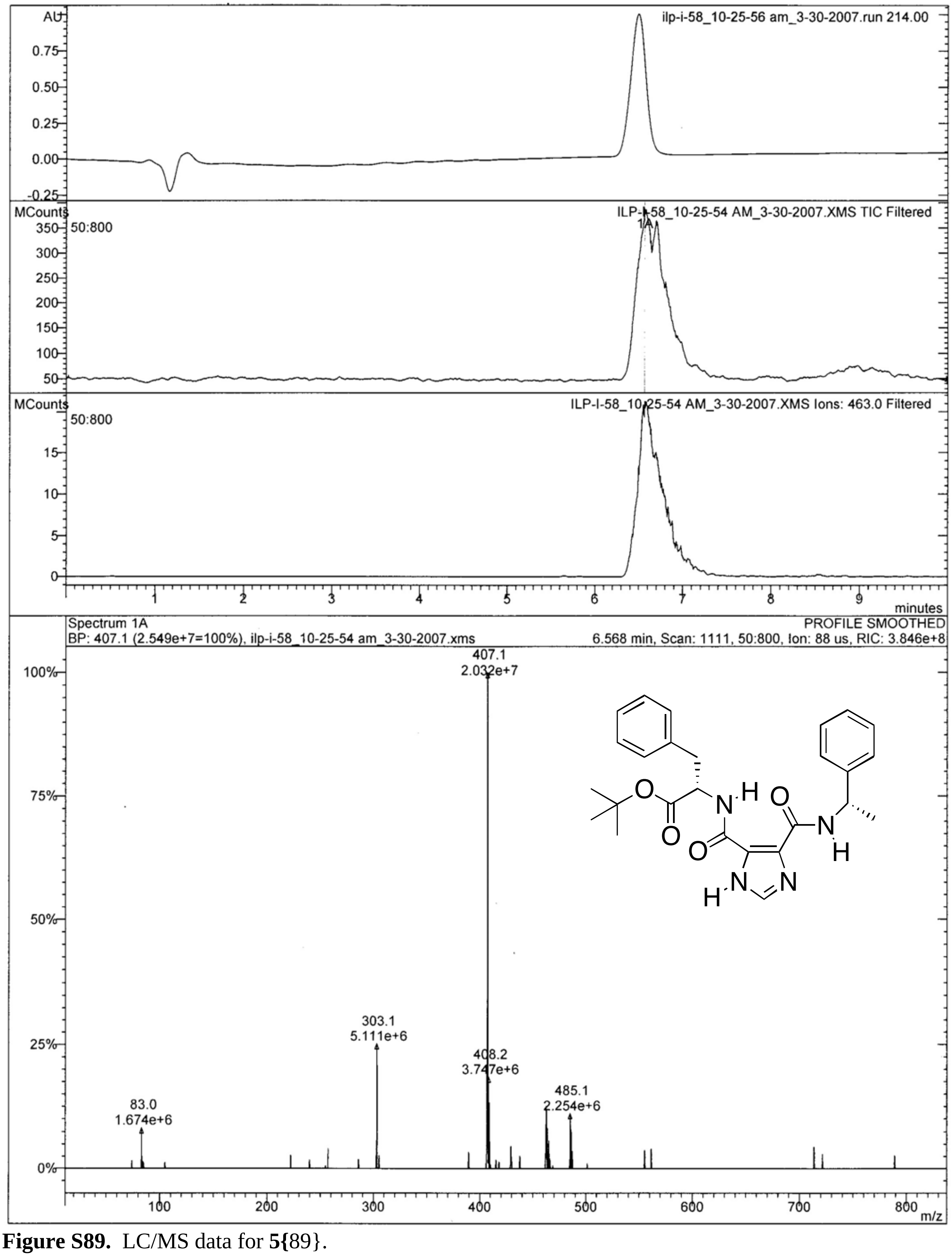


**Figure S89.** LC/MS data for **5{**89}.

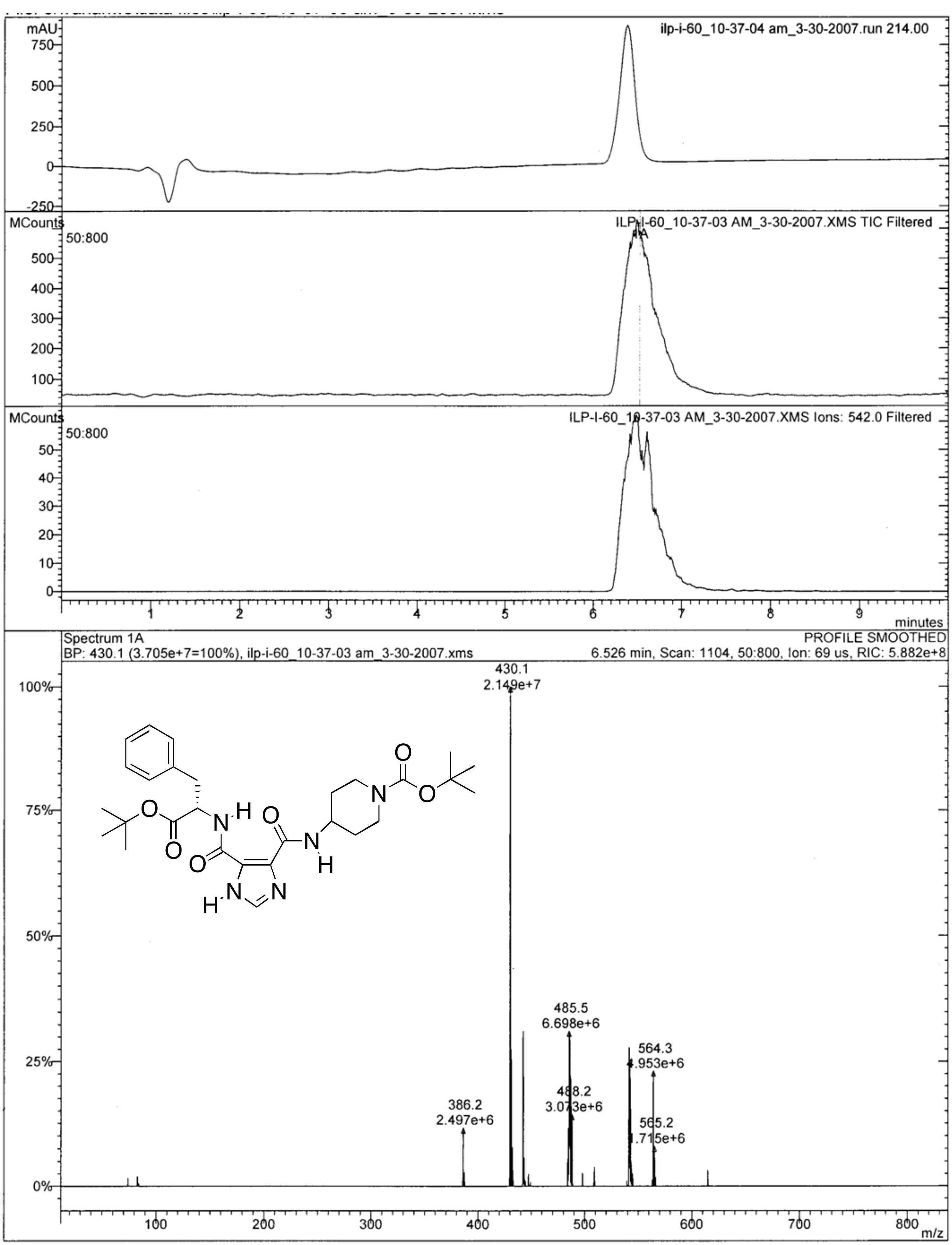


**Figure S90.** LC/MS data for **5{**90}.

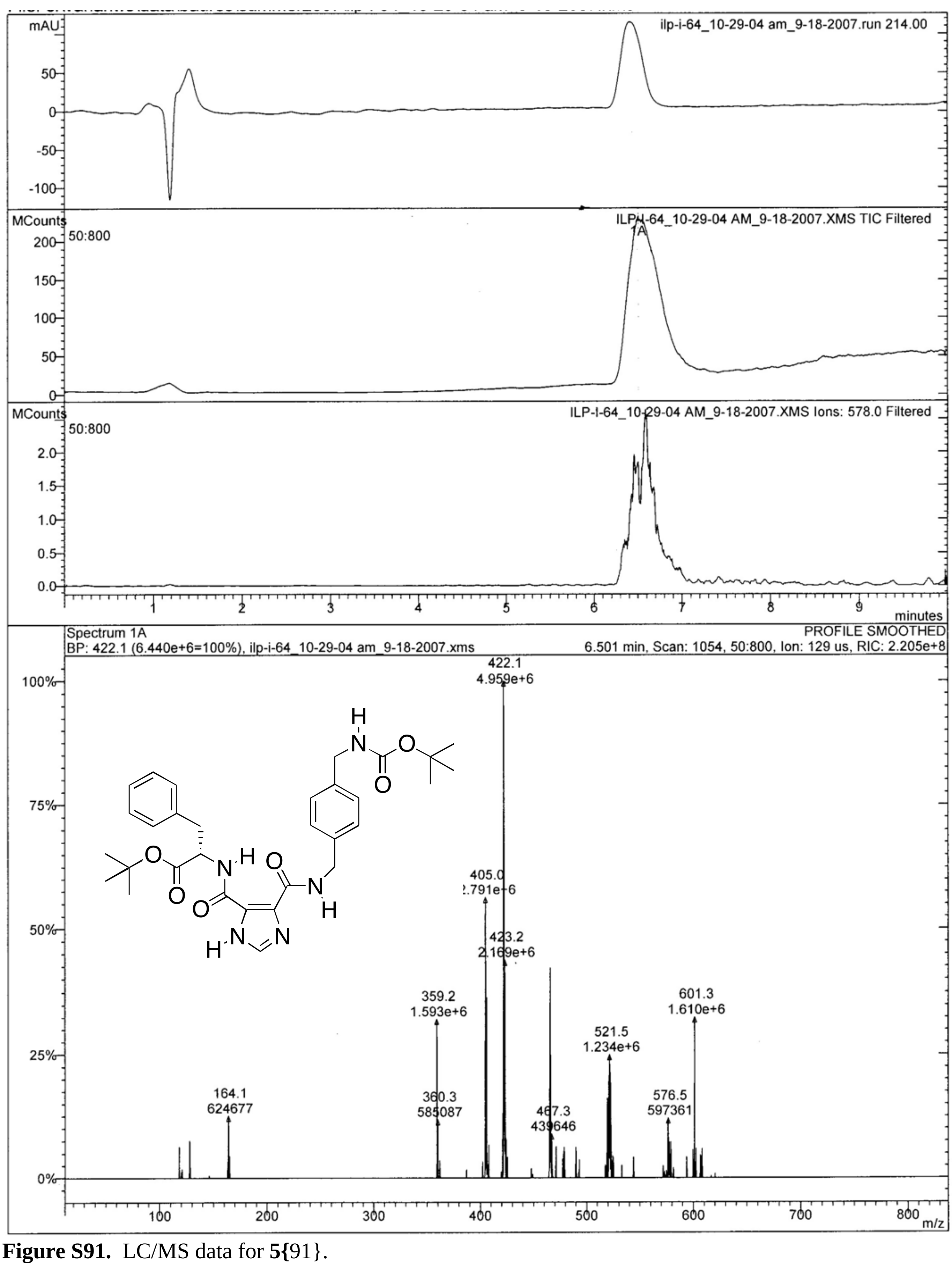


**Figure S91.** LC/MS data for **5{**91}.

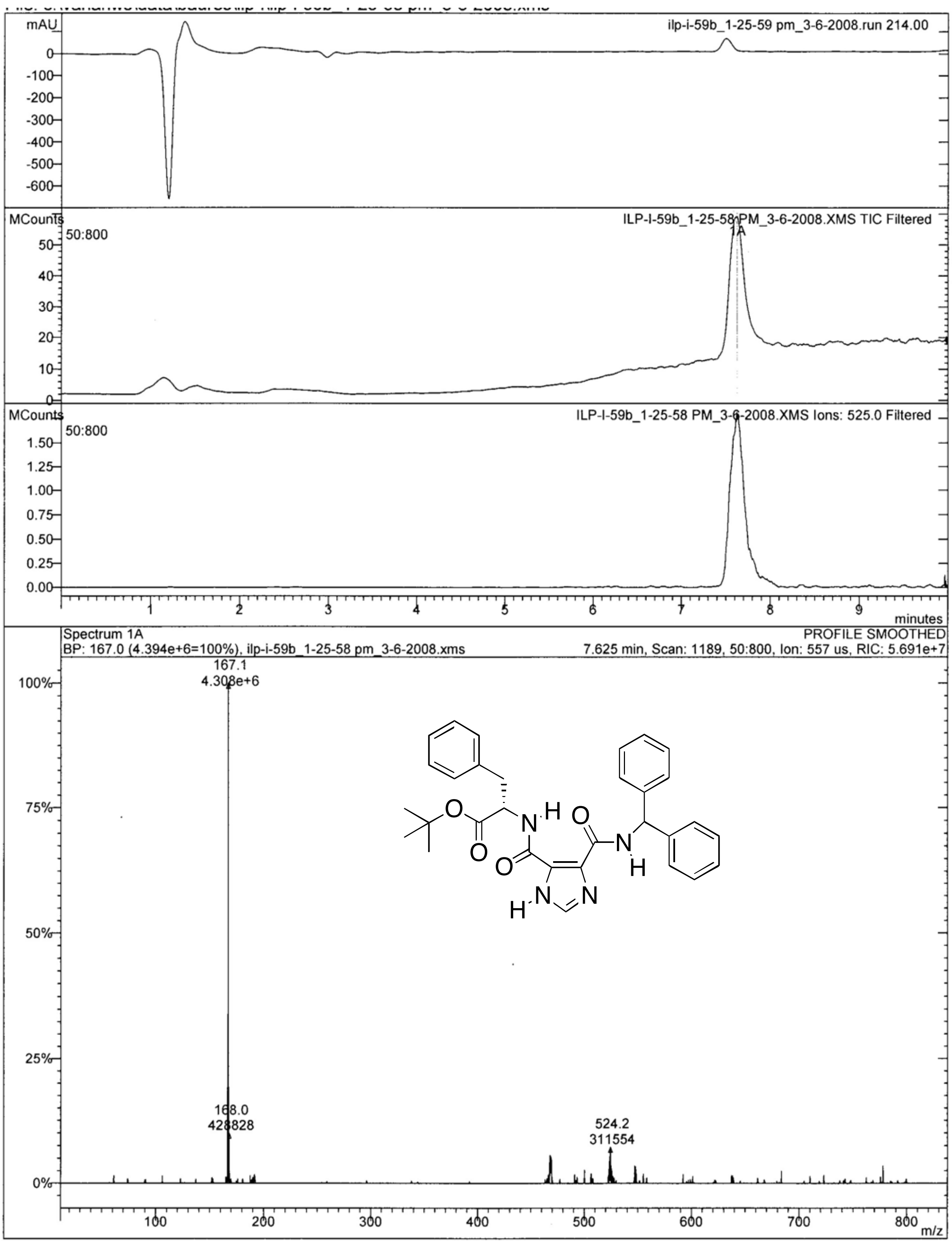


**Figure S92.** LC/MS data for **5{**92}.

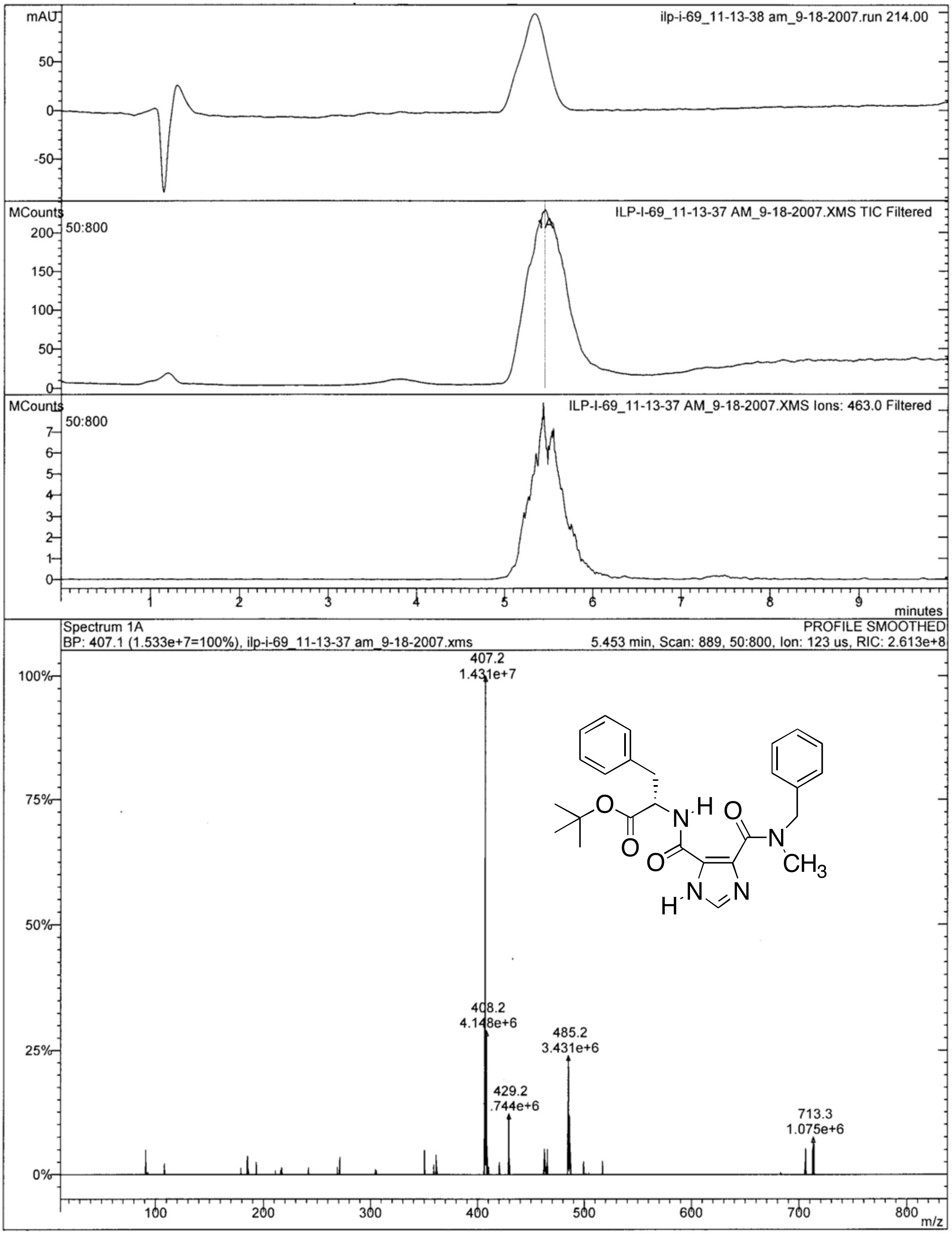


**Figure S93.** LC/MS data for **5{**93}.

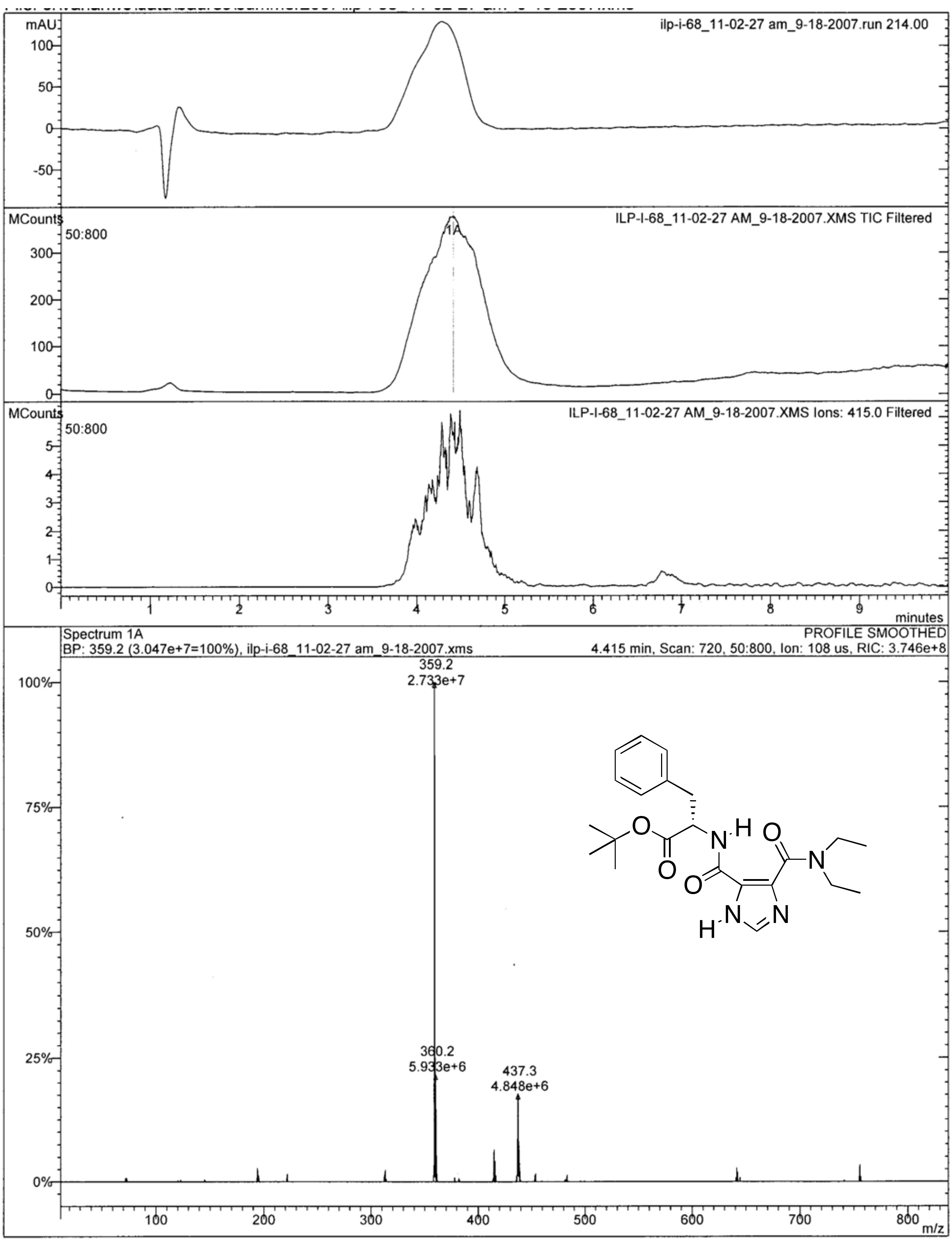


**Figure S94.** LC/MS data for **5{**94}.

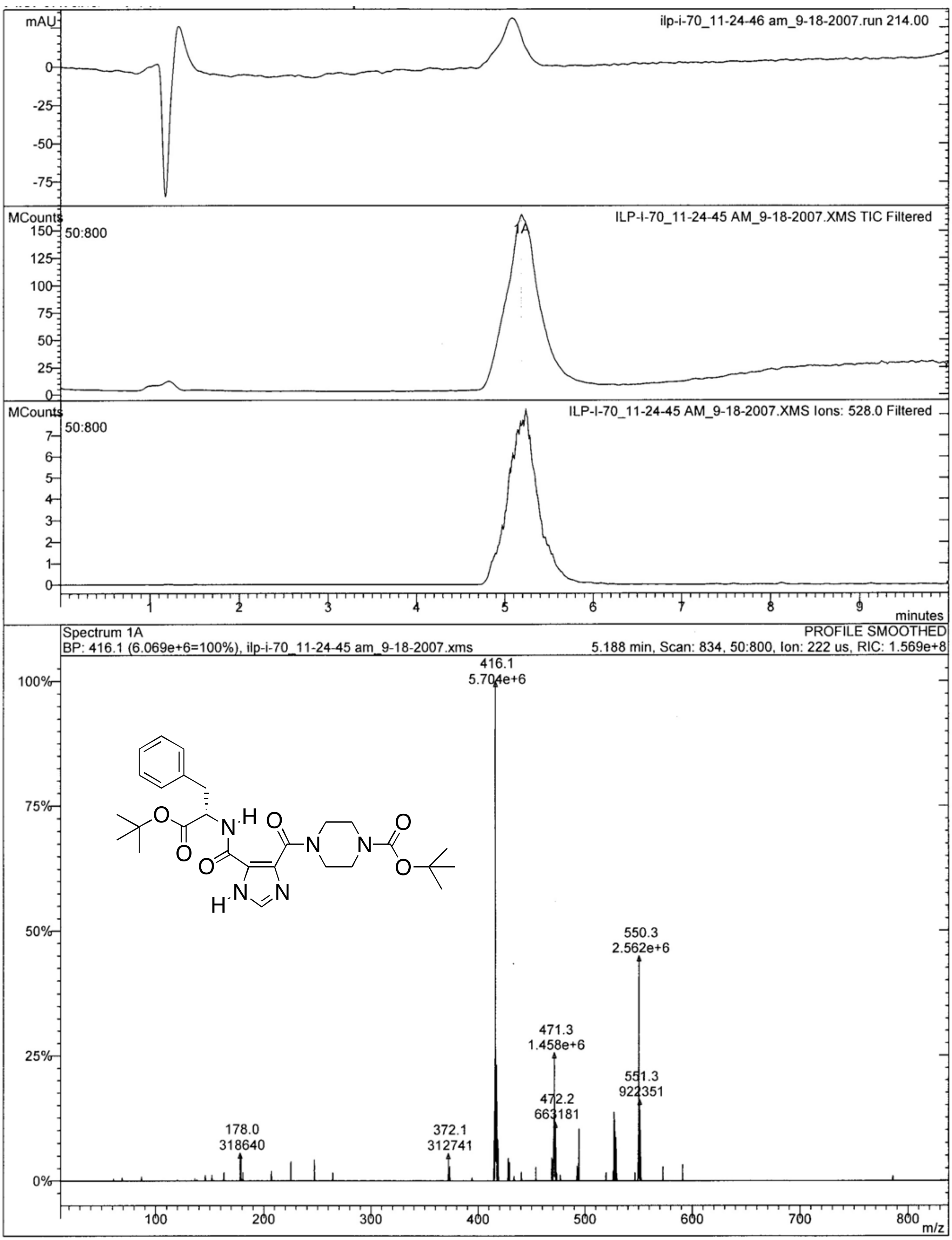


**Figure S95.** LC/MS data for **5{**95}.

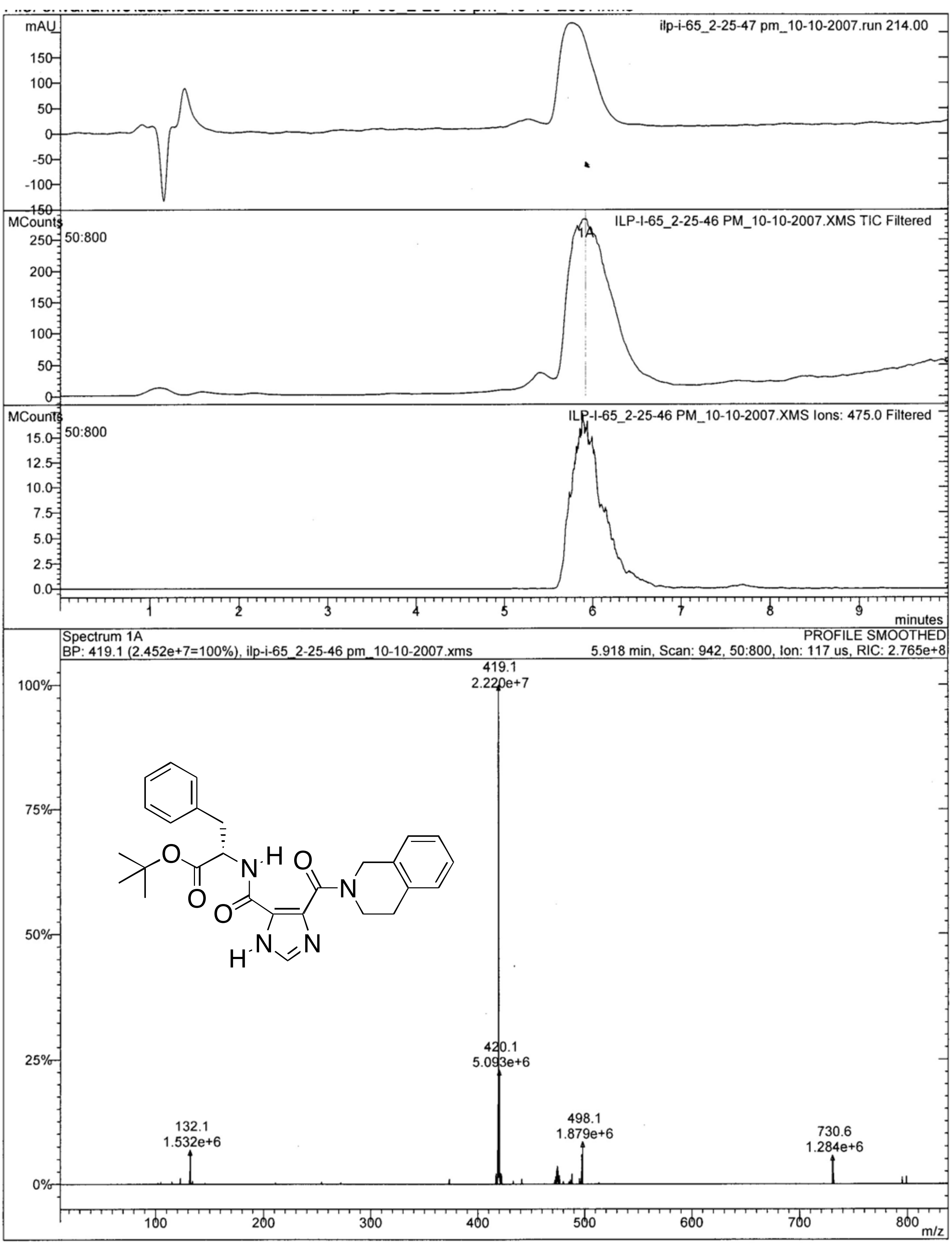


**Figure S96.** LC/MS data for **5{**96}.

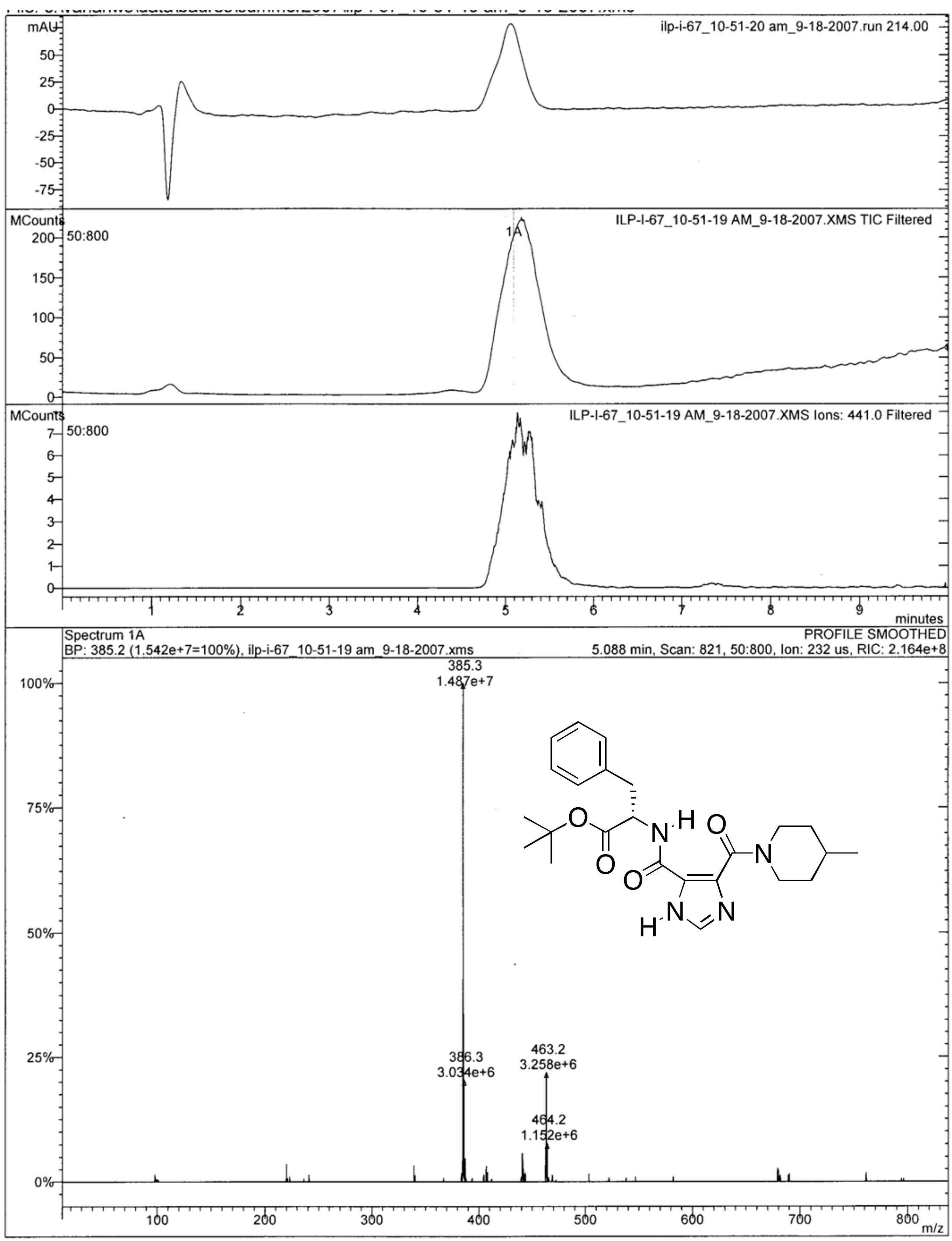


**Figure S97.** LC/MS data for **5{**97}.

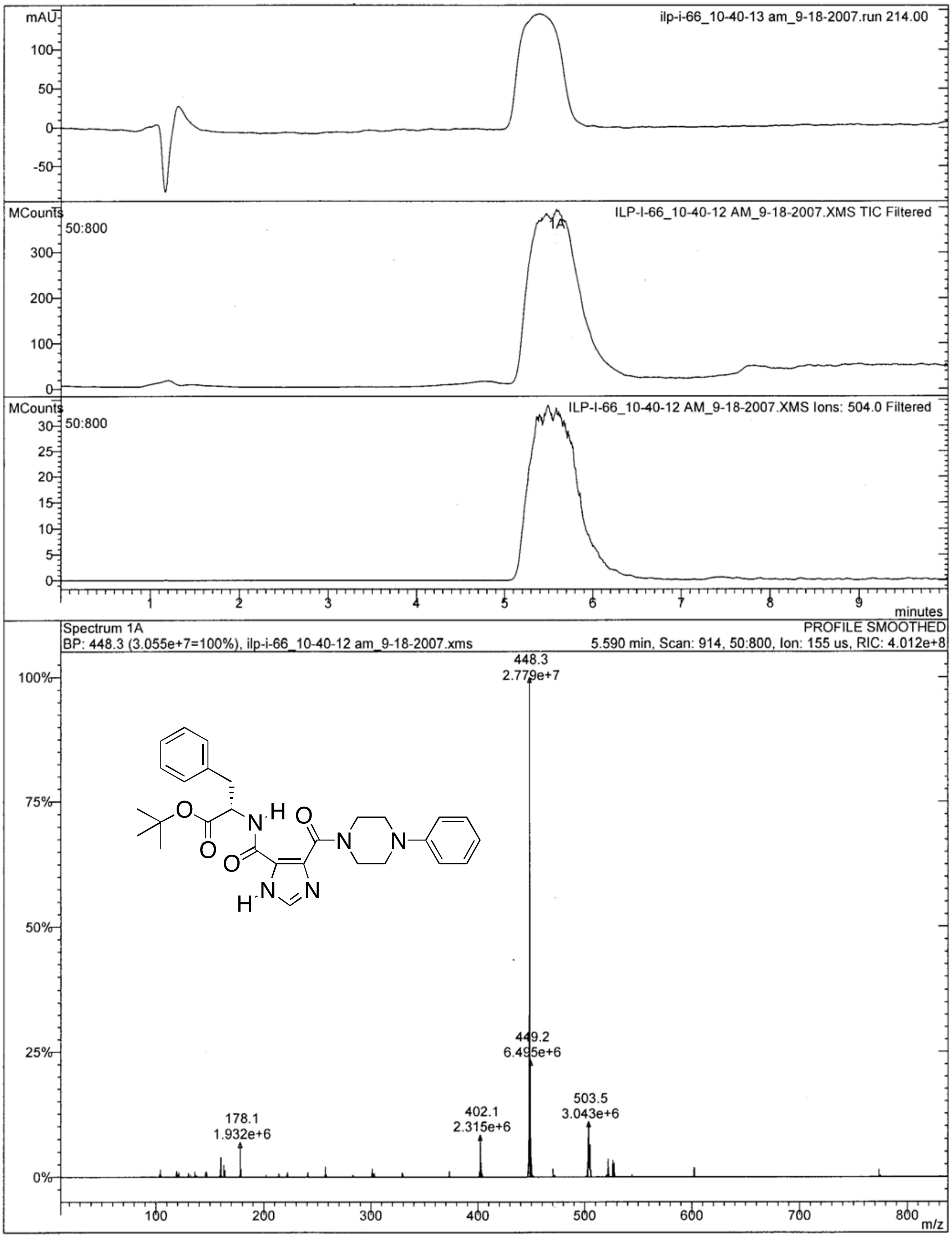


**Figure S98.** LC/MS data for **5{**98}.

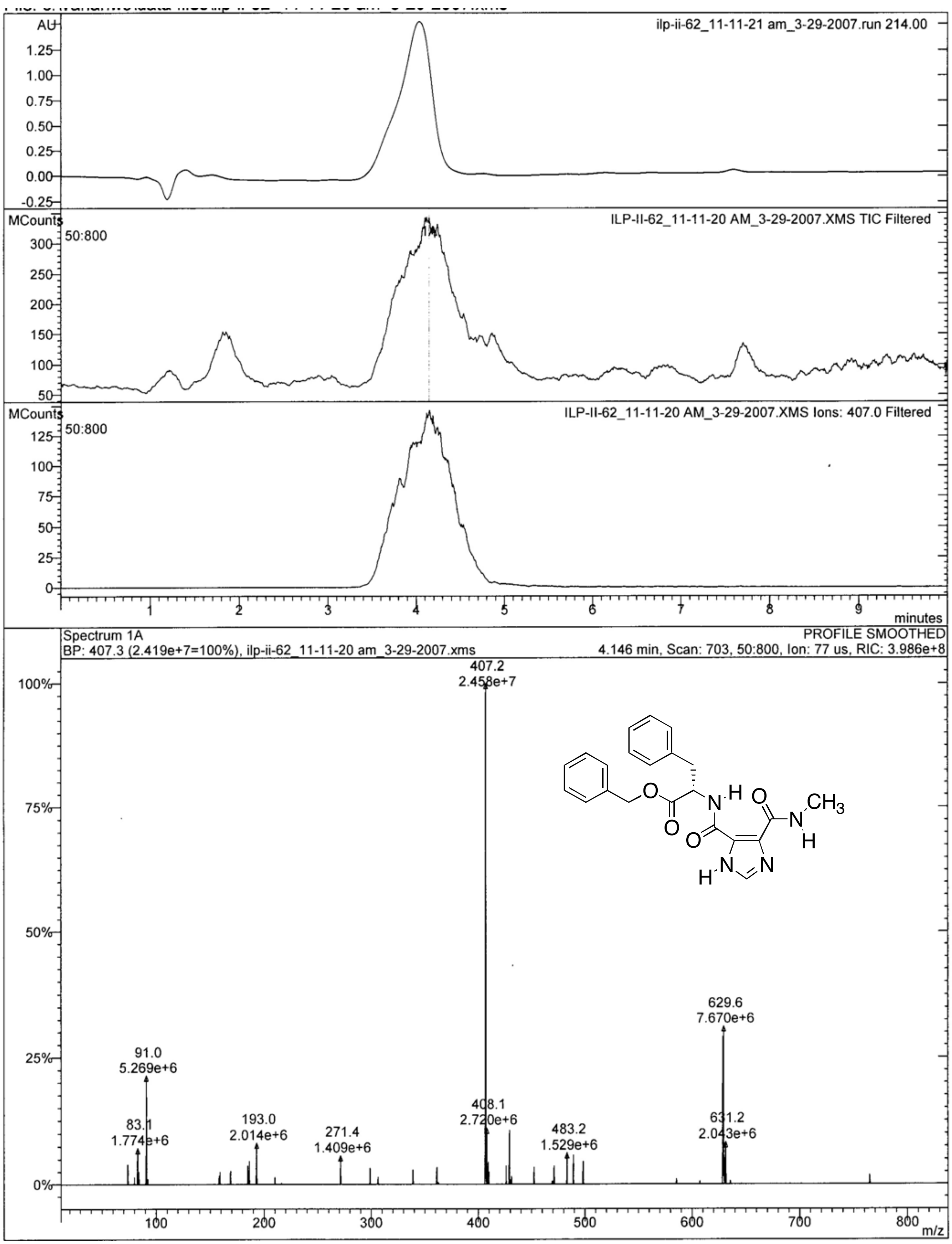


**Figure S99.** LC/MS data for **5{**99}.

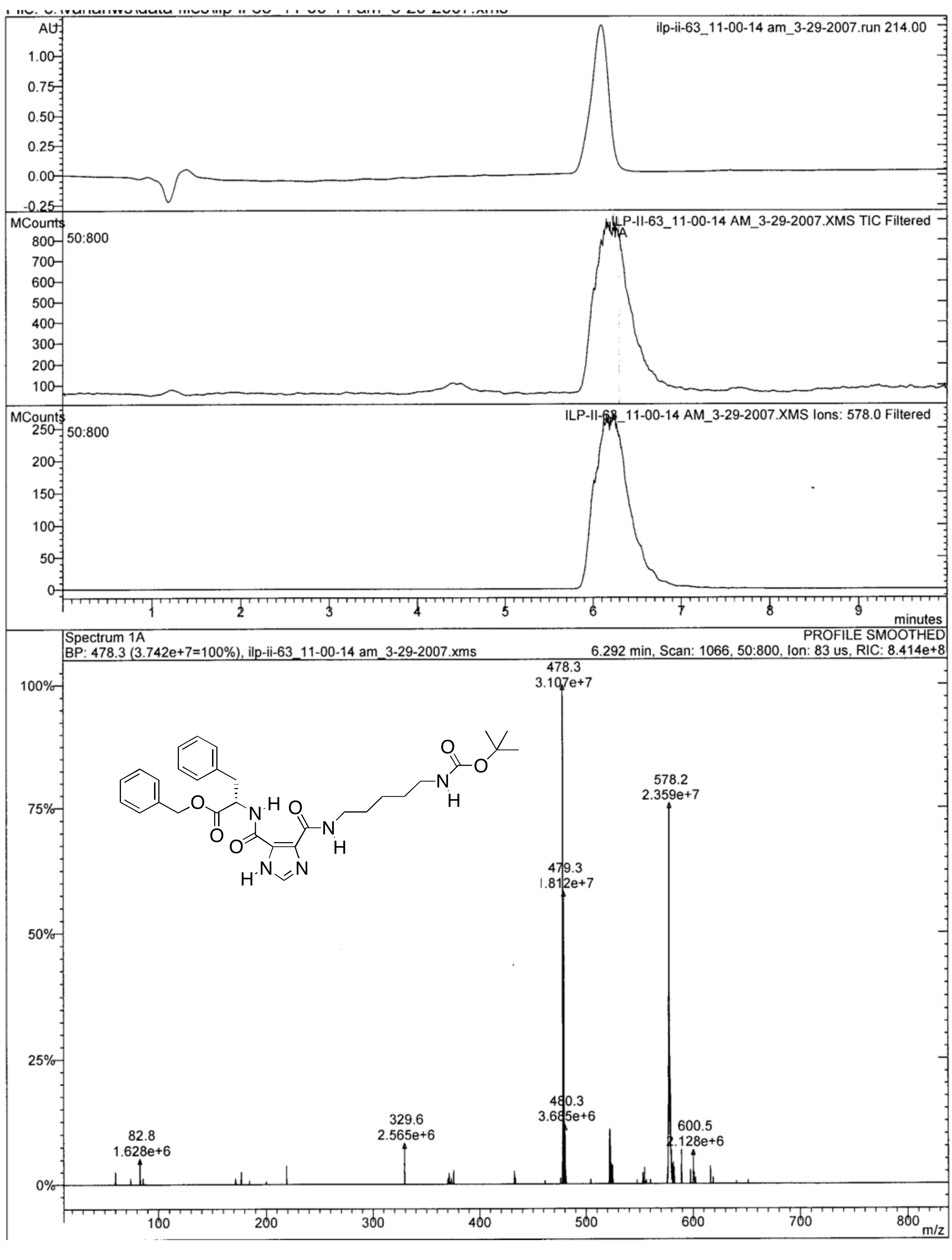


**Figure S100.** LC/MS data for **5{**100}.

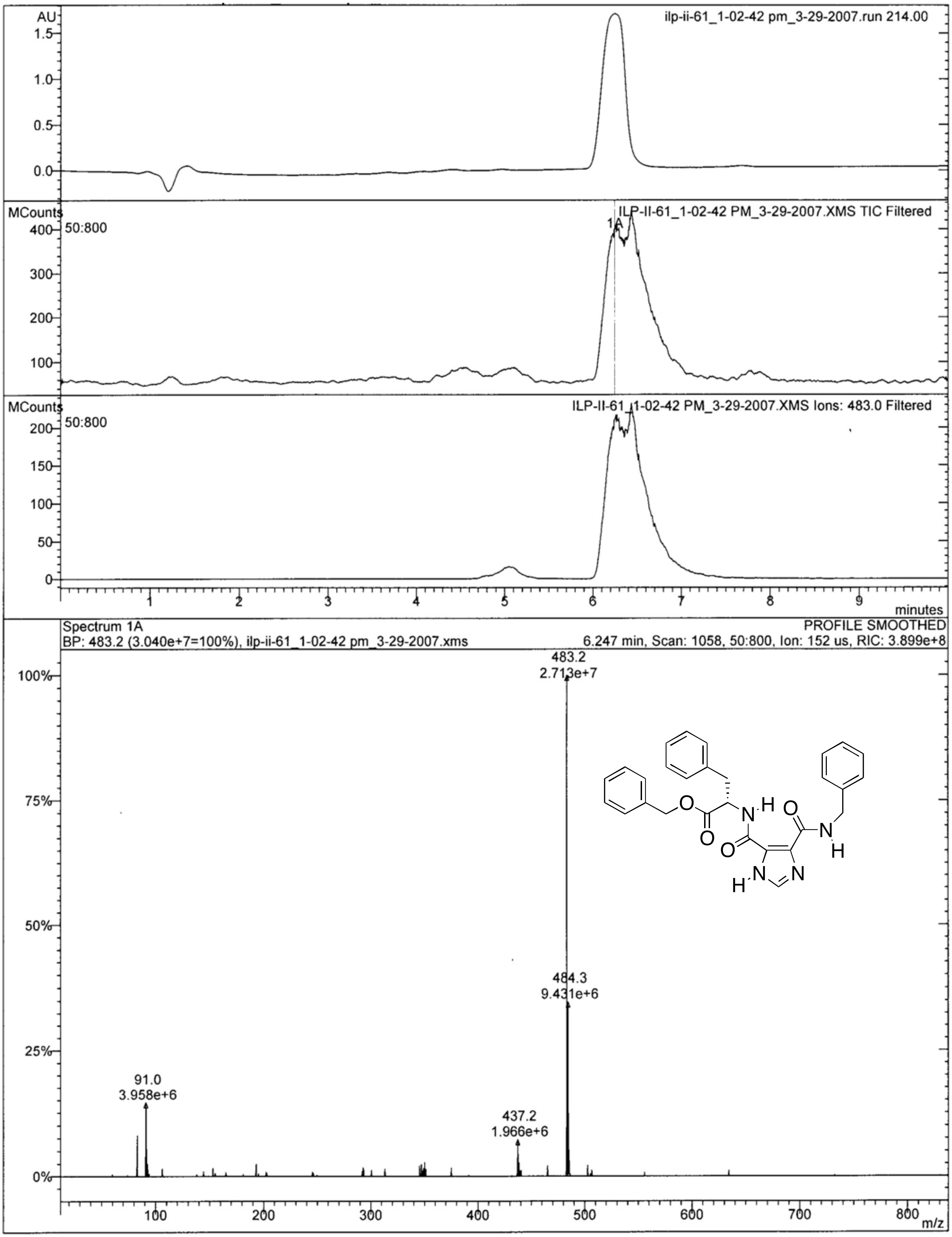


**Figure S101.** LC/MS data for **5{**101}.

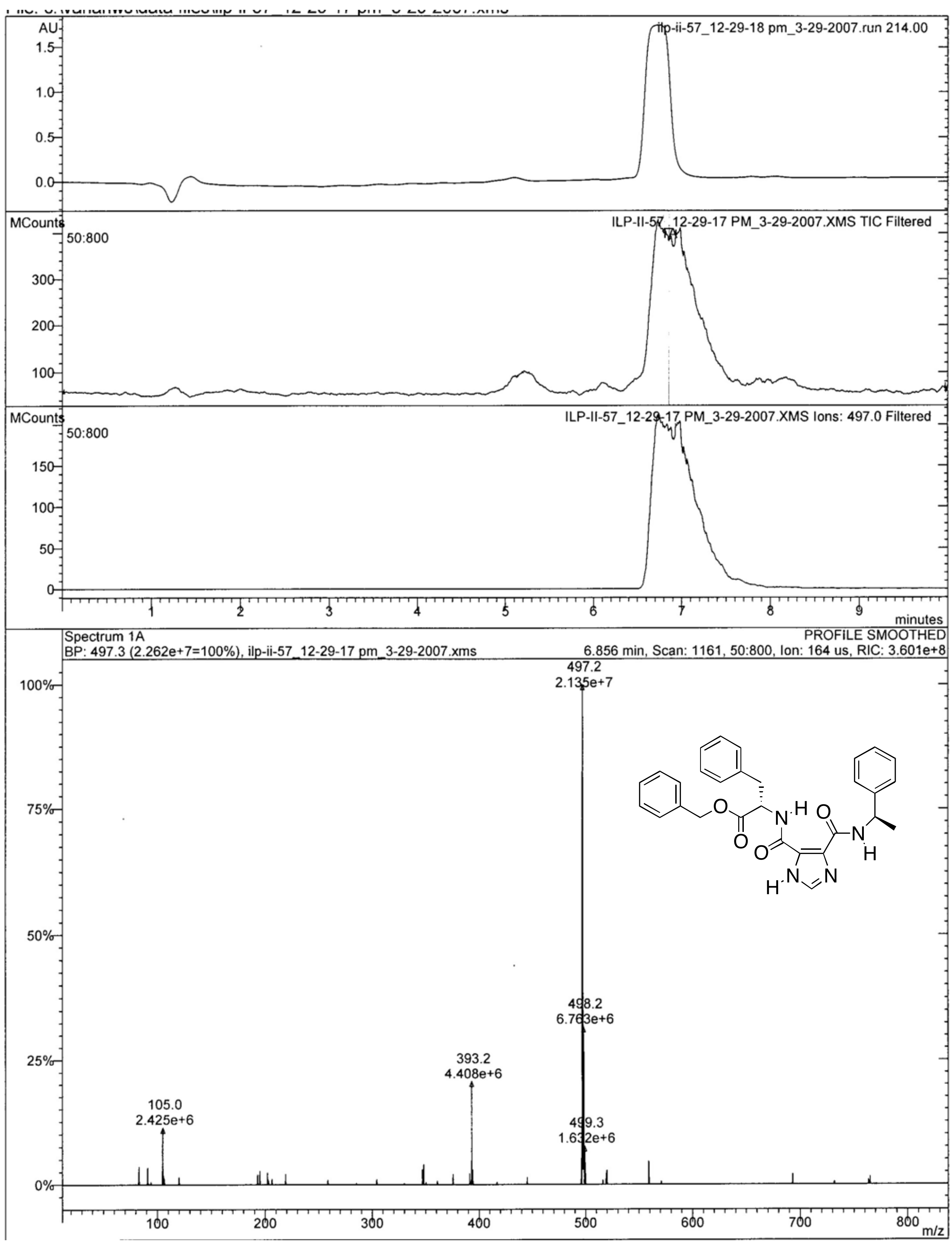


**Figure S102.** LC/MS data for **5{**102}.

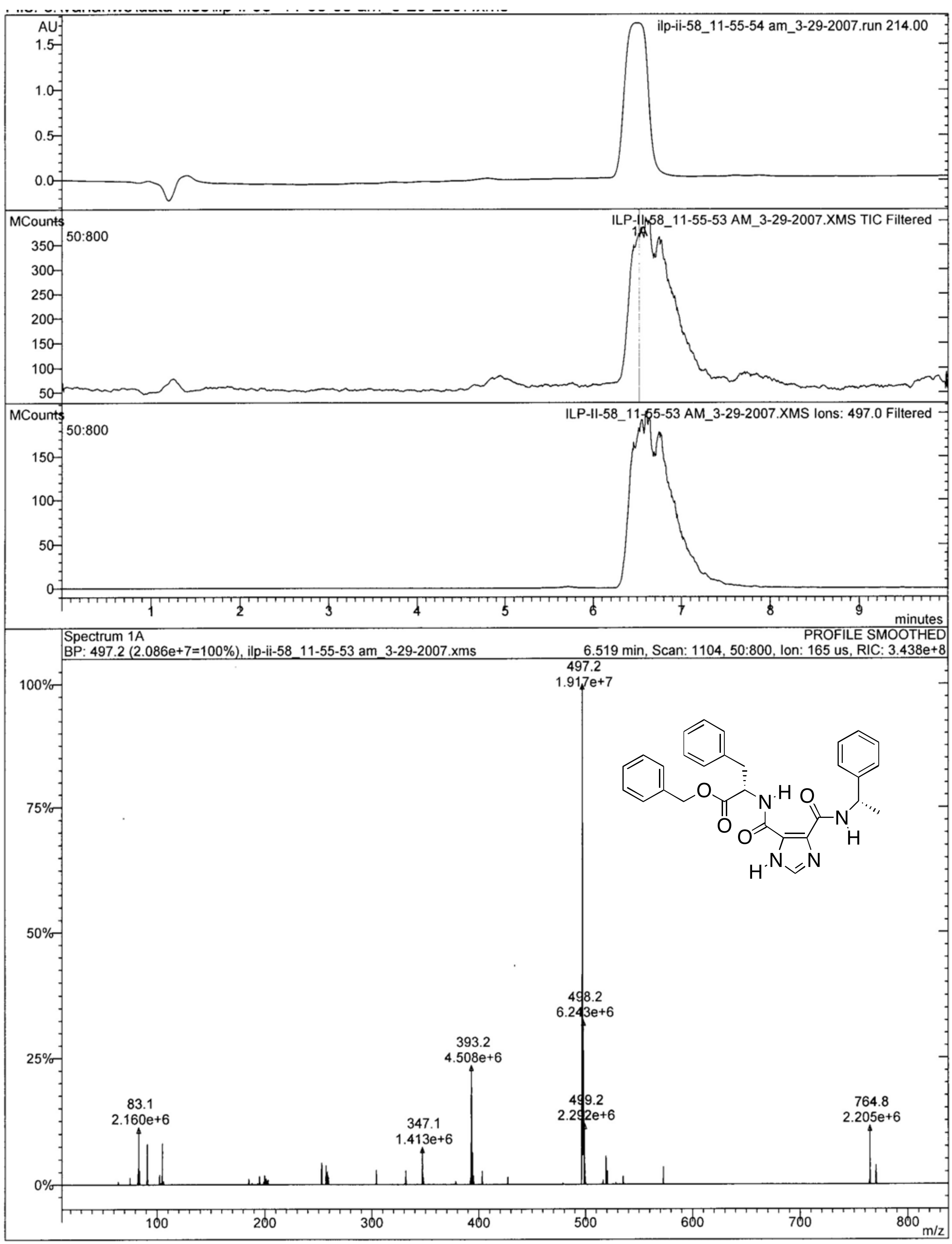


**Figure S103.** LC/MS data for **5{**103}.

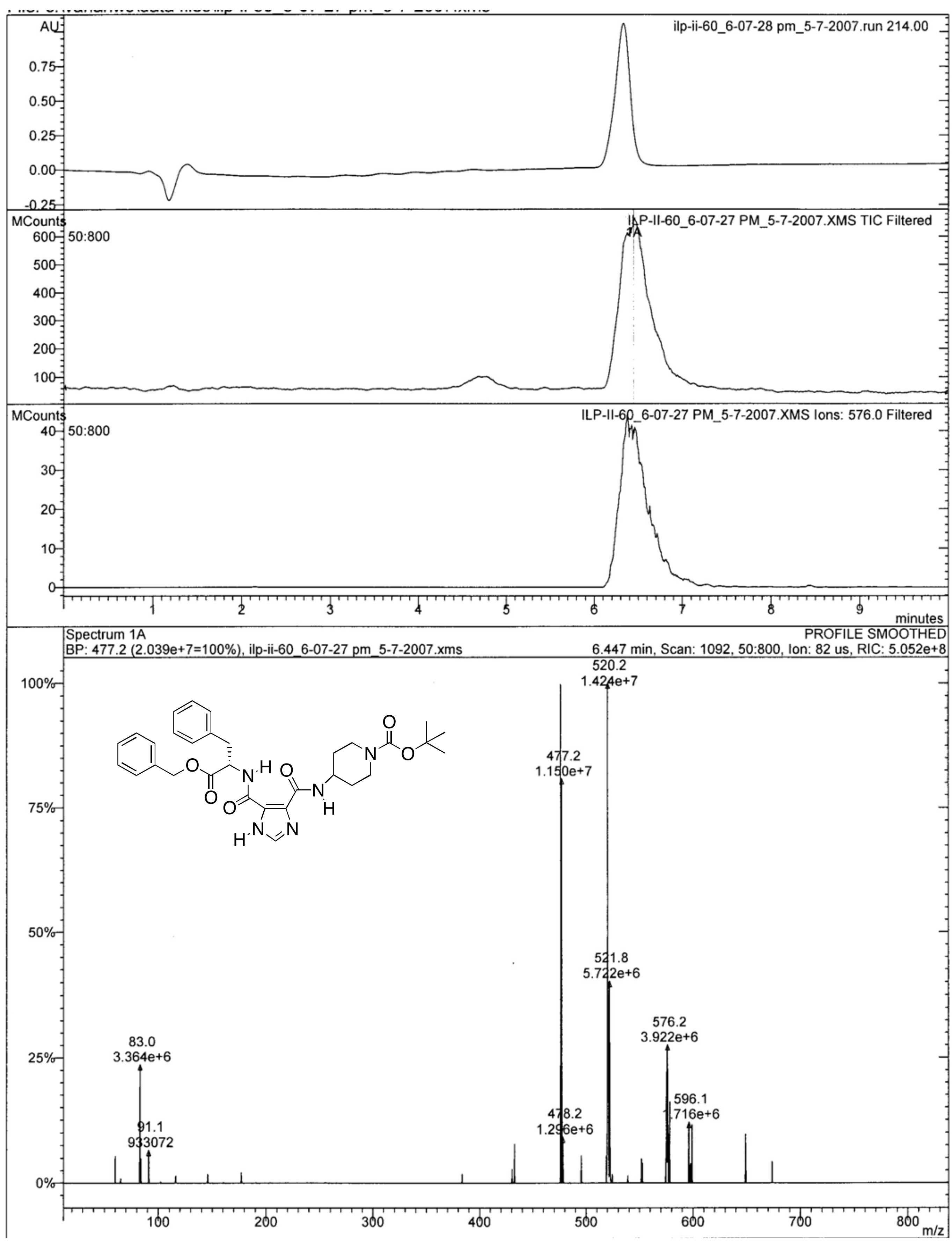


**Figure S104.** LC/MS data for **5{**104}.

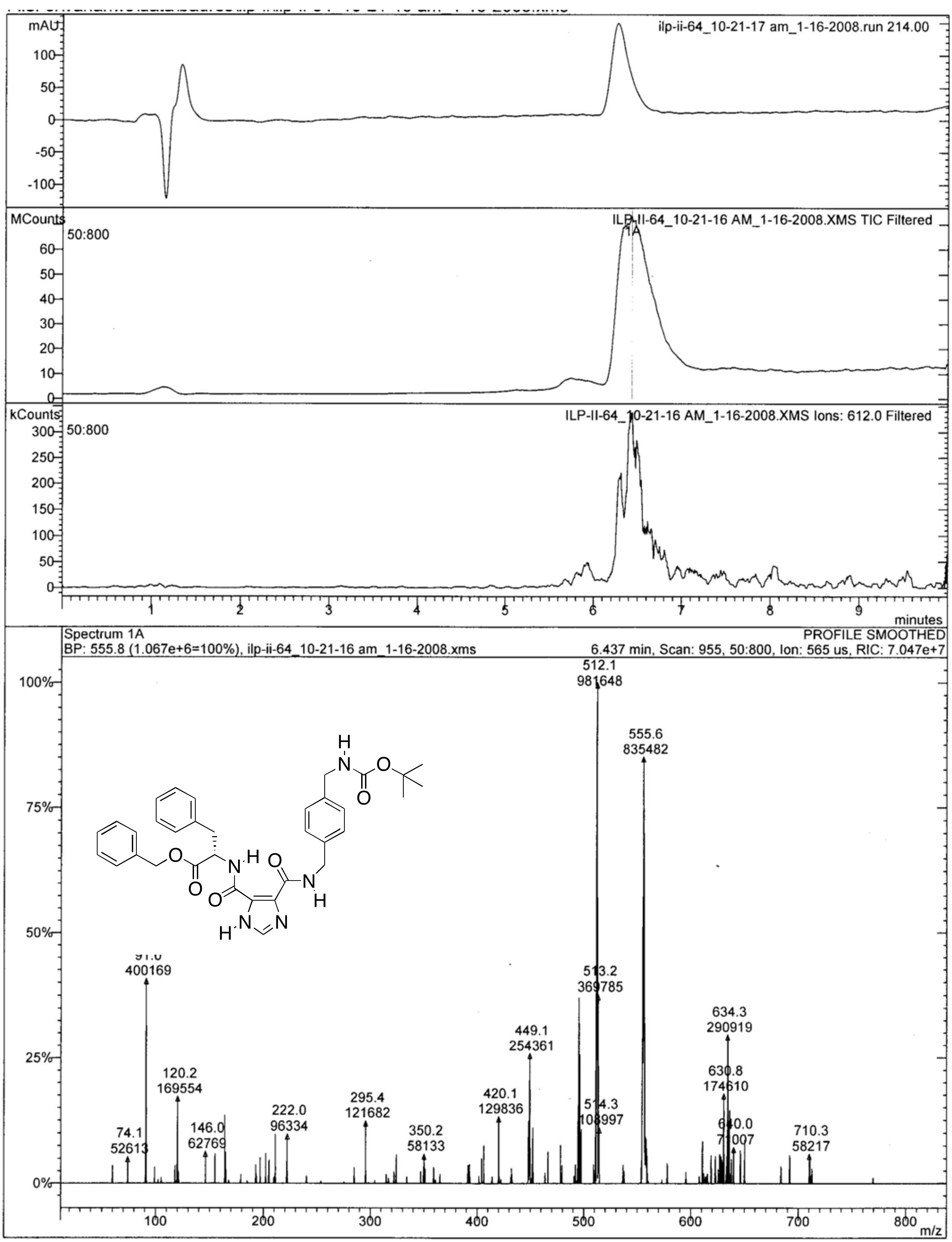


**Figure S105.** LC/MS data for **5{**105}.

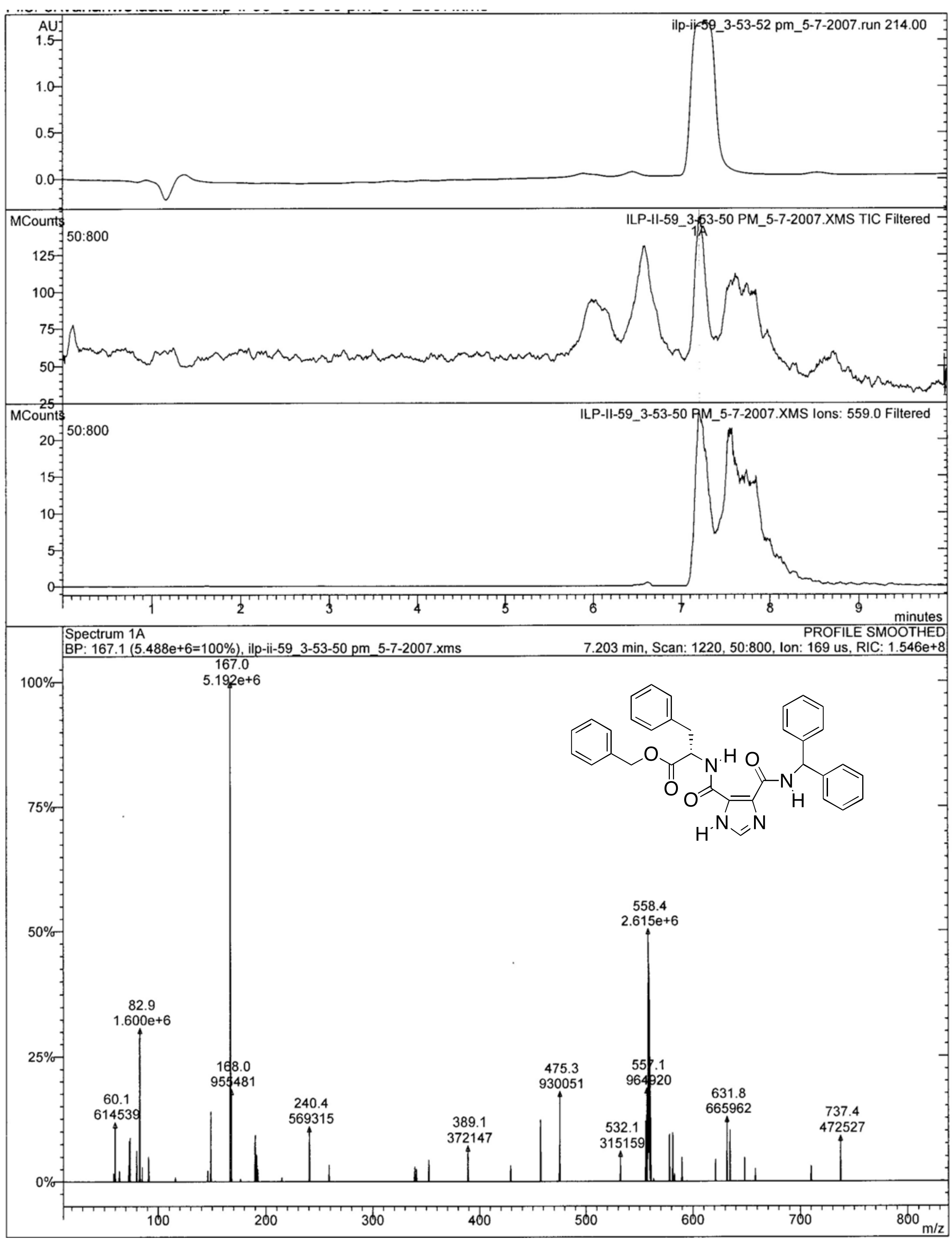


**Figure S106.** LC/MS data for **5{**106}.

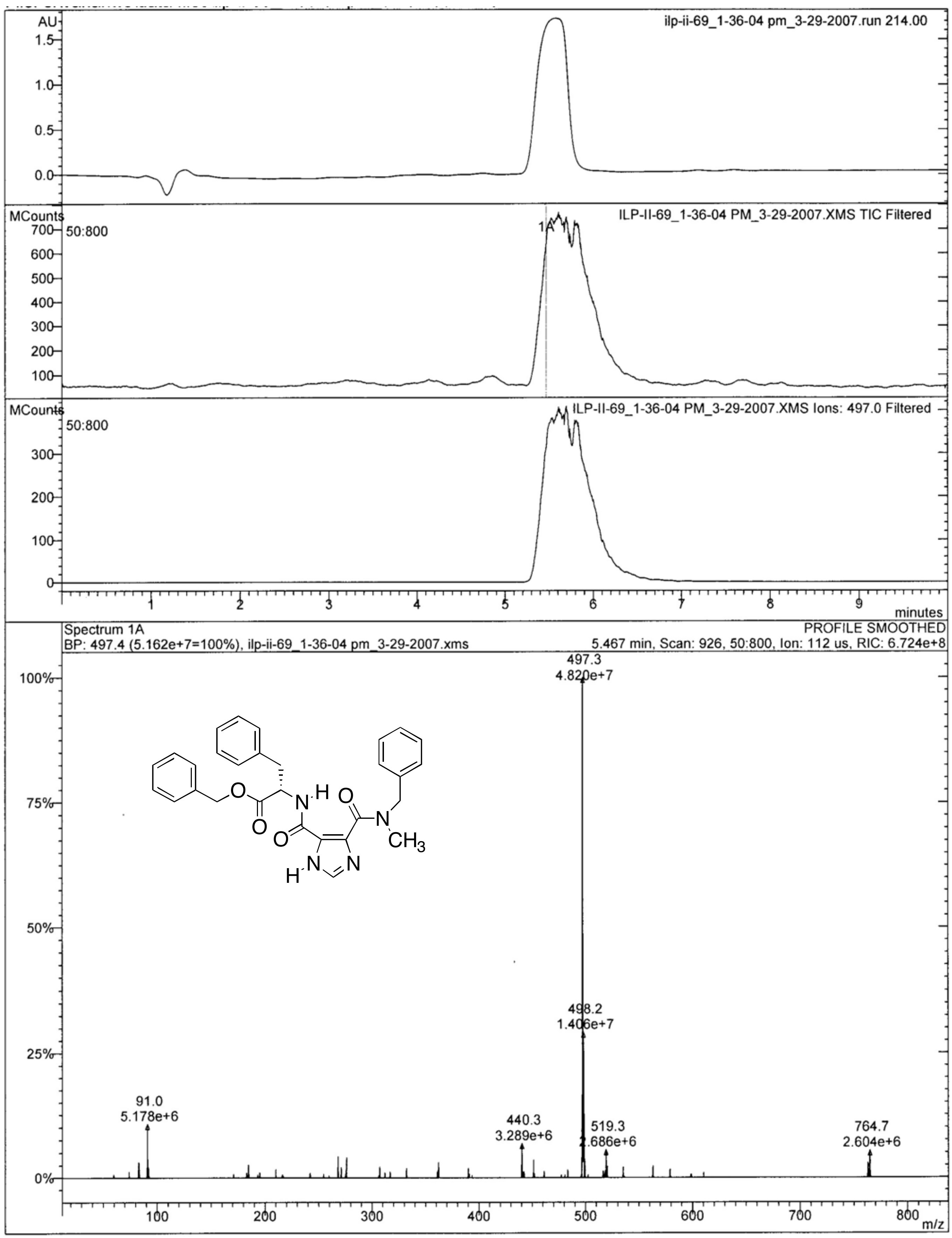


**Figure S107.** LC/MS data for **5{**107}.

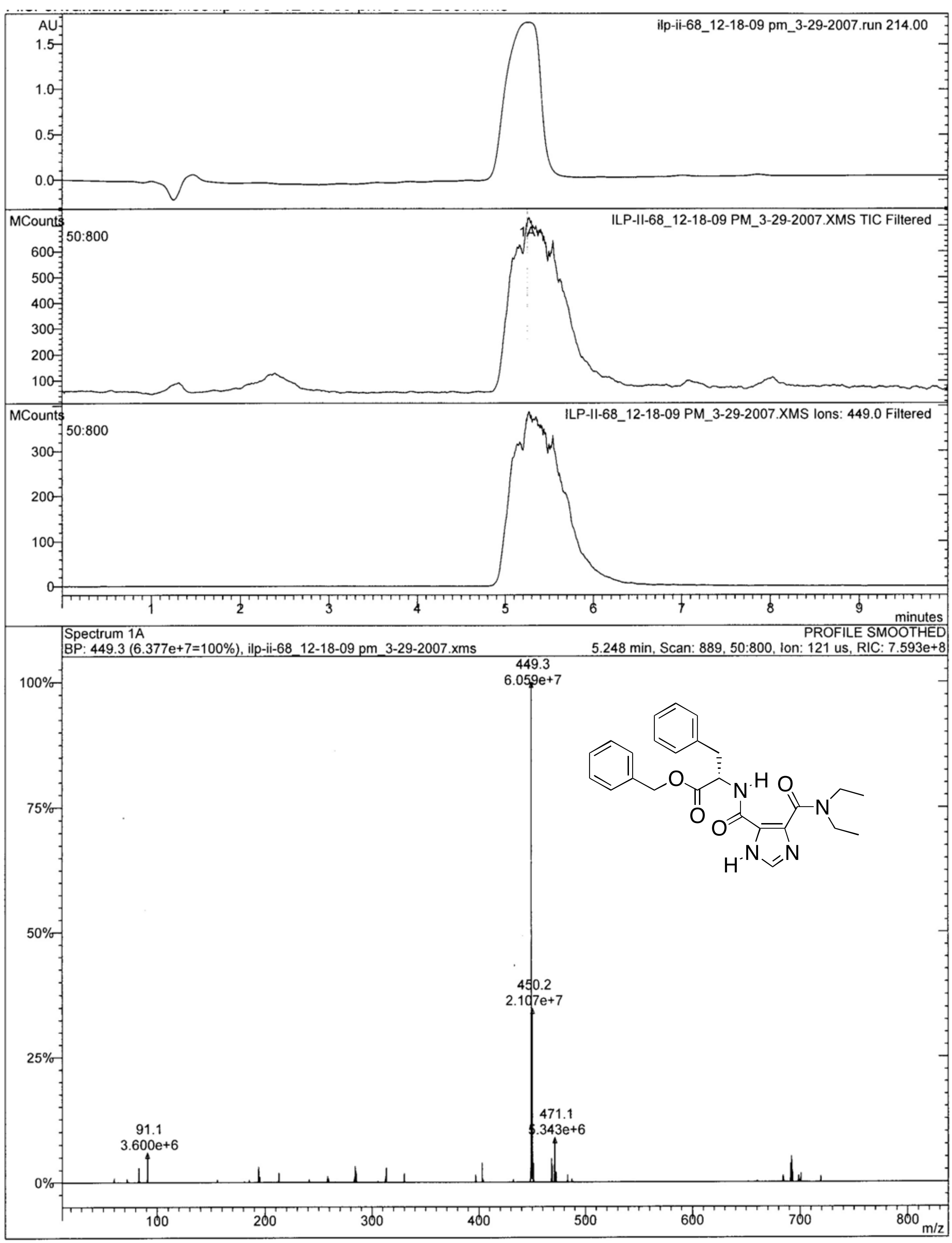


**Figure S108.** LC/MS data for **5{**108}.

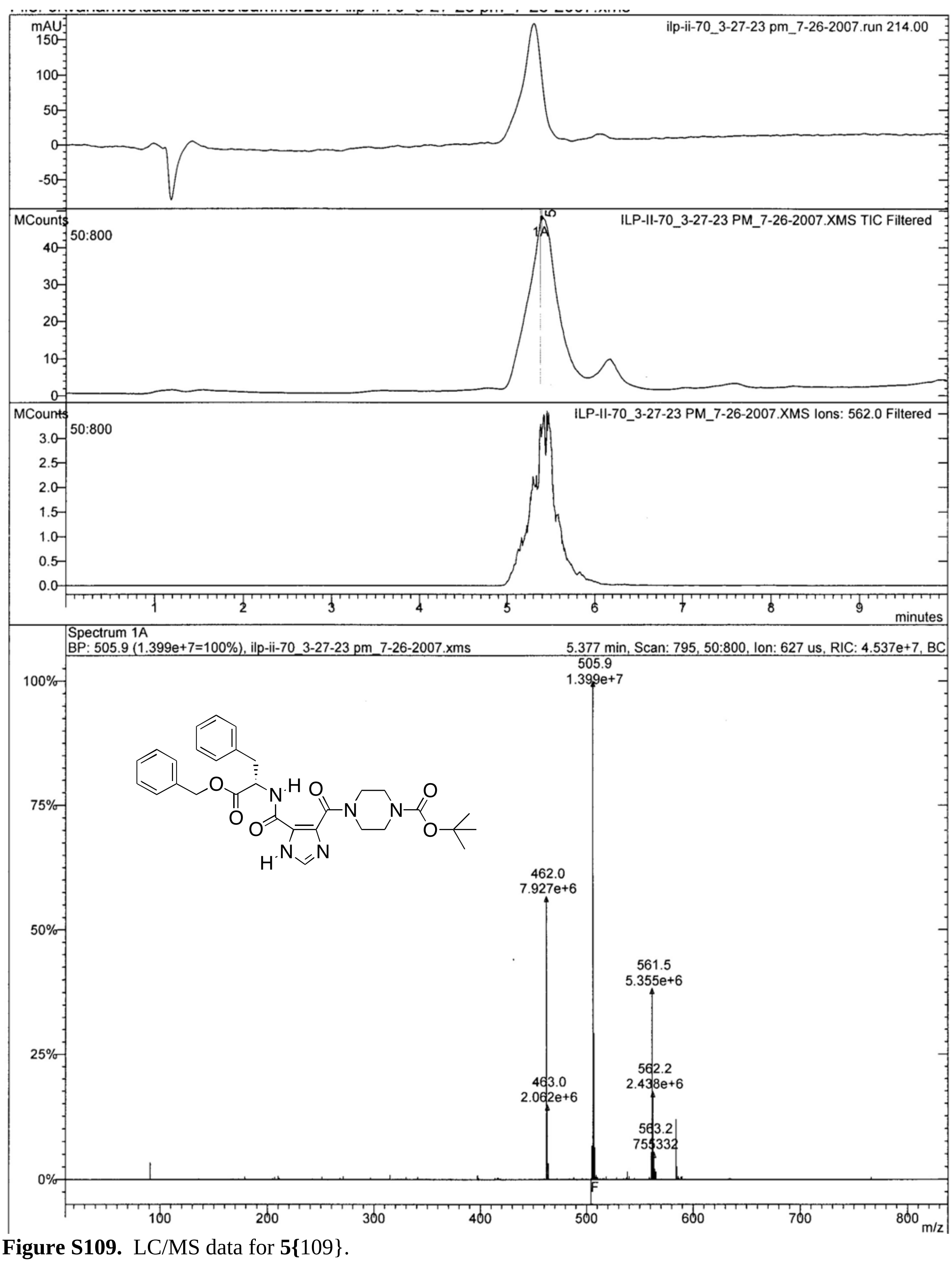


**Figure S109.** LC/MS data for **5{**109}.

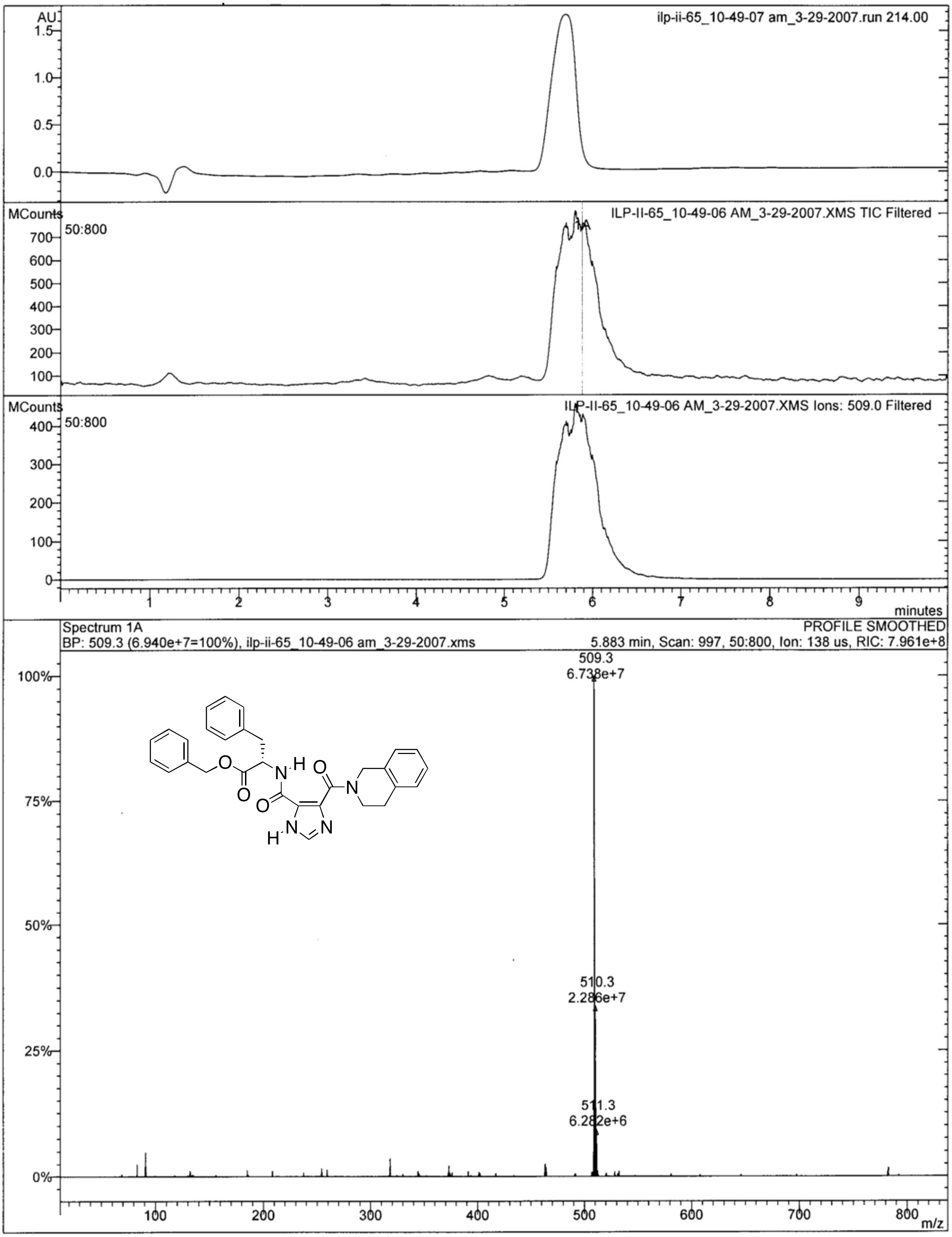


**Figure S110.** LC/MS data for **5{**110}.

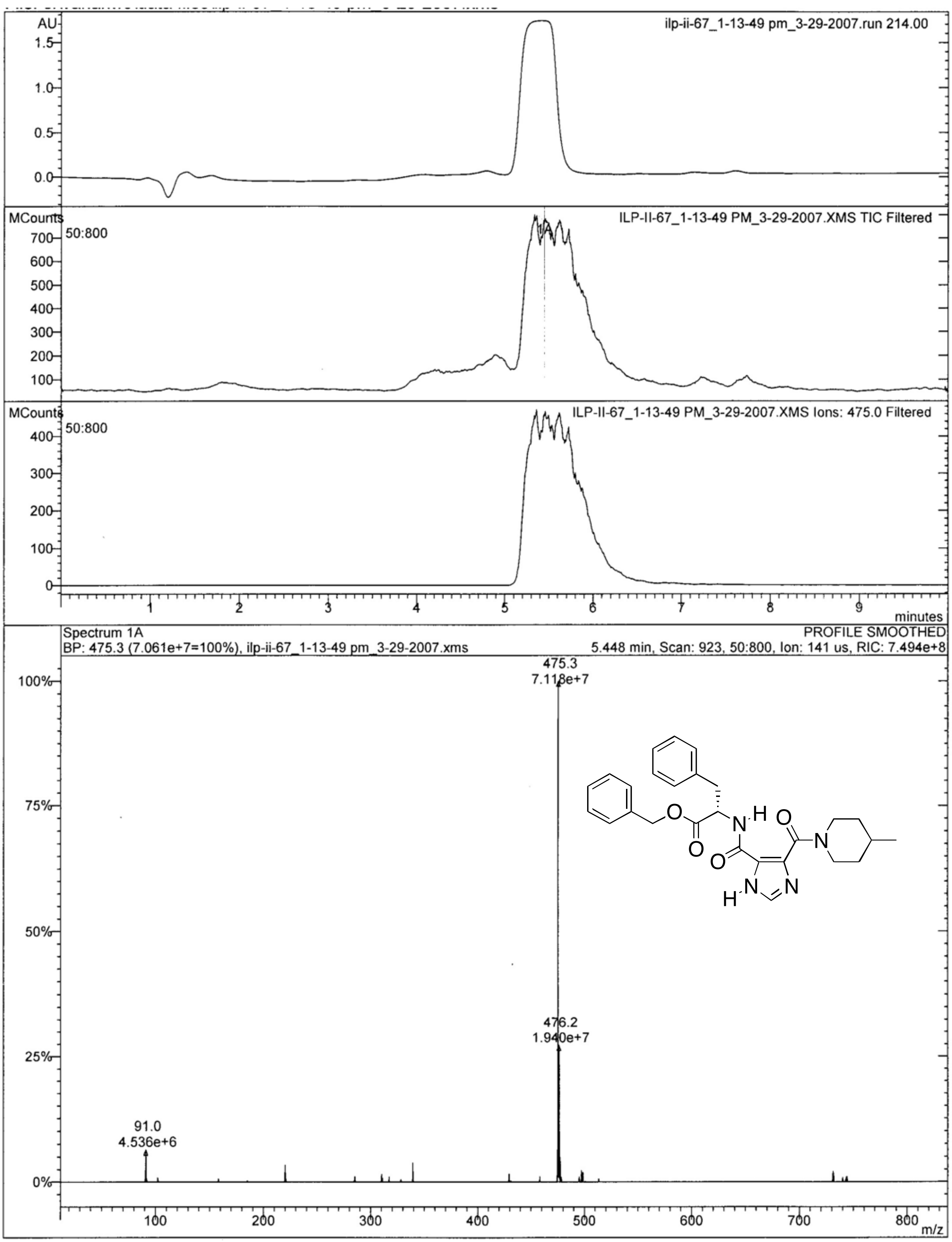


**Figure S111.** LC/MS data for **5{**111}.

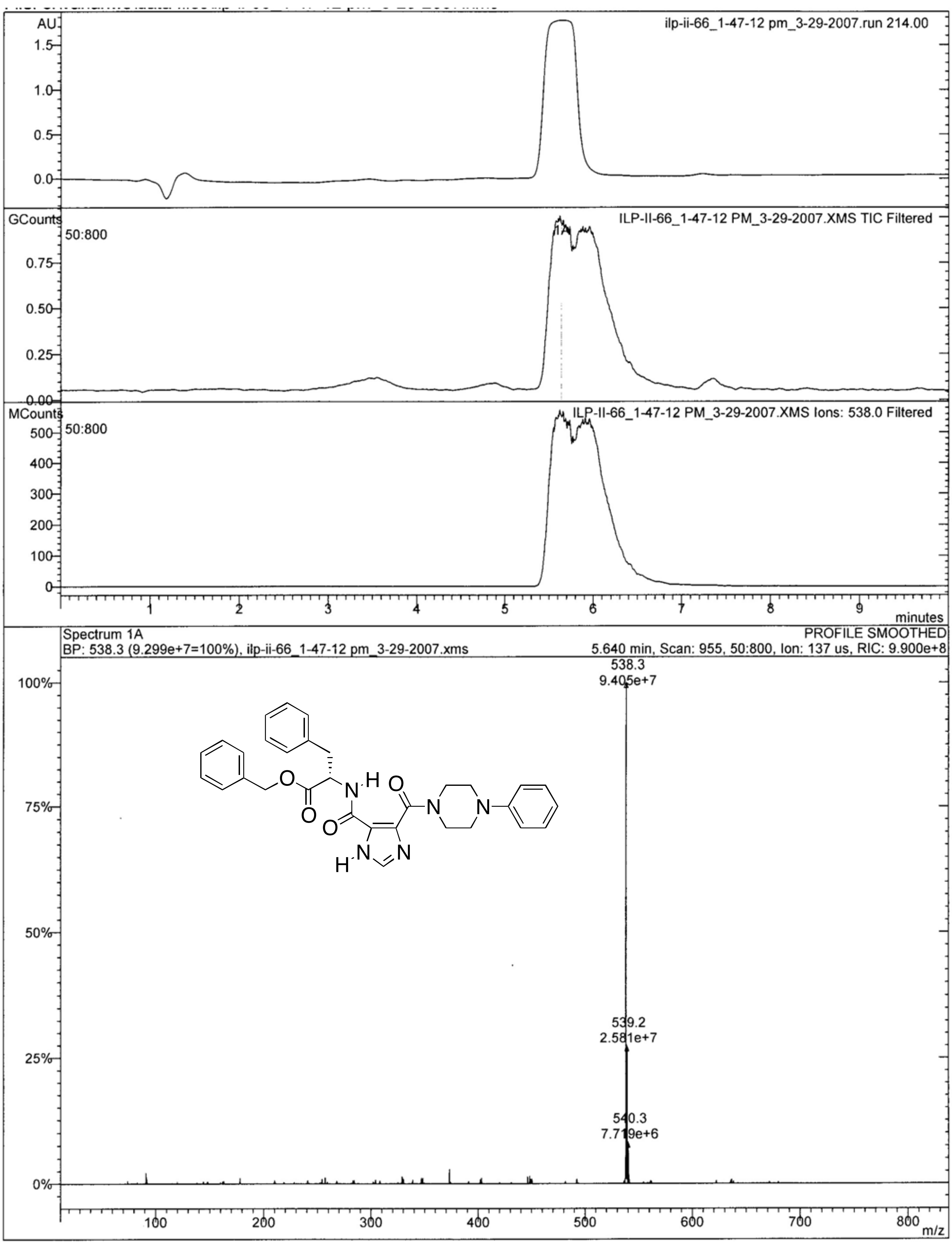


**Figure S112.** LC/MS data for **5{**112}.

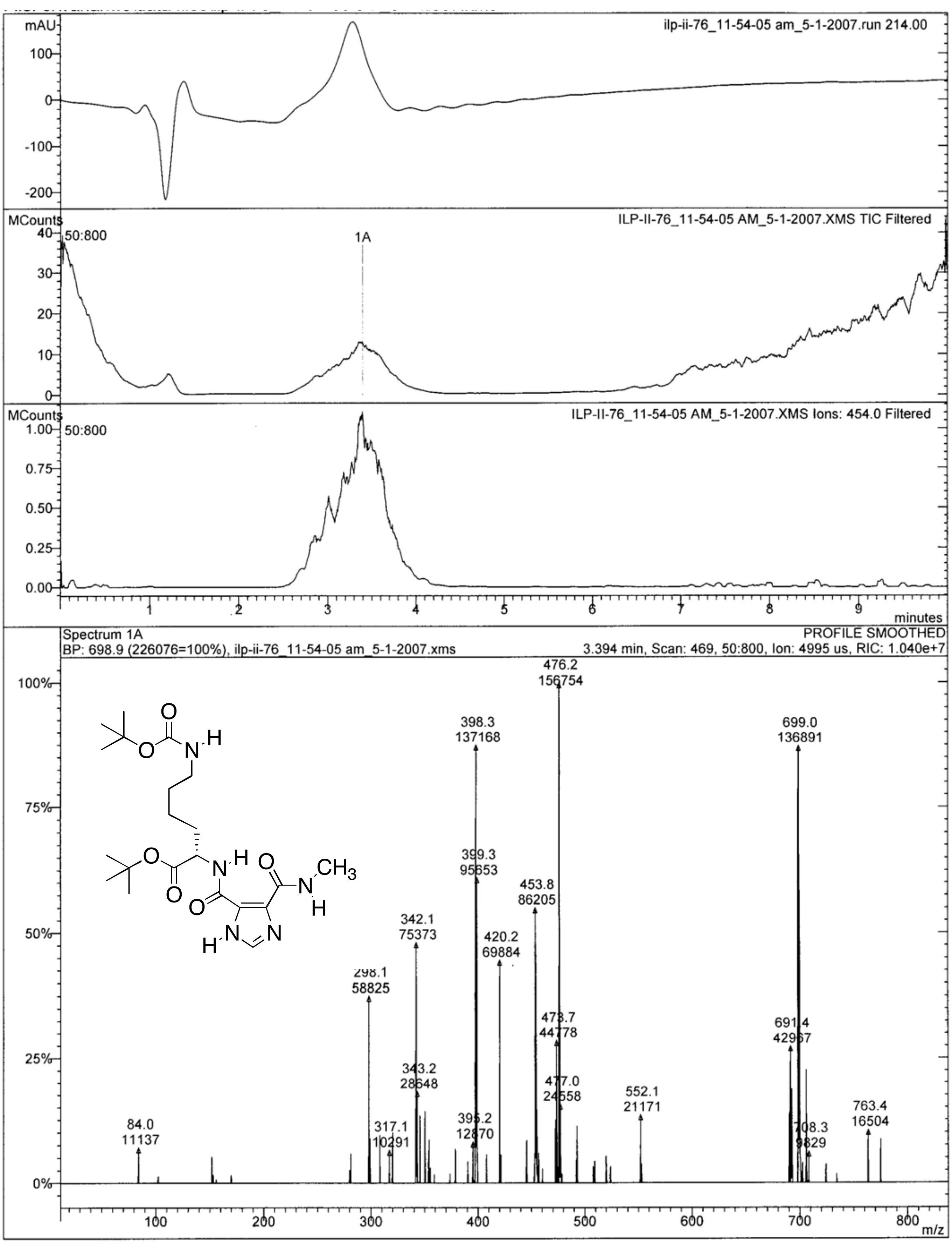


**Figure S113.** LC/MS data for **5{**113}.

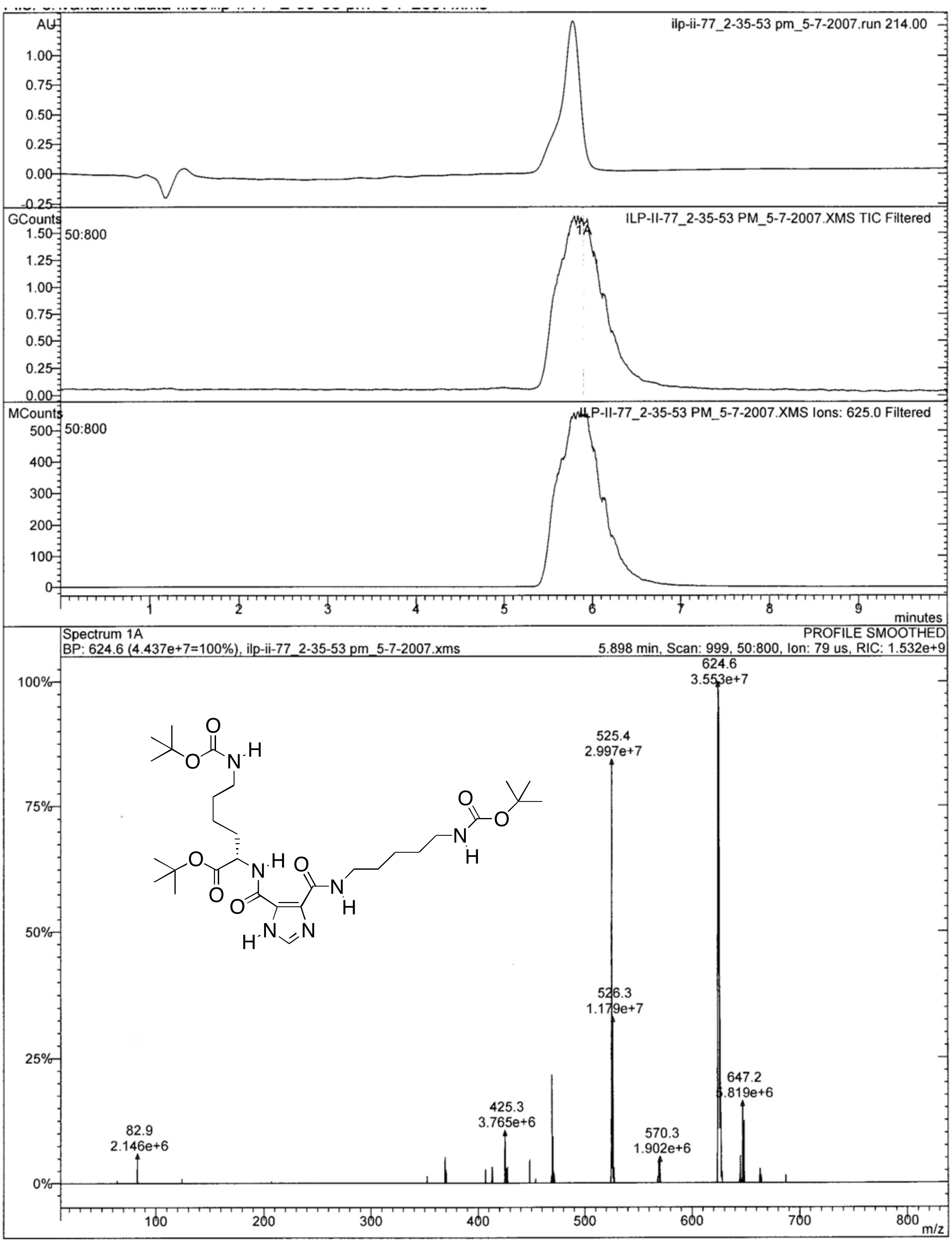


**Figure S114.** LC/MS data for **5{**114}.

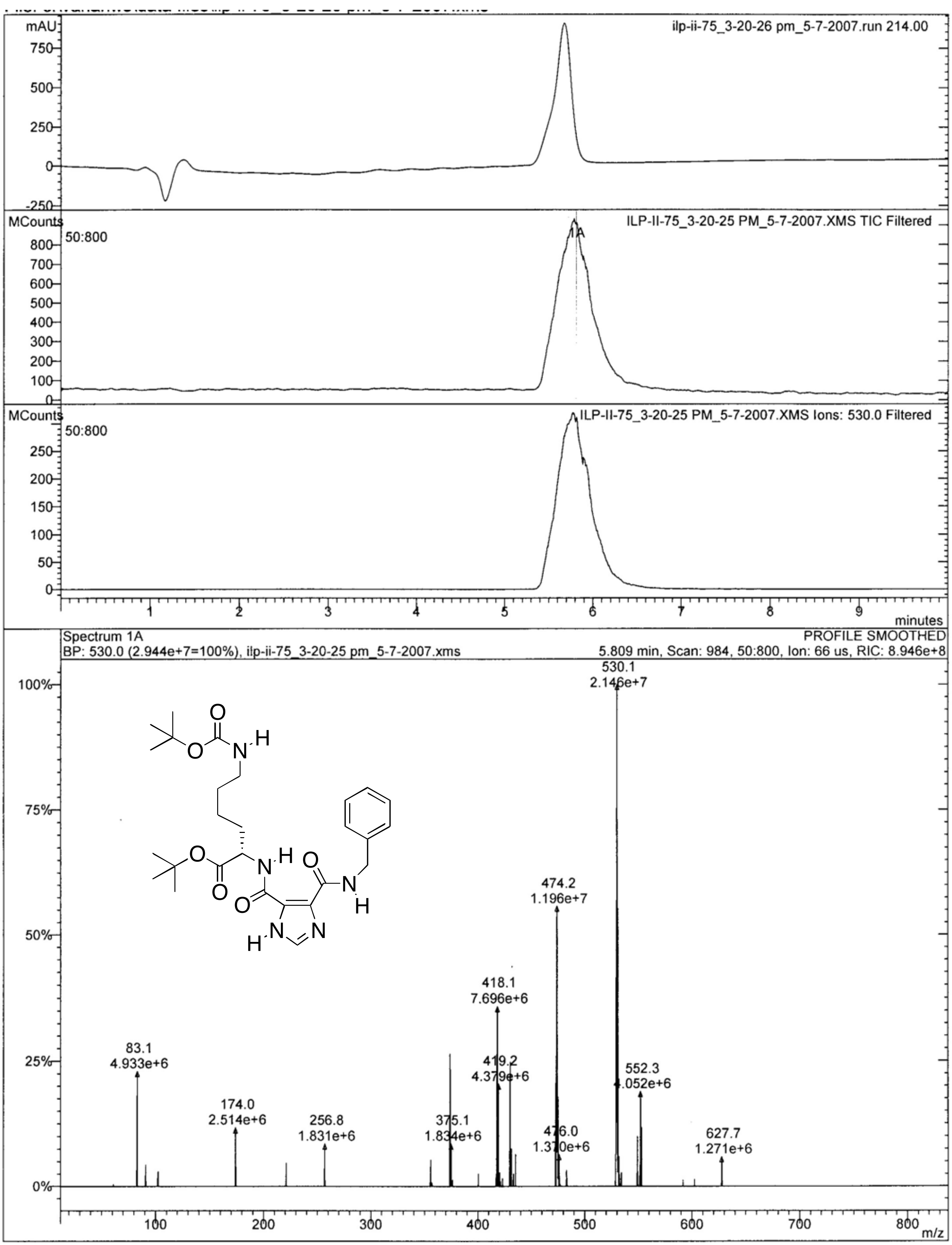


**Figure S115.** LC/MS data for **5{**115}.

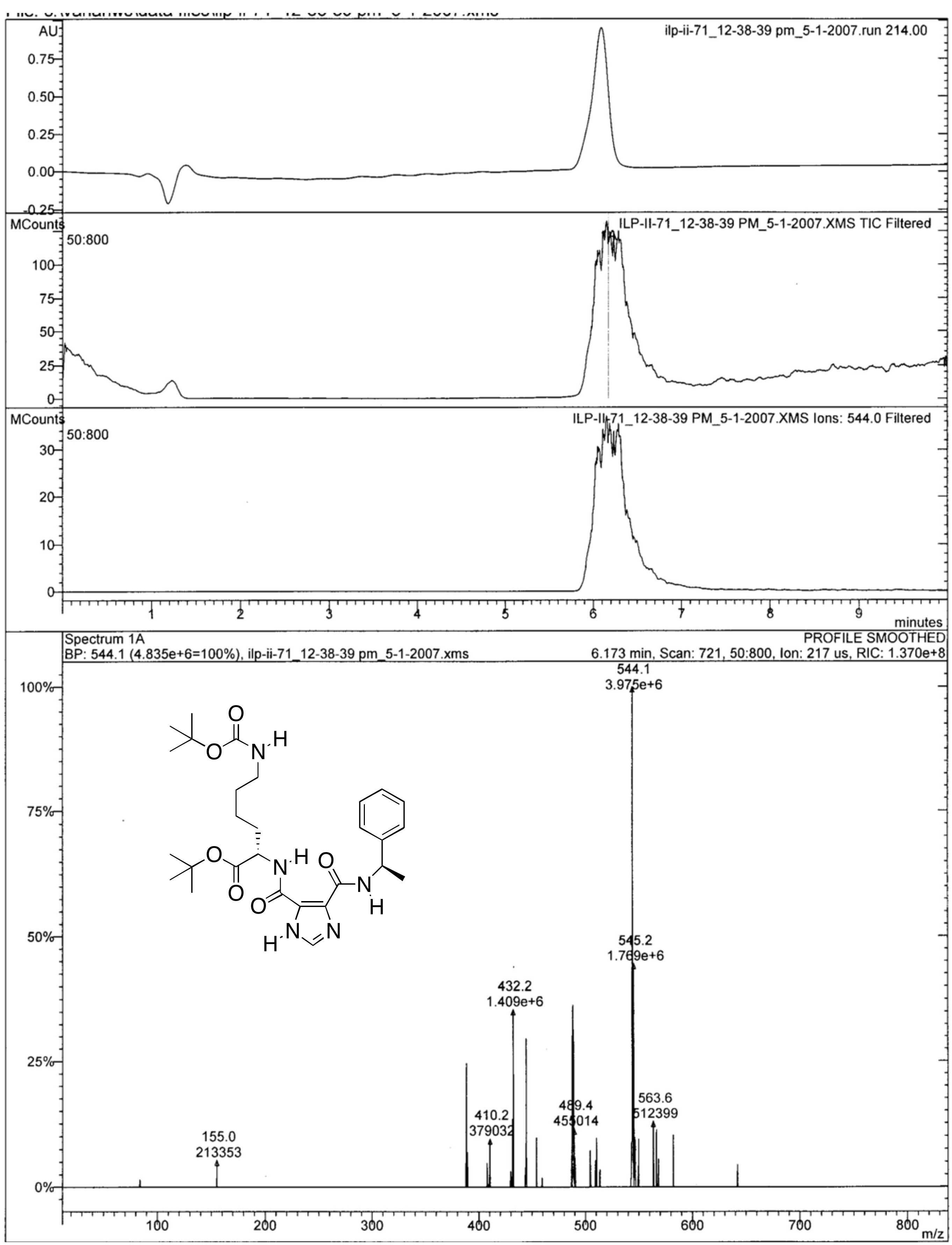


**Figure S116.** LC/MS data for **5{**116}.

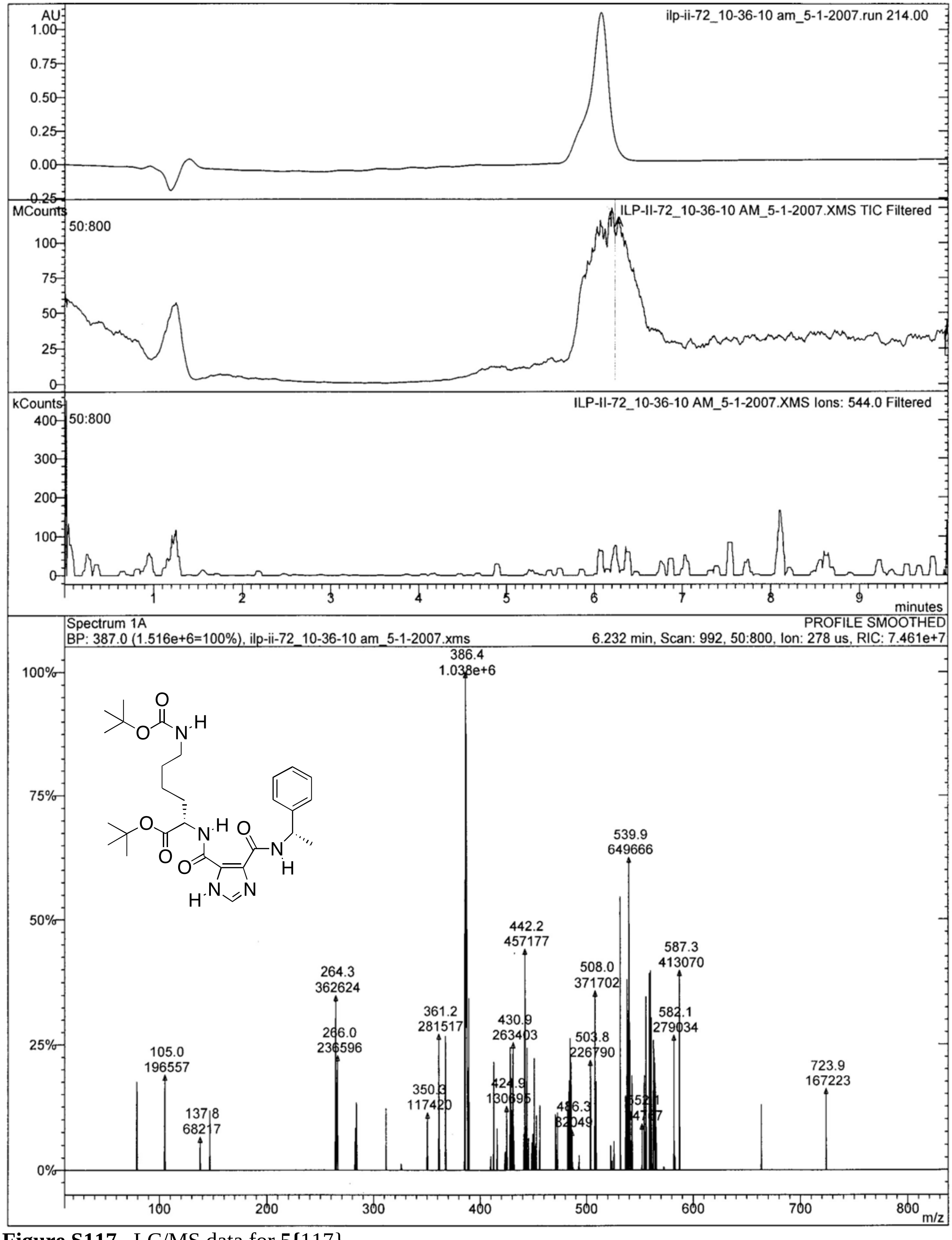


**Figure S117.** LC/MS data for **5{**117}.

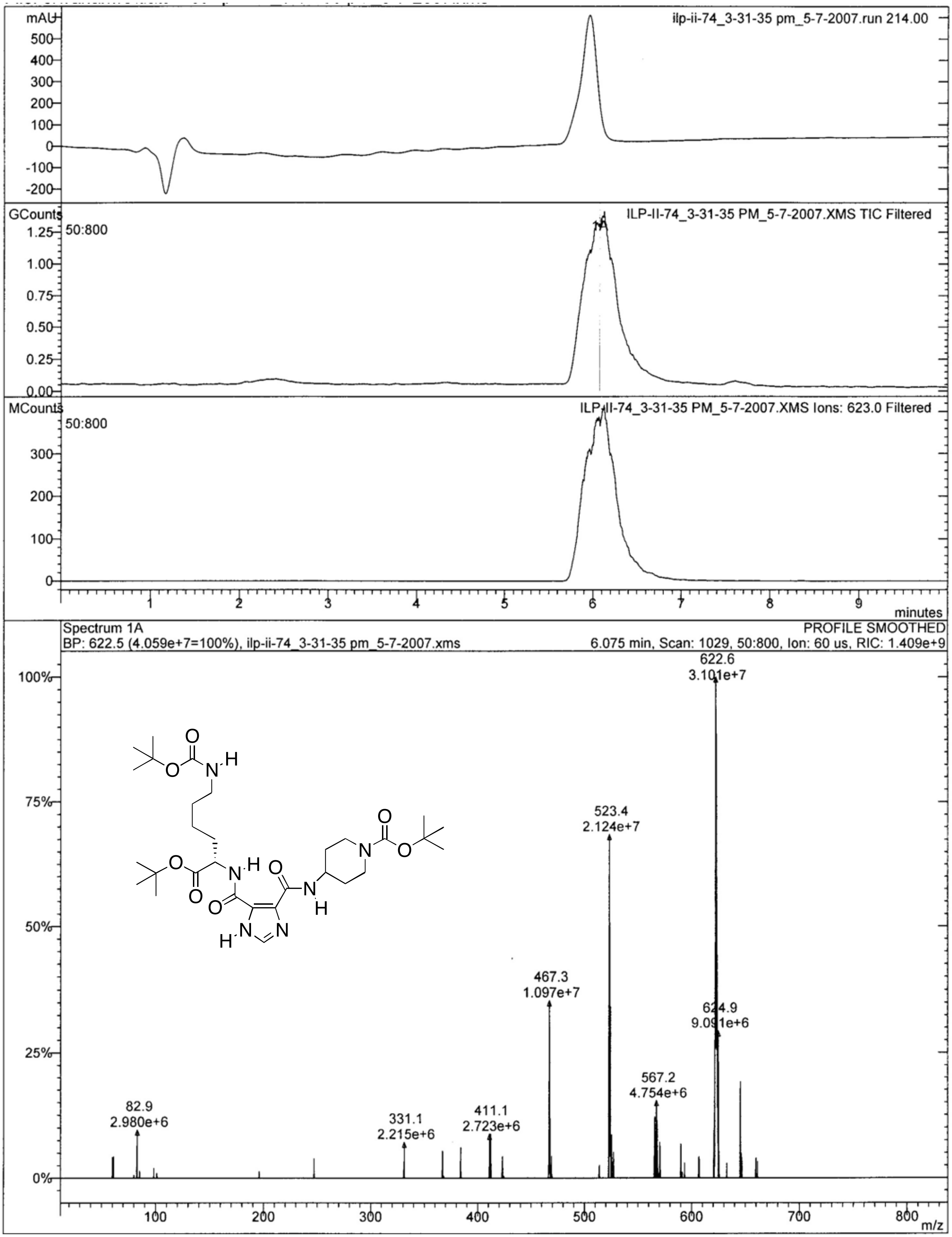


**Figure S118.** LC/MS data for **5{**118}.

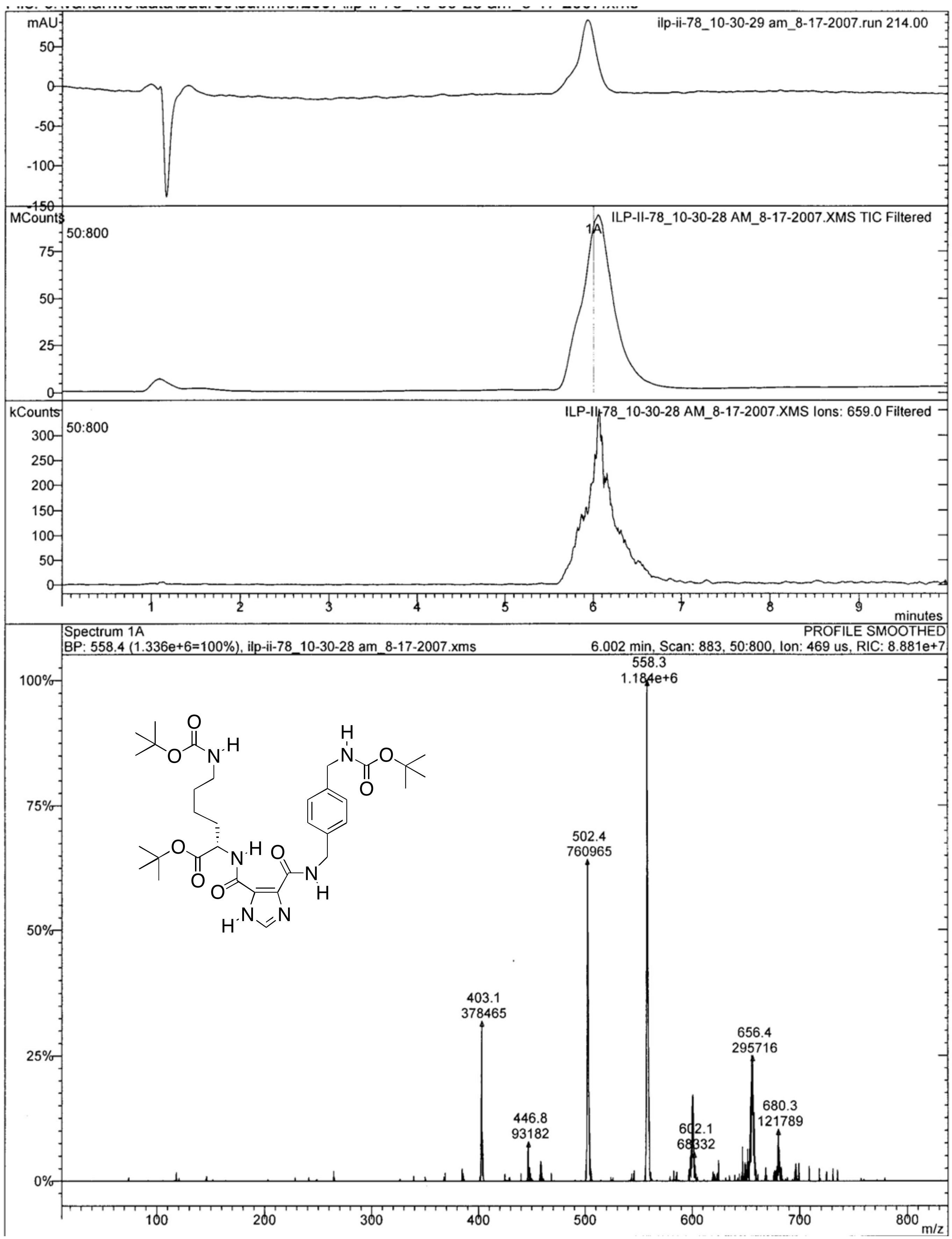


**Figure S119.** LC/MS data for **5{**119}.

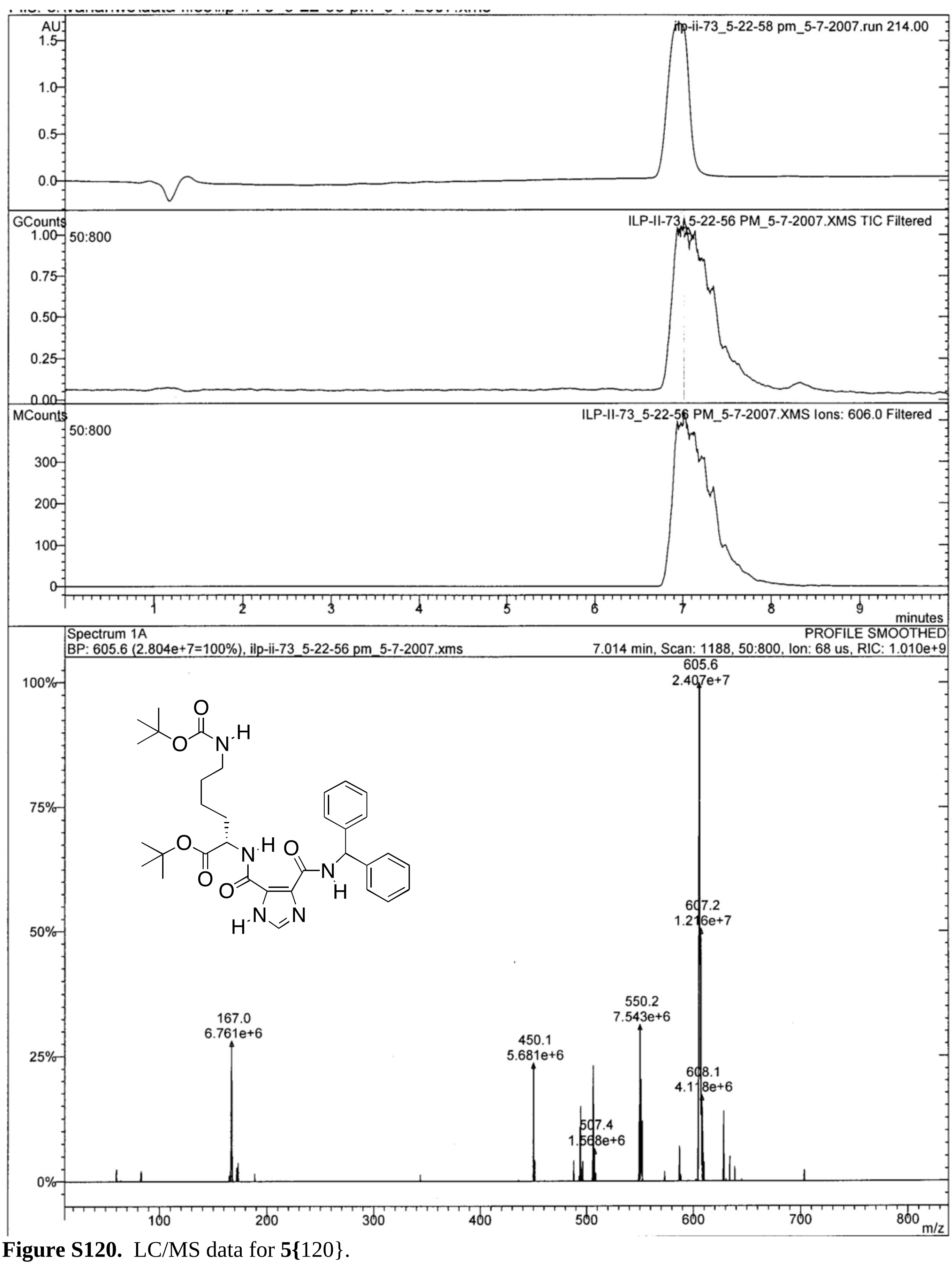


**Figure S120.** LC/MS data for **5{**120}.

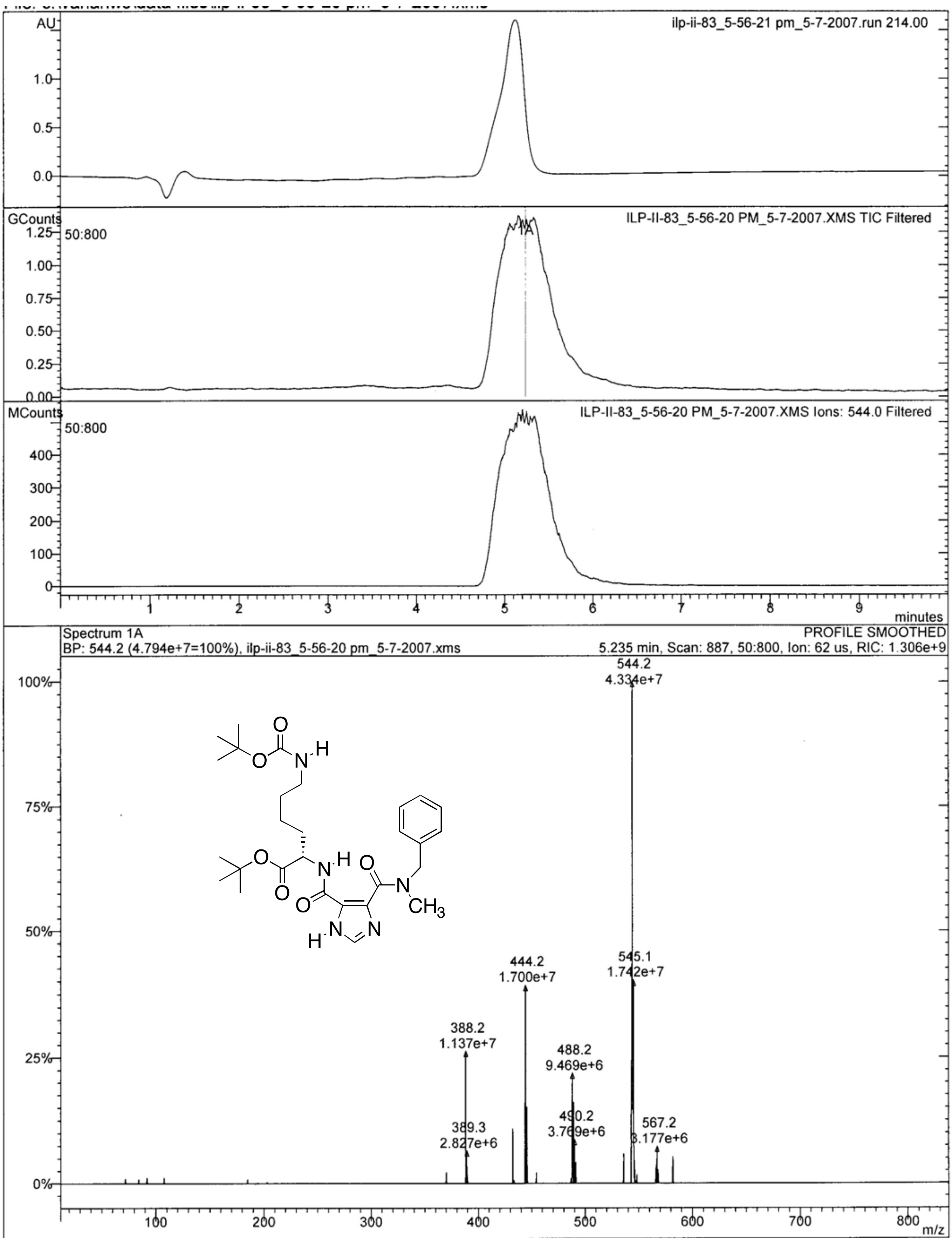


**Figure S121.** LC/MS data for **5{**121}.

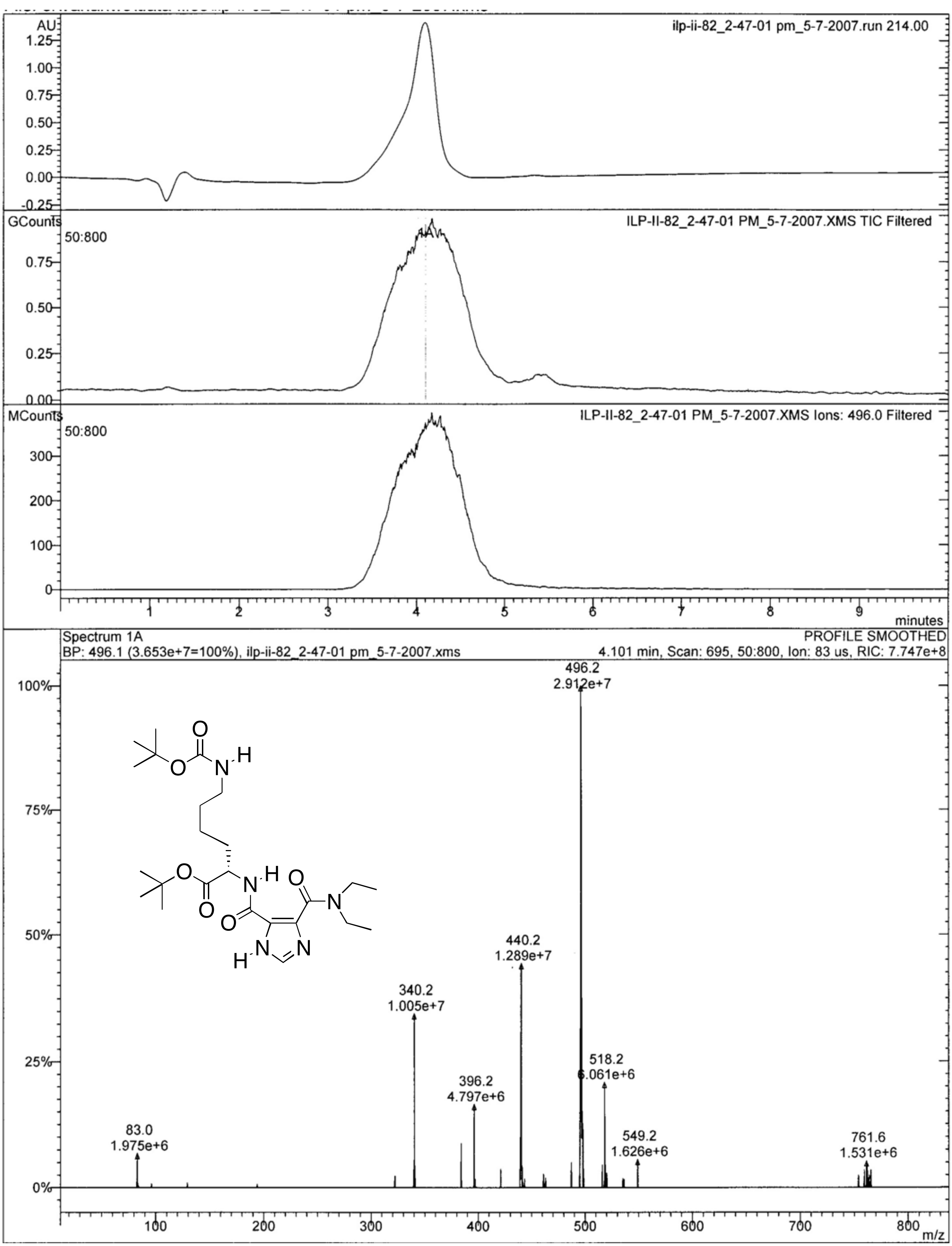


**Figure S122.** LC/MS data for **5{**122}.

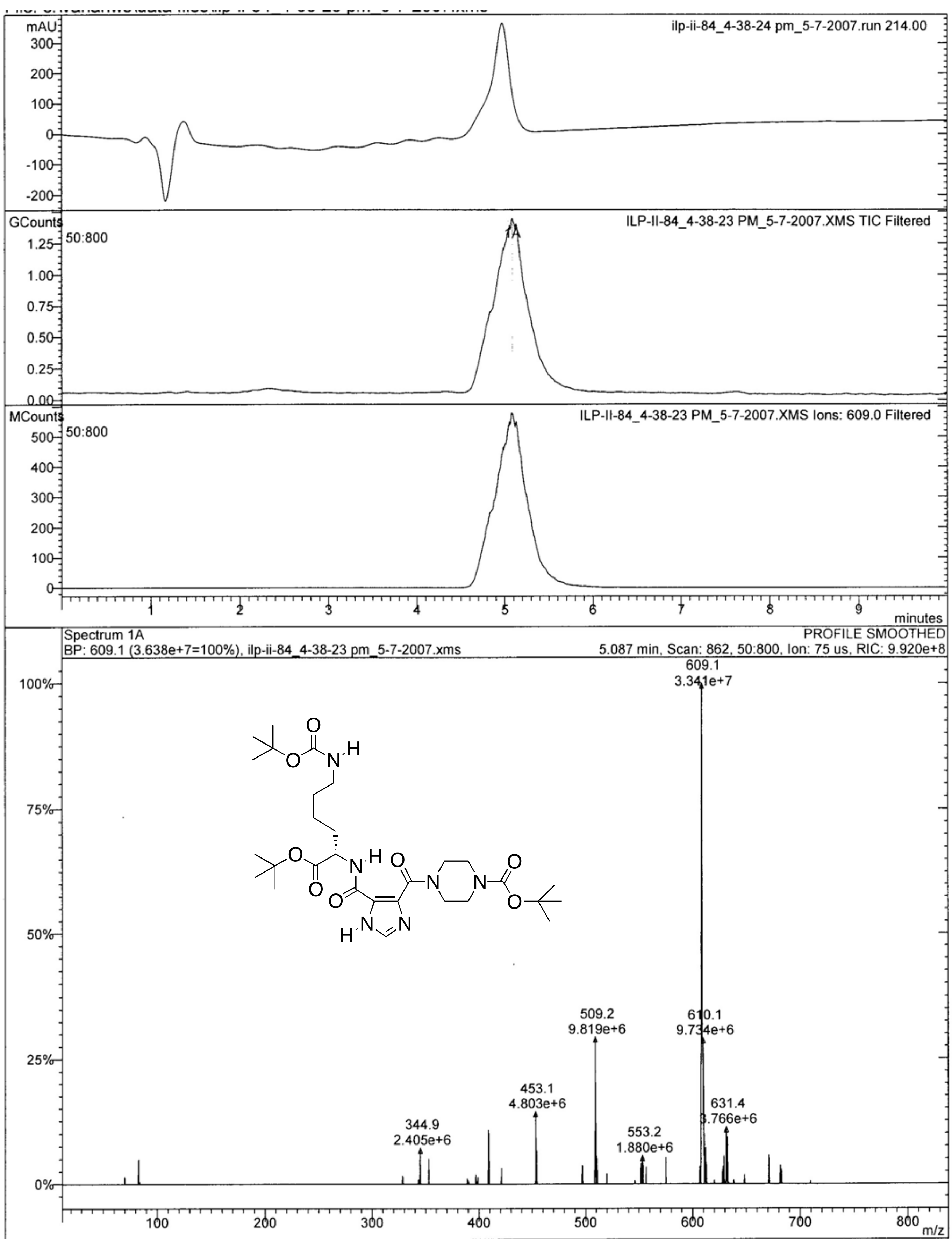


**Figure S123.** LC/MS data for **5{**123}.

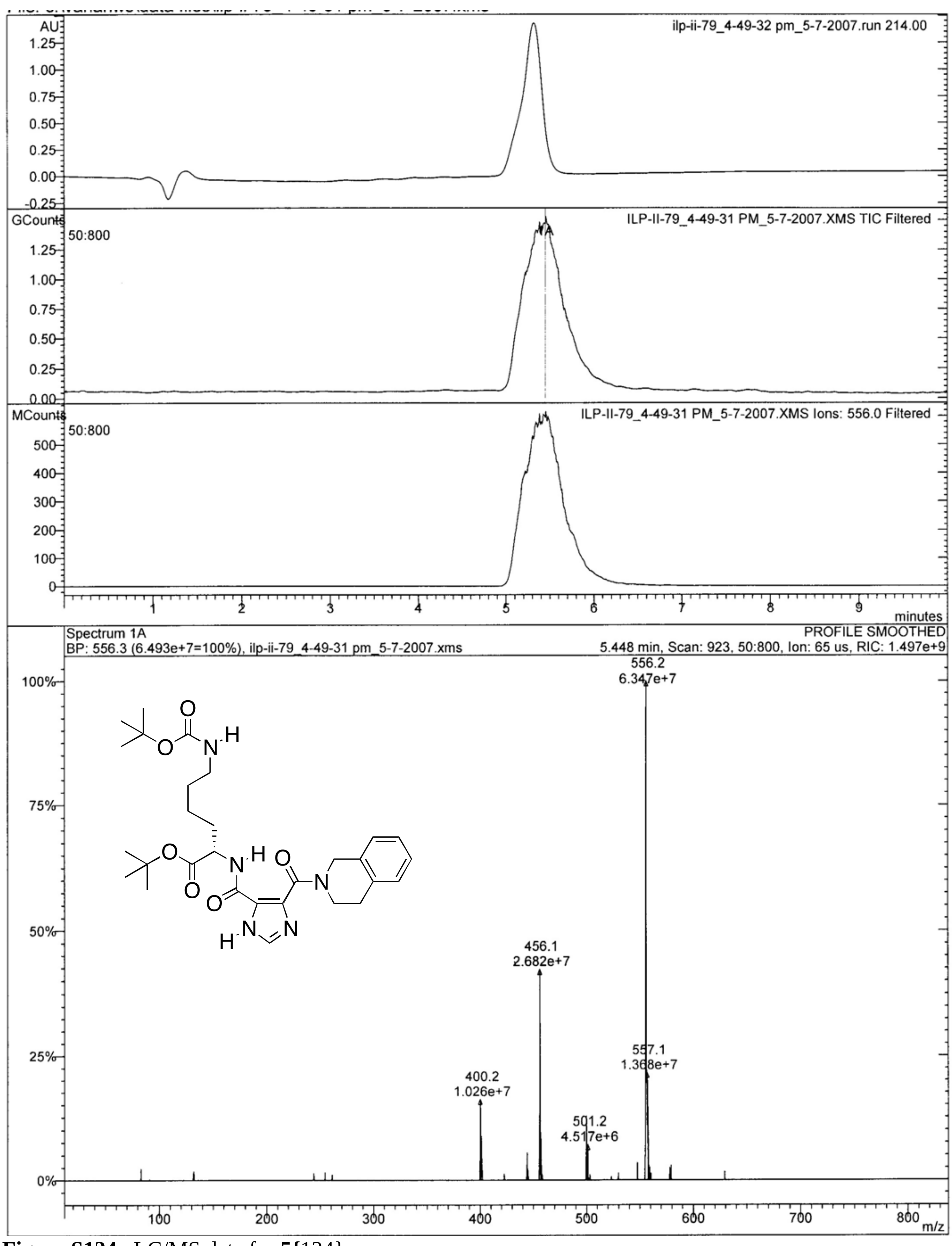


**Figure S124.** LC/MS data for **5{**124}.

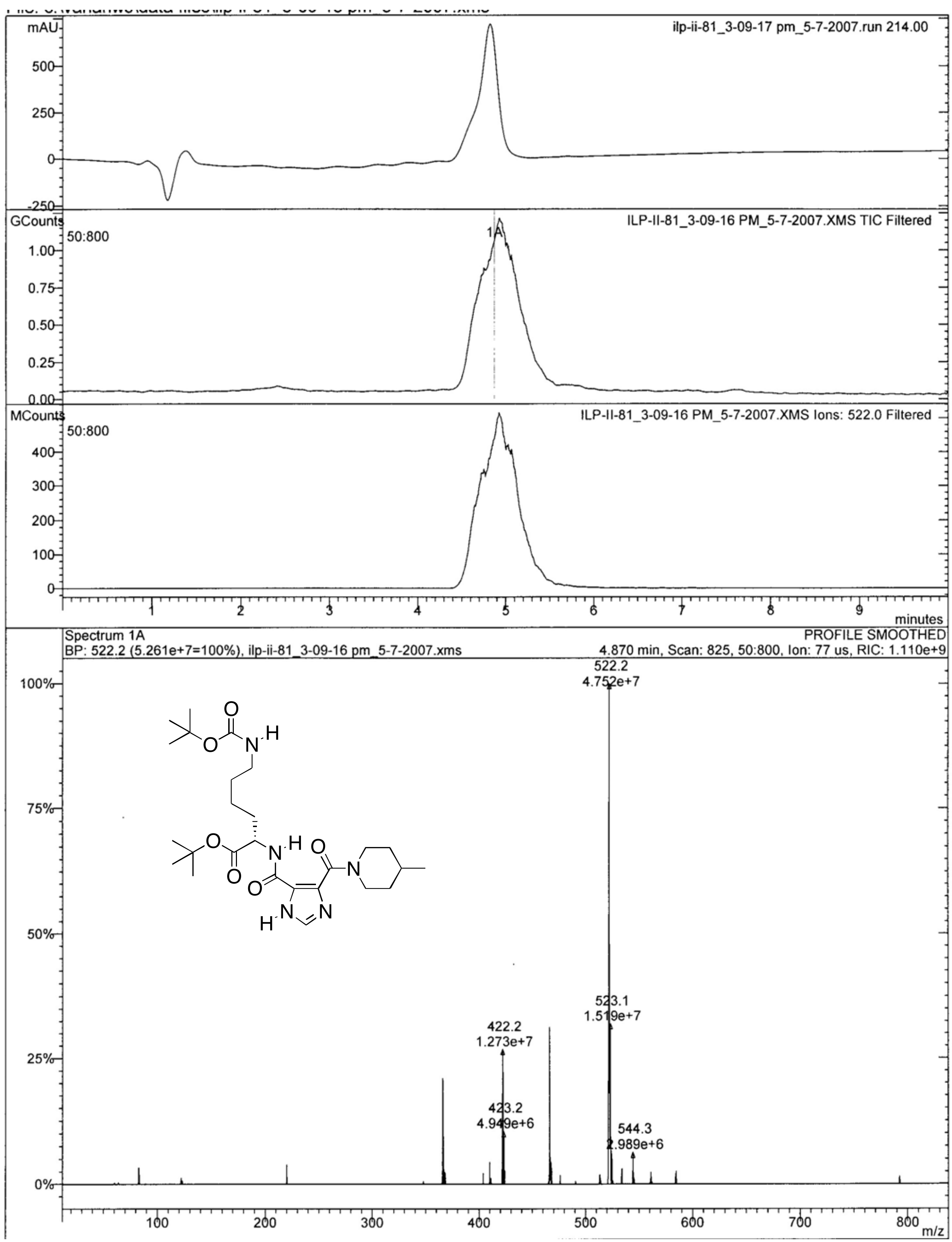


**Figure S125.** LC/MS data for **5{**125}.

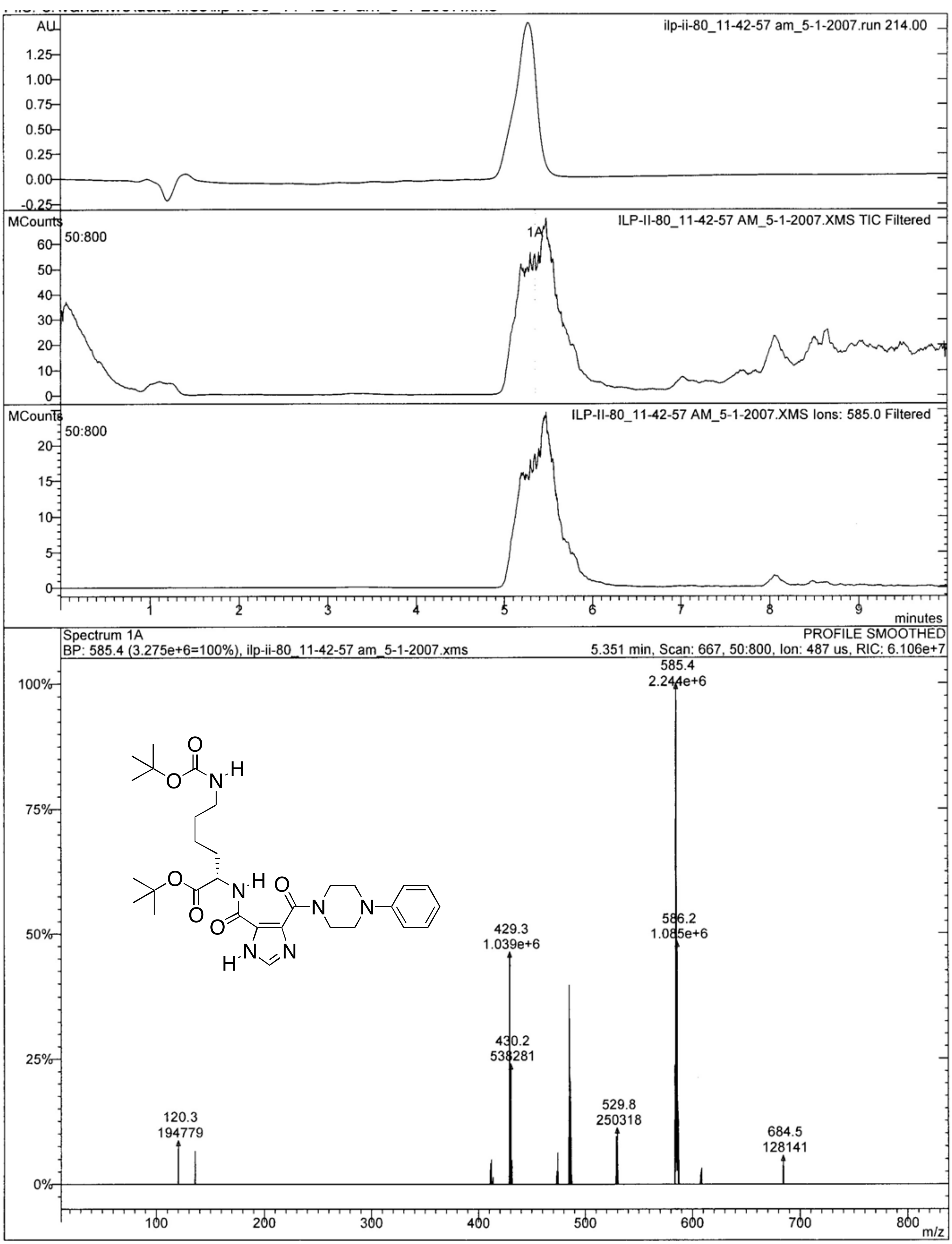


**Figure S126.** LC/MS data for **5{**126}.

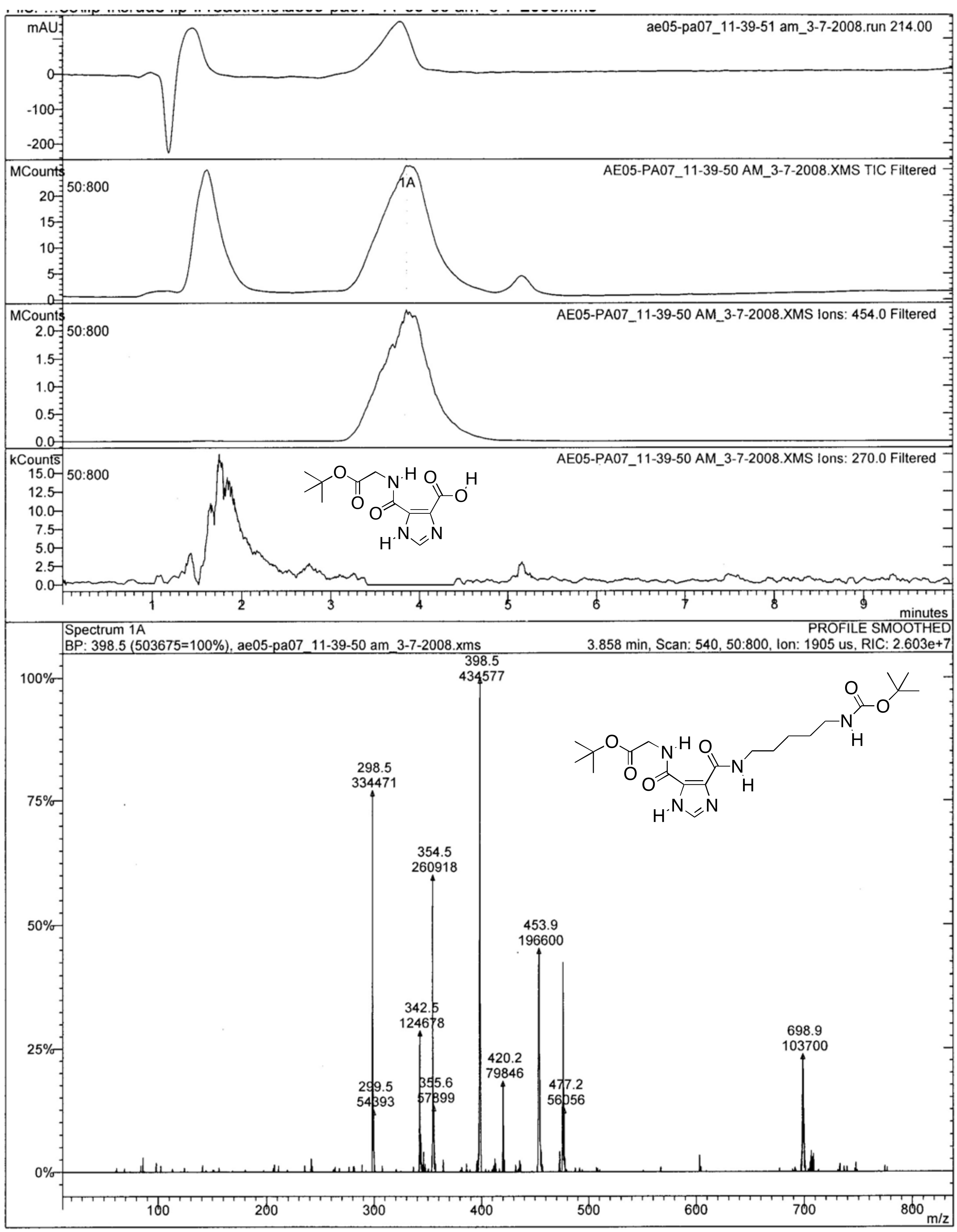


**Figure S127.** LC/MS data for the crude reaction to yield **5{**2}.

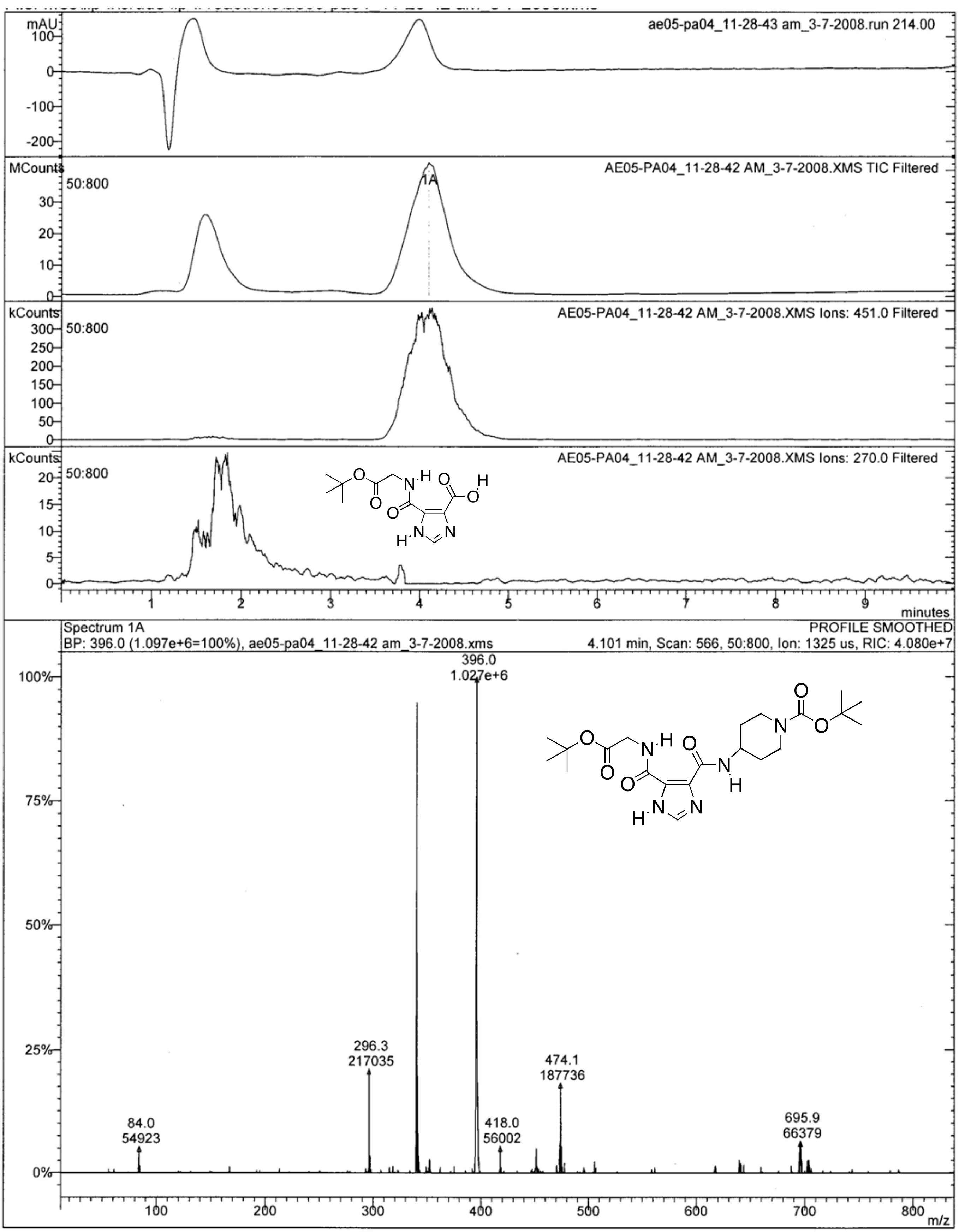


**Figure S128.** LC/MS data for the crude reaction to yield **5{**6}.

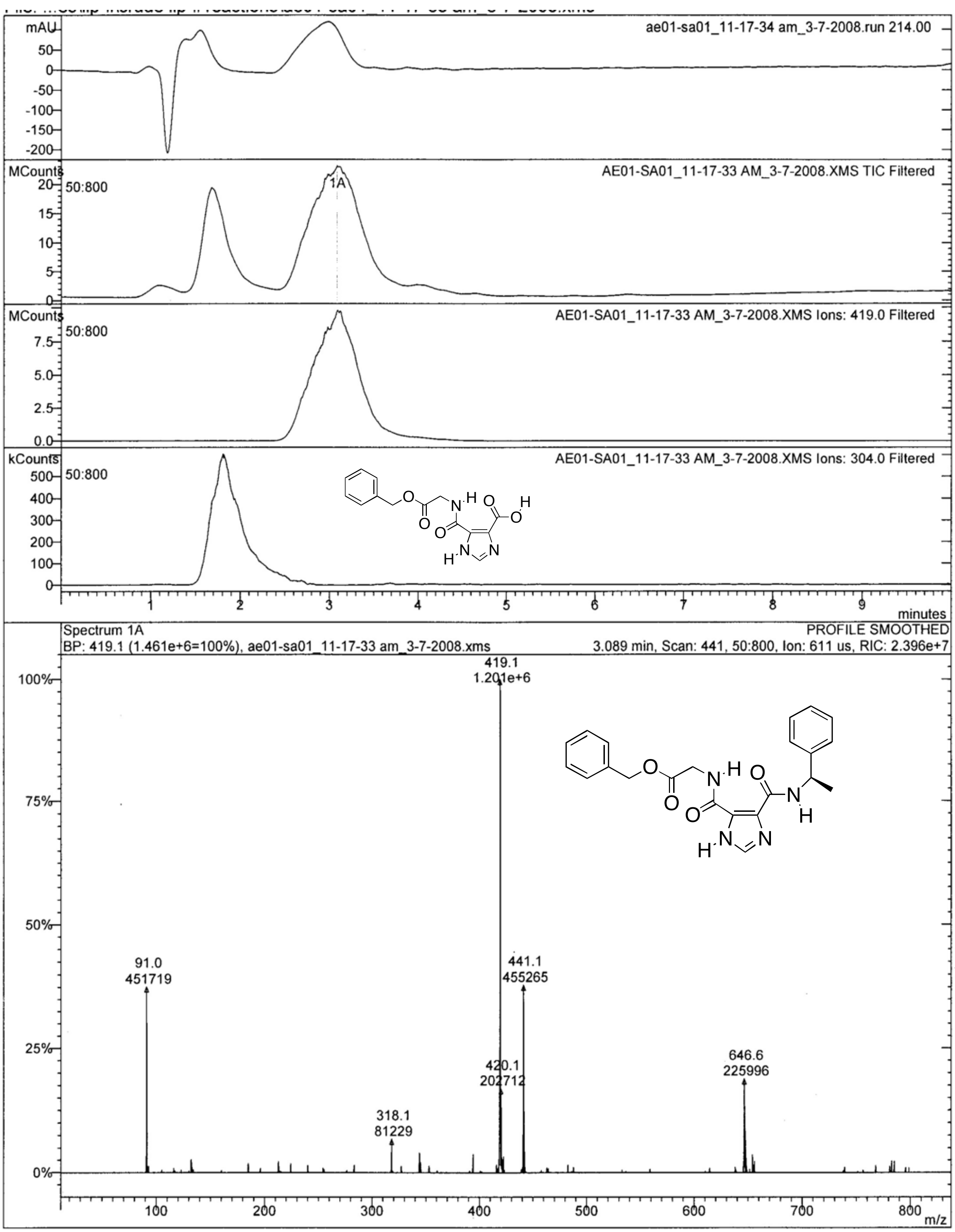


**Figure S129.** LC/MS data for the crude reaction to yield **5{**26}.

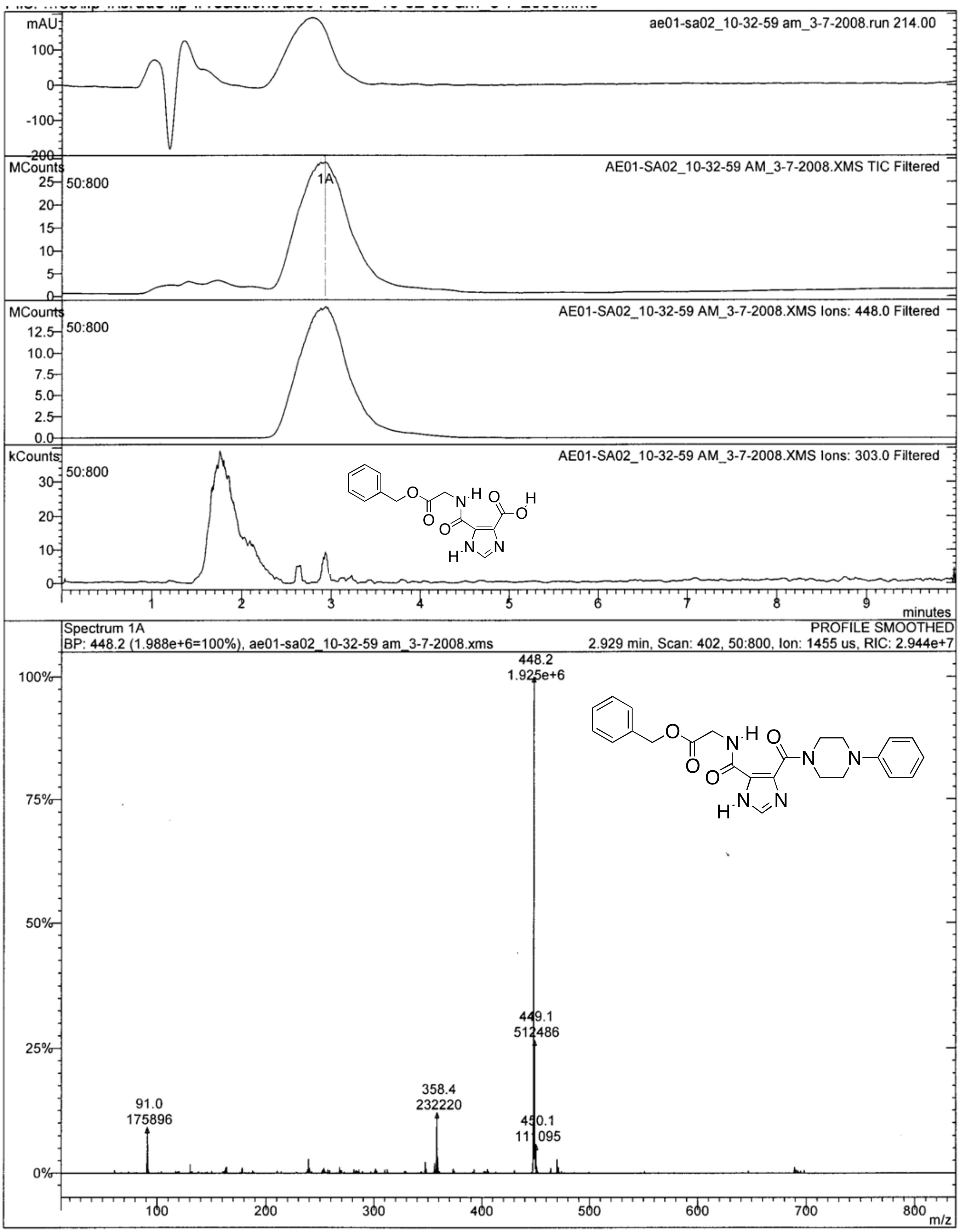


**Figure S130.** LC/MS data for the crude reaction to yield **5{**28}.

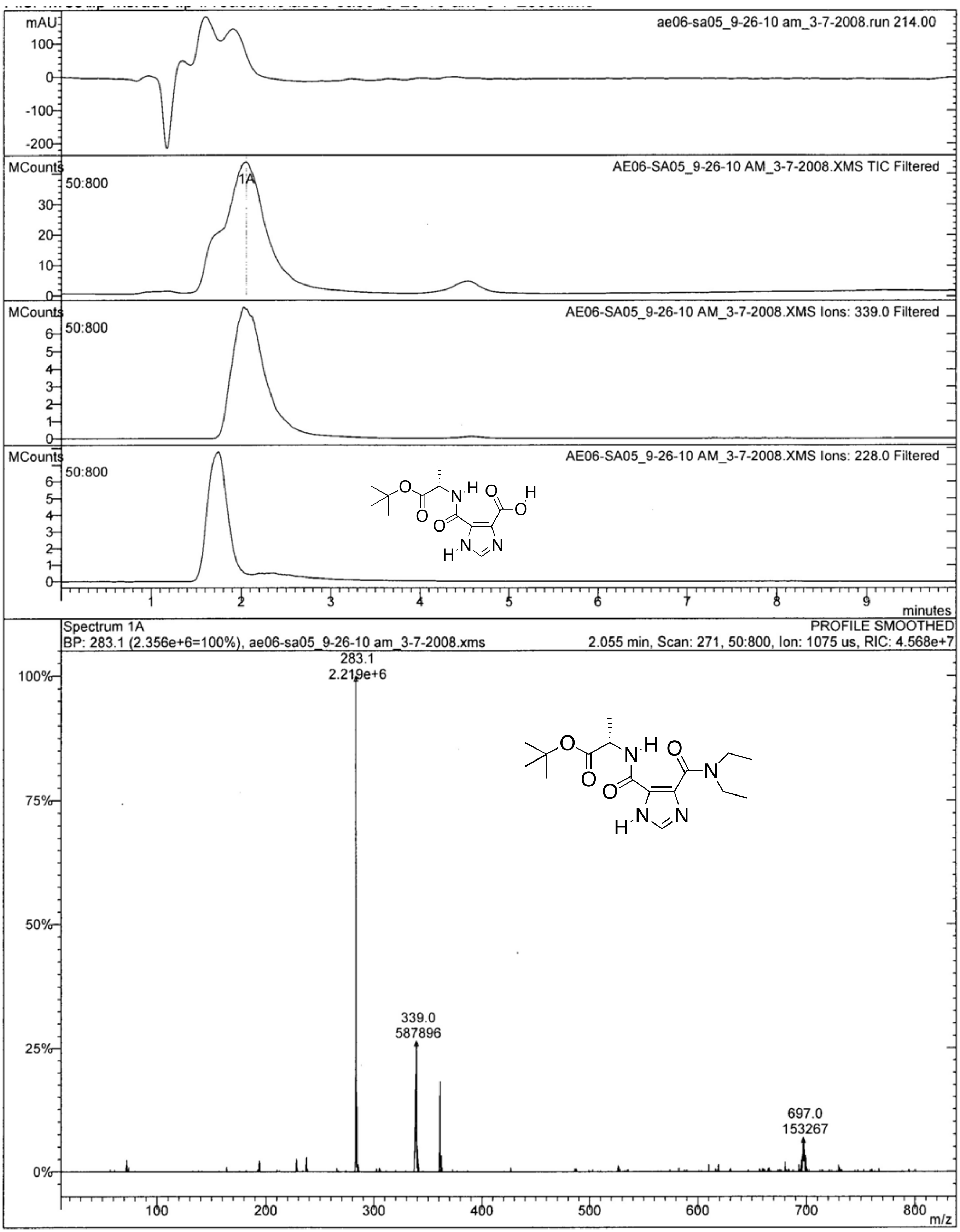


**Figure S131.** LC/MS data for the crude reaction to yield **5{**38}.

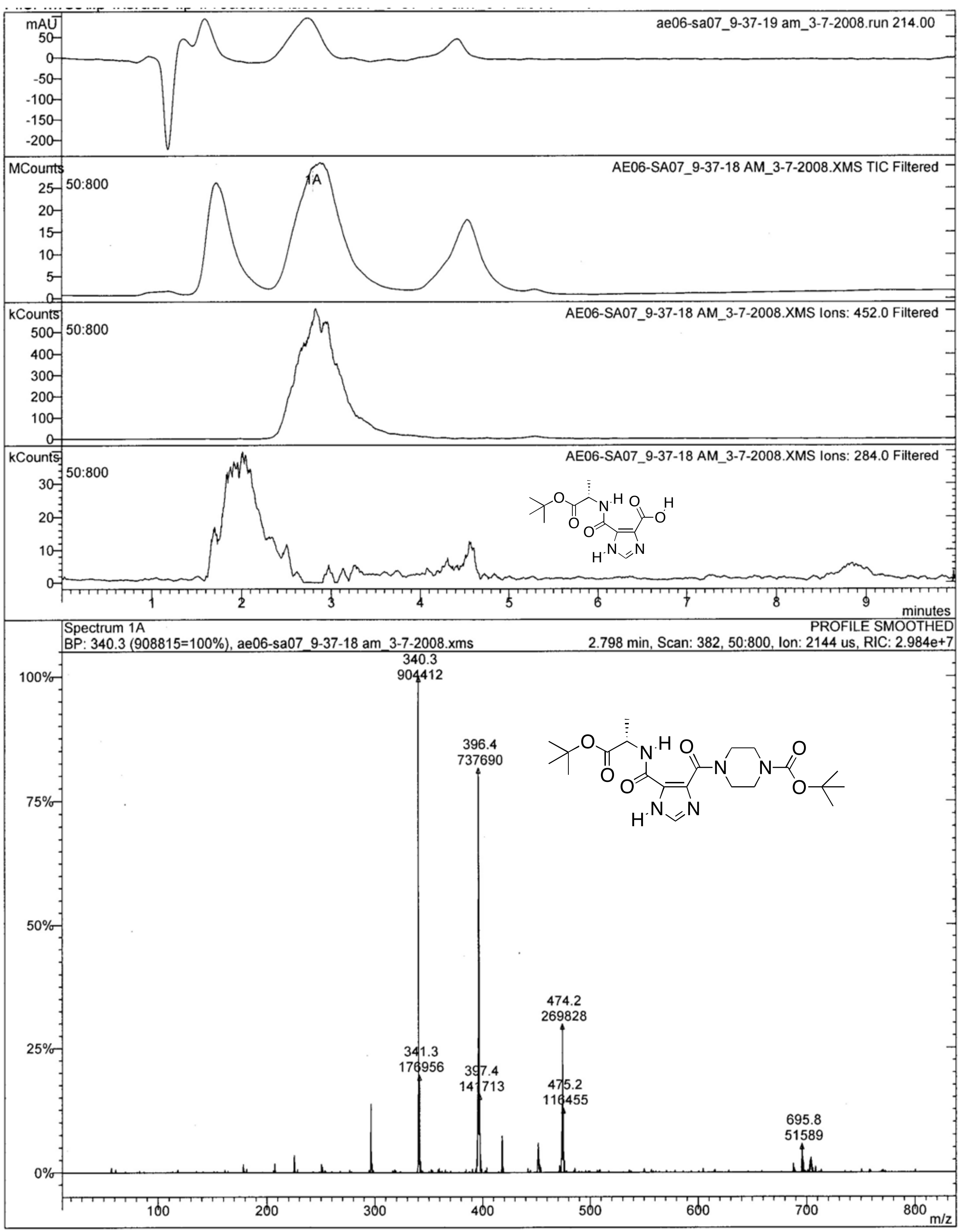


**Figure S132.** LC/MS data for the crude reaction to yield **5{**39}.

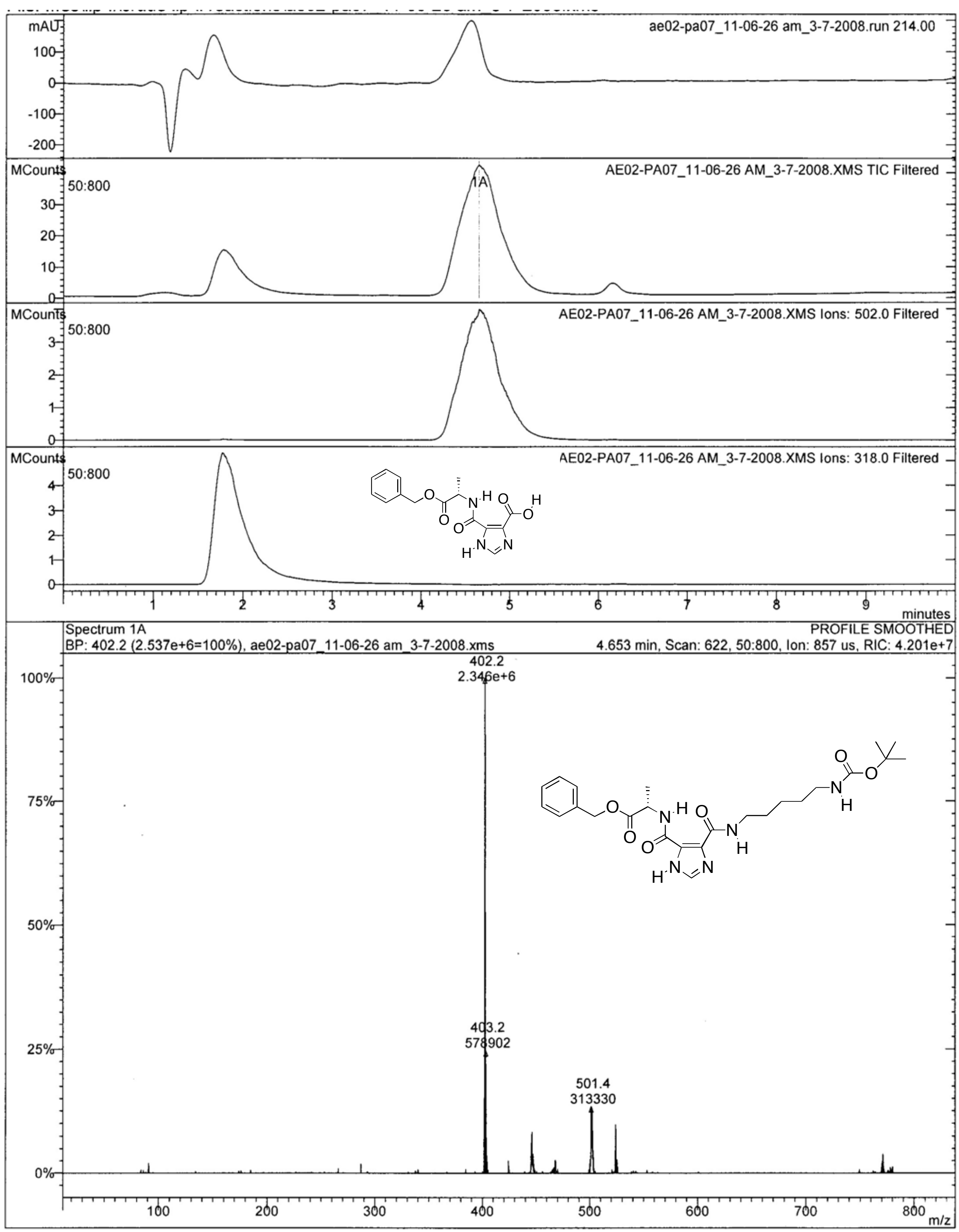


**Figure S133.** LC/MS data for the crude reaction to yield **5{**44}.

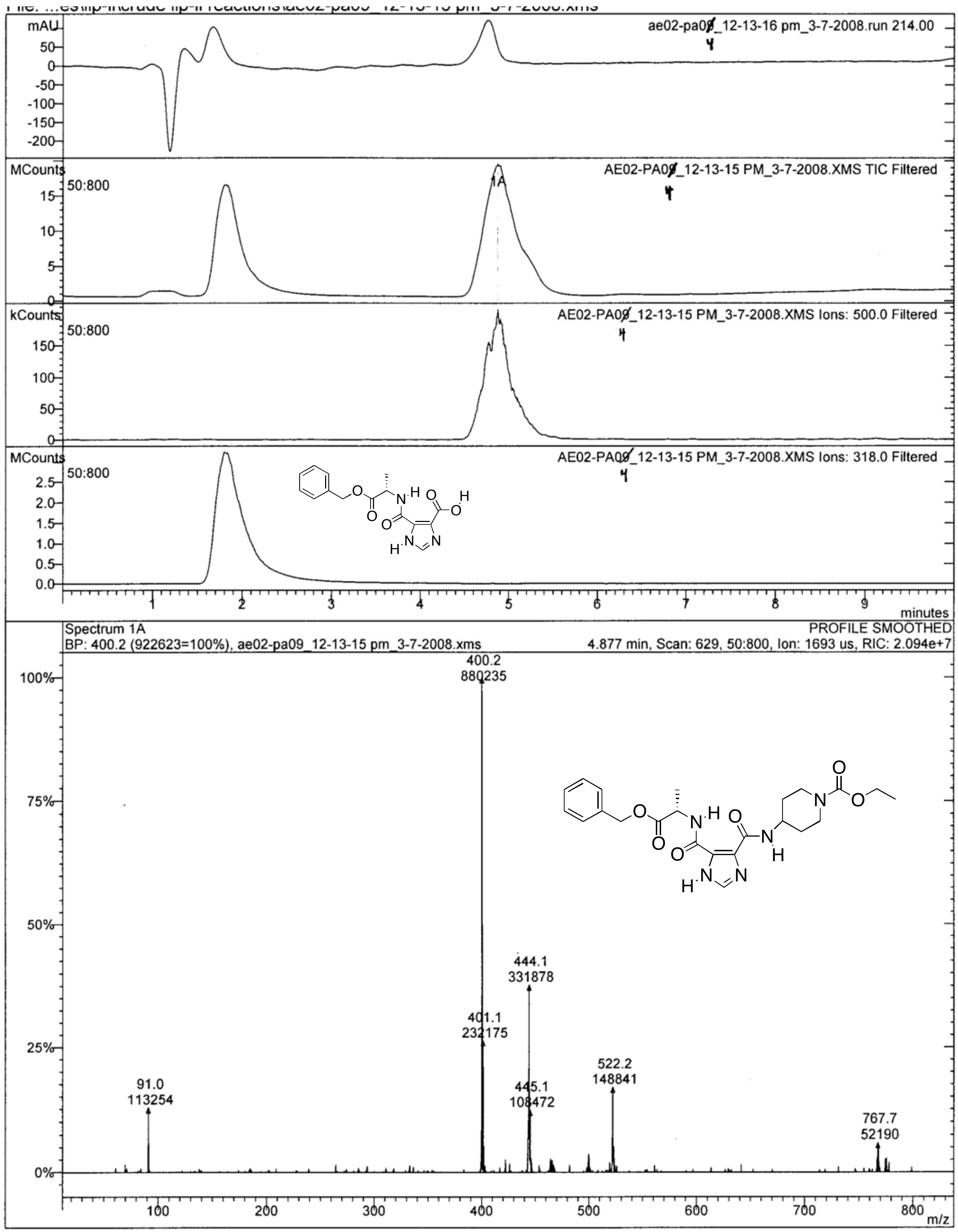


**Figure S134.** LC/MS data for the crude reaction to yield **5{**48}.

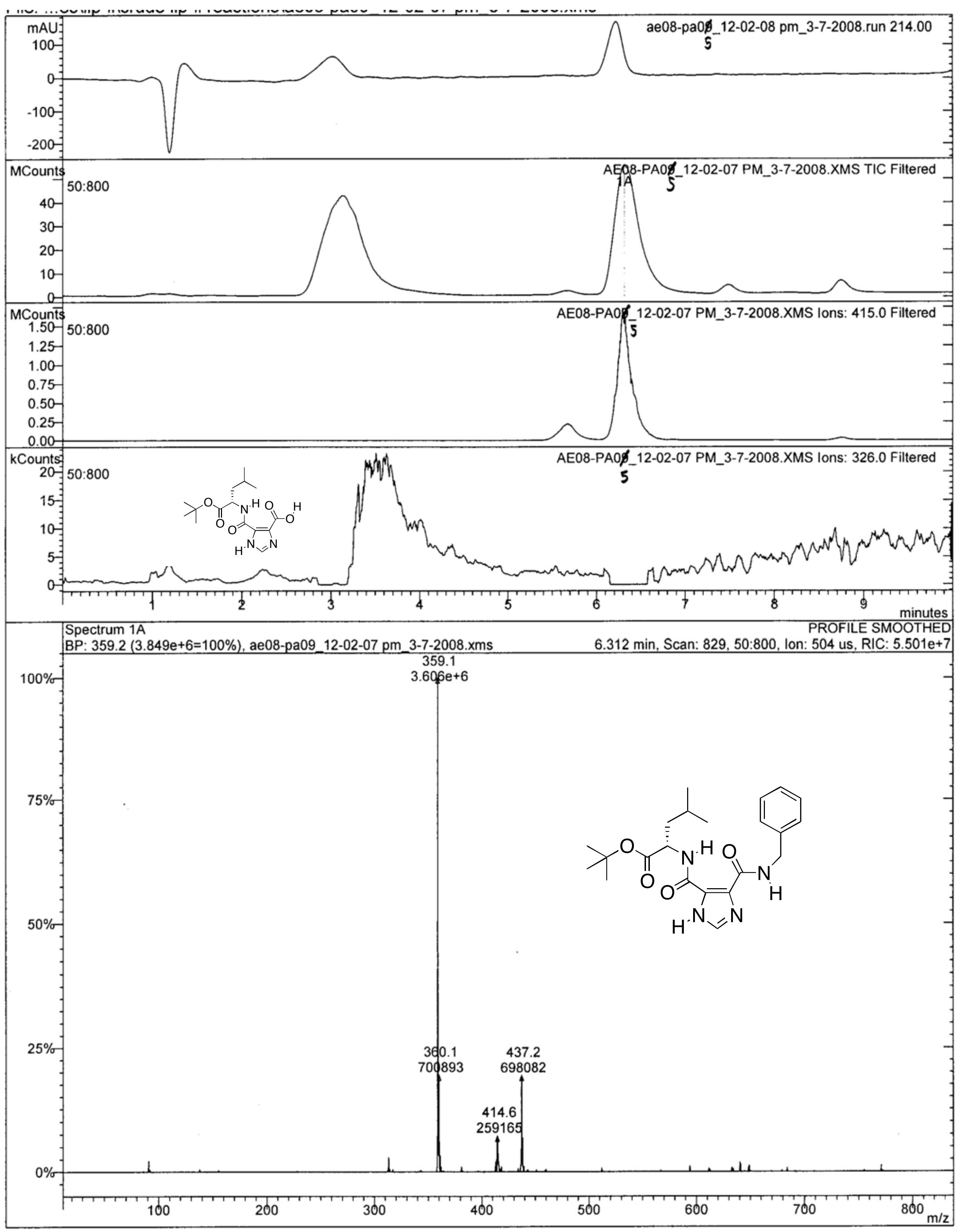


**Figure S135.** LC/MS data for the crude reaction to yield **5{**59}.

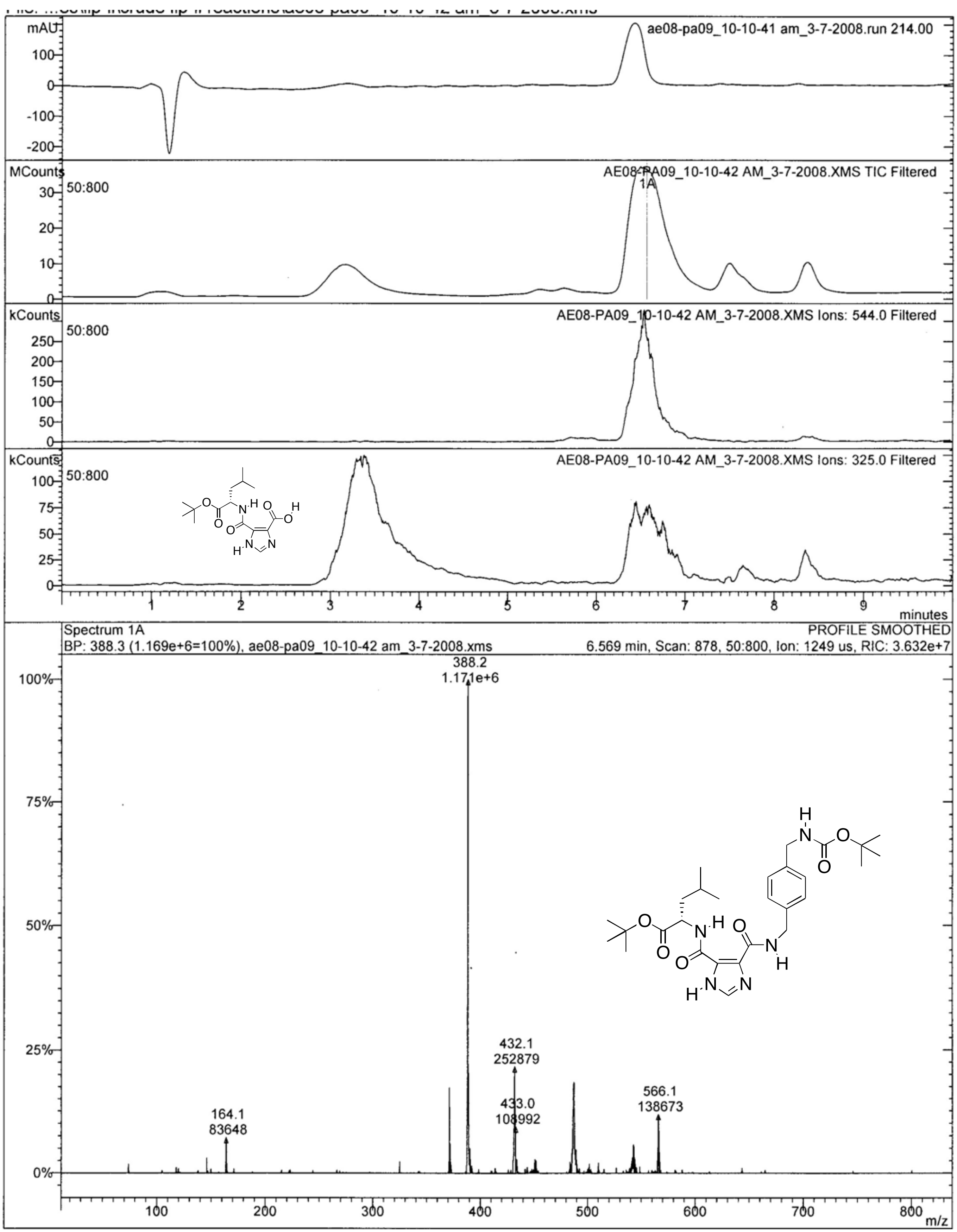


**Figure S136.** LC/MS data for the crude reaction to yield **5{**63}.

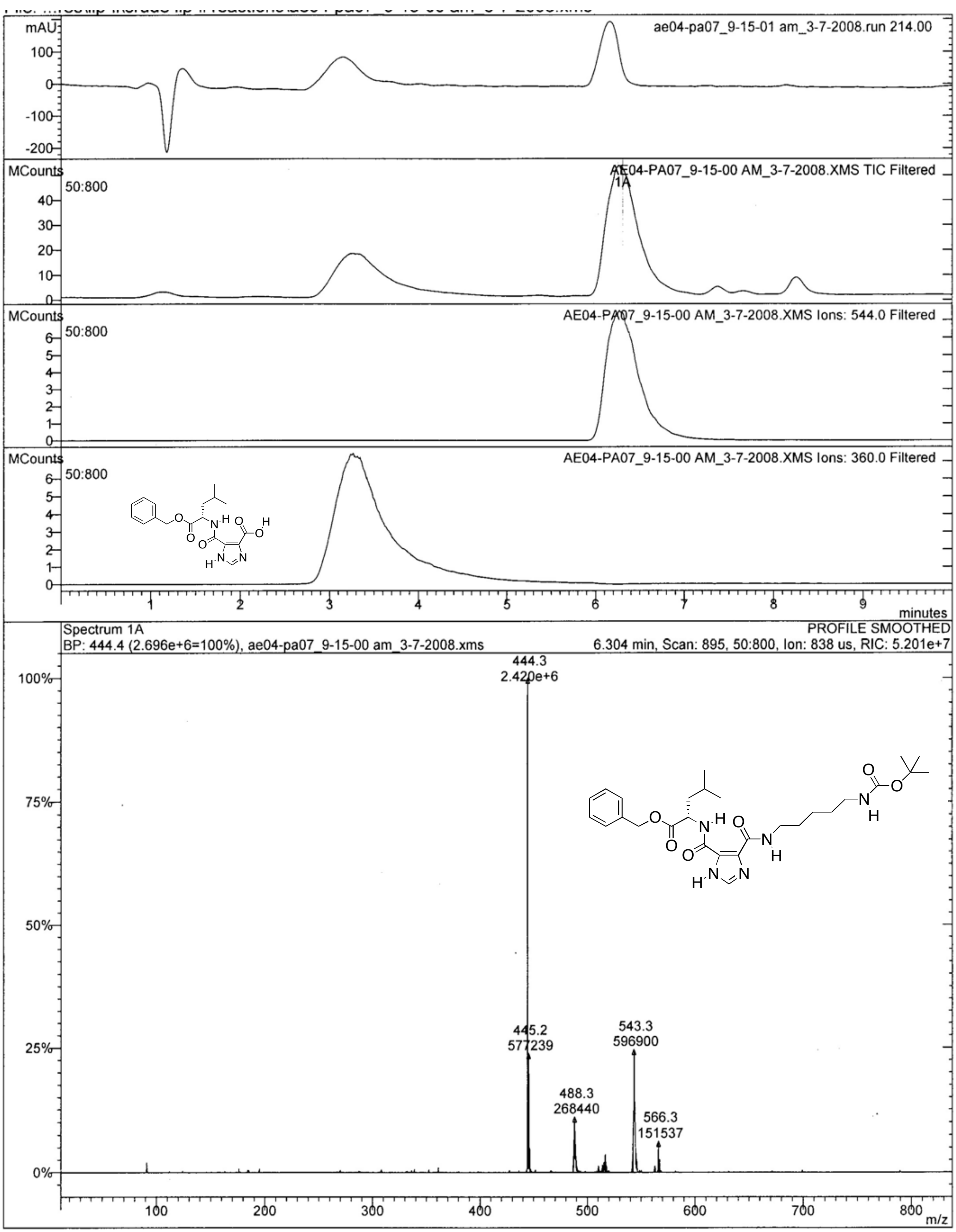


**Figure S137.** LC/MS data for the crude reaction to yield **5{**72}.

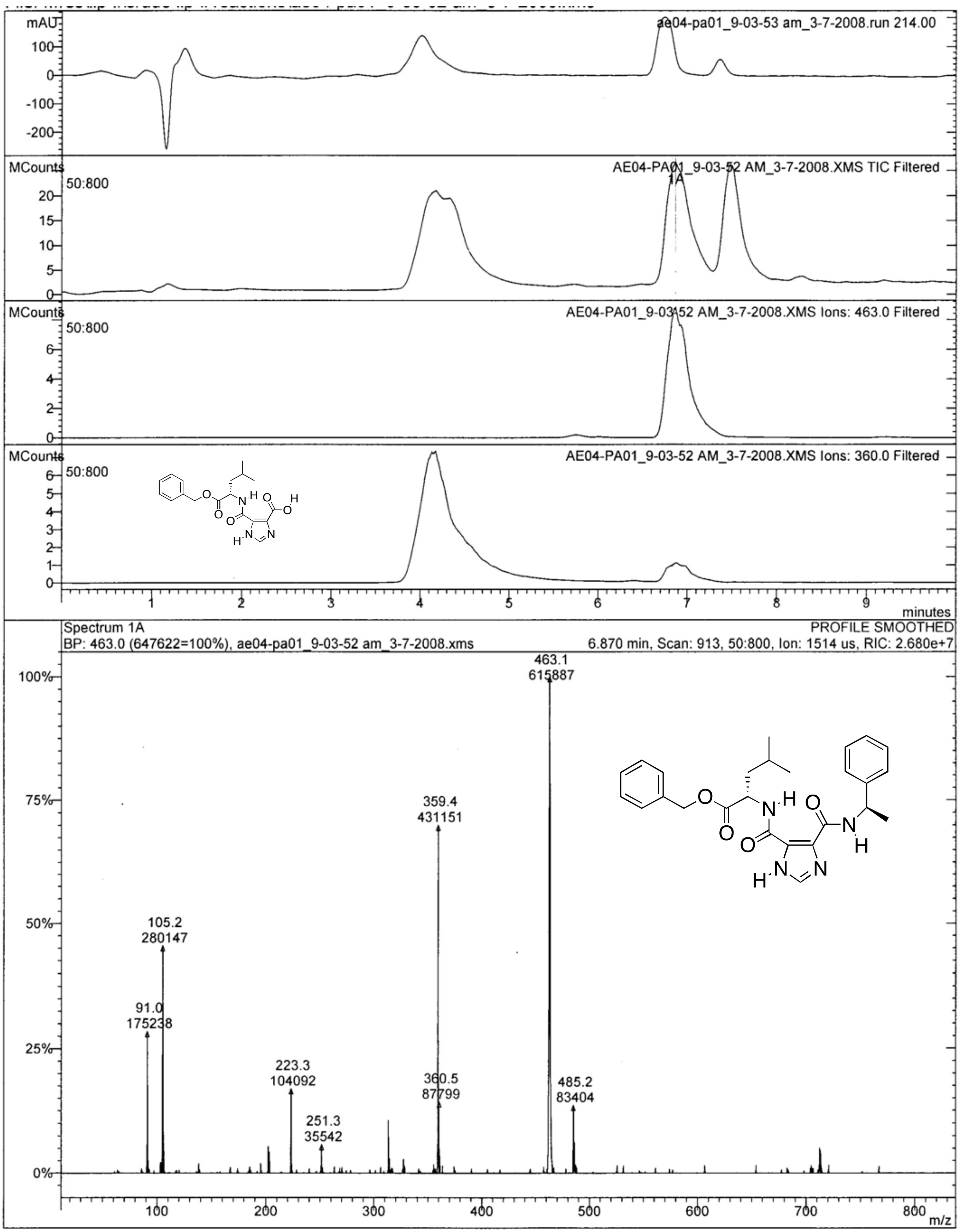


**Figure S138.** LC/MS data for the crude reaction to yield **5{**74}.

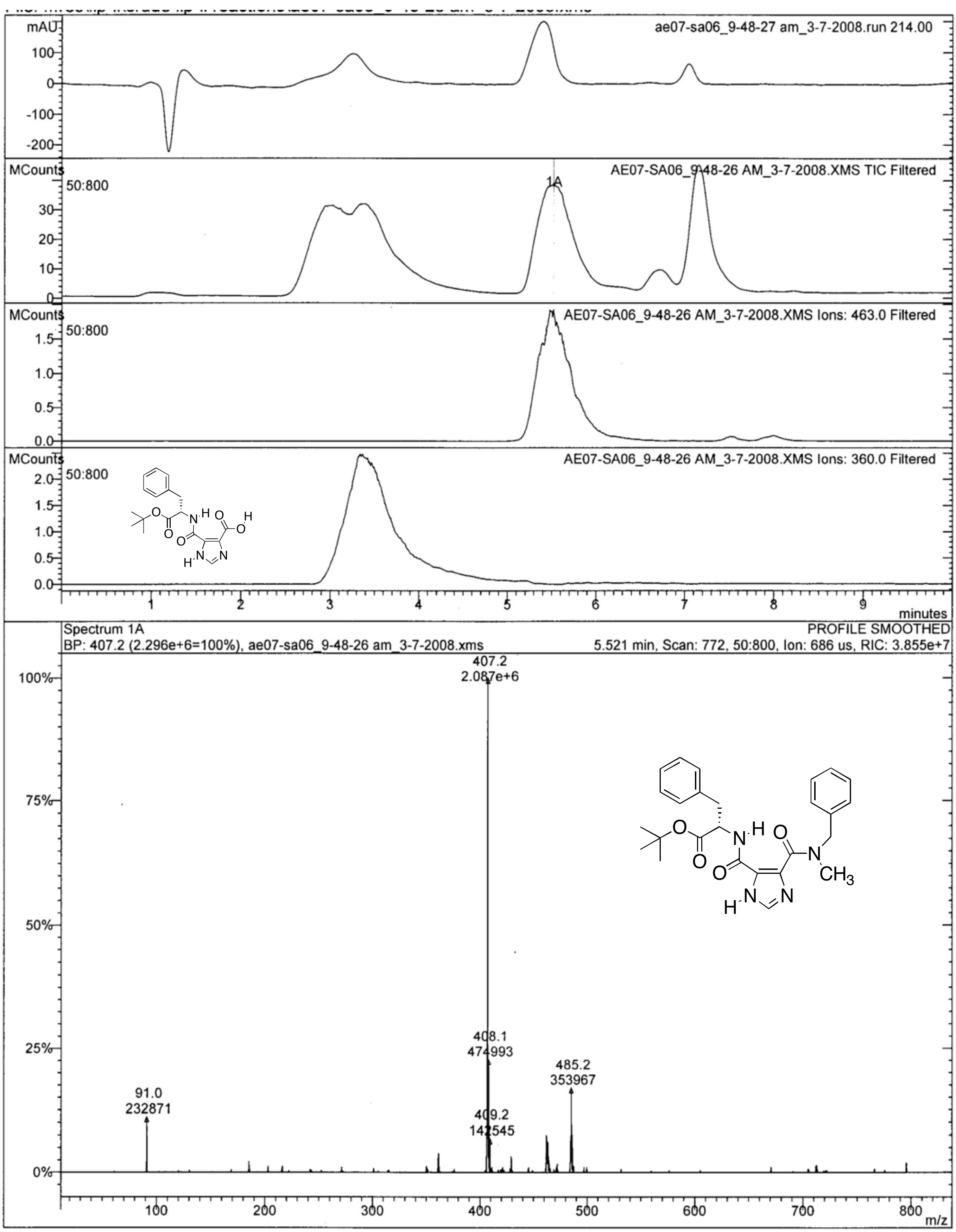


**Figure S139.** LC/MS data for the crude reaction to yield **5{**93}.

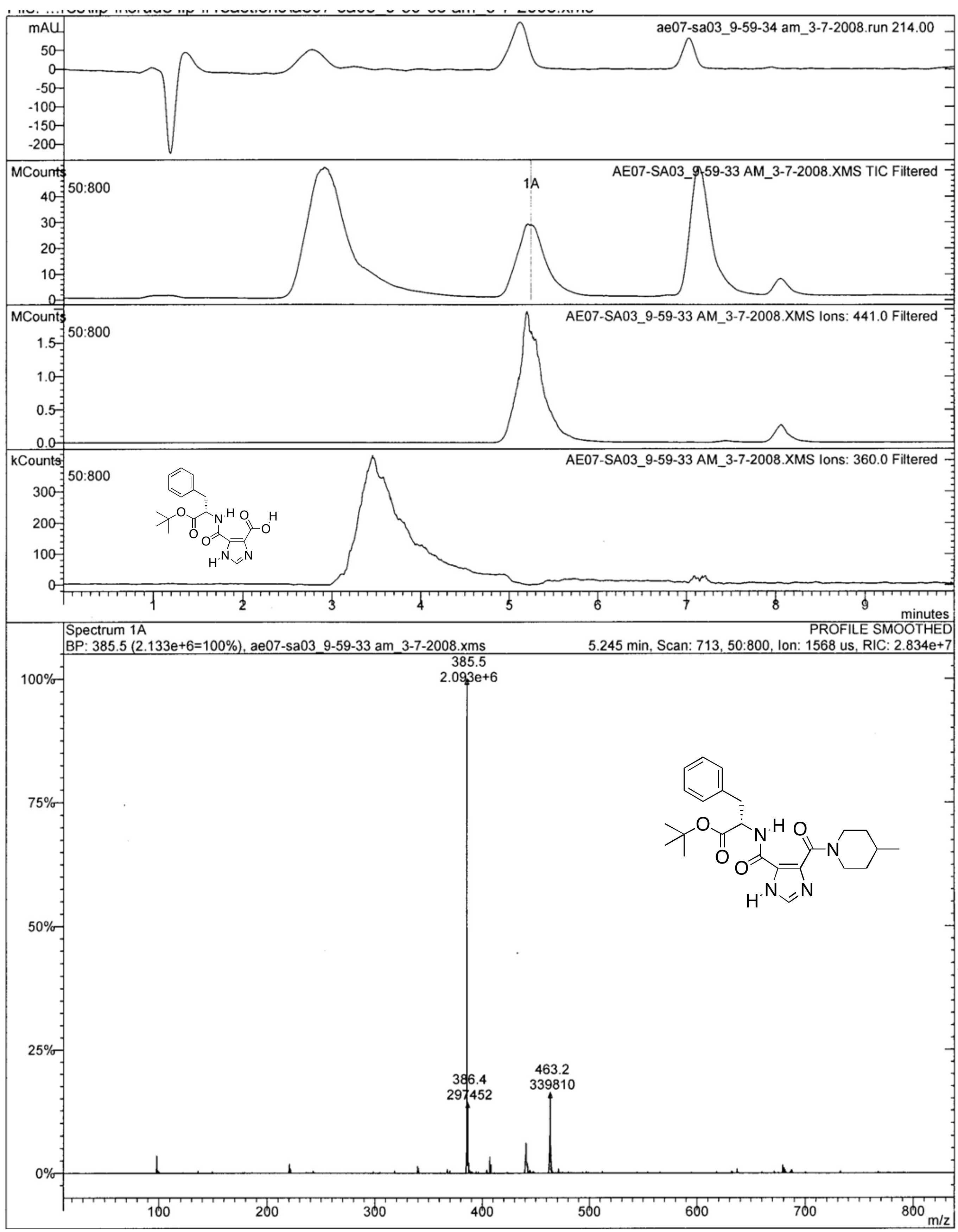


**Figure S140.** LC/MS data for the crude reaction to yield **5{**97}.

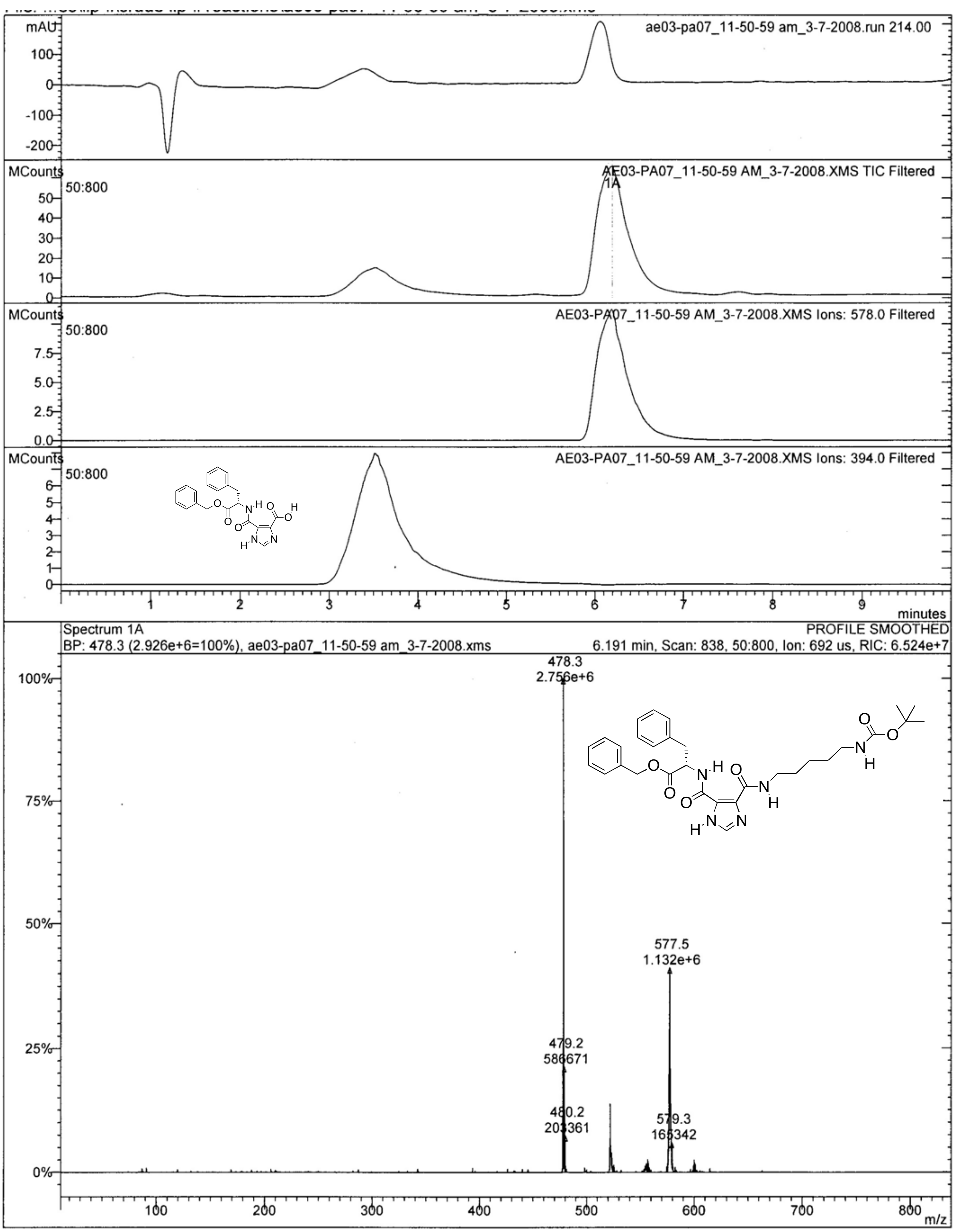


**Figure S141.** LC/MS data for the crude reaction to yield **5{**100}.

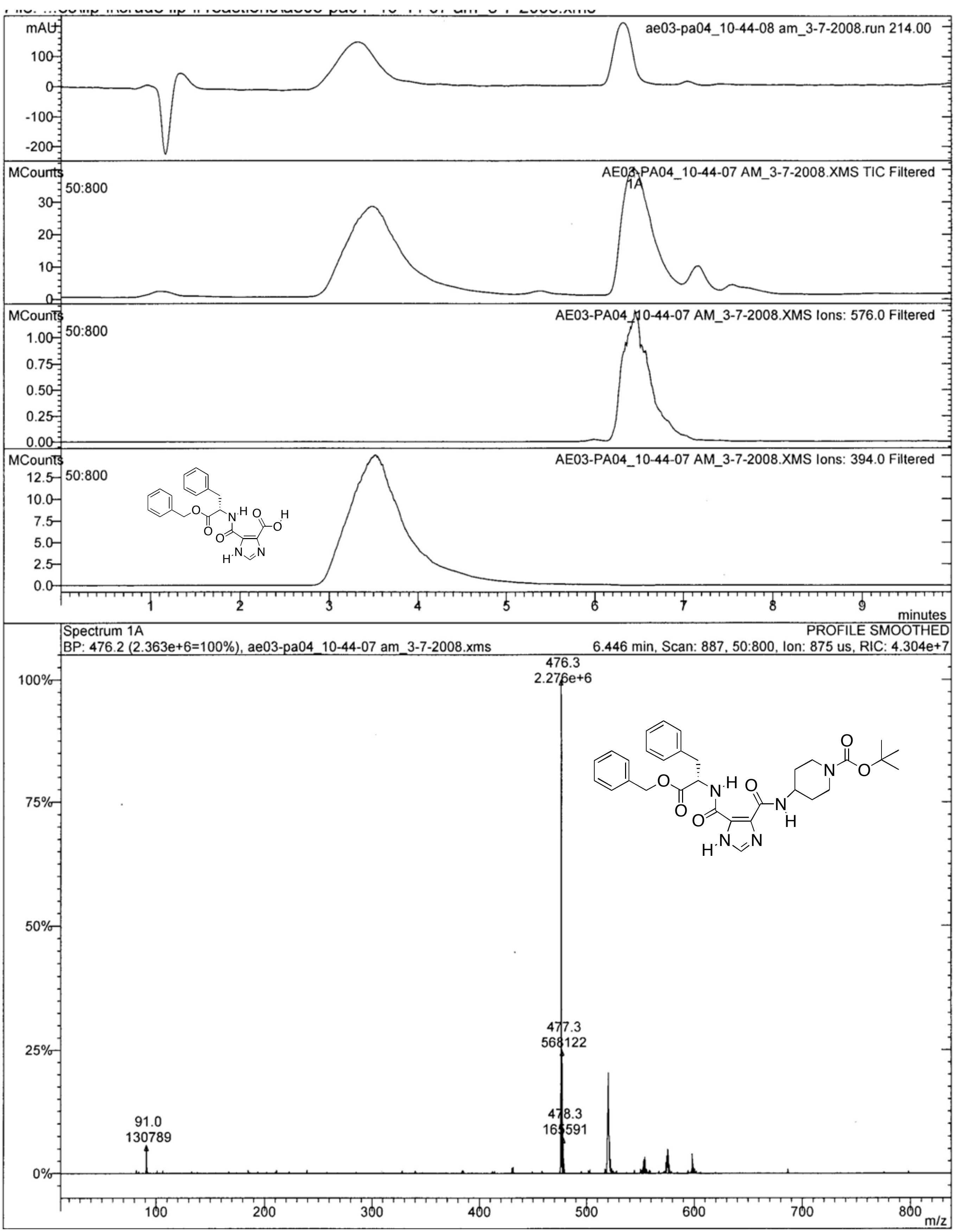


**Figure S142.** LC/MS data for the crude reaction to yield **6**104}.

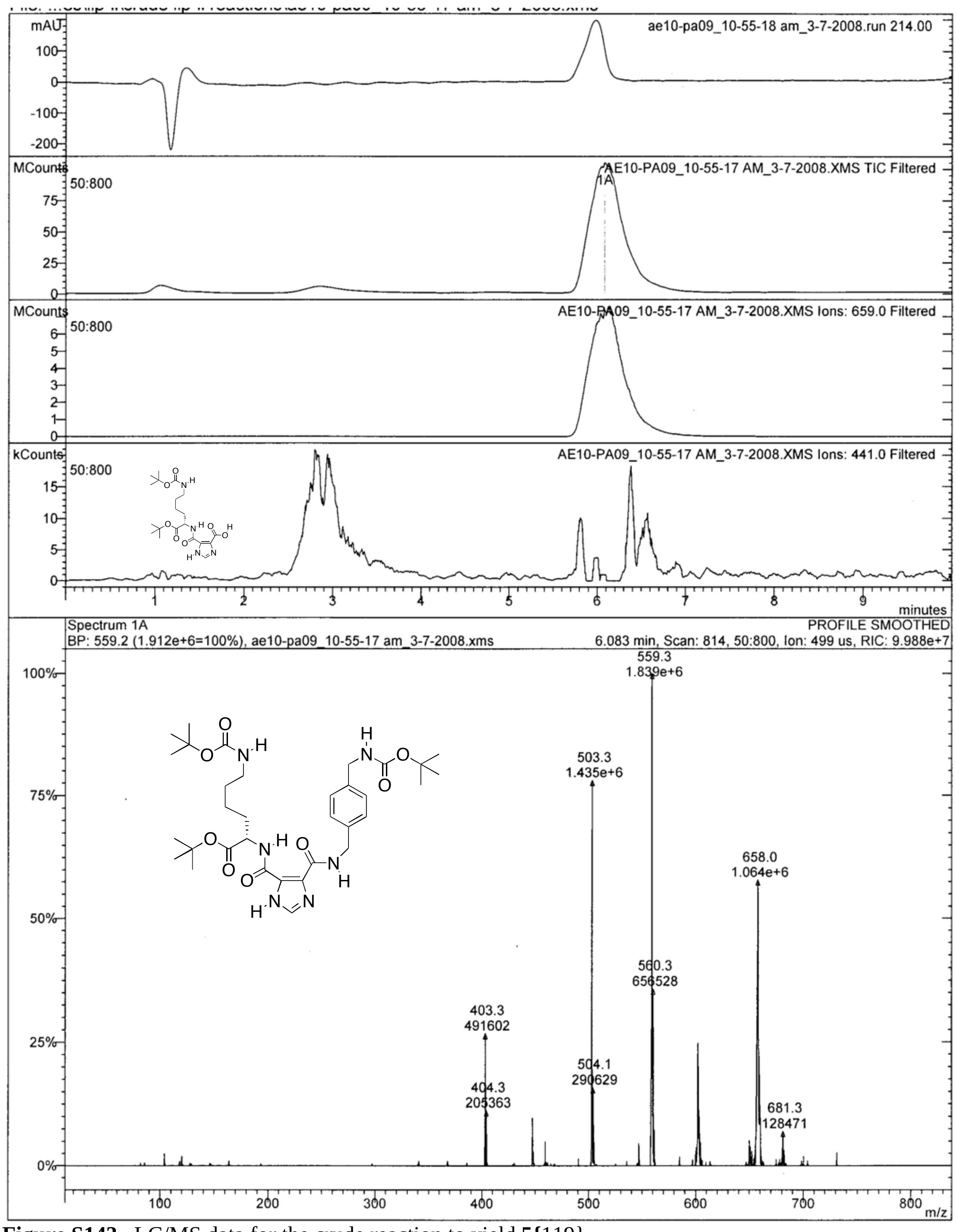


**Figure S143.** LC/MS data for the crude reaction to yield **5{**119}.

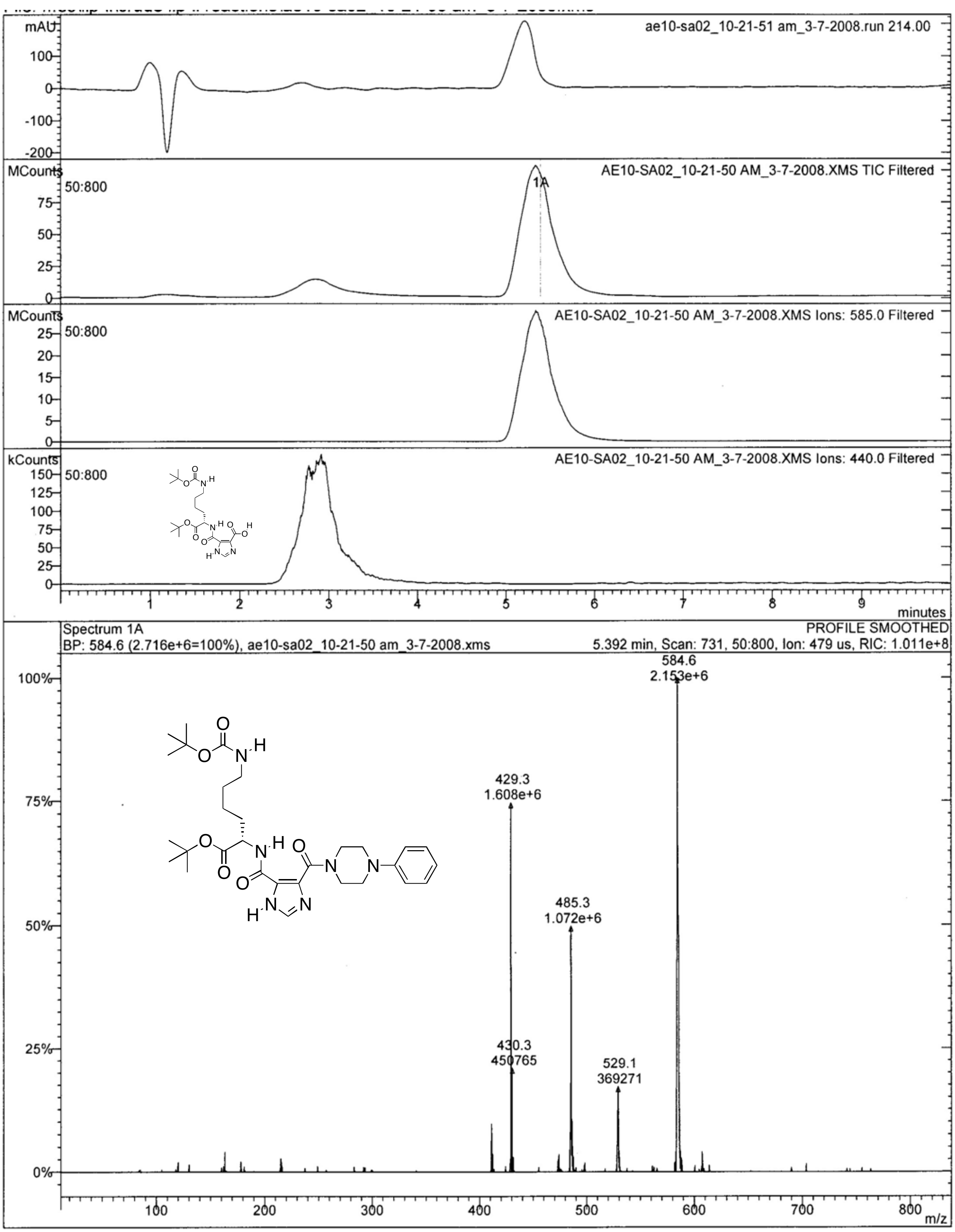


**Figure S144.** LC/MS data for the crude reaction to yield **5{**126}.

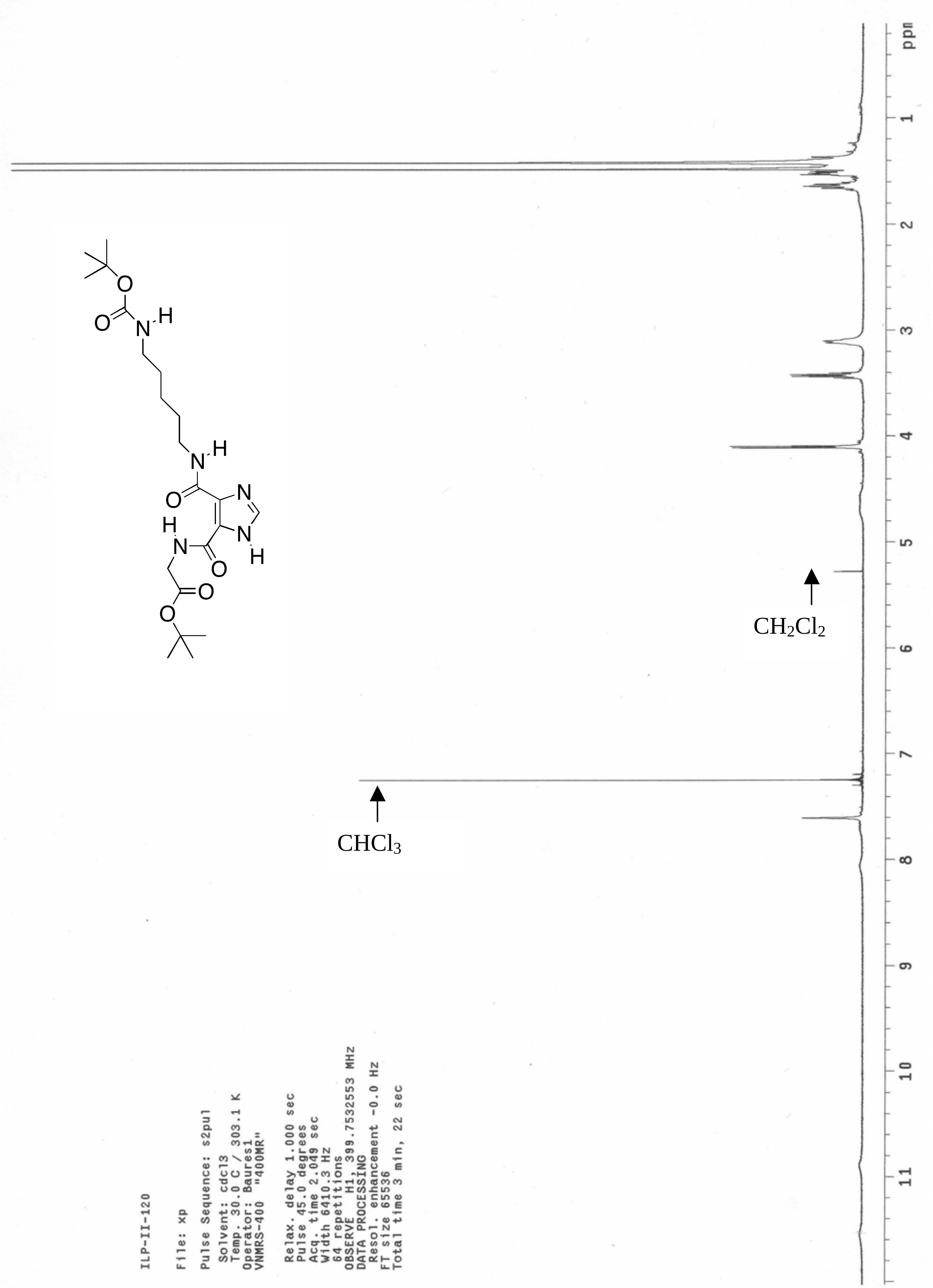


**Figure S145.** $^{1}$H-NMR for **5{**2}.

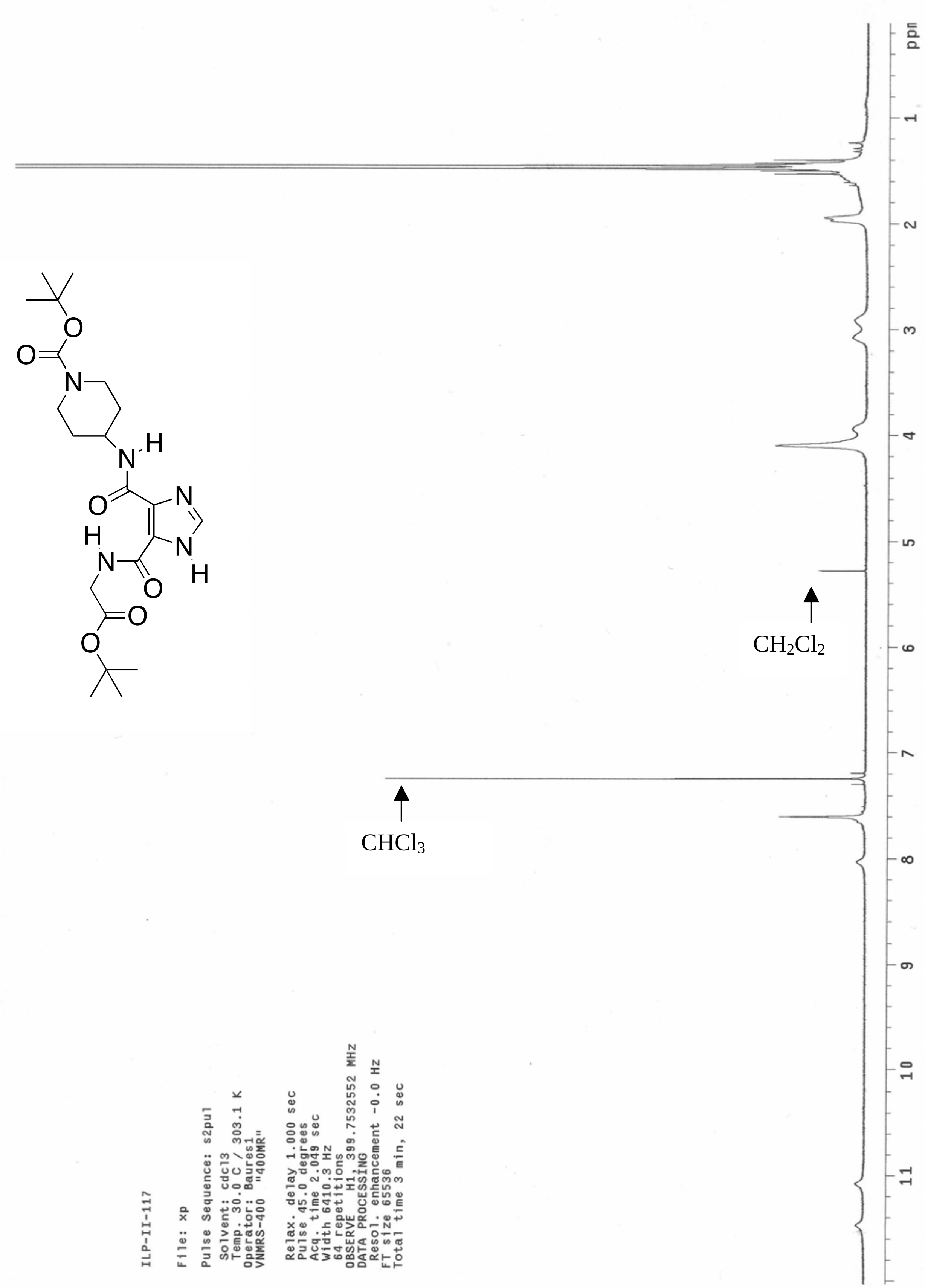


**Figure S146.** $^{1}$H-NMR for **5{**6}.

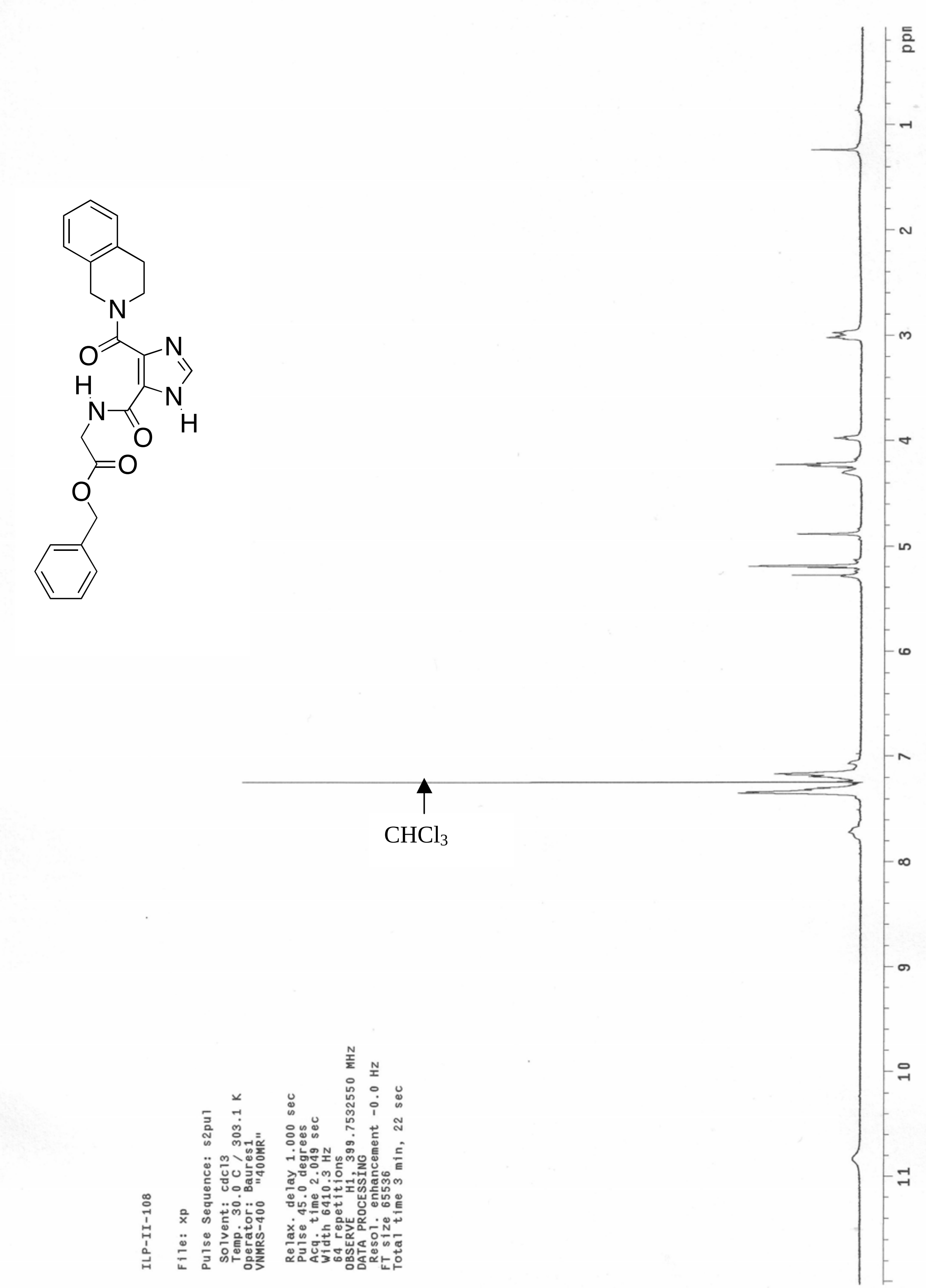


**Figure S147.** $^1$H-NMR for **5{**26}.

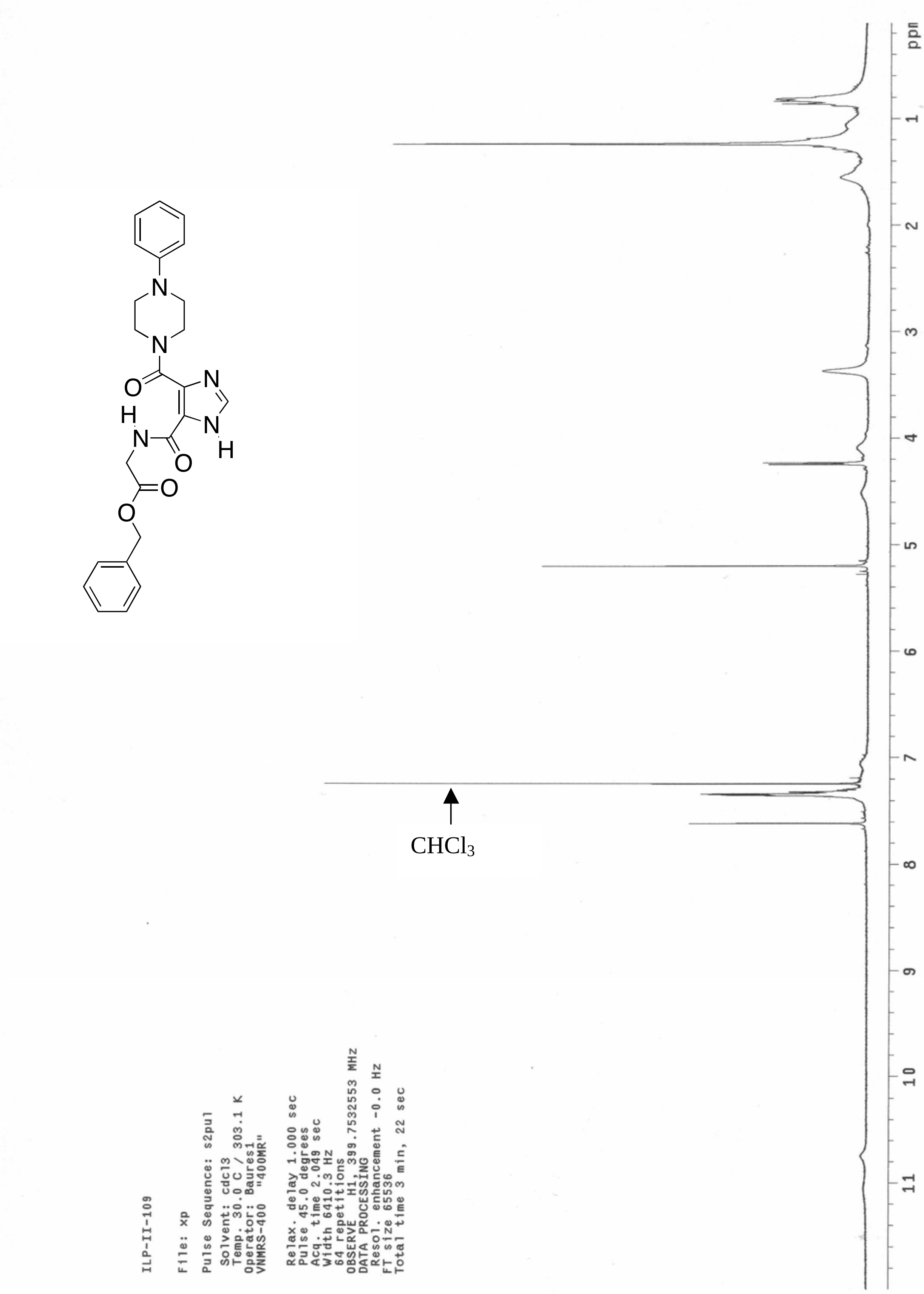


**Figure S148.** $^{1}$H-NMR for **5{**28}.

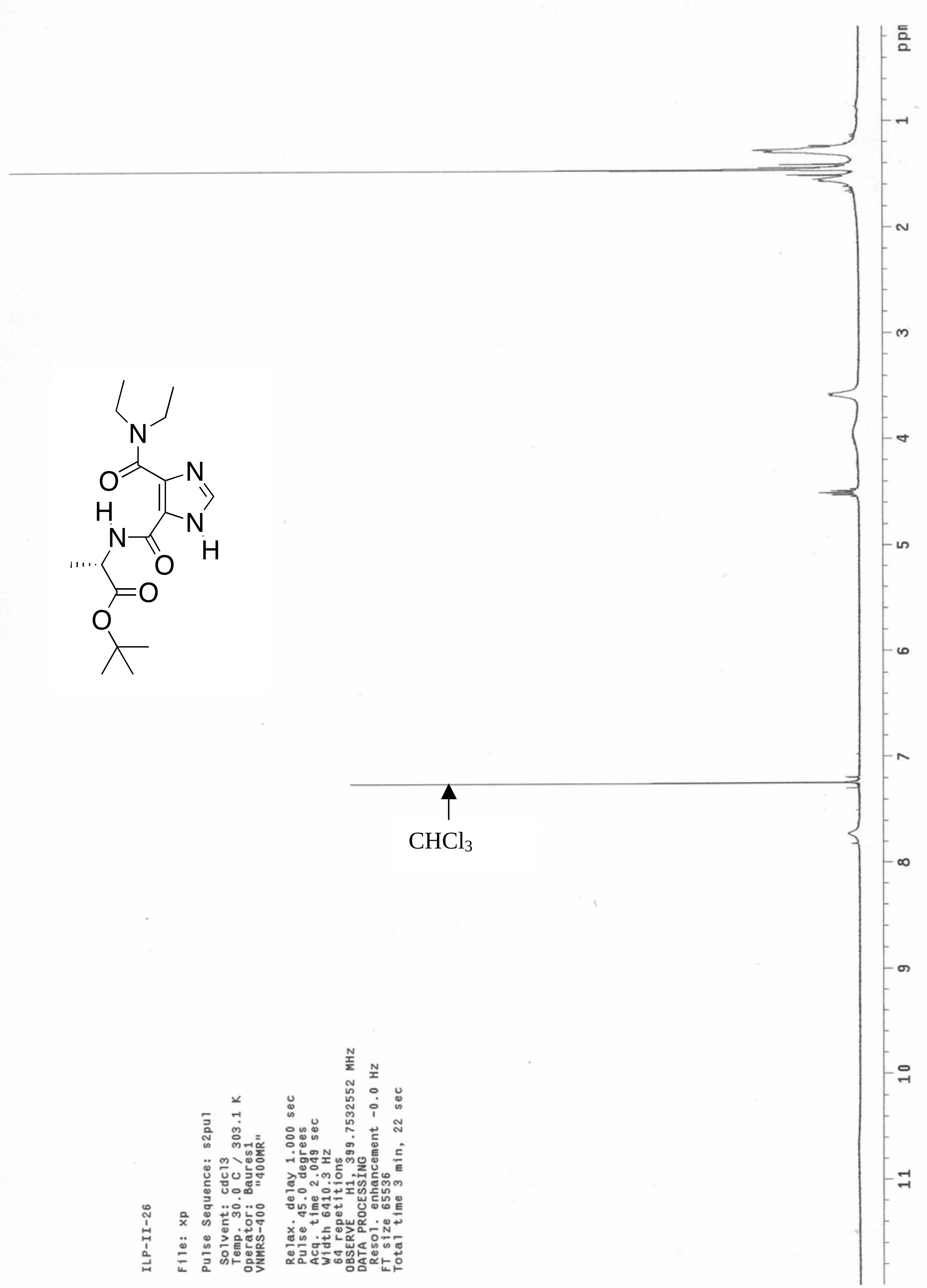


**Figure S149.** $^1$H-NMR for **5{**38}.

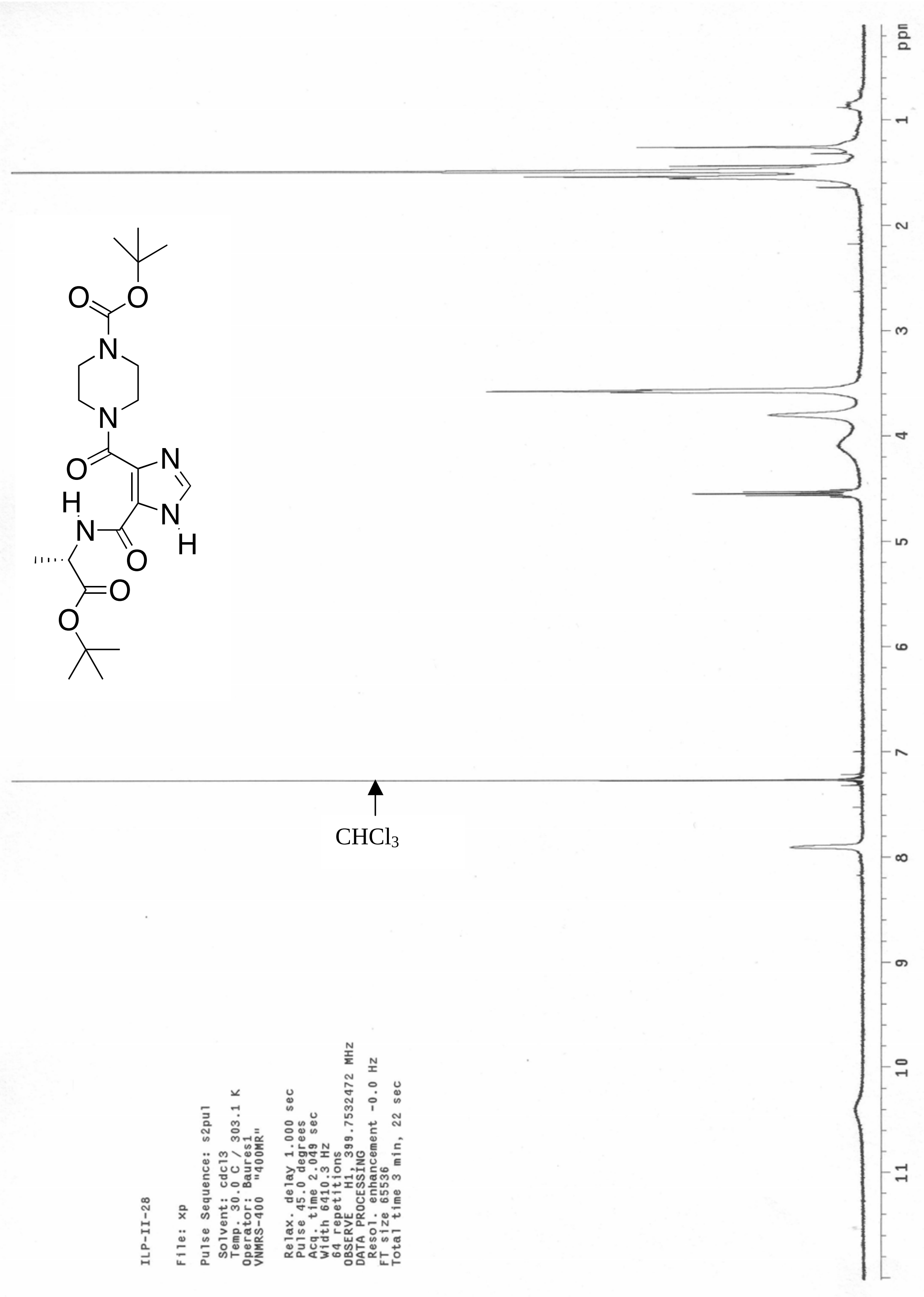


**Figure S150.** $^{1}$H-NMR for **5{**39}.

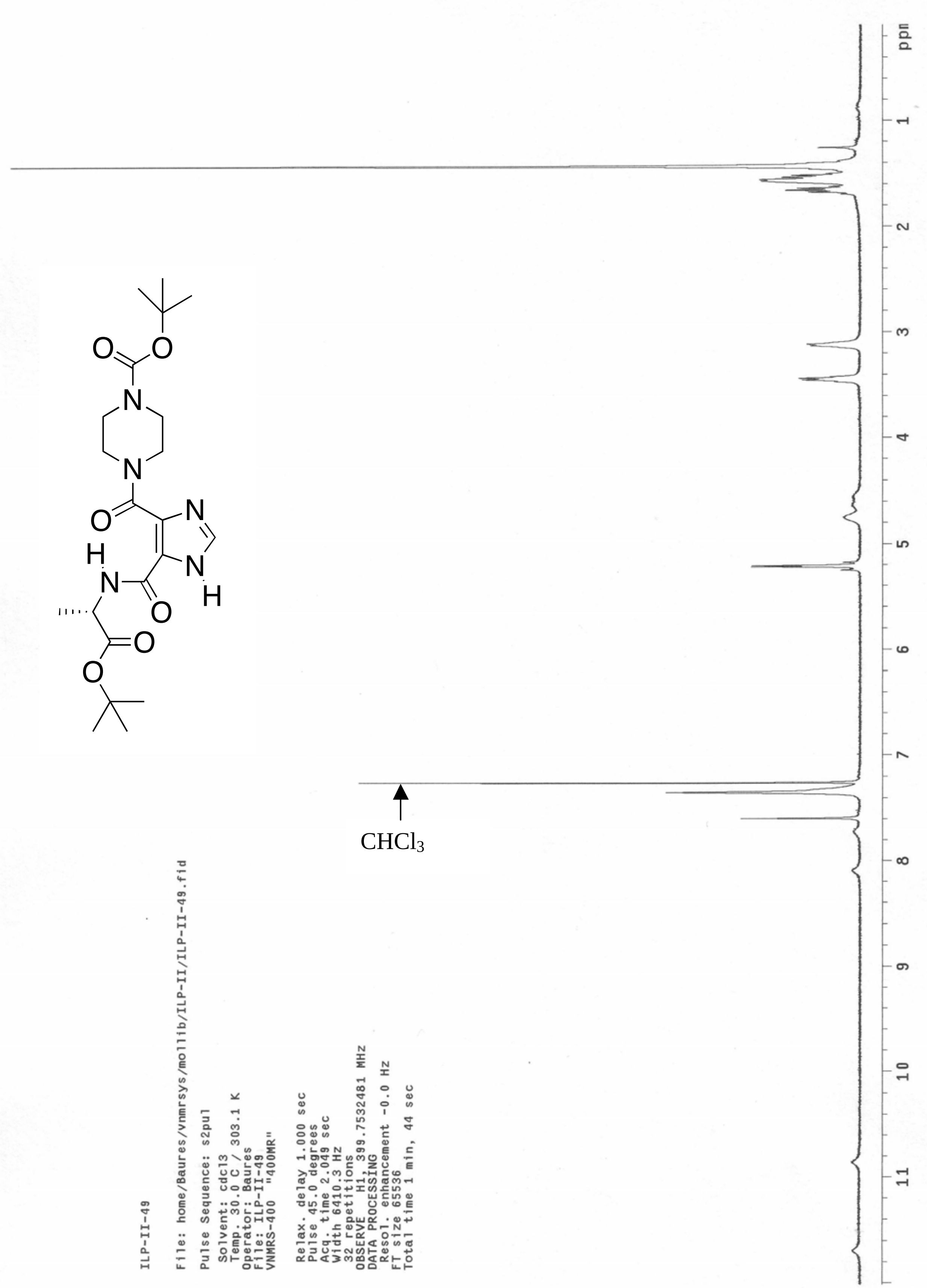


**Figure S151.** $^{1}$H-NMR for **5{**44}.

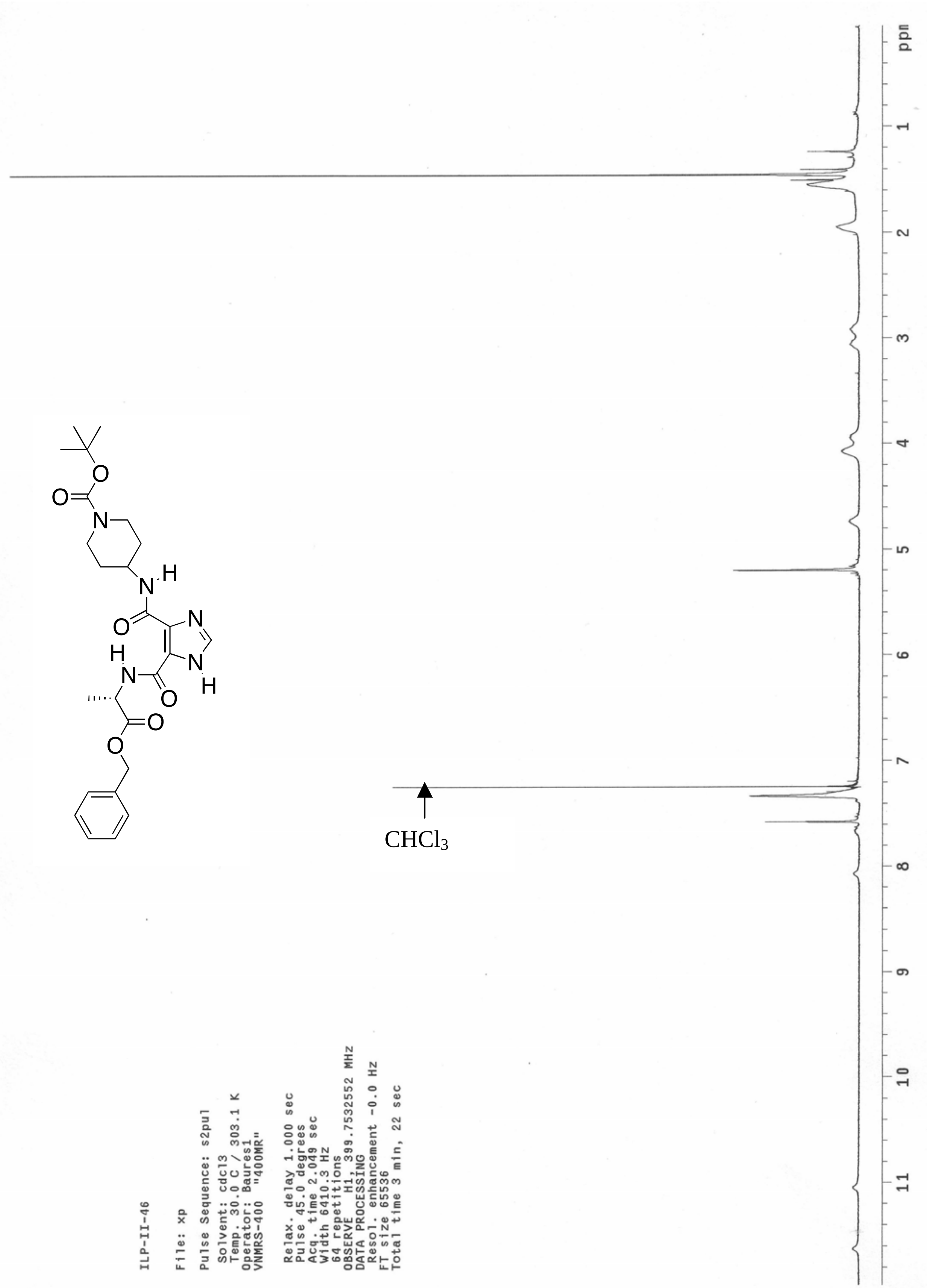


**Figure S152.** $^{1}$H-NMR for **5{**48}.

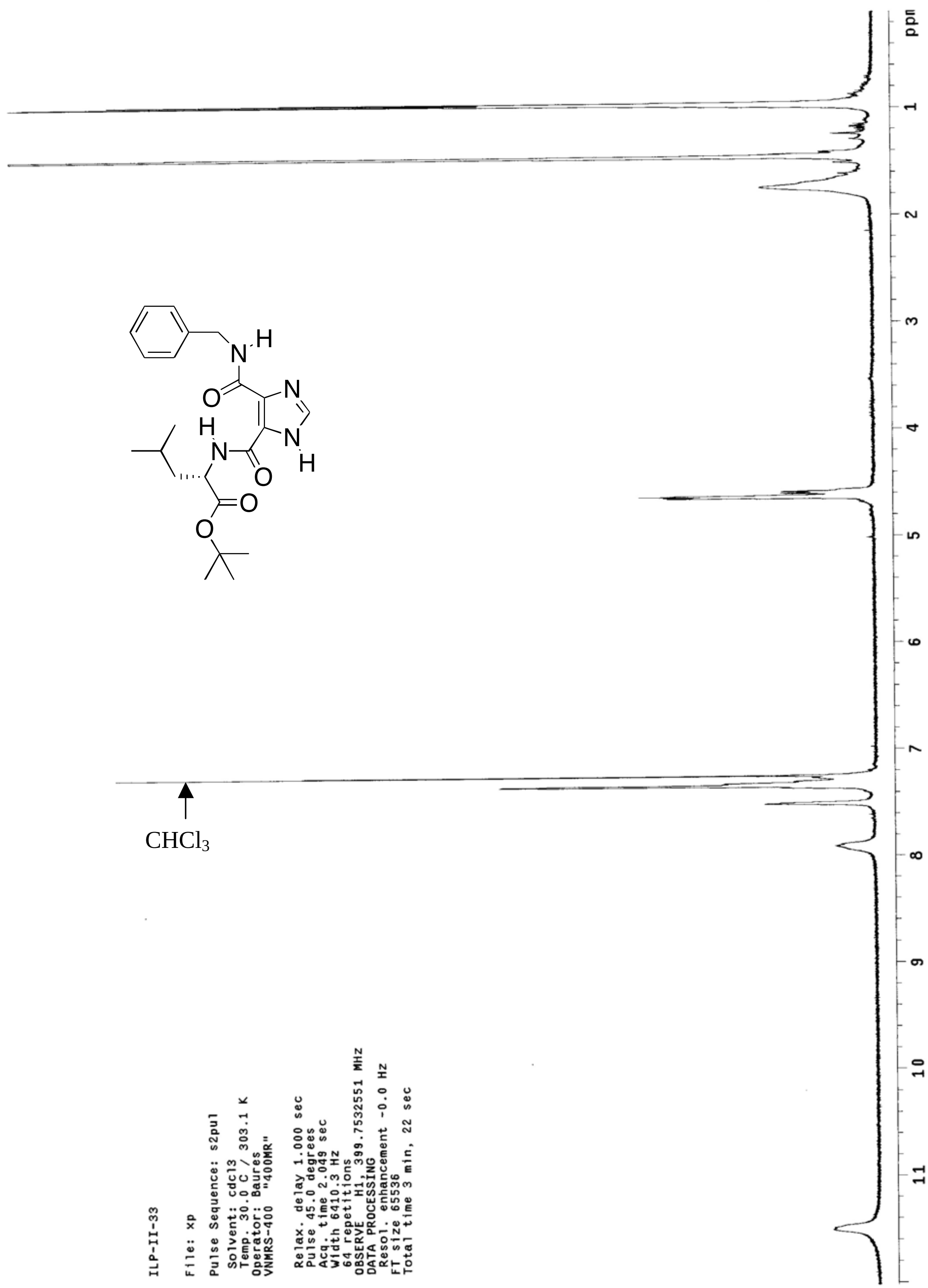


**Figure S153.** $^{1}$H-NMR for **5{**59}.

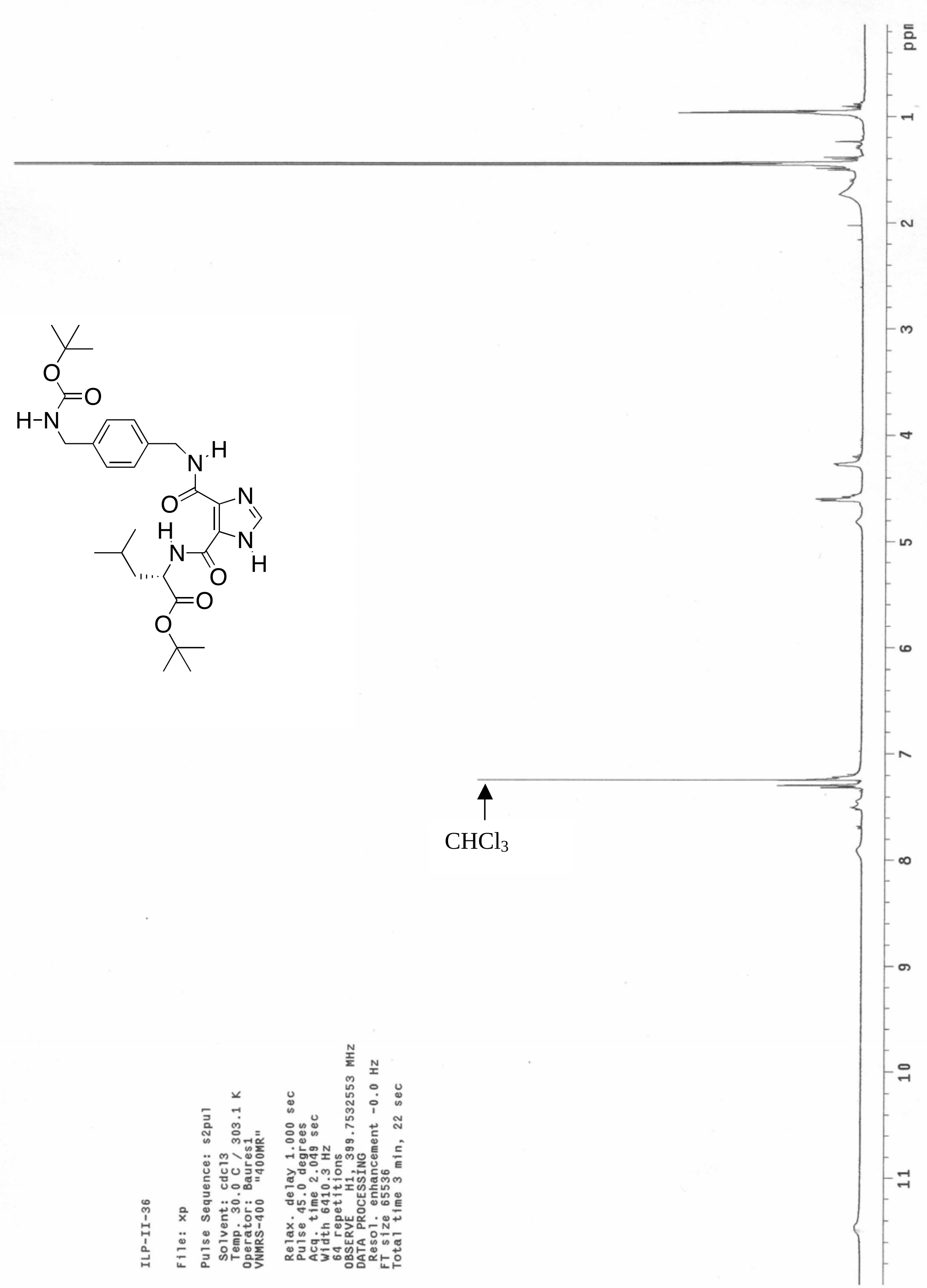


**Figure S154.** $^{1}$H-NMR for **5{**63}.

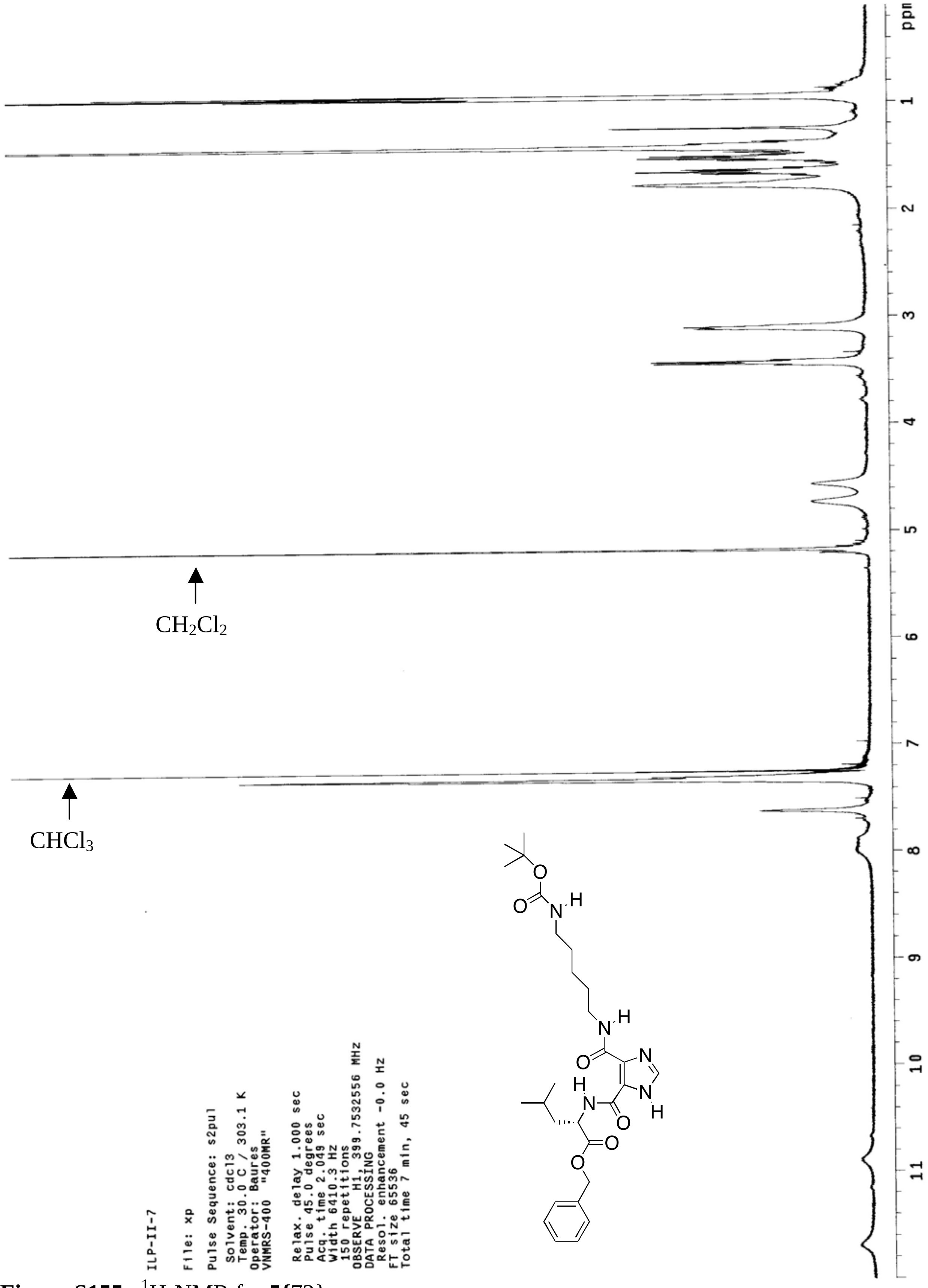


**Figure S155.** $^1$H-NMR for **5{**72}.

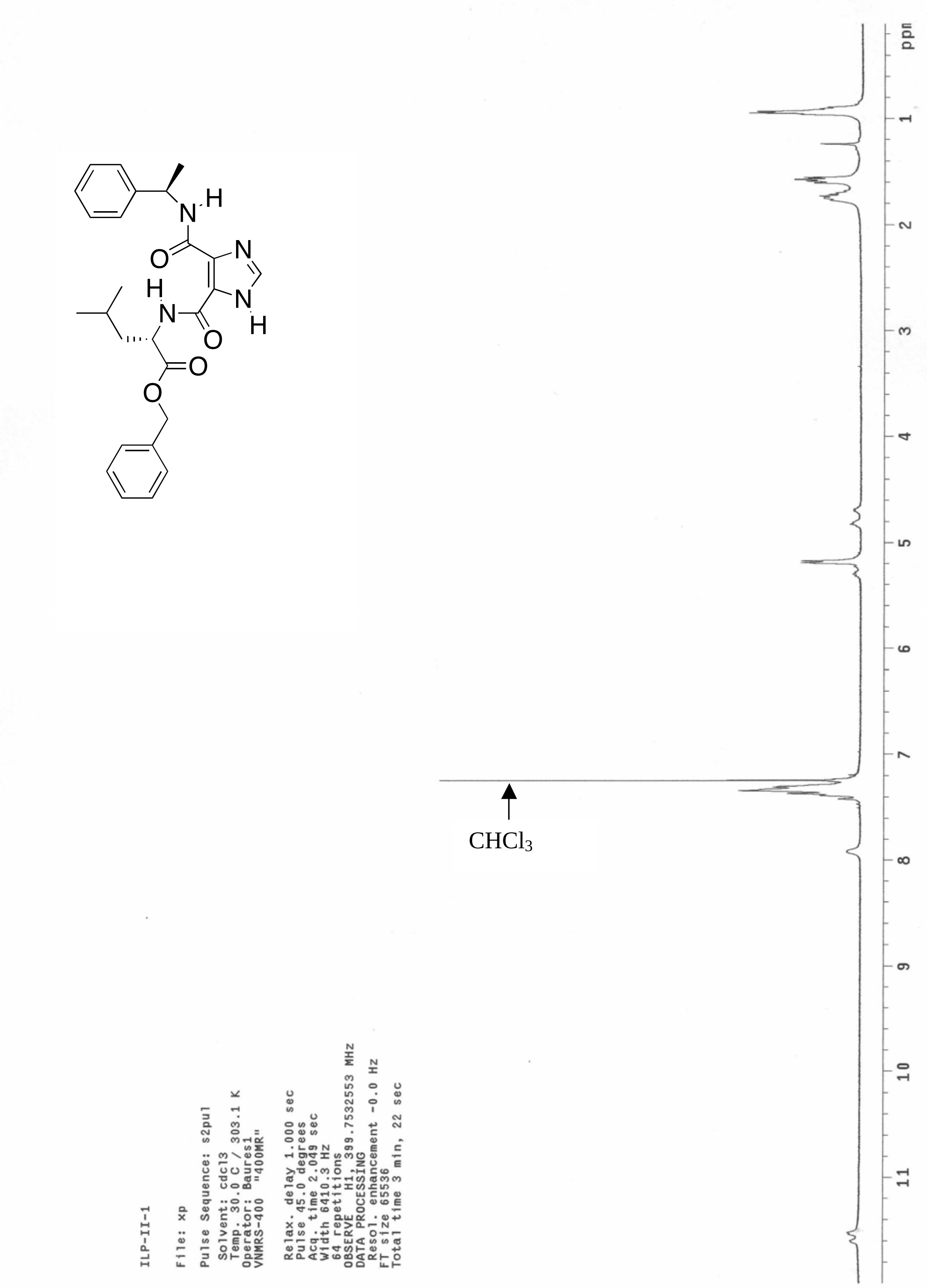


**Figure S156.** $^{1}$H-NMR for **5{**74}.

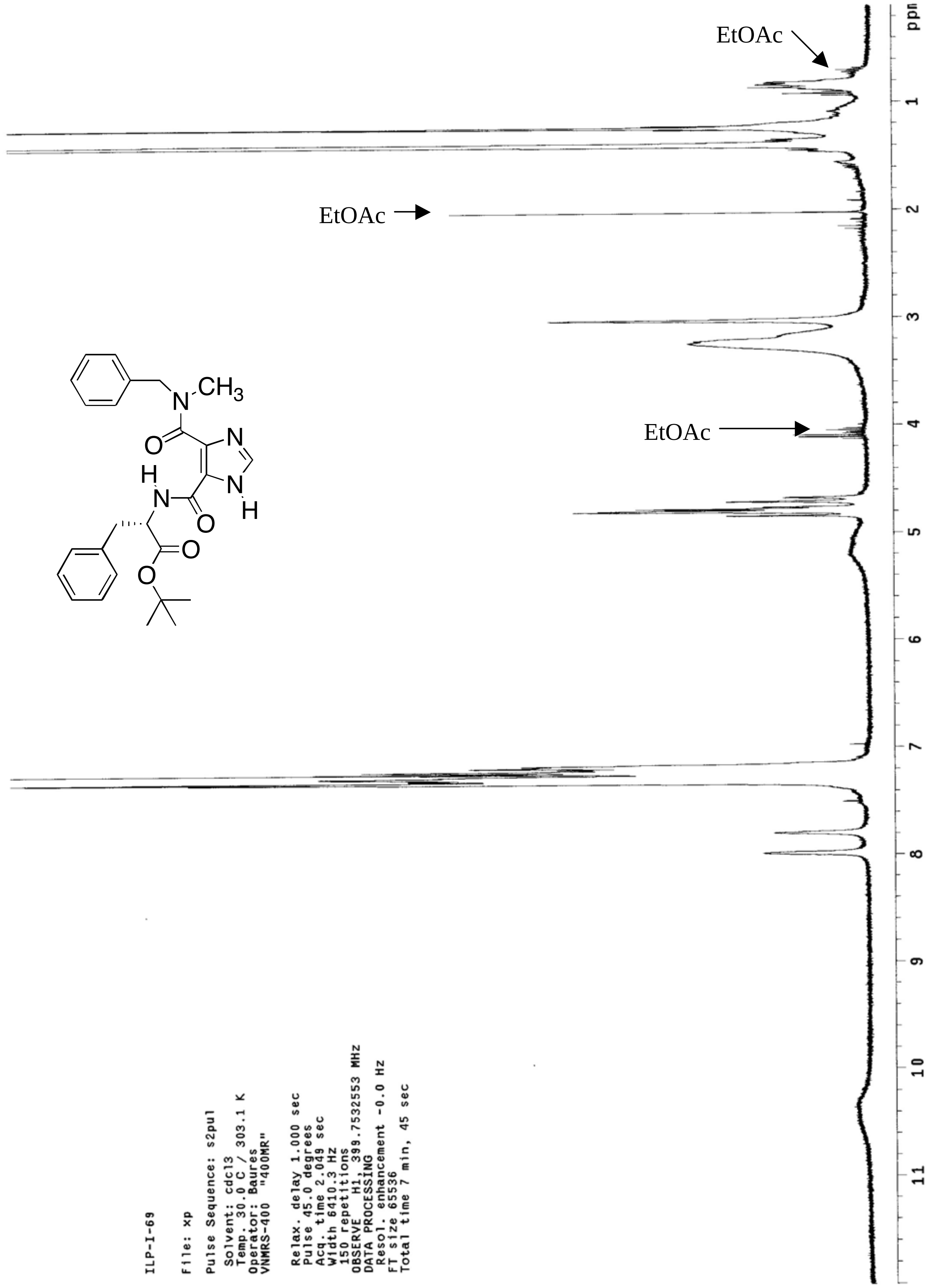


**Figure S157.** $^{1}$H-NMR for **5{**93}.

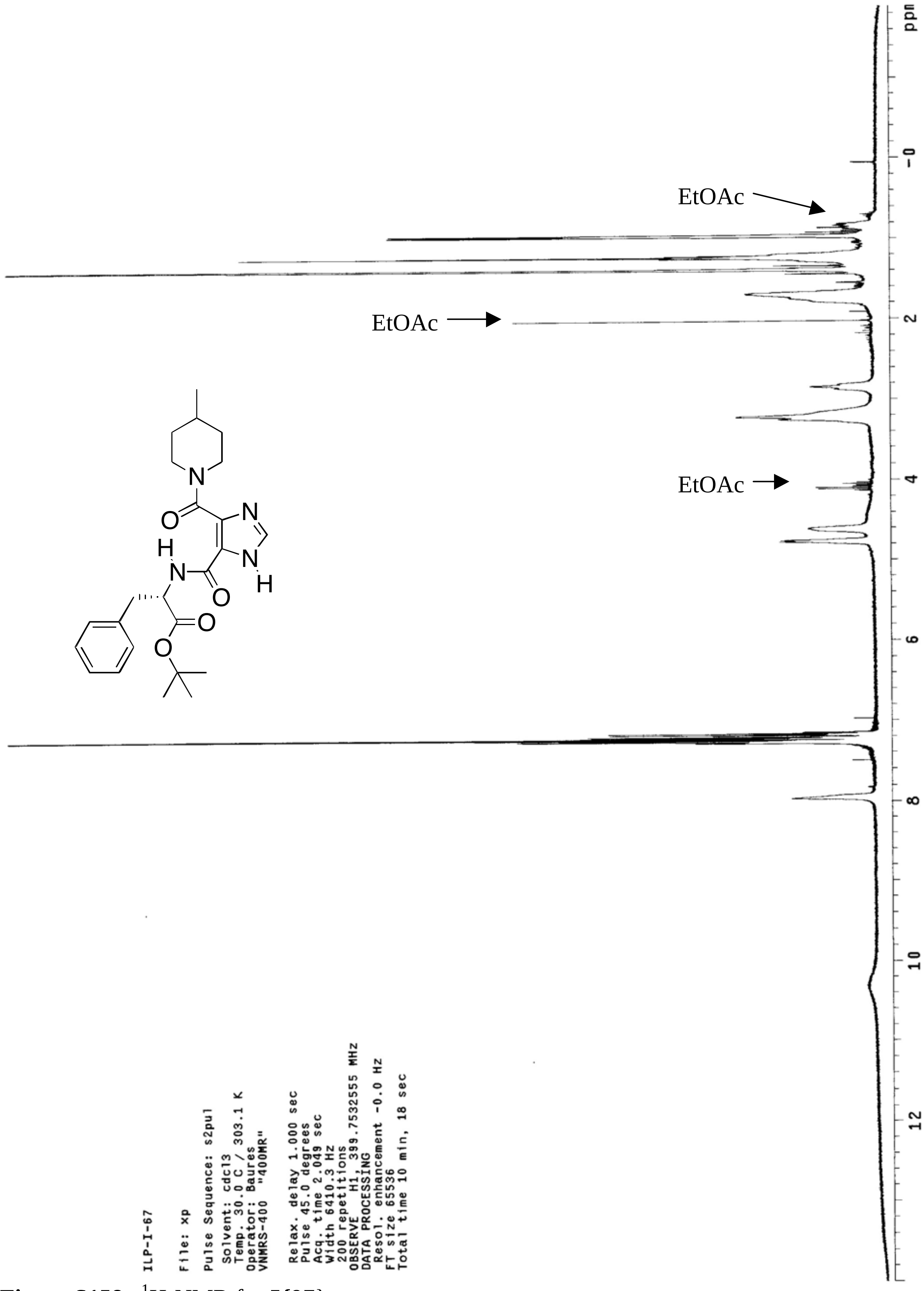


**Figure S158.** $^{1}$H-NMR for **5{**97}.

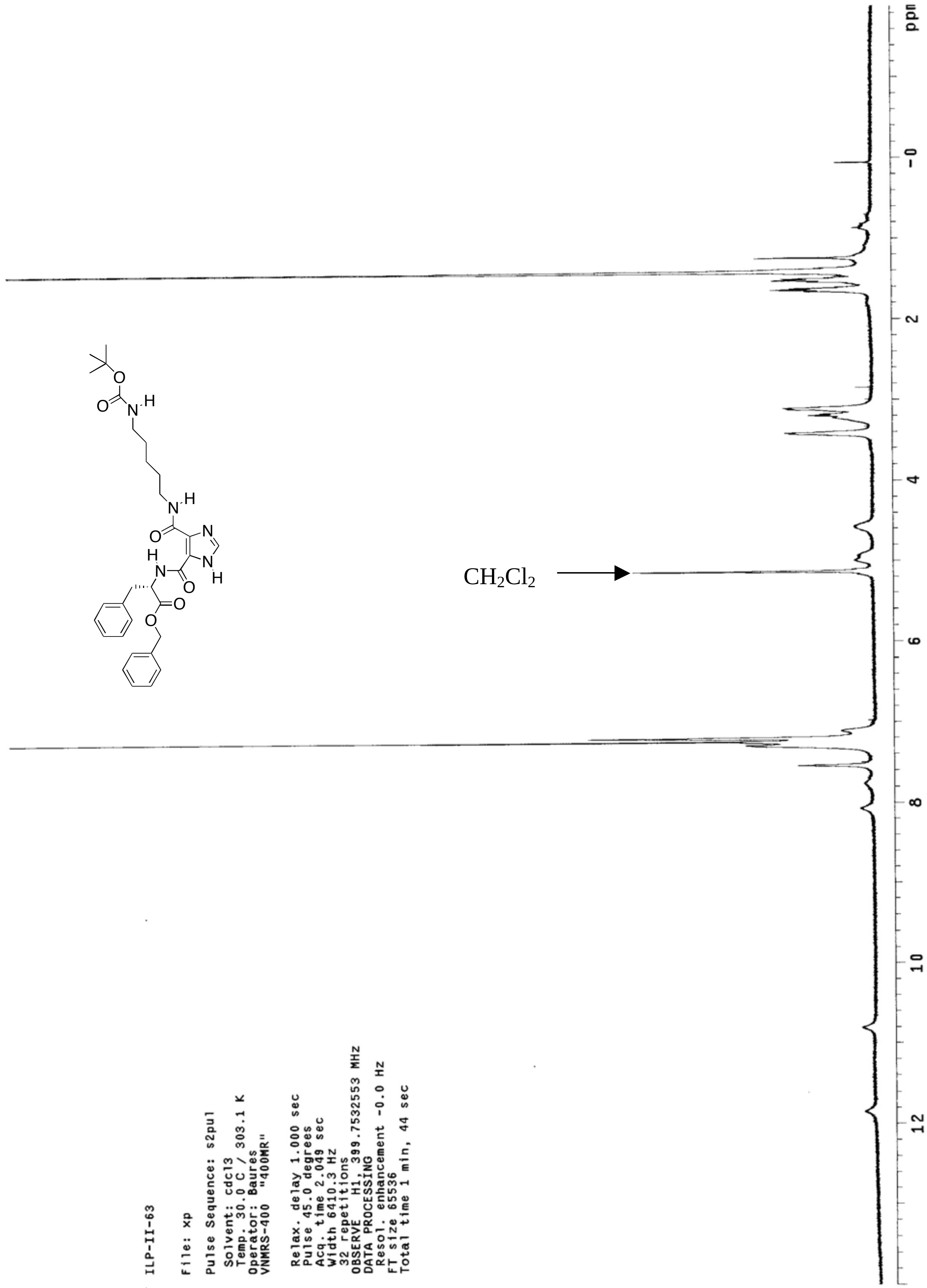


**Figure S159.** $^{1}$H-NMR for **5{**100}.

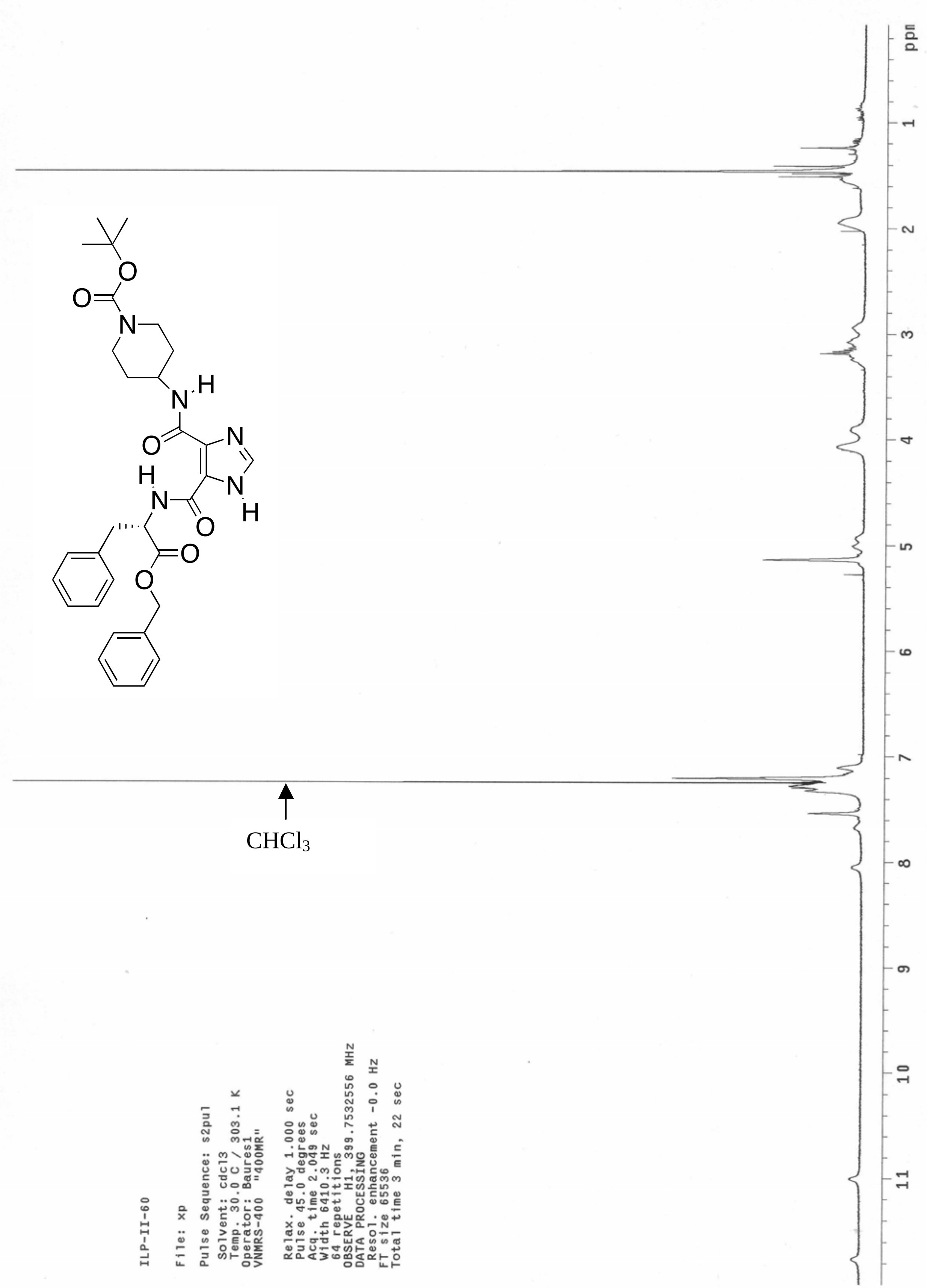


**Figure S160.** $^1$H-NMR for **5{**104}.

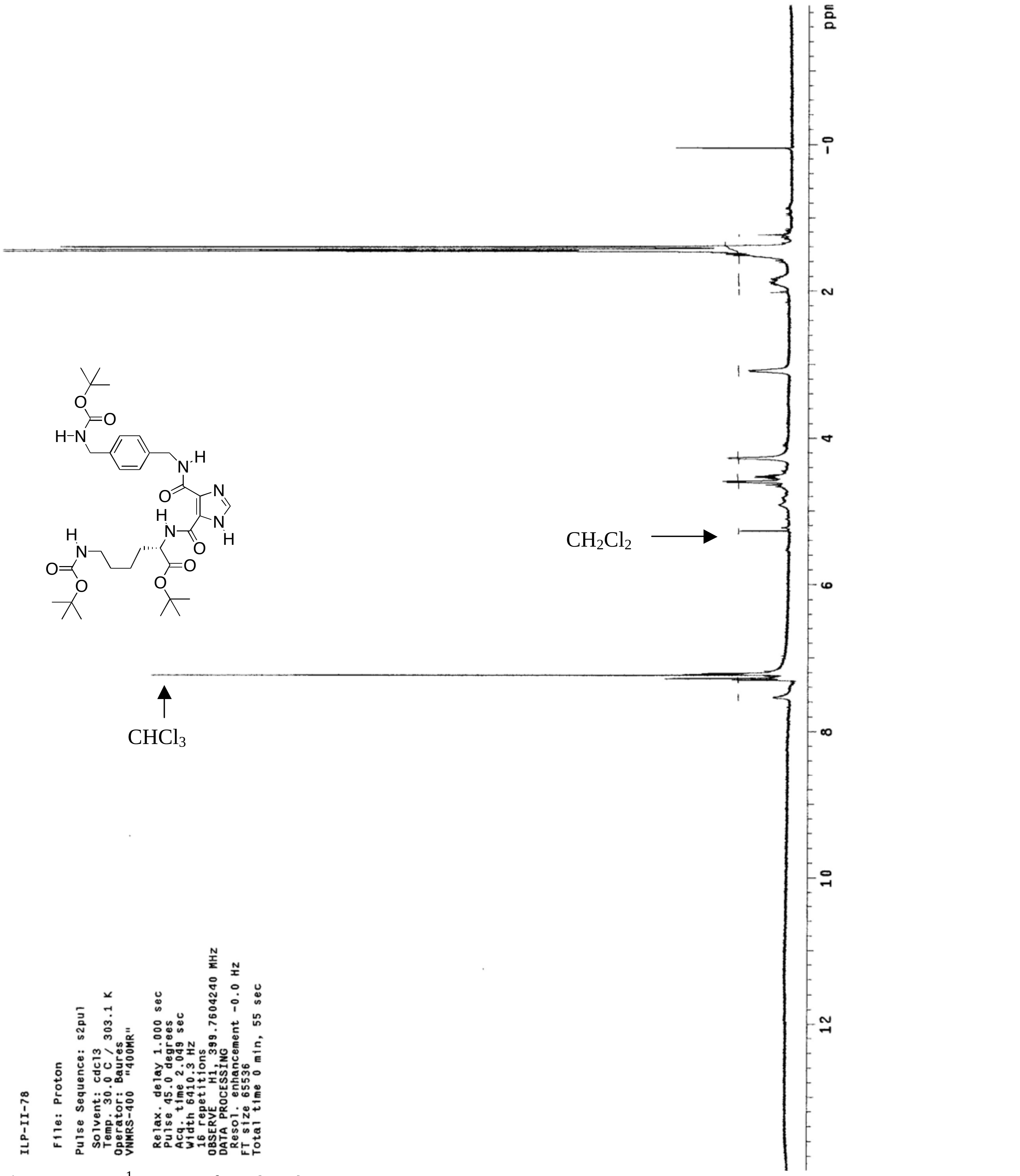


**Figure S161.** $^{1}$H-NMR for **5{**119}.

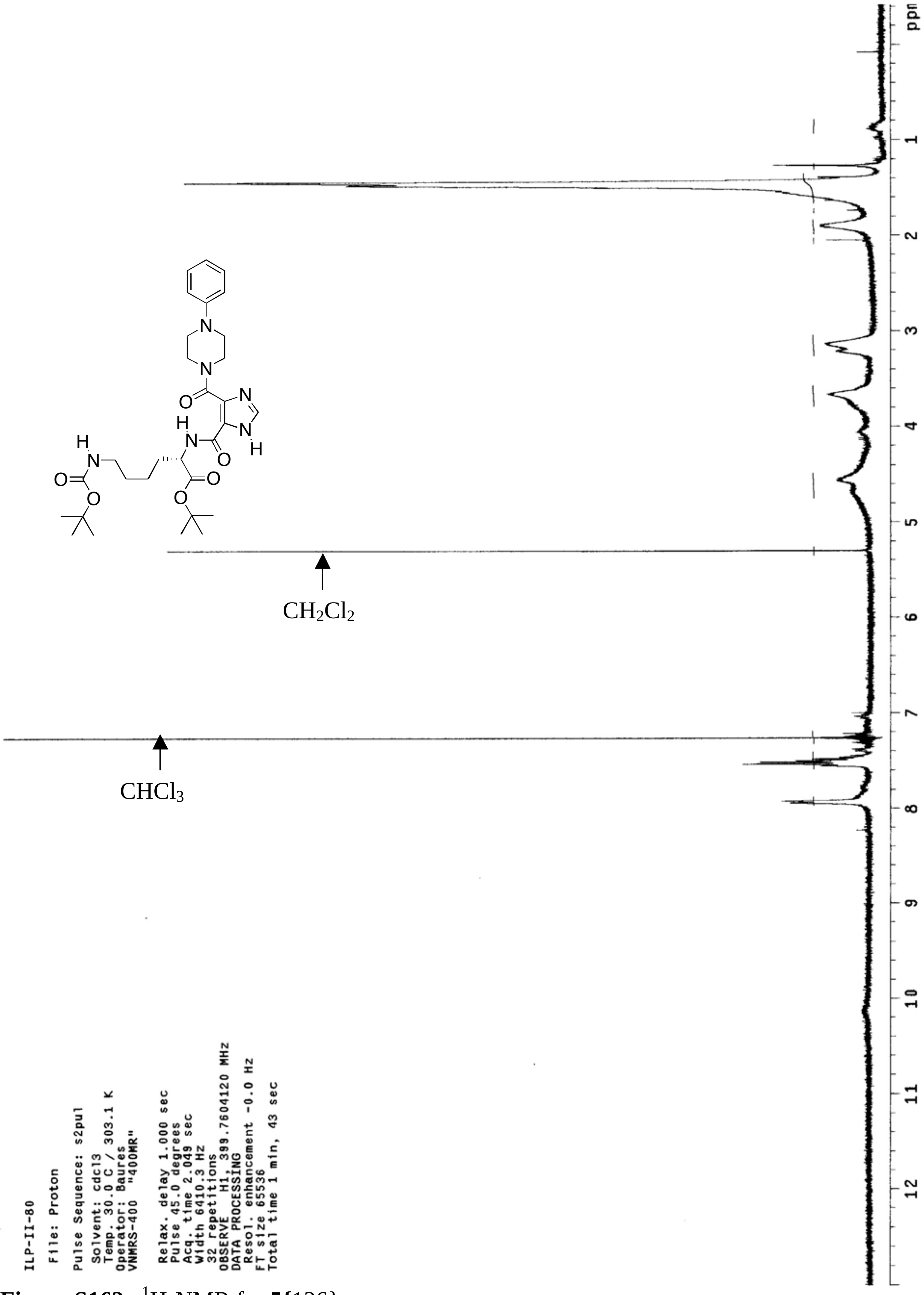


**Figure S162.** $^1$H-NMR for **5{**126}.

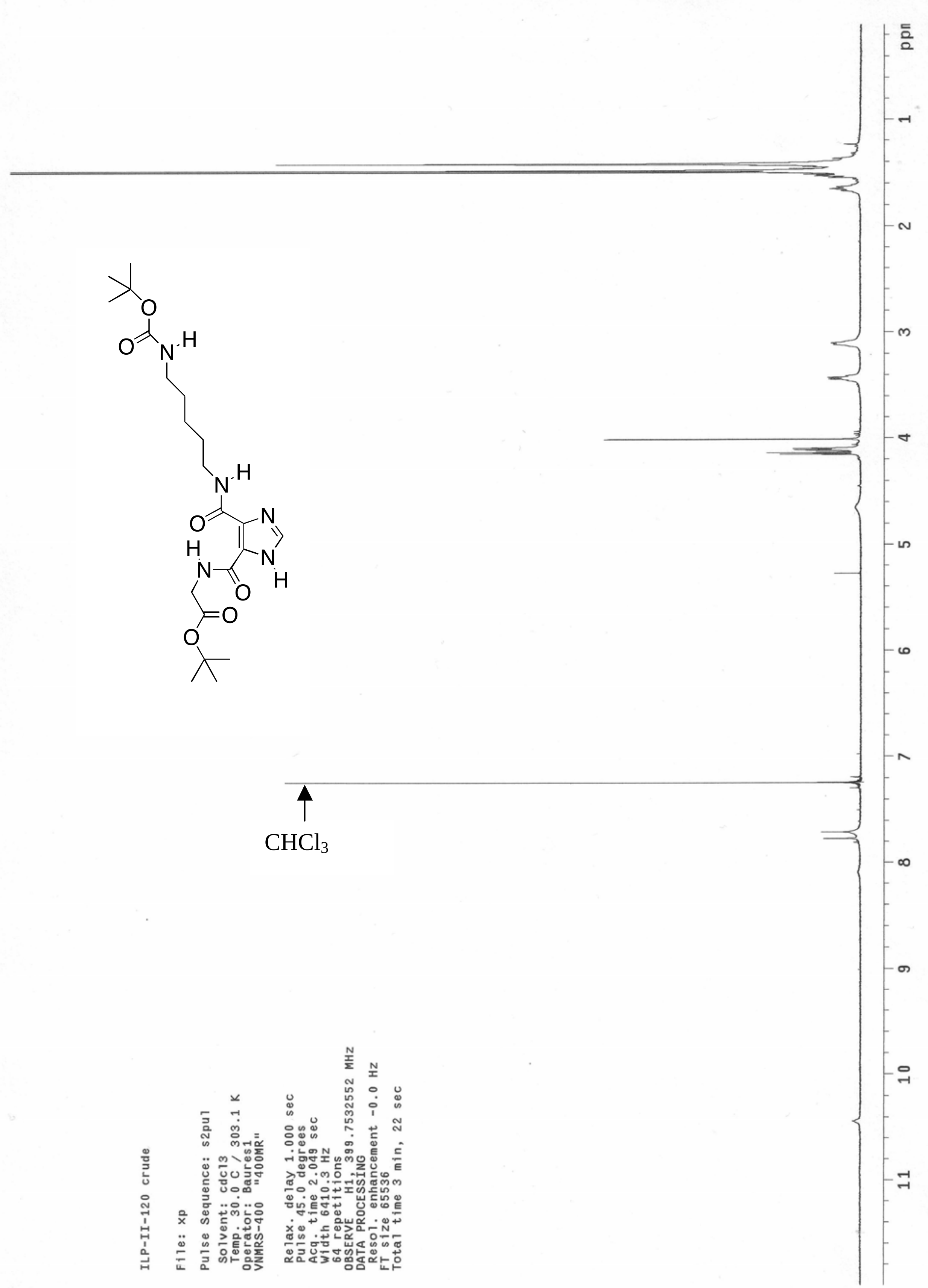


**Figure S163.** $^1$H-NMR for the crude reaction to yield **5{**2}.

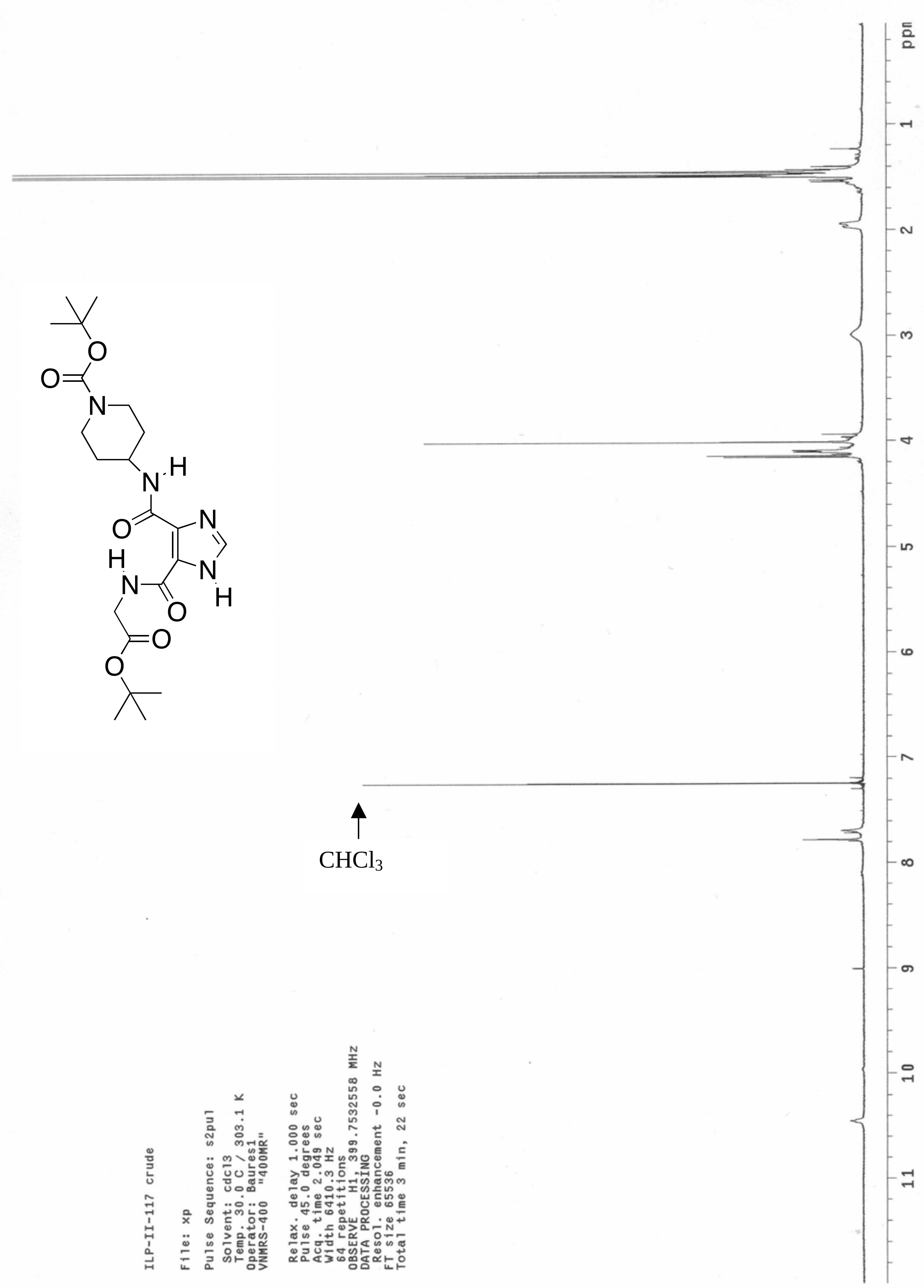


**Figure S164.** $^1$H-NMR for the crude reaction to yield **5{**6}.

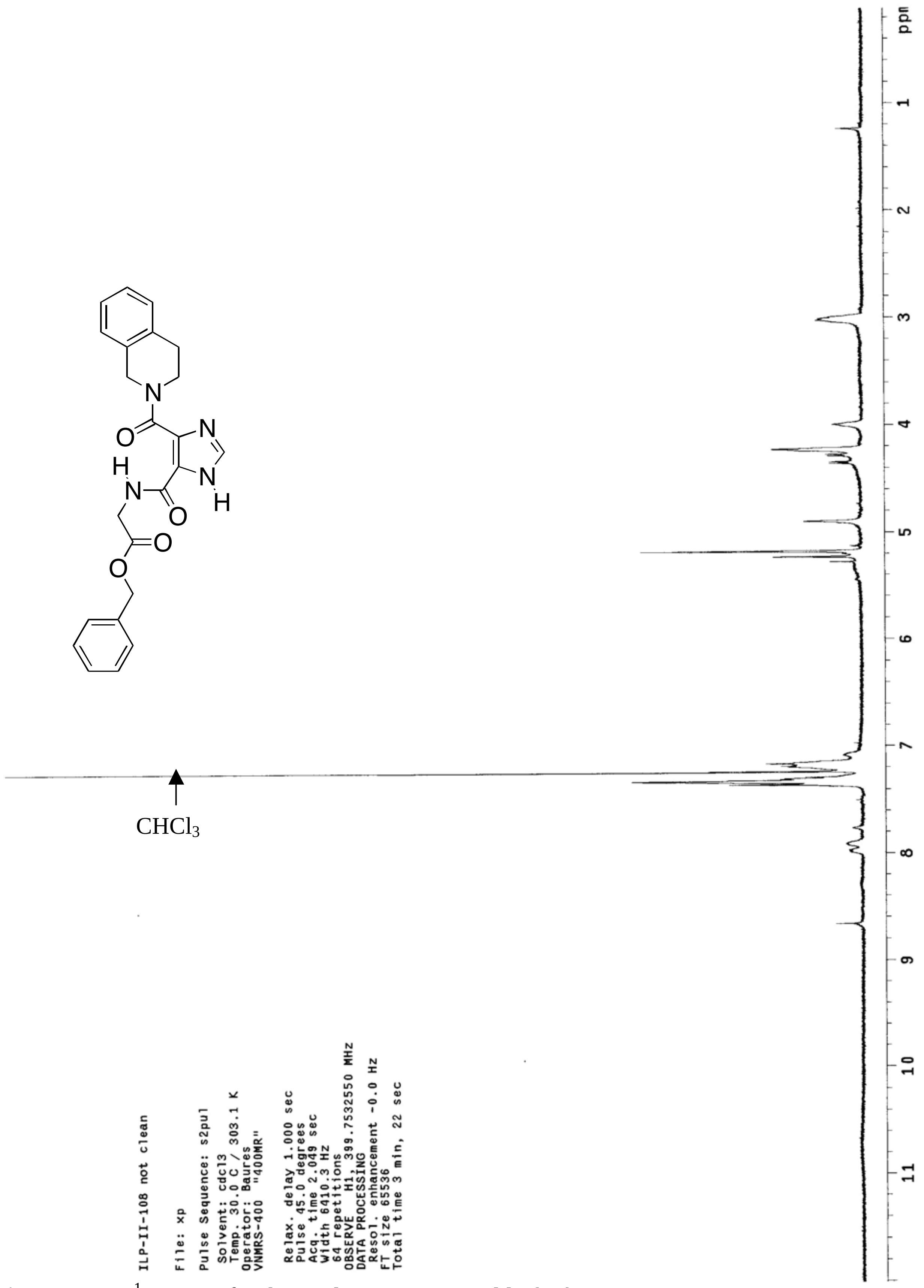


**Figure S165.** $^1$H-NMR for the crude reaction to yield **5{**26}.

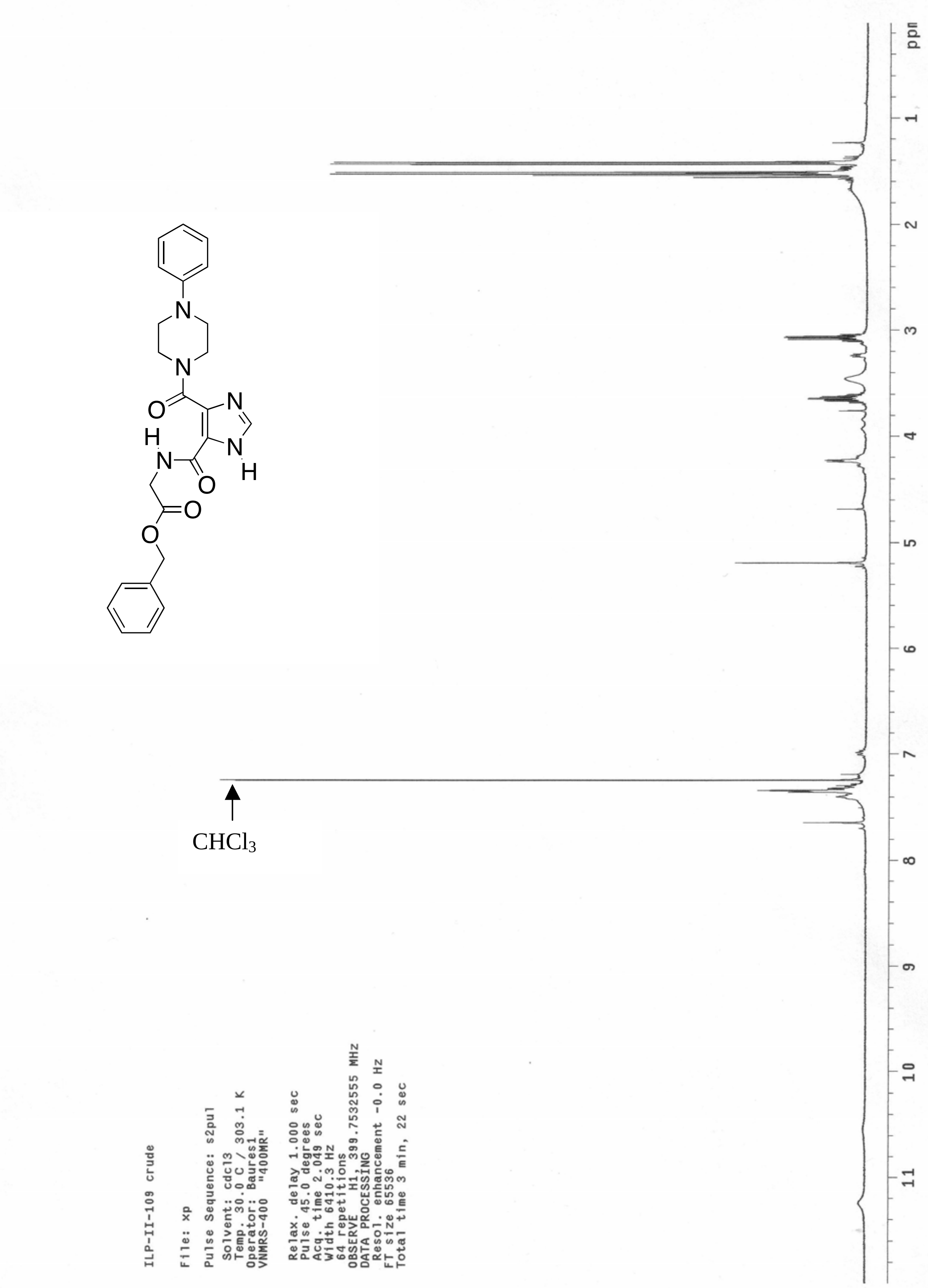


**Figure S166.** $^{1}$H-NMR for the crude reaction to yield **5{**28}.

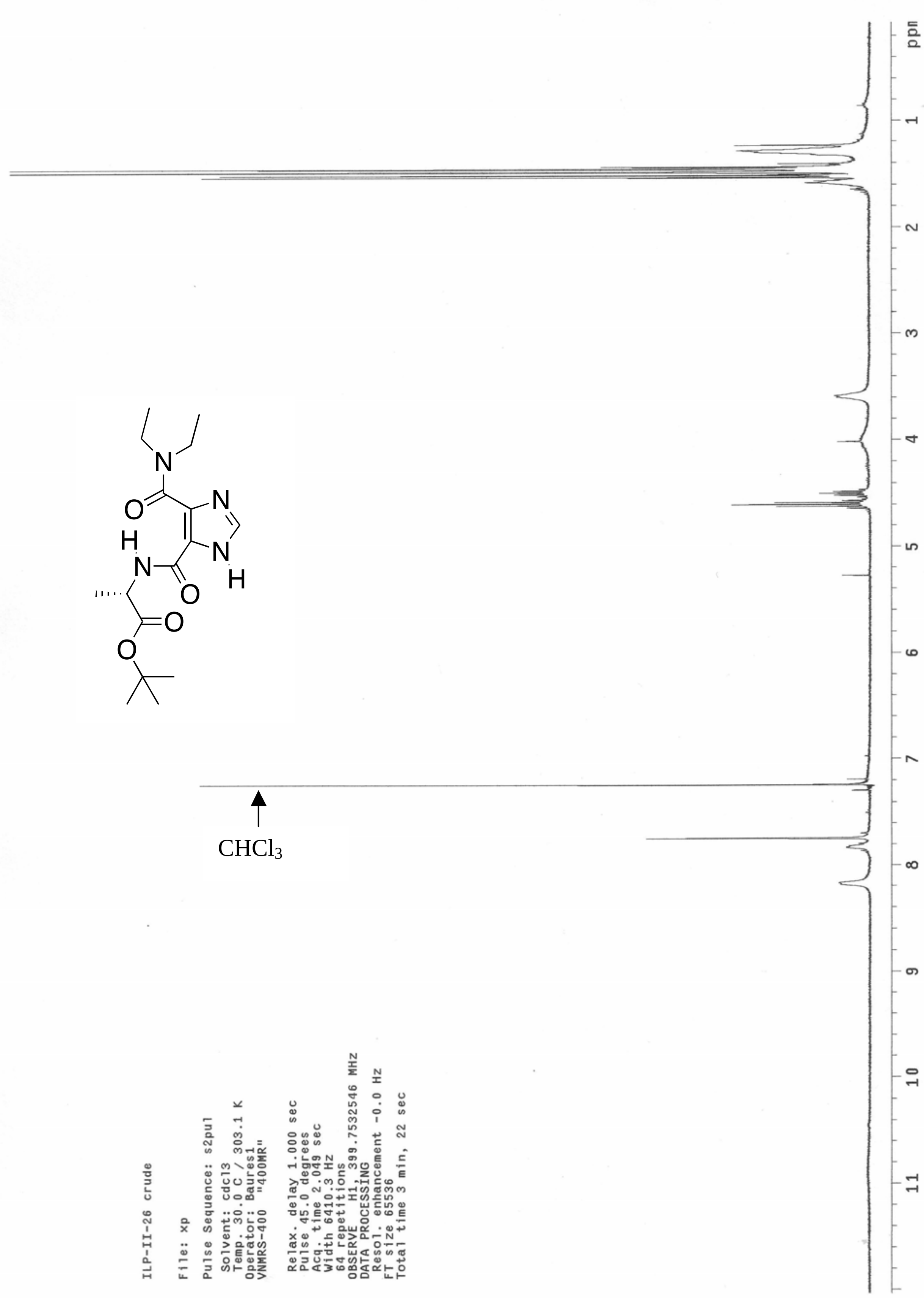


**Figure S167.** $^{1}$H-NMR for the crude reaction to yield **5{**38}.

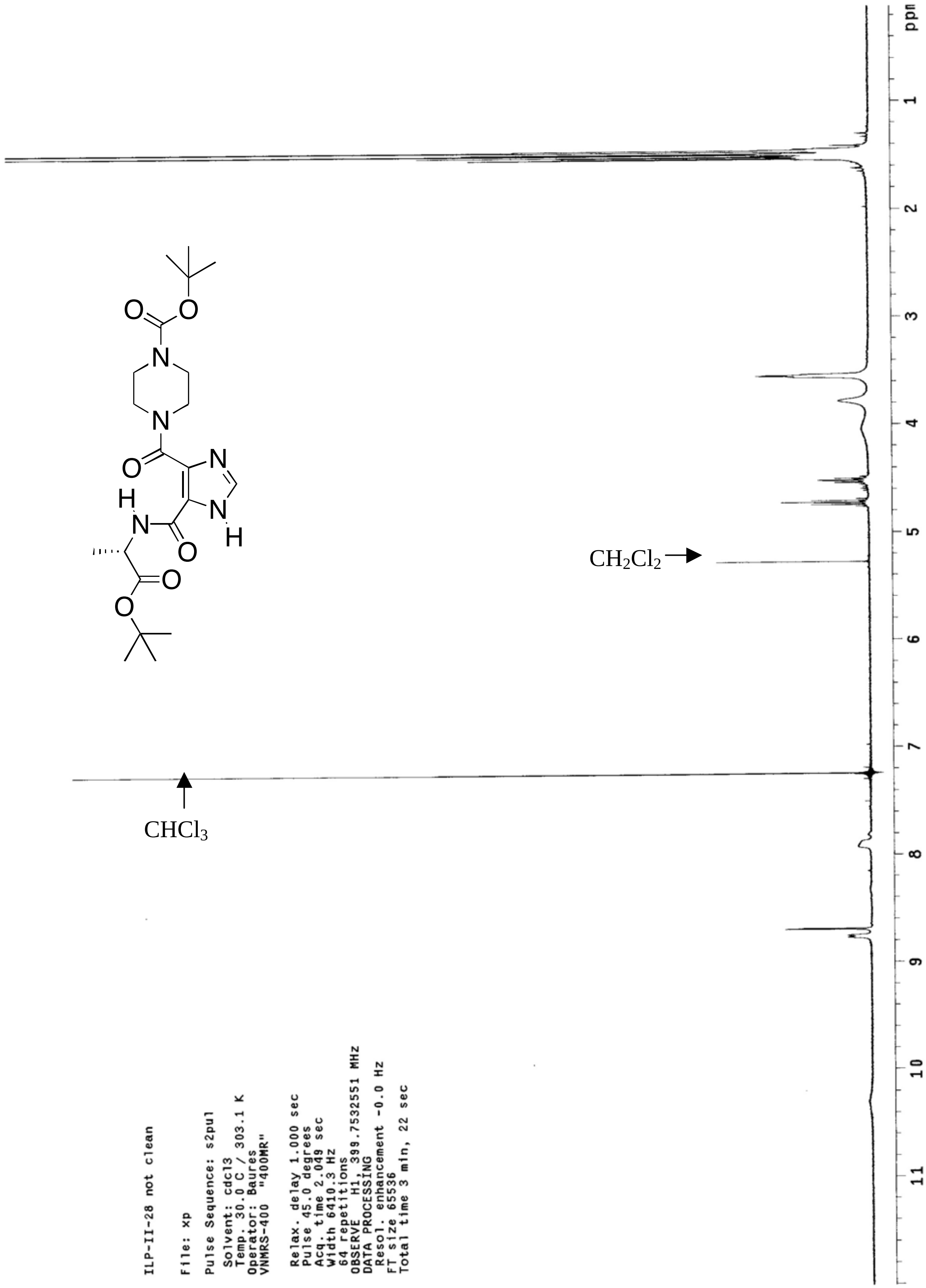


**Figure S168.** $^1$H-NMR for the crude reaction to yield **5{**39}.

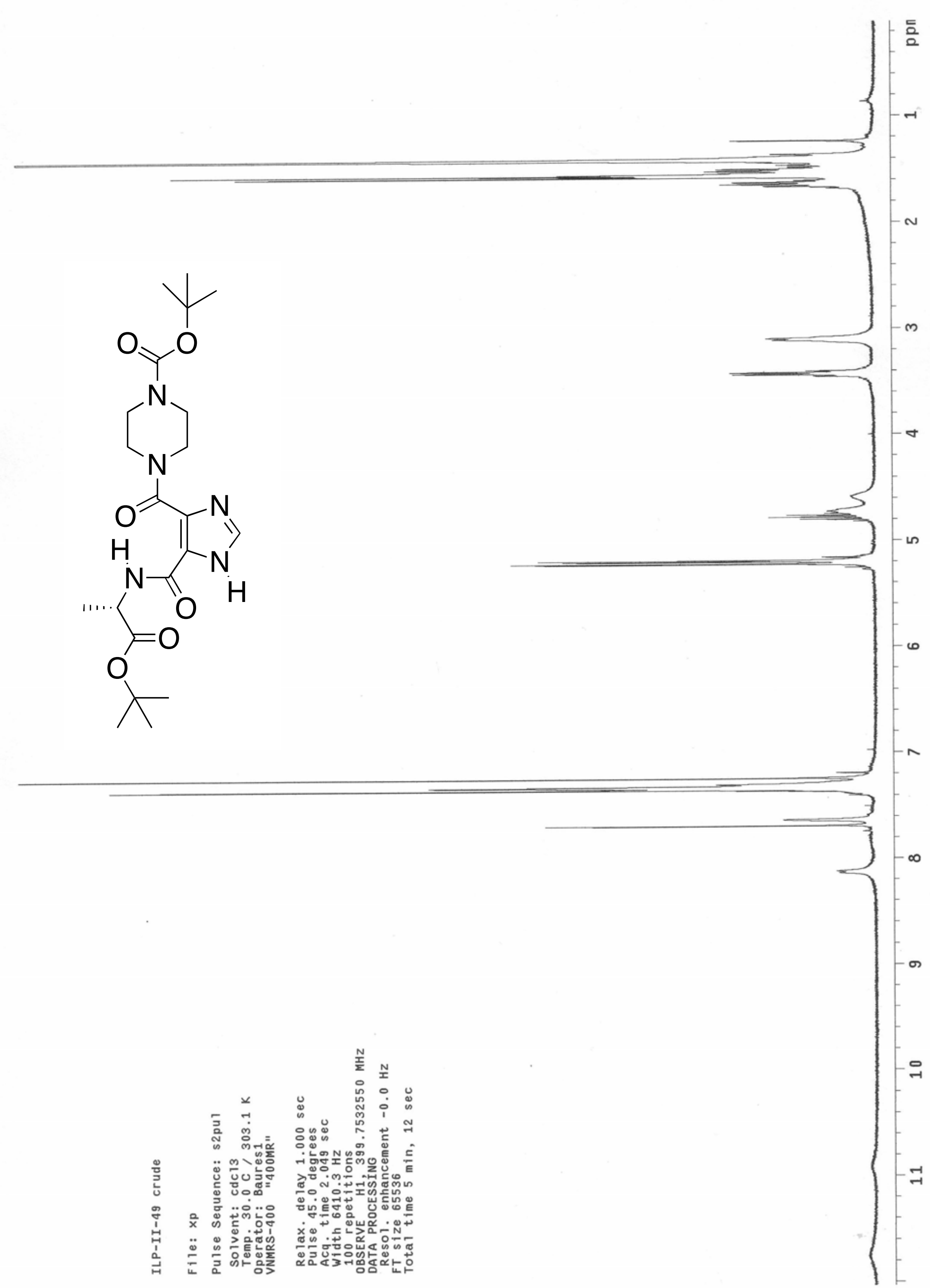


**Figure S169.** $^1$H-NMR for the crude reaction to yield **5{**44}.

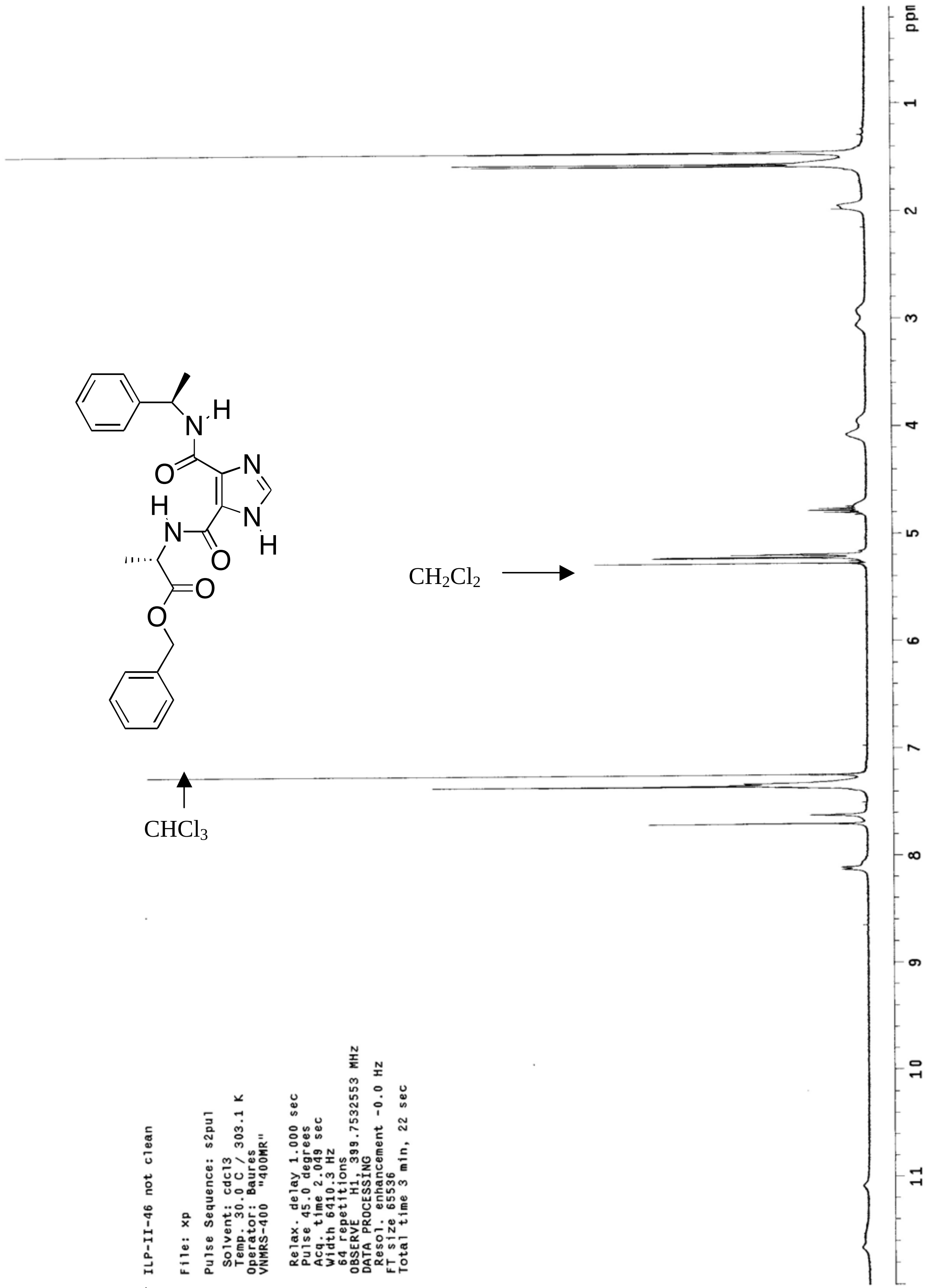


**Figure S170.** $^{1}$H-NMR for the crude reaction to yield **5{**48}.

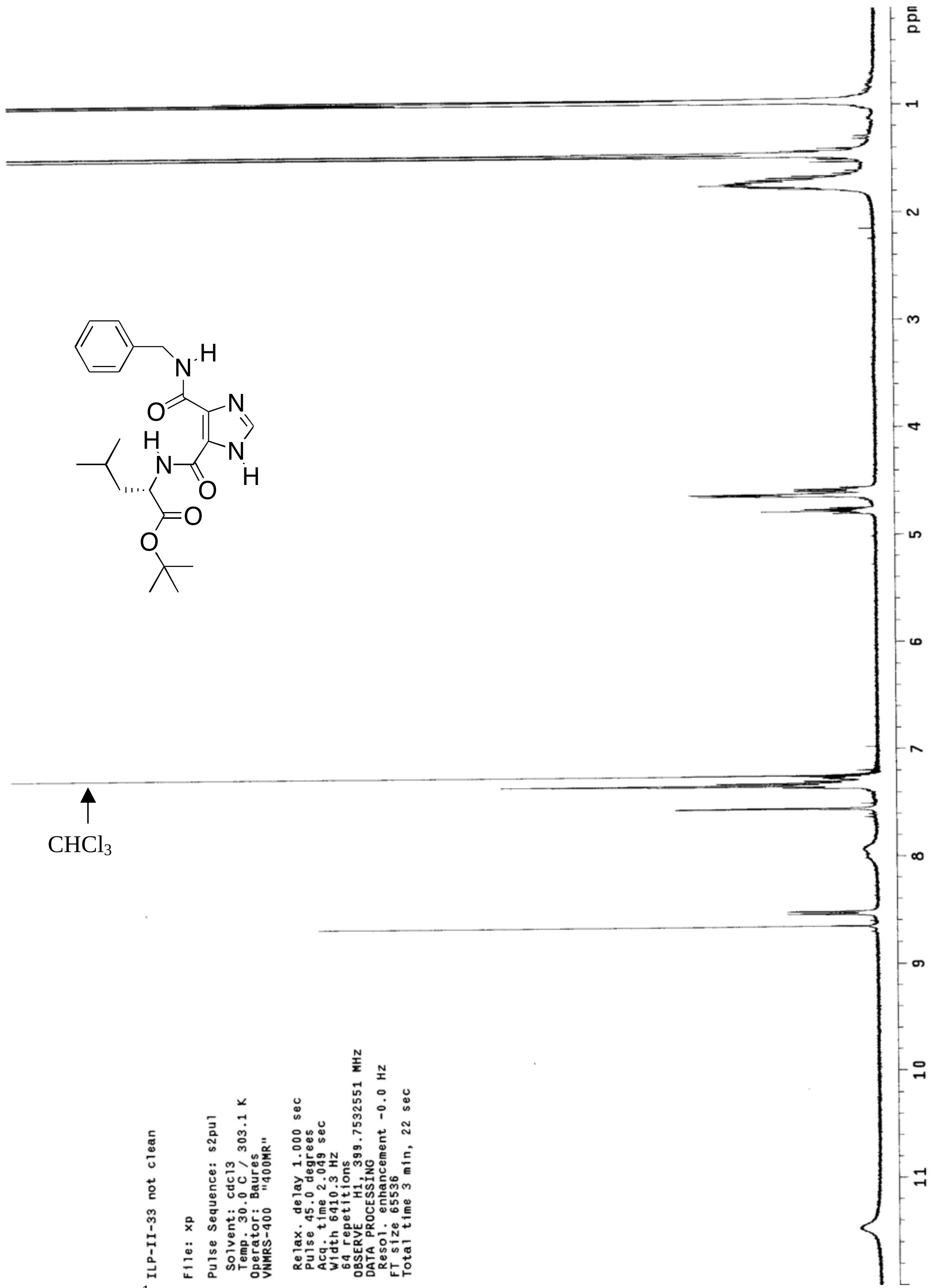


**Figure S171.** $^{1}$H-NMR for the crude reaction to yield **5{**59}.

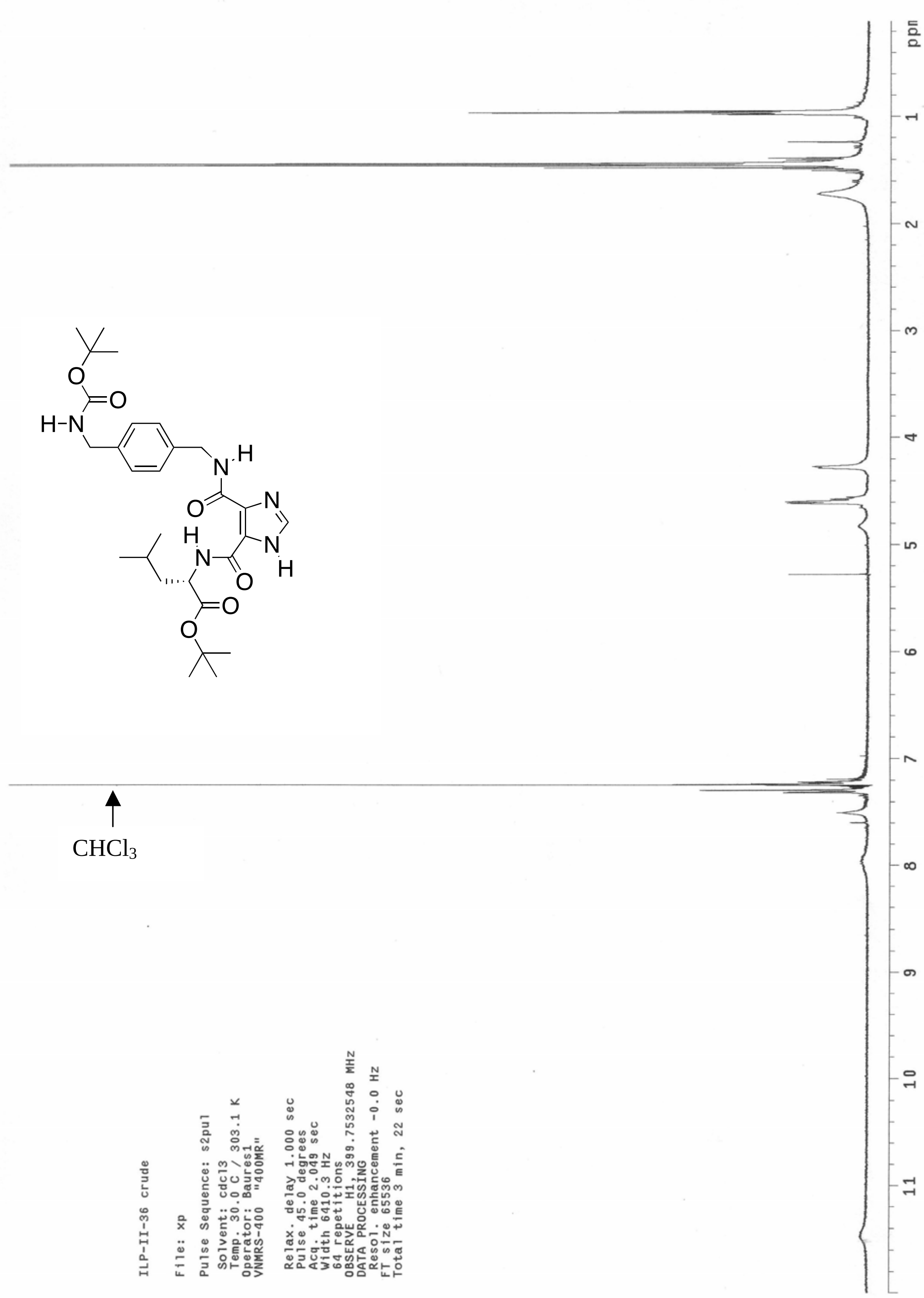


**Figure S172.** $^1$H-NMR for the crude reaction to yield **5{**63}.

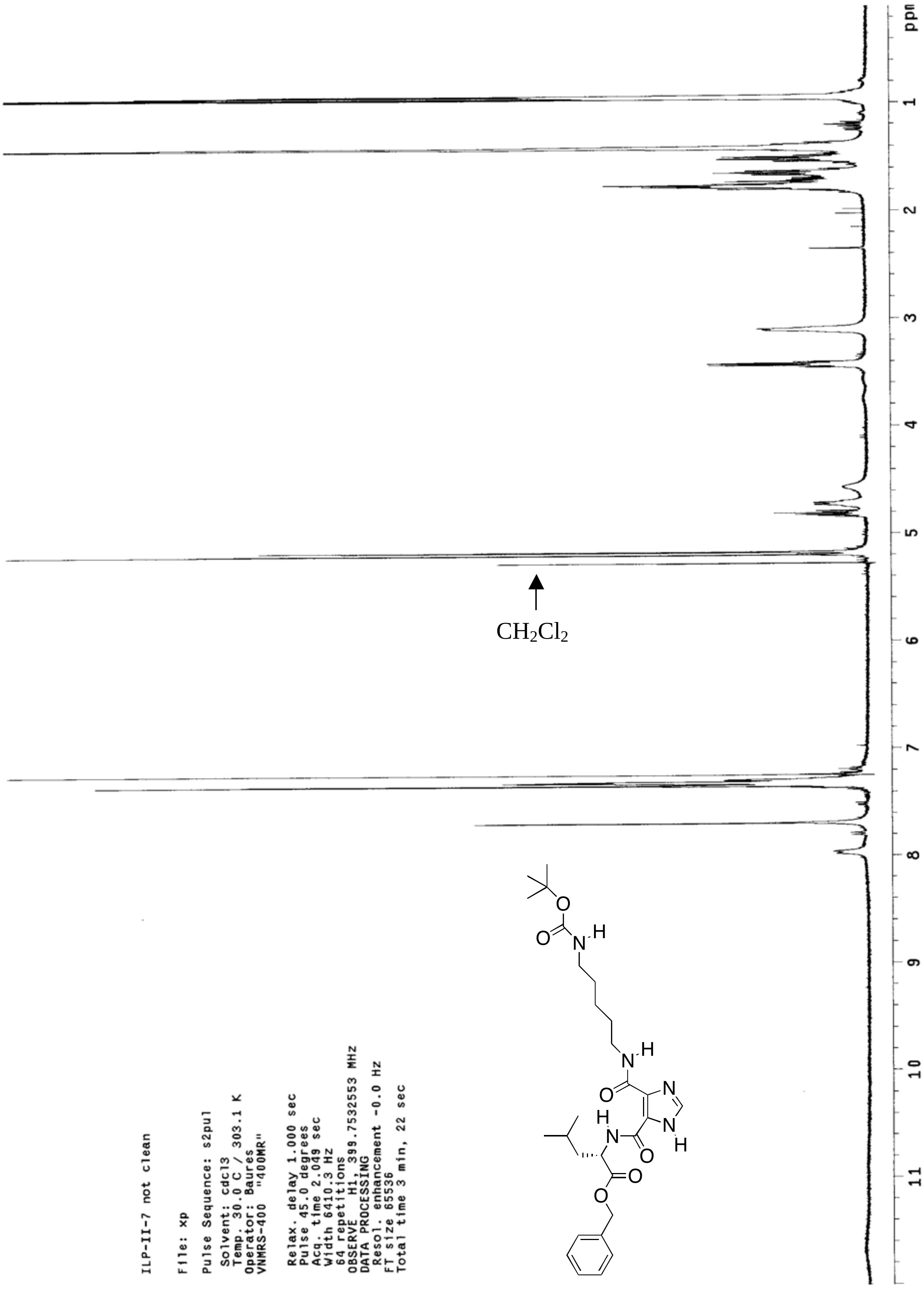


**Figure S173.** $^{1}$H-NMR for the crude reaction to yield **5{**72}.

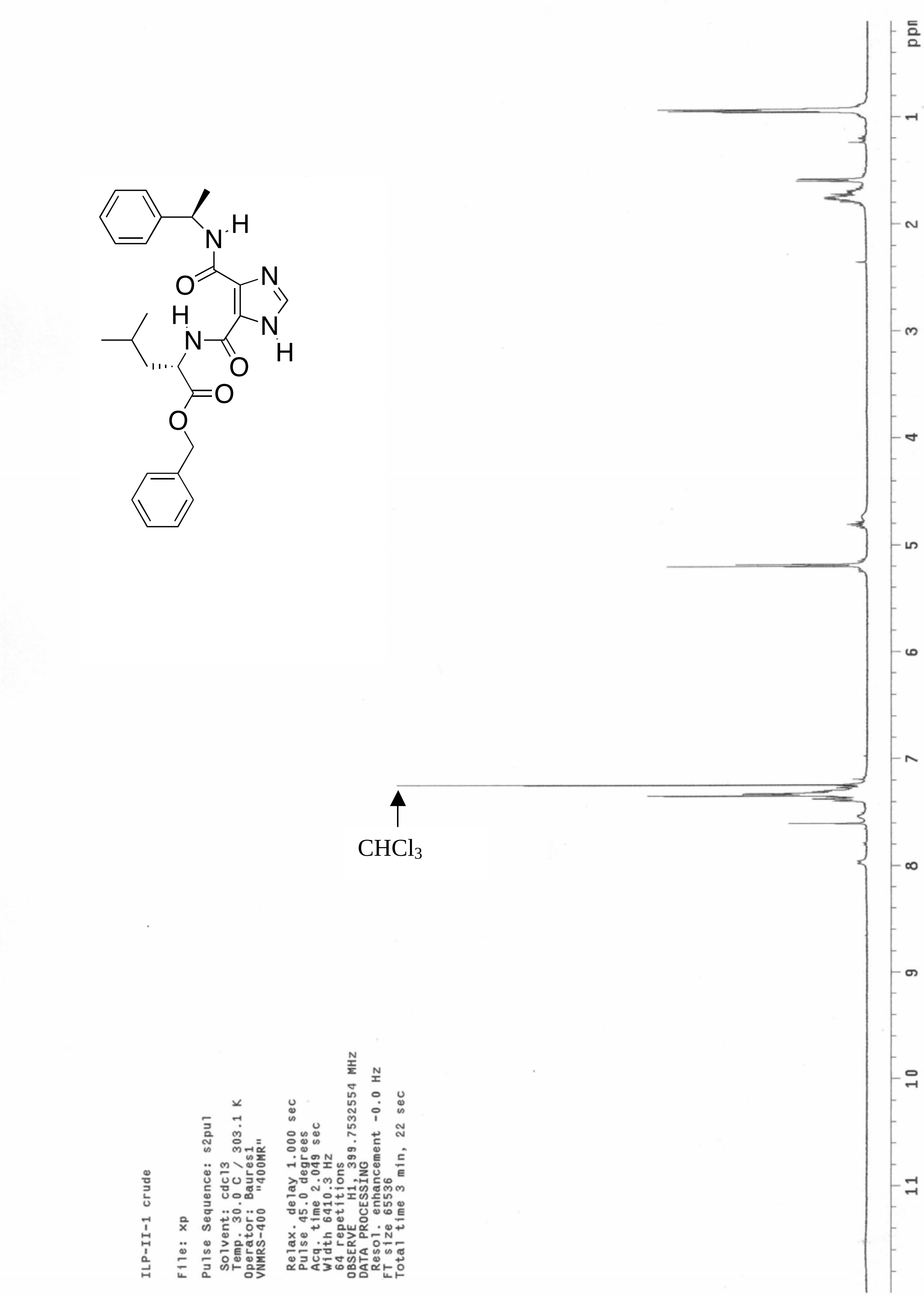


**Figure S174.** $^{1}$H-NMR for the crude reaction to yield **5{**74}.

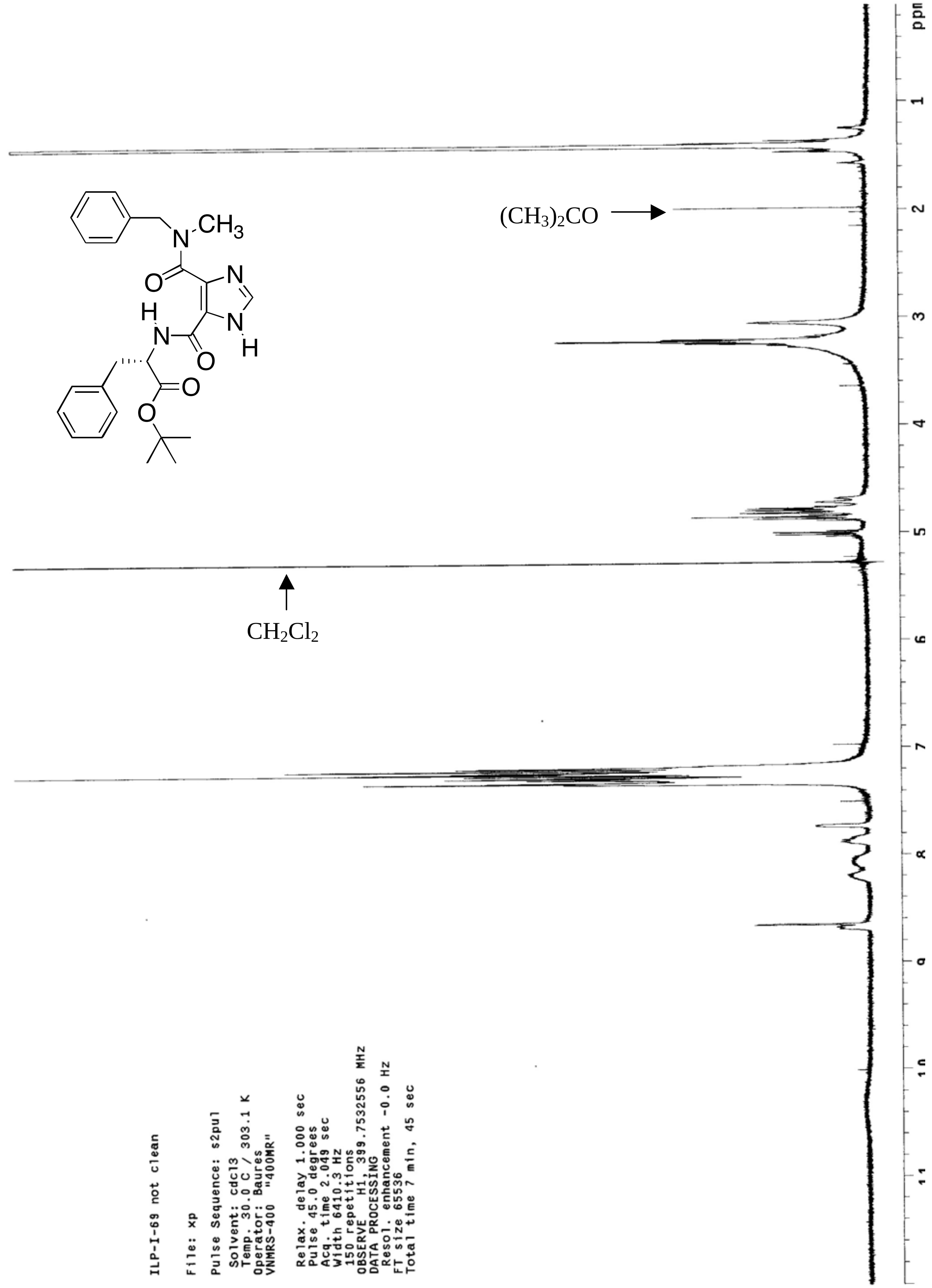


**Figure S175.** $^{1}$H-NMR for the crude reaction to yield **5{**93}.

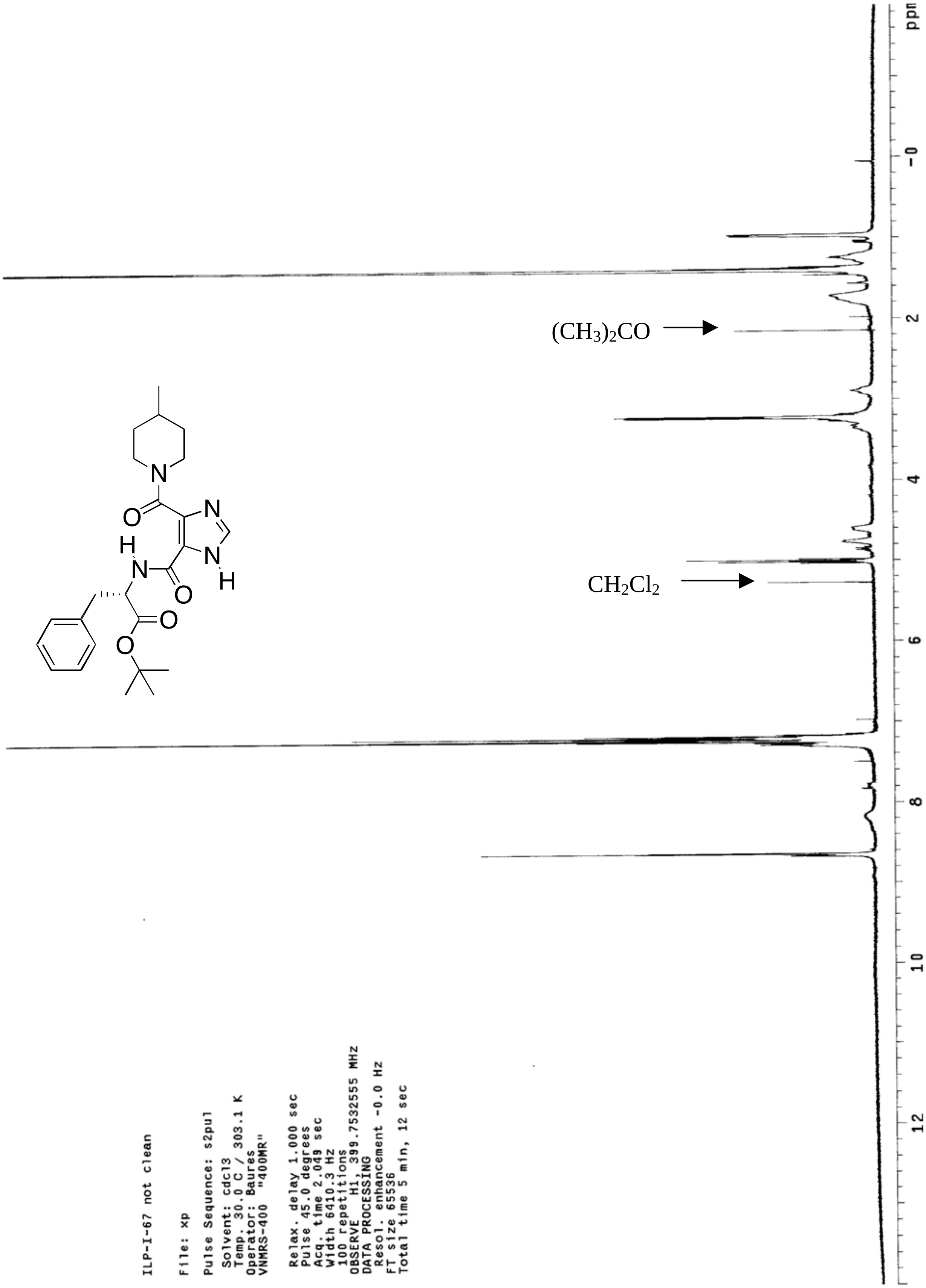


**Figure S176.** $^{1}$H-NMR for the crude reaction to yield **5{**97}.

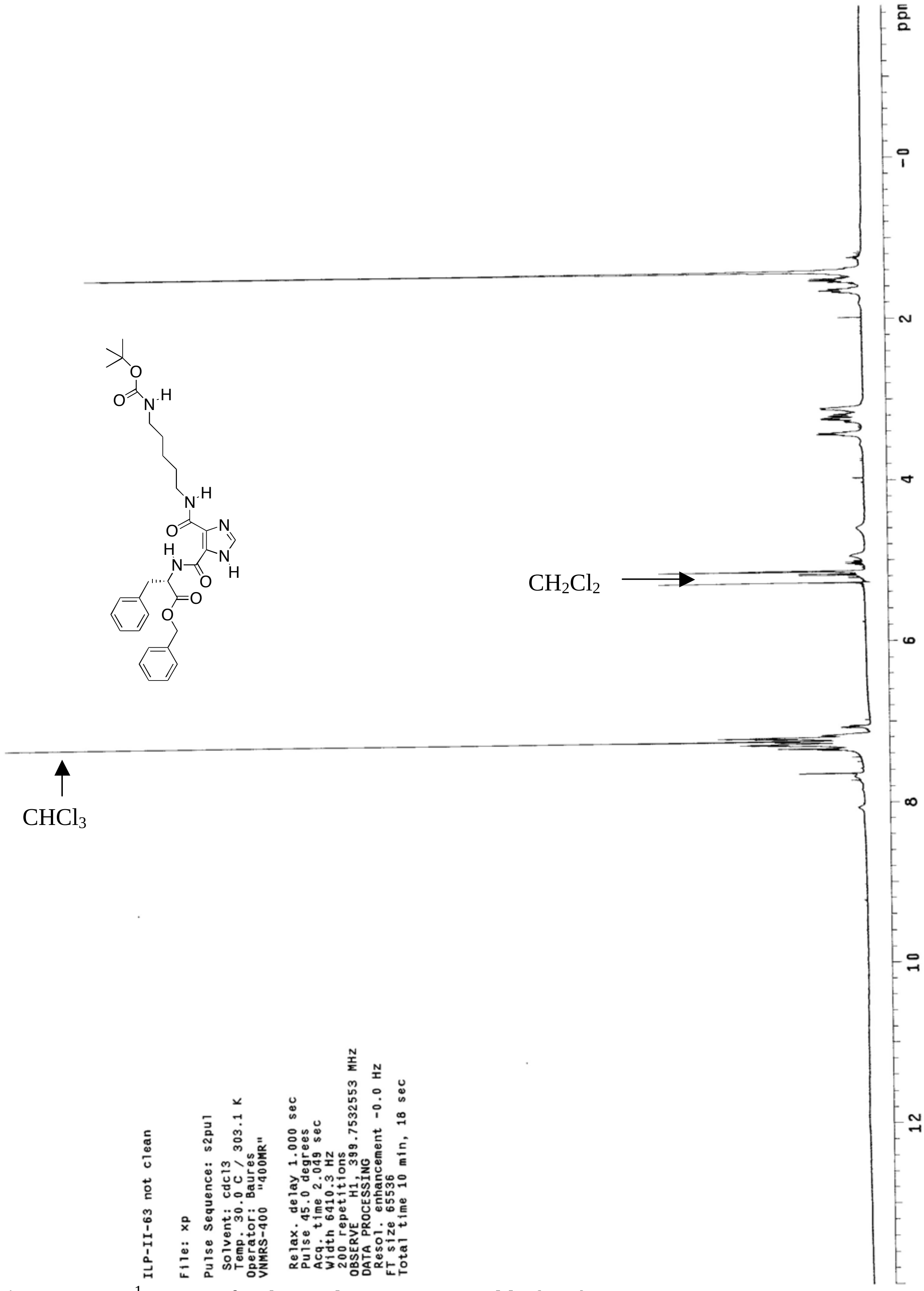


**Figure S177.** $^{1}$H-NMR for the crude reaction to yield **5{**100}.

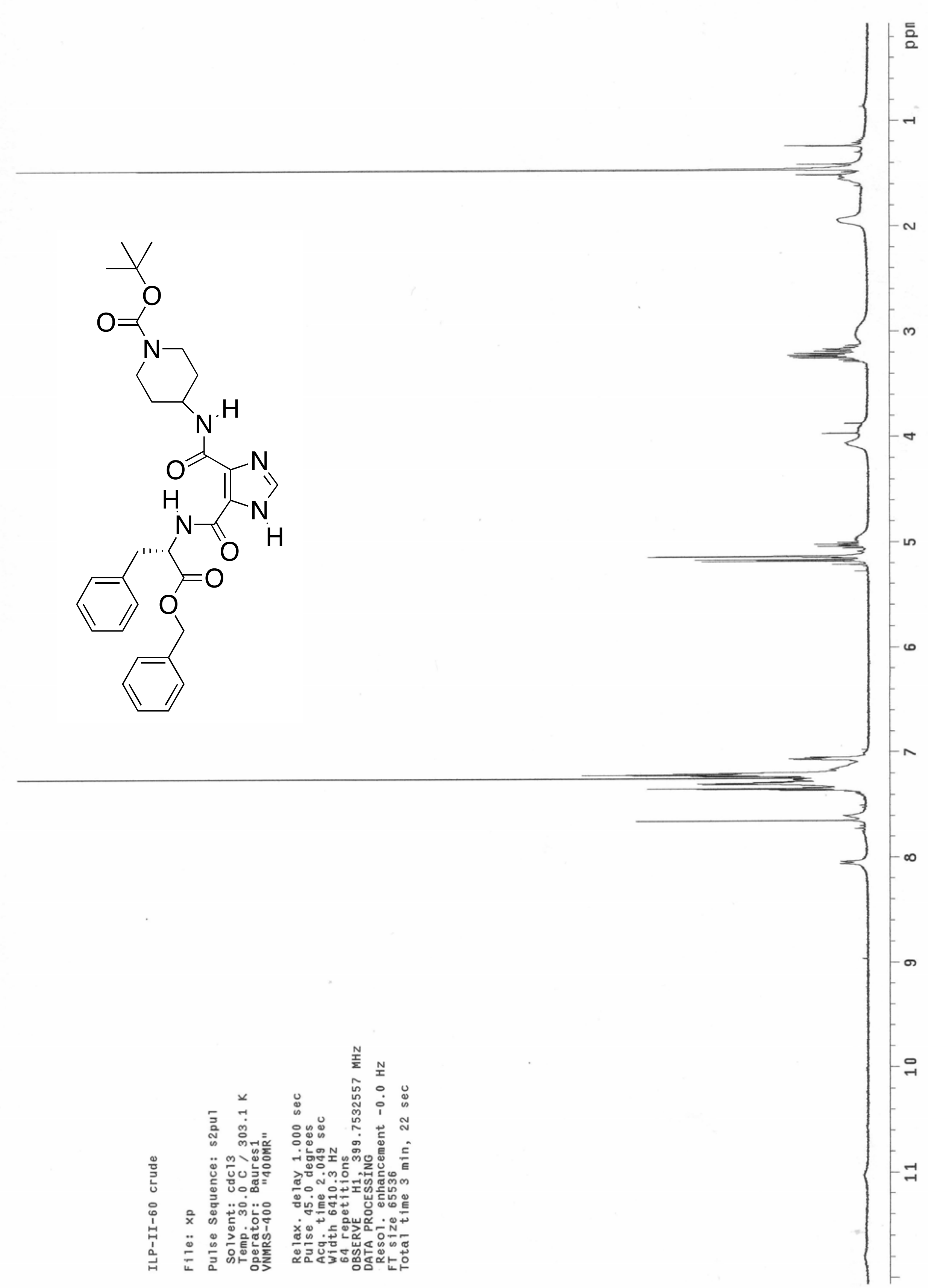


**Figure S178.** $^1$H-NMR for the crude reaction to yield **5{**104}.

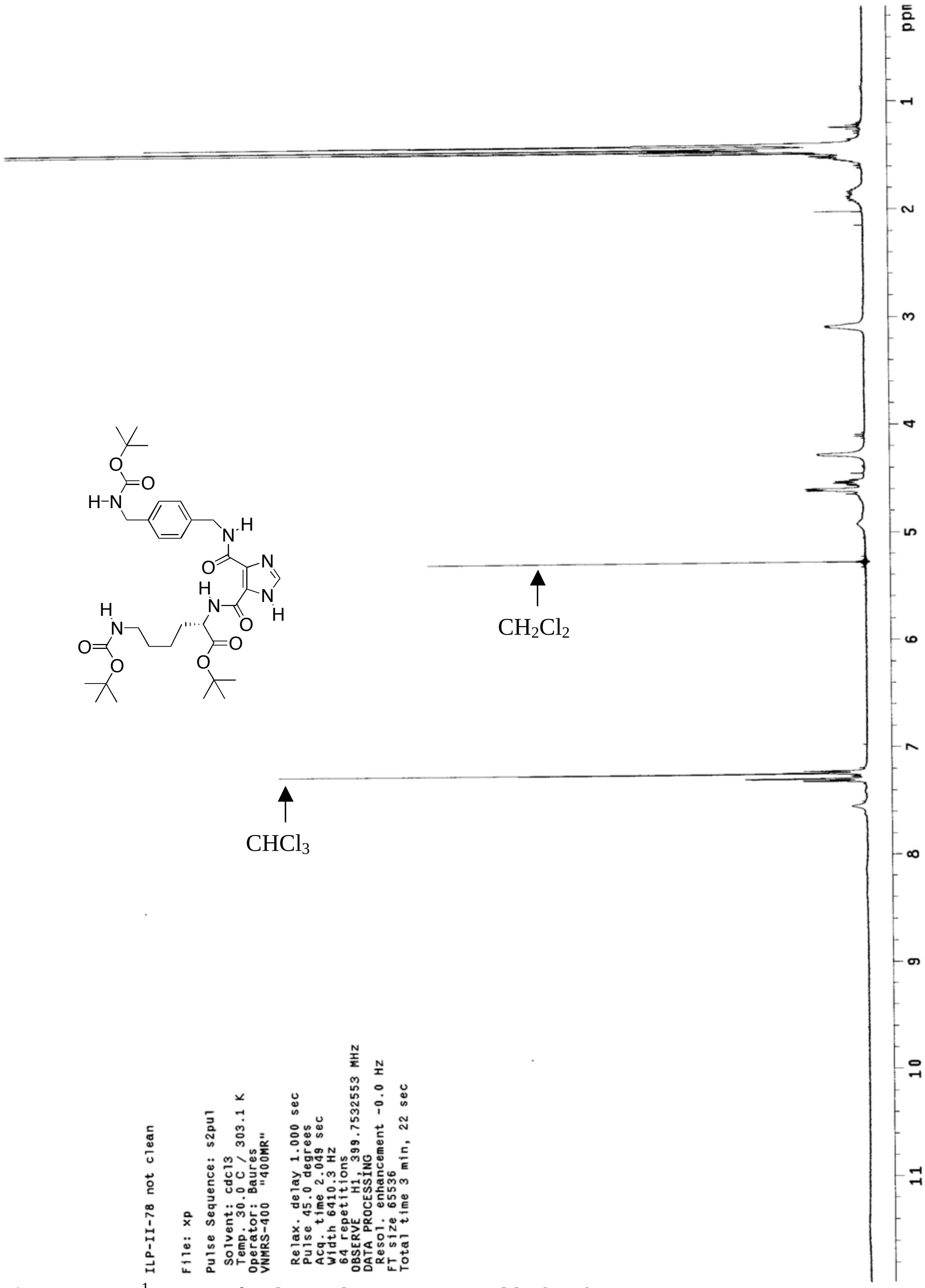


**Figure S179.** $^{1}$H-NMR for the crude reaction to yield **5{**119}.

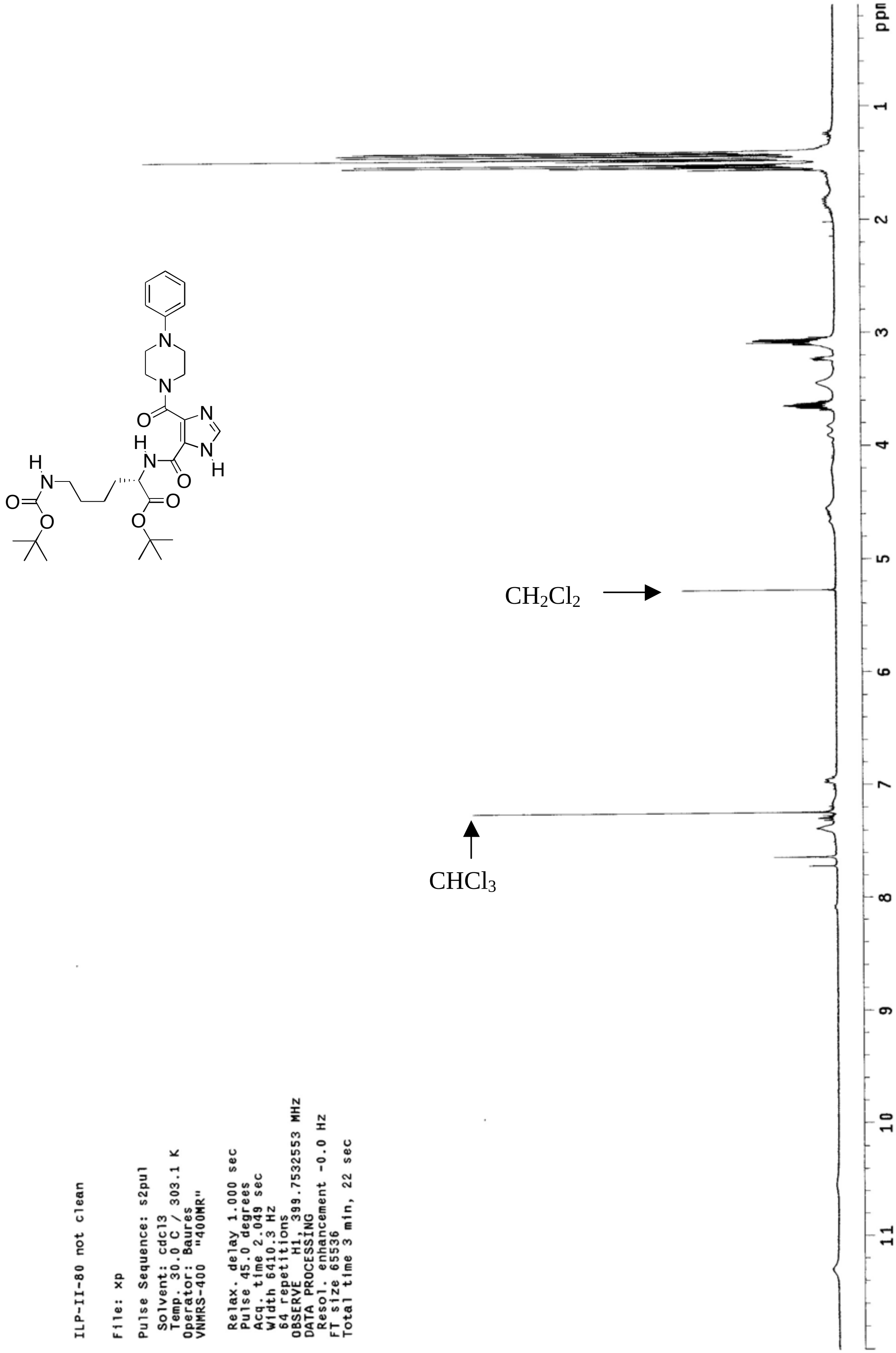


**Figure S180.** $^{1}$H-NMR for the crude reaction to yield **5{**126}.
